# Copper(II)‐Photocatalyzed Radical Anellation of Nitroalkanes with Alkenes or Alkynes for the Synthesis of Isoxazolines and Isoxazoles

**DOI:** 10.1002/anie.202509658

**Published:** 2025-08-01

**Authors:** Sunaina Sardana, Aryaman Pattanaik, Julia Rehbein, Oliver Reiser

**Affiliations:** ^1^ University of Regensburg Department of Organic Chemistry Universitätsstr. 31 93053 Regensburg Germany

**Keywords:** Anellation, Cu photocatalysis, Heterocycles, Radical pathway, α‐nitro radicals

## Abstract

The visible light‐mediated copper(II)‐catalyzed one‐step synthesis of isoxazolines and isoxazoles from readily available ethyl nitroacetate or phenyl nitromethane is reported. The developed protocol eliminates the need for substrate preactivation or additives, and offers an extensive scope of activated and unactivated alkenes and alkynes as coupling partners. Key intermediates for this formal [3 + 2]‐cycloaddition are α‐nitro radicals generated via photoinduced single‐electron oxidation of nitronates, contrasting the generation of such radicals via Cu(I)‐photocatalysis by a reductive pathway, which shows a different reaction pattern in the coupling with alkenes.

## Graphical abstract

We present a Cu(II)‐photocatalyzed method that employs commercially available nitroalkanes for the synthesis of isoxazolines and isoxazoles from alkenes and alkynes, respectively. Detailed mechanistic investigations revealed that the broad substrate scope, encompassing both activated and unactivated alkenes and alkynes, is attributable to a radical pathway involving α‐nitro alkyl radicals.

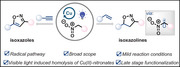

## Introduction

Photoredox catalysis proceeds via single‐electron reduction or oxidation, generating radicals for diverse synthetic transformations.^[^ ^1^ ^]^

**Figure 1:**
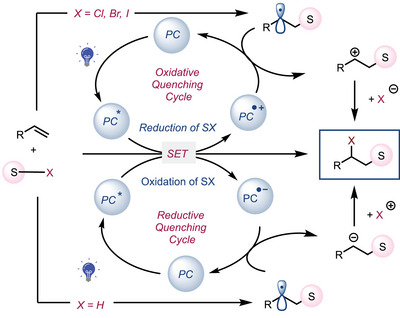
Radical addition to alkenes initiated by single‐electron reduction or oxidation of S–X.

Both reaction modes can form the same radical intermediate (S•), which might add to alkenes followed by electron transfer between the oxidized or reduced photocatalyst (PC) and the radical intermediate.^[^ ^2^, ^3^, ^4^, ^5^, ^6^ ^]^ The nature of substituent X determines whether electron transfer occurs via reduction or oxidation with a suitable photocatalyst. For instance, sulfonyl radicals add to alkenes, resulting in chlorosulfonylation^[^ ^7^ ^]^ from sulfonyl chlorides via the oxidative quenching cycle or hydrosulfonylation from sulfinates or sulfonamides via the reductive quenching cycle.^[^ ^8^, ^9^, ^10^ ^]^ We questioned if the PC's interaction with radical intermediates can fundamentally change the reaction pathway beyond the simple addition process outlined in Figure 1.

The interaction of radical intermediates has been proposed most prominently with copper‐based photocatalysts either by Cu(I)‐substrate assemblies that can be selectively excited by light or by Cu(II)‐substrate assemblies that interact with transient radicals via radical ligand transfer or Cu(III)/reductive elimination cascades.^[^ ^11^, ^12^, ^13^, ^14^ ^]^

**Figure 2:**
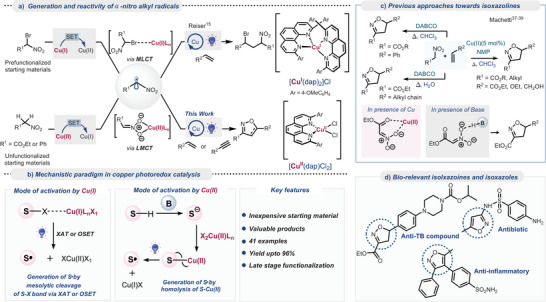
State of the art and divergent reactivity of Cu photocatalysts. a) Generation and reactivity of α‐nitro alkyl radicals. b) Mechanistic paradigms in copper photoredox catalysis. c) Previous approaches toward isoxazolines. d) Bio‐relevant isoxazolines and isoxazoles.

We previously reported the selective atom transfer radical addition (ATRA) of bromonitroalkanes onto alkenes via α‐nitro alkyl radicals with a Cu(I)‐photocatalyst (Figure 2a, top).^[^ ^15^ ^]^ In contrast, we show here that α‐nitro alkyl radicals generated by a Cu(II)‐photocatalyst lead to a formal [3 + 2]‐cycloaddition, yielding isoxazolines or isoxazoles under mild conditions (Figure 2a, bottom) rather than the expected ATRA products. This distinction suggests the role of copper's oxidation state in modulating reaction pathways from the same radical intermediate and coupling partner (vide infra, mechanistic discussion).

Given the significance of isoxazolines or isoxazoles,^[^ ^16^, ^17^, ^18^, ^19^, ^20^ ^]^ various strategies for their synthesis have been developed,^[^ ^21^, ^22^, ^23^, ^24^, ^25^, ^26^, ^27^, ^28^, ^29^, ^30^, ^31^, ^32^, ^33^ ^]^ including photochemical variants.^[^ ^34^, ^35^, ^36^ ^]^ Relevant to this work, pioneering studies by Machetti and coworkers (Figure 2c) showed that activated nitroalkanes can be directly cyclized with styrene, phenyl acetylene, or norbornene in the presence of a base under thermal conditions (60–80 °C).^[^ ^37^, ^38^ ^]^ This group also noticed the accelerating effects of Cu(II) salts as an additive to achieve isoxazoline formation between nitroalkanes and alkenes.^[^ ^39^ ^]^ In both variants, the formation of the nitronate 1,3‐dipole is facilitated through the interaction of the base or Cu(II) acting as a Lewis acid (Figure 2c), which subsequently undergoes [3 + 2]‐cycloaddition with alkenes via a concerted pathway. We demonstrate in this study that by moving to a radical pathway through the photooxidation of nitroalkanes, the resulting α‐nitro radicals readily react with alkenes and alkynes to afford the corresponding isoxazolines or isoxazoles. Notably, this protocol not only makes the title reaction possible at room temperature in shorter reaction times but also broadens the scope to substrates that are not successful in the concerted manifold, even at elevated temperatures.

## Results and Discussion

The light‐mediated oxoalkylation of styrenes with malonates in the presence of oxygen was achieved by utilizing Cu^II^(dap)Cl_2_ (dap = 2,9‐bis(para‐anisyl)‐1,10‐phenanthroline as a photocatalyst without the need of employing a base.^[^ ^40^ ^]^ Expecting an analogous outcome, we reacted styrene (**1a**) and ethyl nitroacetate (ENA) (**2a**) under identical conditions (Table 1, entry 1); however, the formation of **3a’** was not observed.

**Table 1:** Optimization of the Reaction Conditions^a)^

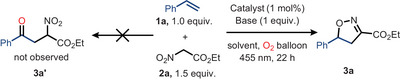				
Entry	Photocatalyst	Base	Sovent	Yield^b)^
1	Cu(dap)Cl_2_	–	MeCN	traces
2	Cu(dap)Cl_2_	DABCO	MeCN	22
3	Cu(dap)Cl_2_	Quinuclidine	CHCl_3_	36
4	Cu(dap)Cl_2_	Quinuclidine	CHCl_3_	52
5^c)^	Cu(dap)Cl_2_	Quinuclidine	CHCl_3_	76
6^d)^	Cu(dap)Cl_2_	Quinuclidine	CHCl_3_	nr
7^e)^	Cu(dap)Cl_2_	Quinuclidine	CHCl_3_	nr
8	–	Quinuclidine	CHCl_3_	nr
9	[Ir‐F]	Quinuclidine	CHCl_3_	nr
10	4‐CzIPN	Quinuclidine	CHCl_3_	nr
11^e)^	–	DABCO/r.t.	CHCl_3_	traces
12^e)^, ref. [37]	–	DABCO/60 °C/40h	CHCl_3_	74
13^e)^, ref. [39]	Cu(OAc)_2_	NMP/60 °C/40h	CHCl_3_	nr

^a)^ Styrene (**1a**, 0.20 mmol, 1.0 equiv.), ethyl nitroacetate (**2a**, 0.30 mmol, 1.5 equiv.), Base (0.20 mmol, 1.0 equiv.), Photocatalyst (20 µmol, 1.0 mol%), solvent (1.5 mL, 0.13 M), irradiation at 455 nm under oxygen atmosphere (O_2_ balloon) for 22 h at room temperature

^b)^ 1H‐NMR yield using 1,1,2,2‐tetrachloroethane as an internal standard

^c)^ Schlenk Blue LED Set up (see S.I.)

^d)^ under N_2_.

^e)^ no irradiation. nr = no reaction.

**Scheme 1:**
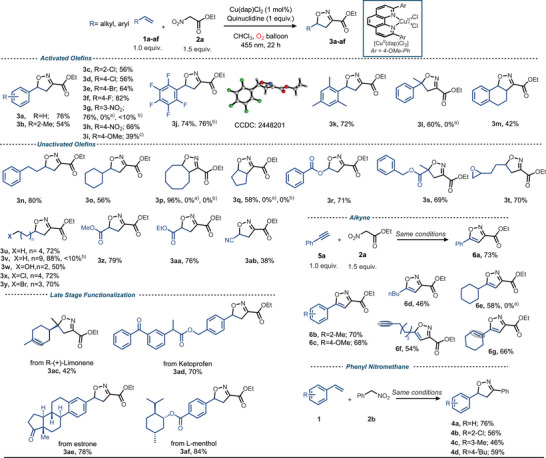
Cu(II)‐photocatalyzed synthesis of isoxazolines and isoxazoles: Olefin (0.20 mmol, 1.0 equiv.), ethylnitroacetate (0.30 mmol, 1.5 equiv.), Quinuclidine (0.20 mmol, 1.0 equiv.), catalyst (20 µmol, 1.0 mol%) in CHCl_3_ (1.5 mL, 0.13 M), Irradiation at 455 nm under an oxygen atmosphere (O_2_ balloon) for 22 h at room temperature. a) Ref. ^[^ ^39^ ^]^ ethyl nitroacetate (2.5 equiv.), alkene (1.0 equiv.), Cu(OAc)_2_ (5 mol%), NMP (0.5 equiv.), CHCl3 (1.4 mL), 40 h at 60 °C. b) Ref.^[^ ^37^ ^]^ ethyl nitroacetate (2.5 equiv.), alkene (1.0 equiv.), DABCO (0.5 equiv.), CHCl_3_ (1.4 mL), 40 h at 60 °C. c) The product was isolated as an inseparable mixture with (4‐methoxyphenyl)(5‐(4‐methoxyphenyl)‐4,5‐dihydroisoxazol‐3‐yl)methanone (12%, see S.I. for more details).

Reasoning that the oxidation of **2a** would proceed via its nitronate anion, we were pleased to find that by the addition of a base (DABCO) a reaction took place, but rather than **3a’** the isoxazoline **3a** was observed in 22% yield. Further optimization by changing the solvent to chloroform and the base to quinuclidine improved the yield of **3a** to 52% (Table 1, entry 4), which could be further raised to 76% by switching to a Blue‐LED Schlenk set‐up that allowed the irradiation of the reaction solution from the top of the vessel (Table 1, entry 5; see Supporting Information for full details). Importantly, the reaction is dependent on oxygen (Table 1, entry 6; vide infra for mechanistic discussion) and both light and Cu(dap)_2_Cl proved to be essential (Table 1, entries 7 and 8). Increasing the reaction temperature from room temperature to 60 °C and extending the reaction time to 40 h, **3a** can also be obtained in the dark and in the absence of the copper(II) catalyst (74% yield) in line with the results reported by Cecchi and coworkers.^[^ ^37^ ^]^ However, this parallelly does not hold for other classes of alkenes. While exploring the scope of our protocol (Scheme 1), we also included the Machetti protocols ^[^ ^37^, ^39^ ^]^ for selected substrates, and especially 1,1‐disubstituted and cyclic alkenes did not give the cycloadducts under their conditions. Other established photocatalysts such as 4‐CzIPN (1,2,3,5‐tetrakis(carbazol‐9‐yl)‐4,6‐dicyanobenzene) or Ir[dF(CF_3_ppy]_2_(dtbbpy))PF_6_ ([Ir‐F], [4,4′‐bis(tert‐butyl)‐2,2′‐bipyridine]bis[3,5‐difluoro‐2‐[5‐(trifluoromethyl)‐2‐pyridinyl]phenyl]iridium(III) hexafluorophosphate failed in this transformation (Table 1, entries 9 and 10) (for full details on the optimization see the Supporting Information).

Armed with the optimized conditions, we explored the scope of alkenes by initially keeping ethyl nitroacetate as coupling partner (Scheme 1). Vinyl arenes of diverse electronic nature and different substitution patterns afforded **3a‐h** (54–82%). Comparable yields of isoxazolines obtained from highly electron‐deficient 1,2,3,4,5‐pentafluorostyrene (**3j**, 74%) and electron‐rich 2,4,6‐trimethyl styrene (**3k**, 72%) highlight the versatility of this anellation reaction toward the electronic nature of the alkene. A limitation was found for *p*‐methoxystyrene, which gave product **3i** in only 39% yield. Unactivated cyclic and acyclic alkenes were also amenable substrates and afforded the desired product (**3n‐s)**. Successful reactions with long‐chain terminal alkenes in the presence of reactive epoxy and hydroxy functional groups showcase the protocol's functional group tolerance (**3t‐y**). Additionally, electron‐poor olefins like α,β‐unsaturated carbonyl compounds and acrylonitrile also displayed notable reactivity (**3z, 3aa, 3ab**). The corresponding isoxazolines were also obtained from R‐(+)‐limonene (**3ac**, 42%), ketoprofen (**3ad**, 70%), estrone (**3ae**, 78%), and L‐menthol (**3af**, 84%). The reaction conditions also effectively accessed isoxazoles from aryl alkynes, yielding products **6a‐c**. Additionally, the protocol was applied to unactivated alkynes, resulting in the formation of **6d‐g**. The developed conditions also effectively utilized phenyl nitromethane (PNM) to yield the corresponding isoxazolines (**4a‐d**). Notably, alkenes such as **1g**, **1l**, **1p**, and **1q** failed to afford the corresponding isoxazolines under the Cu‐catalyzed conditions reported by Cecchi and coworkers.^[^ ^39^ ^]^ Nevertheless, limitations of our protocol were noted in the cases of 4‐vinyl pyridine, presumably interfering through coordination to copper, and trans‐1,2‐disubstituted olefins, which is attributed to steric effects.

### Mechanistic Studies

**Figure 3:**
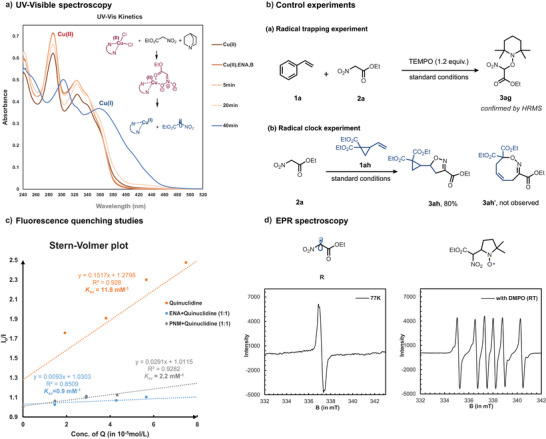
Summary of mechanistic studies: a) UV‐spectroscopy, b) control experiments, c) fluorescence quenching studies, d) EPR spectroscopy.

**Scheme 2:**
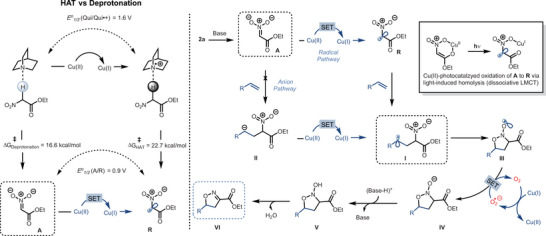
Plausible reaction mechanism. The HAT versus deprotonation barriers is computed in CPCM (CHCl_3_) M06‐2x/6–31++G (d, p)//M06‐2x/6–31G(d), while E°_1/2_ values are computed in CPCM (CHCl_3_) M062x/6–31 + G(d, p) level of theory (see Supporting Information for full details).

**Figure 4:**
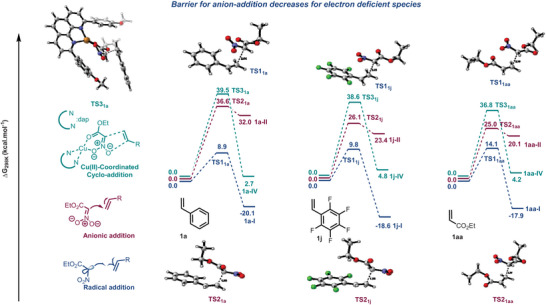
Reaction barrier for the addition of ENA to alkenes. All energies are computed in CPCM (CHCl_3_) M062x/6–31++G(d, p)//M062x/6–31G(d, p) level of theory.

To understand the mechanism of the reaction, a series of control experiments, spectroscopic studies, and computational analysis were carried out. The use of radical scavengers, such as TEMPO, drastically reduced the yield, and a corresponding adduct **3ag** was confirmed by HRMS, indicating the presence of a radical intermediate **R** in the reaction pathway (Figure 3b). In agreement, a strong EPR signal (*g* = 2.002) at 77 K was observed, thus confirming the presence of an organic radical (Figure 3d). A carbon‐centered radical (confirmed by the hyperfine splitting constants a(N) = 1.5 mT, a(H) = 2.3 mT) could also be trapped with DMPO at room temperature. Nevertheless, a radical clock experiment with alkene **1ah** yielded isoxazoline **3ah** exclusively, with no detection of ring‐opened product **3ah’**, suggesting the rapid cyclization of intermediate **I** to **III** (Scheme 2). The cyclization to form the isoxazoline **3ah** is found to be conformationally preferred and enthalpically exergonic compared to the rearrangement pathway that would lead to **3ah'** (see the Supporting Information for full reaction profile). Stern–Volmer experiments (Figure 3c) revealed that quinuclidine alone has the potential to quench the Cu photocatalyst (*K*_sv _= 11.8 mM^−1^), but when a 1:1 mixture of ENA and quinuclidine was employed, the quenching was suppressed (*K*_sv_ = 0.9 mM^−1^). Similarly, a 1:1 mixture of PNM and quinuclidine also demonstrated a suppressed quenching of the excited photocatalyst (*K*_sv_ = 2.2 mM^−1^) (for full plots refer Supporting Information section 8.3). Thus, in the presence of ENA, quinuclidine acts as a base rather than getting oxidized, suggesting that the reaction is initiated by the oxidation of the nitronate anion **A**. DFT studies also corroborate this observation (see the Supporting Information for full details). The formation of ENA radical **R** was studied using UV–visible absorption spectroscopy on a mixture of photocatalyst Cu(dap)Cl_2_, quinuclidine, and ENA (Figure 3a). An enhancement of the characteristic peaks of the absorption spectra of Cu(dap)Cl_2_ in the presence of base and ENA is initially observed, suggesting that the nitronate **A** coordinates to Cu(II). After 20 min of irradiation, the signals corresponding to Cu(II) noticeably dropped in intensity with a slight blueshift, and after 40 min, the characteristic shape changed completely, indicating the formation of Cu(I) via LMCT homolysis of the Cu(II)‐coordinated nitronate complex under reaction conditions to generate radical **R**. A Giese‐type addition of **R** to the terminal position of the alkene then occurs, yielding a more stabilized carbon‐centered radical intermediate **I**. This intermediate undergoes an intramolecular cyclization to form the oxygen‐centered radical species **III**, which is reduced by O_2_^•−^ to furnish the N‐hydroxy isoxazoline intermediate **V**. The final dehydration leads to the desired product **VI** (Scheme 2). Since both nitronate **A** and nitro radical **R** are possible intermediates *en* route to the [3 + 2]‐cycloadducts, with the help of DFT, we evaluated three possible pathways for their interaction with the alkenes, i.e., the addition of radical **R** (TS1), the addition of noncoordinated nitronate anion **A** (TS2), and concerted cycloaddition of **A** coordinated by Cu(II) (TS3) (Figure 4). The dap ligand was chosen to align best with the experimental conditions. Three alkenes **1a**, **1j,** and **1aa** were chosen as models for the study to cover the broad substrate scope.

For each substrate, the radical addition pathway is strongly favored over the anionic pathway, even when the alkene is electron‐deficient, as in **1aa**. The barrier for the addition of Cu(II)‐coordinated **A** is very high because the nucleophilicity of the anion is reduced due to coordination with Cu(II). The barriers for **A**‐attack versus **R**‐attack are computed for a few additional substrates, including the very electron‐deficient acrylonitrile **1ab** (see the Supporting Information). From these calculations, we conclude that the preference of a radical pathway over an anionic addition or a concerted‐cyclic pathway reflects the versatility of the process developed.

## Conclusion

**Scheme 3:**
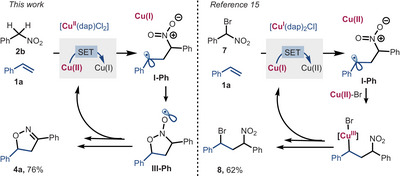
Divergent reaction pathways of nitroalkyl radicals generated by oxidation of nitroalkanes or reduction of bromonitroalkanes.

In summary, we have synthesized isoxazolines and isoxazoles—valuable classes of compounds relevant to agricultural and medicinal chemistry—via a radical pathway, utilizing the copper(II) photocatalyzed oxidation of nitronates. The so‐generated nitroalkyl radicals, along with the formation of Cu(I), undergo facile addition to a broad range of alkenes and alkynes at room temperature, generating intermediates of type **I‐Ph** (Scheme 3; *cf* Scheme 1) that subsequently cyclize to **III‐Ph** en route to the heterocyclic products. Strikingly, the same intermediates, e.g., **I‐Ph** can be generated by a reductive photocatalytic pathway, along with the formation of Cu(II), which, being a persistent radical, efficiently traps **I‐Ph** to deliver ATRA products such as **8**. These examples point to the possibility to modulate radical reactions by different oxidation states of metals, which will be further investigated by us.

## Conflict of Interests

The authors declare no conflict of interest.

## Supporting information



Supporting Information

## Data Availability

The data that support the findings of this study are available in the supplementary material of this article. The authors have cited additional references within the Supporting Information.^[^ ^9^, ^37^, ^39^, ^41^, ^42^, ^43^, ^44^, ^45^, ^46^, ^47^, ^48^, ^49^, ^50^, ^51^, ^52^, ^53^, ^54^, ^55^, ^56^ ^]^ The primary research data underlying this study are openly available in Radar4Chem at doi: 10.22000/r6fh8r7g1gdadj4q.
