# Supplementary material for: Copper(II)‐Photocatalyzed Radical Anellation of Nitroalkanes with Alkenes or Alkynes for the Synthesis of Isoxazolines and Isoxazoles
Source: Angew Chem Int Ed Engl. 2025 Aug 1;64(37):e202509658. doi: 10.1002/anie.202509658 (PMC12416462; doi:10.1002/anie.202509658)
Supplement: Supplementary file 1 — Supporting Information [file ANIE-64-e202509658-s001.pdf]

Supporting Information

Copper (II)-Photocatalyzed Radical Anellation of Nitroalkanes with Alkenes or Alkynes for the Synthesis of Isoxazolines and Isoxazoles

S. Sardana,[1] A. Pattanaik,[1] J. Rehbein,[1] O. Reiser*[1]*

[1]Institut für Organische Chemie, Universität Regensburg, Universitätsstr. 31, 93053 Regensburg, Germany

Correspondence to: oliver.reiser@chemie.uni-regensburg.de;

julia.rehbein@chemie.uni-regensburg.de

Table of Contents

1. General Information 3
2. Photochemical Setups 5
2.1 Photochemical LED Setup 5
2.2 Photochemical Schlenk Setup 5
3. Structures of copper photocatalyst 6
4. Starting materials used in this study 7
5. Detailed optimization of reaction conditions and control experiments 8
5.1 Robustness of the process using photochemical compared to thermal conditions. 11
6. Synthesis of styrenes derived from bioactive molecules 12
7. General procedure for the photochemical synthesis of isoxazolines and isoxazoles 15
7.1 Reactions of alkenes with ethyl nitroacetate 15
7.2 Reactions of alkynes with ethyl nitroacetate 15
7.3 Reactions of styrenes with phenyl nitromethane 16
7.4 Unsuccessful substrates 16
8. Mechanistic Studies 18
8.1 TEMPO-trapping experiment 18
8.2 Radical clock experiment 19
8.3 Fluorescence quenching studies 21
8.4 UV-Visible and spectroelectrochemistry studies 23
8.5 Electron Paramagnetic Resonance (EPR) analysis 27
8.6 Density Functional Theory (DFT) studies 29
9. Analytical Data of Products 108
10. References 125
11. Appendix 126
11.1 NMR-Spectra of Products 126
11.2 X- Ray Crystal structure of **3j** 175

1. General Information

Commercially available chemicals of high quality were purchased and used without further purification. Consequently, weight was calculated based on the purity stated on the container. All reactions were conducted in oven-dried glassware under atmospheric conditions unless otherwise noted. Reactions involving moisture or oxygen-sensitive reagents were performed in flame-dried glassware under a pre-dried nitrogen atmosphere. All photochemical reactions were carried out at room temperature. Reactions were monitored through thin layer chromatography (TLC). Anhydrous solvents were prepared using established laboratory procedures. DCM, EtOAc, n-pentane, and hexanes (40 - 60 °C) for chromatography were distilled before use. Reported yields refer to isolated compounds unless otherwise noted.

Chromatography

Thin layer chromatography (TLC) was performed with TLC precoated aluminum sheets (Merck) Silica gel 60 F254, 0.2 mm layer thickness, and visualized by a dual short (λ = 254 nm) / long (λ = 366 nm) wavelength UV lamp or stained with potassium permanganate solution. Column chromatography was performed with silica gel (Merck, 0.063-0.200 mm particle size) and flash silica gel (Merck, 0.040-0.063 mm particle size).

NMR-Spectroscopy

^{1}H NMR spectra were recorded on Bruker Avance 300 (300 MHz), Bruker Avance 400 (400 MHz), or Bruker Avance III 400 "Nanobay" (400 MHz). Chemical shifts for ^{1}H NMR were reported as δ, parts per million (ppm), relative to the residual proton signal of $CHCl_3$ at 7.26 ppm. Spectra were evaluated in the first order and coupling constants *J* are reported in Hertz (Hz). Splitting patterns for the spin multiplicity of the signals in the spectra are given as follows: s = singlet, bs = broad singlet, d = doublet, t = triplet, q = quartet, q = quintet, sex = sextet, hept = heptet, m = multiplet, and combinations thereof. Chemical shifts for ^{1}H NMR were reported as δ, parts per million (ppm), relative to the residual proton signal of $CDCl_3$ at 7.26 ppm.

^{13}C NMR spectra were recorded on Bruker Avance 300 (75 MHz), Bruker Avance 400 (101 MHz) or Bruker Avance III 400 "Nanobay" (101 MHz) Spectrometer. Chemical shifts for ^{13}C NMR were reported as δ, parts per million (ppm), relative to the center line signal of the $CDCl_3$ triplet at 77.0 ppm.

^{19}F NMR spectra were recorded on Bruker Avance 300 (282 MHz), Bruker Avance 400 (376 MHz) or Bruker Avance III 400 "Nanobay" (376 MHz) Spectrometer.

Mass Spectrometry

Mass spectra were recorded by the Central Analytic Department of the University of Regensburg using Jeol AccuTOF GCX and Agilent Q-TOF 6540 UHD Spectrometer. High-

resolution mass spectra were measured using atmospheric pressure chemical ionization (APCI), electron ionization (EI) or electrospray ionization (ESI) with a quadrupole time-of-flight (Q-TOF) detector.

X-Ray Crystallography

X-ray crystallographic analysis was conducted by the Central Analytic Department of the University of Regensburg utilizing an Agilent Technologies SuperNova, an Agilent Technologies Gemini R Ultra, an Agilent GV 50, or a Rigaku GV 50 diffractometer. Suitable crystals were mounted on a Lindemann tube oil and maintained at a consistent temperature of T = 293 K during data collection. The structures were solved using the SheIXT (Scheldrick 2015) structure solution program with the Intrinsic Phasing solution method and Olex2 as the graphical interface. The model was refined with SheIXL employing least squares minimization.

EPR Spectroscopy

The X-Band EPR measurements were carried out with MiniScope MS400 device with a frequency of 9,5 GHz and rectangular resonator TE102 of the company Magnettech GmbH.

Spectroelectrochemistry

Spectroelectrochemistry (SEC) was performed using a Cary 8454 Online UV-Visible spectrophotometer and a three-electrode potentiostat/galvanostat PGSTAT302N from Metrohm Autolab.

2. Photochemical Setups

2.1 Photochemical LED Setup

Unless otherwise mentioned, photoreactions under air at regular scale were performed using blue LEDs from Osram OSLON® SSL 80 deep blue (3 W, 700 mA, dominant wavelength λ_{dom} = 455 nm, spectral bandwidth at 50% I_{max} = 20 nm, optical power at 25 °C 1.4 W). Reactions were illuminated from the flat bottom side of the vial (Figure **S1**). The temperature of the reaction mixtures was controlled by a water-cooling circuit consisting of an aluminum cooling block connected to a thermostat.

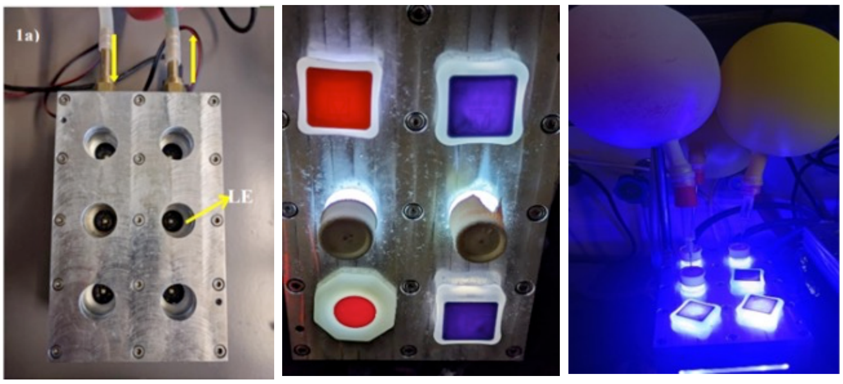

Figure S1: Photochemical setup.

2.2 Photochemical Schlenk Setup

For the reaction conducted under nitrogen, blue light irradiation was achieved using an Osram OSLON® SSL 80 deep blue (3 W, 700 mA, dominant wavelength λ_{dom} = 455 nm, spectral bandwidth at 50% I_{max} = 20 nm, radiant power at 25 °C and 700 mA ~ 900 mW). All photochemical reactions were carried out in flame-dried Schlenk tubes (10.0 mL size; Figure S2, D) equipped with a magnetic stirring bar (Figure S2, E), using monochromatic light-emitting diodes (LEDs) (Figure S2, A) as the irradiation source. The LEDs are positioned on a glass rod (8 mm diameter; borosilicate glass; Schott Borofloat® 33; Figure S2, B), functioning as fiber optics, which is directly immersed in the reaction mixture.

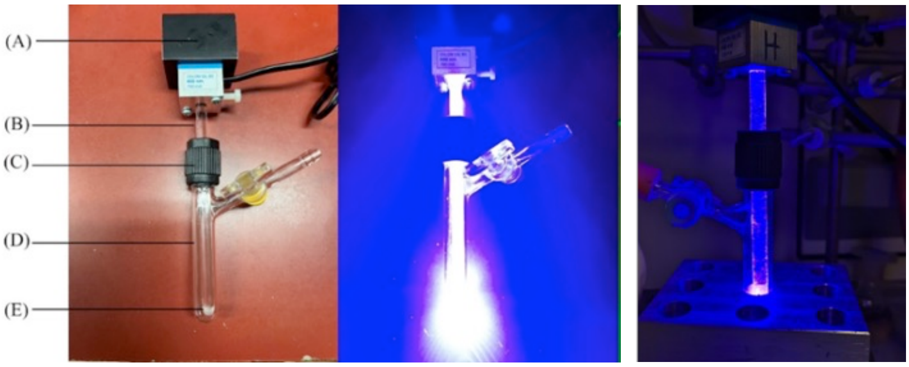

Figure S2: Schlenk LED setup- Irradiation setup (A) LED; (B) glass rod; (C) Teflon adapter; (D) Schlenk tube (10.0 mL size); (E) Teflon-coated stirring bar

3. Structures of copper photocatalyst

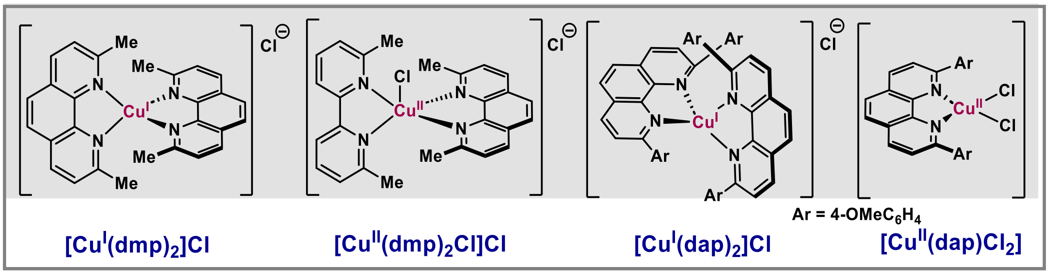

Figure S3: Structures of copper photocatalysts.

These catalysts were prepared according to a known literature procedure.[18]

4. Starting materials used in this study

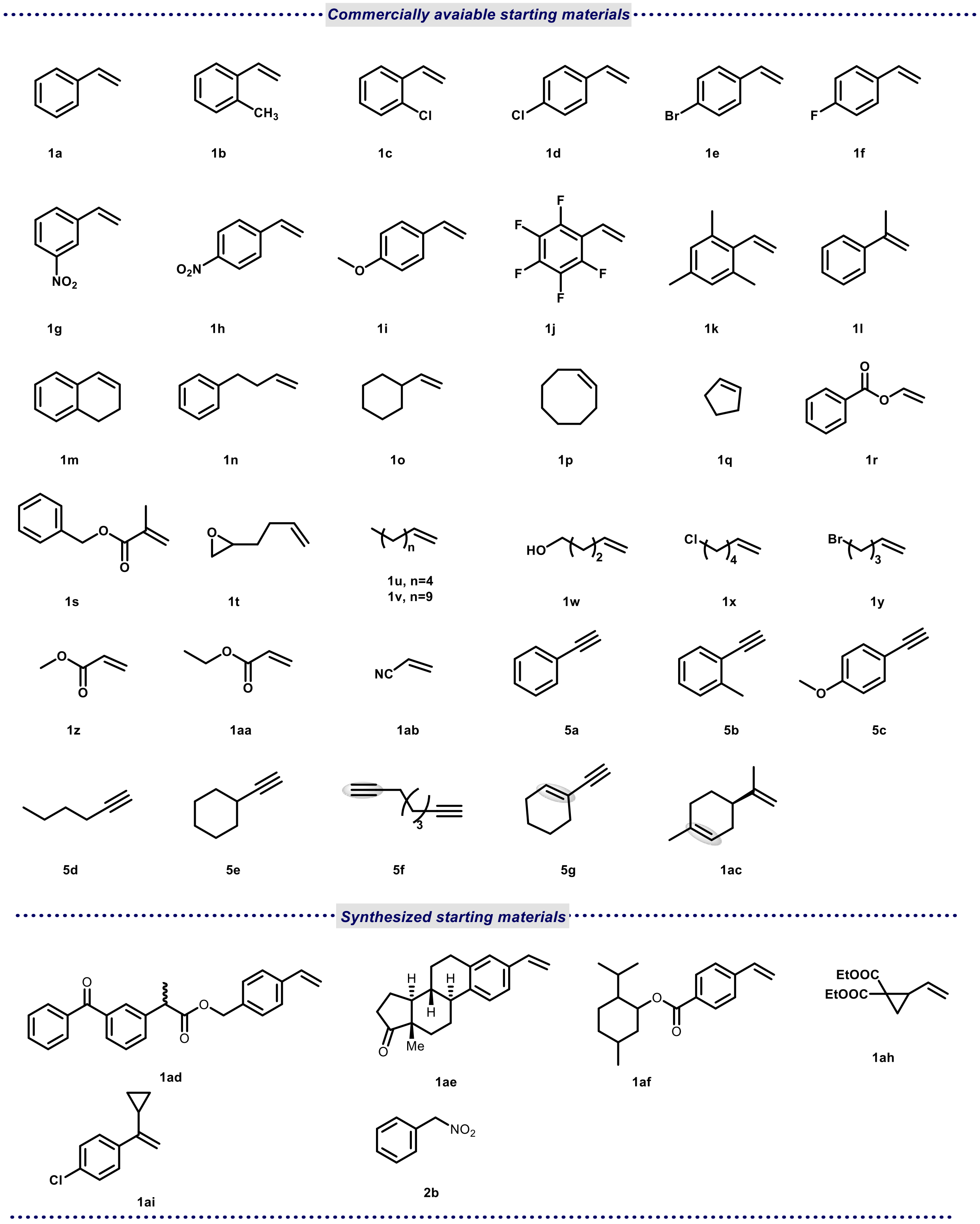

Figure S4: Structures of commercially available and synthesized starting materials.

5. Detailed optimization of reaction conditions and control experiments

Table S1: Screening of catalysts

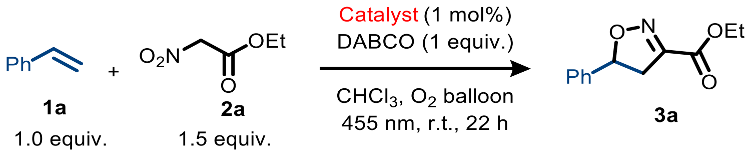

Entry[a]	Catalyst	Yield[b]
1	$Cu^{I}(dap)_2Cl$	12%
2	$Cu^{II}(dap)Cl_2$	22%
3	$Cu^{I}(dmp)_2Cl$	17%
4	$Cu^{II}(dmp)_2Cl_2$	16%
5	4CzIPN	n.d.
6	$(Ir[dF(CF_3)ppy]_2(dtbbpy))PF_6$	n.d.

Reaction conditions: [a] **1a** styrene (0.20 mmol, 1.0 equiv.), **2a** ethyl nitroacetate (0.30 mmol, 1.5 equiv.), DABCO (0.20 mmol, 1.0 equiv.), catalyst (20 µmol, 1.0 mol%) in $CHCl_3$ (1.5 mL, 0.13 M), irradiated at indicated λ_{max} under an O_2 balloon for 22 h at room temperature [b] ^{1}H-NMR yield using 1,1,2,2-tetrachloroethane as an internal standard. n.d. = not detected.

$Cu(dap)Cl_2$ gave the best yield (36% NMR yield) and was hence selected for further screenings.

Table S2: Screening of reactants ratio

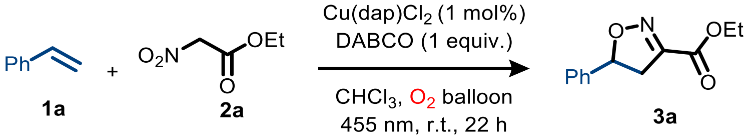

Entry[a]	1a:2a	Yield[b]
1	1:1.5	36%
2	1.5:1	32%
3	1:2	12%
4	2:1	14%
5	1:5	16%
6	5:1	24%

Reaction conditions: [a] **1a** styrene (x equiv.), **2a** ethyl nitroacetate (y equiv.), 0.20 mmol scale with respect to the limiting reagent, DABCO (0.20 mmol, 1.0 equiv.), $Cu(dap)Cl_2$ (20 µmol, 1.0 mol%) in $CHCl_3$ (1.5 mL, 0.13 M), irradiated at 455 nm under an oxygen atmosphere (O_2 balloon) for 22 h at room temperature [b] ^{1}H-NMR yield using 1,1,2,2-tetrachloroethane as an internal standard. n.d. = not detected.

Table S3: Screening of solvent

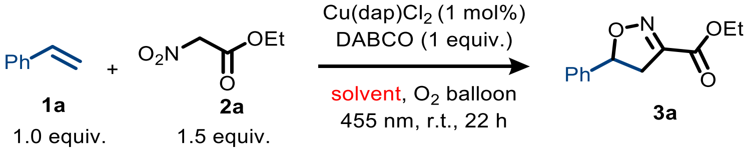

Entry[a]	Solvent	Yield[b]
1	$CHCl_3$	36%
2	DCE	26%
3	Toluene	22%
4	Dioxane	16%
5	Cyclohexane	10%
6	Diethyl ether	traces
7	DMSO	traces
8	DMF	n.d.
9	MeCN	22%
10	Acetone	22%
11	MeOH	30%
12	H_2O	16%

Reaction conditions: [a] **1a** styrene (0.20 mmol, 1.0 equiv.), **2a** ethyl nitroacetate (0.30 mmol, 1.5 equiv.), DABCO (0.20 mmol, 1.0 equiv.), Cu(dap)Cl_2 (20 µmol, 1.0 mol%) in solvent (1.5 mL, 0.13 M), irradiated at indicated λ_{max} under an oxygen atmosphere (O_2 balloon) for 22 h at room temperature [b] ^{1}H-NMR yield using 1,1,2,2-tetrachloroethane as an internal standard. n.d. = not detected.

Table S4: Screening of base

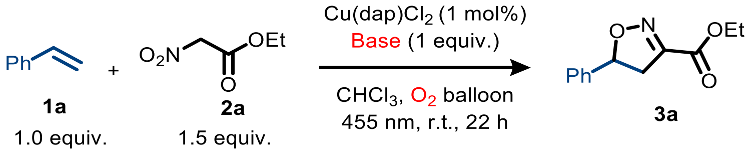

Entry[a]	Base	Yield[b]
1	K_2CO_3	crm
2	K_3PO_4	crm
3	KO^tBu	crm
4	DBU	20%
5	DABCO	36%
6	quinuclidinol	30%
7	triethylamine	14%
8	N-Me piperidine	48%
9	2,4,6-collidine	n.d.
10	quinuclidine	52%

Reaction conditions: [a] **1a** styrene (0.20 mmol, 1.0 equiv.), **2a** ethyl nitroacetate (0.30 mmol, 1.5 equiv.), base (0.20 mmol, 1.0 equiv.), Cu(dap)Cl_2 (20 µmol, 1.0 mol%) in $CHCl_3$ (1.5 mL, 0.13 M), irradiated at indicated λ_{max} an oxygen atmosphere (O_2 balloon) for 22 h at room temperature [b] ^{1}H-NMR yield using 1,1,2,2-tetrachloroethane as an internal standard. n.d. = not detected. crm-complex reaction mixture.

Table S5: Variation from reaction conditions

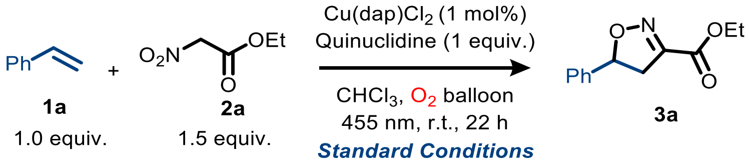

Entry*a*	Variation	Yield*b*
1	2 mol% catalyst	38%
2	10 mol% catalyst	43%
3	Blue LED-Schlenk setup	76%
4	10 h reaction time	20%
5	48 h reaction time	50%
6	0.5 equiv. of base	38%
7	2 equiv. of base	52%
8	Reaction temperature 0 °C	36%
7	Reaction temperature −20 °C	16%

Standard conditions: [a] **1a** styrene (0.20 mmol, 1.0 equiv.), **2a** ethyl nitroacetate (0.30 mmol, 1.5 equiv.), quinuclidine (0.20 mmol, 1.0 equiv.), Cu(dap)Cl_2 (20 µmol, 1.0 mol%) in $CHCl_3$ (1.5 mL, 0.13 M), irradiated at indicated λ_{max} under an O_2 balloon for 22 h at room temperature [b] ^{1}H-NMR yield using 1,1,2,2-tetrachloroethane as an internal standard. n.d. = not detected.

Table S6: Control experiments

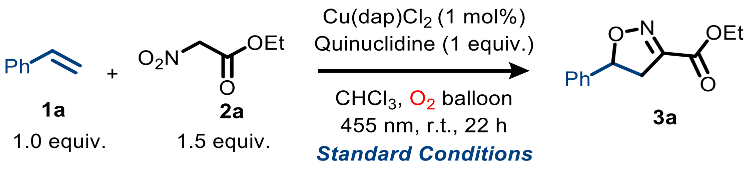

Entry[a]	Variations	Yield[b]
1	under N_2	n.d.
2	no base	traces
3	no photocatalyst	n.d.
4	no light	n.d.

Reaction conditions: [a] **1a** styrene (0.20 mmol, 1.0 equiv.), **2a** ethyl nitroacetate (0.30 mmol, 1.5 equiv.), quinuclidine (0.20 mmol, 1.0 equiv.), Cu(dap)Cl_2 (20 µmol, 1.0 mol%) in $CHCl_3$ (1.5 mL, 0.13 M), irradiated at indicated λ_{max} under an O_2 balloon for 22 h at room temperature [b] ^{1}H-NMR yield using 1,1,2,2-tetrachloroethane as an internal standard. n.d. = not detected.

As shown in Table S6, the presence of an oxygen atmosphere, base, photocatalyst and light are required for the transformation.

5.1 Robustness of the process using photochemical compared to thermal conditions.

To evaluate the robustness of the developed photocatalytic method for synthesizing isoxazolines, selected olefins were subjected to the thermal conditions previously reported by Machetti for comparison.[20] The results clearly demonstrate the superiority of the photocatalytic protocol, which exhibits excellent performance across a wide range of substrates, including unactivated aliphatic and cyclic alkenes, as well as electron-deficient styrenes.

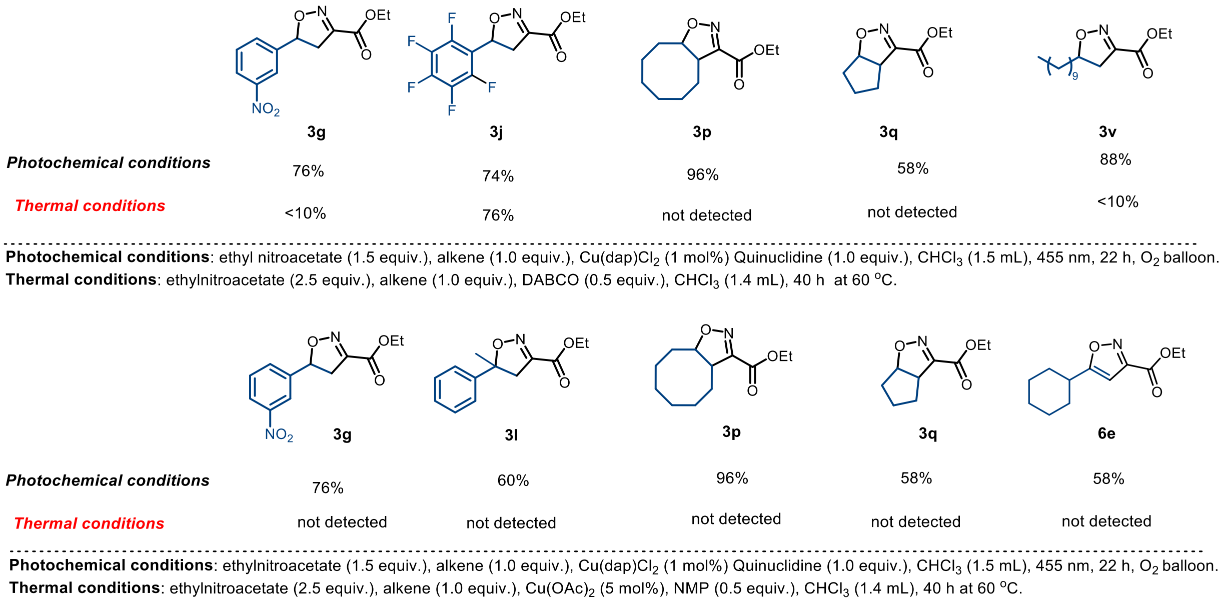

Figure S5: Comparison of product yield under thermal vs photochemical conditions.

6. Synthesis of styrenes derived from bioactive molecules

4-Vinylbenzyl 2-(3-benzoylphenyl)propanoate (1ad)

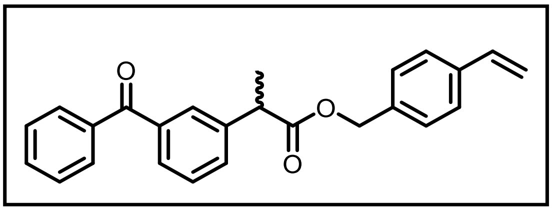

In a 250 mL round-bottomed flask equipped with Teflon-coated magnetic stirring bar was charged with Ketoprofen (1.27 g, 5 mmol, 1 equiv.), KI (1.24 g, 7.5 mmol, 1.5 equiv.) and K_2CO_3 (1.03 g, 7.5 mmol, 1.5 equiv.). DMF (0.2 M) was added, and the solids were stirred for 5 minutes before adding 4-vinylbenzyl chloride (820 µL, 5.5 mmol, 1.1 equiv.). The reaction mixture was stirred overnight (*ca.* 18 h) before checking the completion by TLC analysis. Afterwards, 50 mL of water was added along with 50 mL EtOAc. The organic layer was separated and washed with water (3 × 50 mL) followed by drying over anhydrous Na_2SO_4 and concentrated *in vacuo*. The resulting crude residue was purified on silica (n-hexane/EtOAc, 70:30) to give **1ad** (2.63g, 91%) as a colorless liquid. Spectral data agree with those reported in literature.[19a]

(8R,9S,13S,14S)-13-Methyl-3-vinyl-6,7,8,9,11,12,13,14,15,16-decahydro-17H-cyclopenta[a]phenanthren-17-one (1ae)

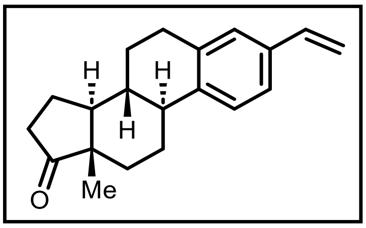

Based on a literature procedure, a flame-dried 3-neck round bottom flask was charged with (8R,9S,13S,14S)-13-methyl-17-oxo-7,8,9,11,12,13,14,15,16,17-decahydro-6H-cyclopenta[a]phenanthren-3-yl trifluoromethanesulfonate (2.01 g, 5.0 mmol, 1.0 equiv.), potassium vinyltrifluoroborate (739.9 mg, 5.0 mmol, 1.0 equiv.), Cs_2CO_3 (4.89 g, 15.0 mmol, 3.0 equiv.), $PdCl_2$ (17.7 mg, 0.1 mmol, 2 mol%), PPh_3 (78.7 mg, 0.3 mmol, 60 mol%), and THF/ water (9:1, 10 mL) were stirred under refluxing conditions for 24 h. The reaction was monitored by TLC analysis. Afterwards, the reaction mixture was diluted with H_2O (50 mL) and extracted three times with DCM (3 × 25 mL). The combined organic layers were dried over anhydrous $MgSO_4$, filtered, and concentrated *in vacuo*. The residue was purified on silica (n-hexane/EtOAc, 10:1 to 5:1) to yield **1ae** (757.1 mg, 54%) as a white solid. Spectral data agree with those reported in literature.[19a]

(1R,2S,5R)-2-Isopropyl-5-methylcyclohexyl 4-vinylbenzoate (1af)

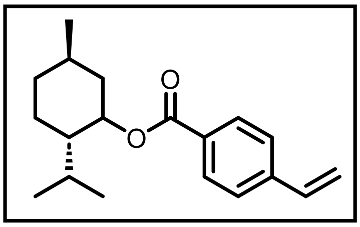

In a 50 mL round-bottomed flask equipped with Teflon-coated magnetic stirring bar was added L-Menthol (781 mg, 5 mmol, 1 equiv.), 4-vinylbenzoic acid (740 mg, 5 mmol, 1 equiv.) EDC·HCl (1.05 g, 5.5 mmol, 1.1 equiv.) and DMAP (61 mg, 0.5 mmol, 0.1 equiv., 10 mol%). DCM (1 M) was added, and the reaction was stirred overnight (*ca.* 18 h) before checking the completion by TLC analysis. The reaction mixture was transferred to a separating funnel and washed with 1 M HCl (20 mL). The organic layer was collected and washed with saturated aq. $NaHCO_3$ solution (1 × 20 mL) and brine (1 × 20 mL) followed by drying over anhydrous Na_2SO_4 and concentrated *in vacuo*. The crude product was purified on silica (n-hexane/EtOAc, 10:1) to yield **1af** (580 mg, 32%) as a colorless oil. Spectral data agrees with those reported in literature.[19a]

Diethyl 2-vinylcyclopropane-1,1-dicarboxylate (1ah)

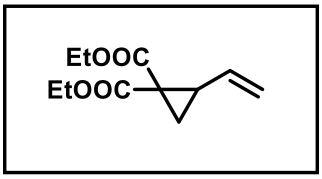

Based on a literature procedure, a flame-dried 3-neck round bottom flask was filled with nitrogen, a solution of 1,4-dibromo-2-butene (641 mg, 3 mmol, 1 equiv.) in THF (2.5 mL) was added followed by addition of diethyl malonate (480 mg, 3 mmol, 1 equiv.). The mixture was cooled down to 0 °C. A solution of NaH (60% in mineral oil) (240 mg, 6 mmol, 2 equiv.) in THF (2 mL) was added to resulting solution. The resulting mixture was stirred overnight at 78 °C. The reaction mixture was filtered and diluted with diethyl ether. The combined organic phases were dried over $MgSO_4$ and concentrated in *vacuo*. The residue was distilled under reduced pressure to afford **1ah** (407 mg, 64%) as a colorless oil. Spectral data agree with those reported in literature.[19b]

1-Chloro-4-(1-cyclopropylvinyl)benzene (1ai)

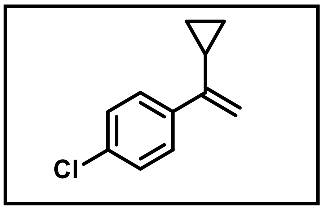

Based on a literature procedure, sodium bis(trimethylsilyl)amide (NaHDMS, 2.0 M solution in THF, 1.2 equiv.) was added dropwise to a solution of methyltriphenylphosphonium bromide (7.2 mmol, 1.2 equiv.) in anhydrous THF (10 mL) at 0 °C. After addition, the mixture was

stirred at 0 °C for 30 min. Then respective ketone (6 mmol, 1.0 equiv.) was added dropwise in THF (10 mL) at –78 °C. The reaction mixture is allowed to warm to room temperature overnight and quenched with ammonium chloride solution. The organic phase was separated, and the aqueous layer was extracted with EtOAc (3 × 20 mL). The combined organic phases were dried over $MgSO_4$ and concentrated in *vacuo*. The crude product was purified on silica (n-hexane/EtOAc, 10:1) to yield **1ai** (801 mg, 75%) as a yellow oil. Spectral data agree with those reported in literature.[19c]

Phenyl nitromethane (2b)

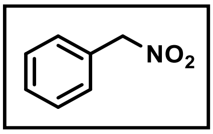

Based on a literature procedure, oxime (5 mmol, 1.0 equiv.) was dissolved in 25 ml of acetic acid, followed by the addition of sodium perborate tetrahydrate (30 mmol, 6.0 equiv.), and the reaction mixture was allowed to stir at 55 °C for 20 h. The reaction mixture was diluted with diethyl ether and water. The aqueous layer was then extracted again with ether. The organic layers were washed with $NaHCO_3$ solution, dried over $MgSO_4$, and concentrated in *vacuo*. The crude product was purified on silica (n-hexane/EtOAc, 10:1) to yield **2b** (506 mg, 74%) as a yellow oil. Spectral data agree with those reported in the literature.[19d]

7. General procedure for the photochemical synthesis of isoxazolines and isoxazoles

7.1 Reactions of alkenes with ethyl nitroacetate

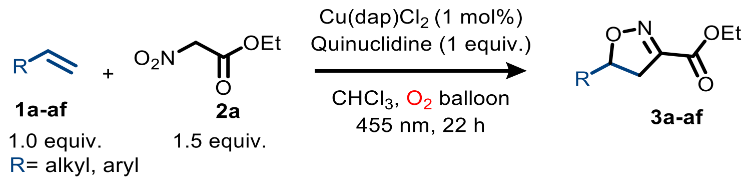

Setup shown in Figure S1: A glass reaction vial (5 mL), equipped with a Teflon-coated magnetic stirring bar, was charged with the corresponding alkene (0.2 mmol, 1.0 equiv.), [Cu(dap)Cl_2] (1.14 mg, 0.01 equiv., 1.0 mol%), and quinuclidine (25.4 mg, 0.2 mmol, 1.0 equiv.) followed by the addition of 1.5 mL $CHCl_3$. Ethyl nitroacetate (33.27 µL, 0.3 mmol, 1.5 equiv.) was added to the vial *via* a microsyringe. The vial was irradiated with a blue LED (λ_{max} = 455 nm, 6 W optical power) under an oxygen atmosphere (O_2 balloon), and the solution was stirred at room temperature (25 °C) maintained by continuous cold-water flow through the “LED Box” connected to a thermostat. Upon completion (22 h, monitored by TLC analysis), the reaction mixture was diluted with DCM (10 mL), transferred to a round-bottomed flask, concentrated *in vacuo*, and the crude residue was purified on silica (eluent: EtOAc/n-hexane) to afford target products.

Setup shown in Figure S2: An oven-dried Schlenk tube (10 mL) equipped with a Teflon-coated magnetic stirring bar was charged with the corresponding alkene (0.2 mmol, 1.0 equiv.), [Cu(dap)Cl_2] (1.14 mg, 0.01 equiv., 1.0 mol%) and quinuclidine (25.4 mg, 0.2 mmol, 1.0 equiv.) followed by the addition of 1.5 mL $CHCl_3$. Ethyl nitroacetate (33.27 µL, 0.3 mmol, 1.5 equiv.) was added to the vial *via* a microsyringe. A Teflon sealed inlet for a glass rod was placed inside the reaction tube, through which irradiation with λ_{max} = 455 nm took place from above under an oxygen atmosphere (O_2 balloon). The mixture was stirred in an aluminium block at room temperature (25 °C). After completion of the reaction (22 h, monitored by TLC analysis), the reaction mixture was diluted with DCM (10 mL), transferred to a round-bottomed flask, concentrated *in vacuo*, and the crude residue was purified on silica (eluent: EtOAc/n-hexane) to afford the products.

7.2 Reactions of alkynes with ethyl nitroacetate

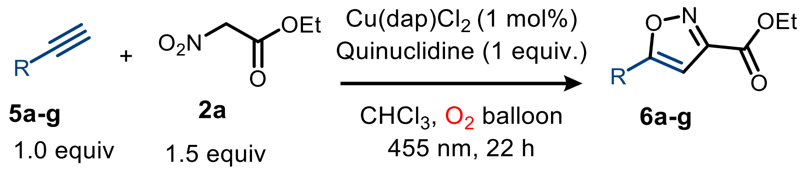

Setup shown in Figure S1: A glass reaction vial (5 mL), equipped with a Teflon-coated magnetic stirring bar, was charged with the corresponding alkyne (0.2 mmol, 1.0 equiv.),

[Cu(dap)Cl$_2$] (1.14 mg, 0.01 equiv., 1.0 mol%), and quinuclidine (25.4 mg, 0.2 mmol, 1.0 equiv.) followed by the addition of 1.5 mL $CHCl_3$. Ethyl nitroacetate (33.27 µL, 0.3 mmol, 1.5 equiv.) was added to the vial *via* a microsyringe. The vial was irradiated with a blue LED (λ_{max} = 455 nm, 6 W optical power) under an oxygen atmosphere (O_2 balloon), and the solution was stirred at room temperature (25 °C) maintained by continuous cold-water flow through the "LED Box" connected to a thermostat. Upon completion (22 h, monitored by TLC analysis), the reaction mixture was diluted with DCM (10 mL), transferred to a round-bottomed flask, concentrated *in vacuo*, and the crude residue was purified on silica (eluent: EtOAc/n-hexane) to afford respective isoxazoles.

7.3 Reactions of styrenes with phenyl nitromethane

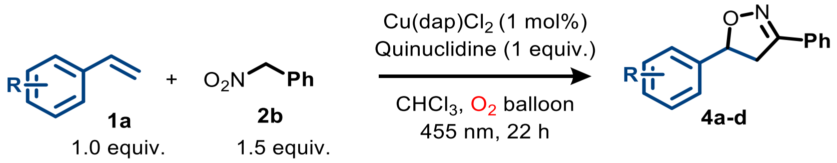

Setup shown in Figure S1: A glass reaction vial (5 mL), equipped with a Teflon-coated magnetic stirring bar, was charged with the corresponding styrene (0.2 mmol, 1.0 equiv.), [Cu(dap)Cl$_2$] (1.14 mg, 0.01 equiv., 1.0 mol%), and quinuclidine (25.4 mg, 0.2 mmol, 1.0 equiv.) followed by the addition of 1.5 mL $CHCl_3$. Phenyl nitromethane (41.1 mg, 0.3 mmol, 1.5 equiv.) was added to the vial *via* a microsyringe. The vial was irradiated with a blue LED (λ_{max} = 455 nm, 6 W optical power) under an oxygen atmosphere (O_2 balloon), and the solution was stirred at room temperature (25 °C) maintained by continuous cold-water flow through the "LED Box" connected to a thermostat. Upon completion (22 h, monitored by TLC analysis), the reaction mixture was diluted with DCM (10 mL), transferred to a round-bottomed flask, concentrated *in vacuo*, and the crude residue was purified on silica (eluent: EtOAc/n-hexane) to afford target products.

7.4 Unsuccessful substrates

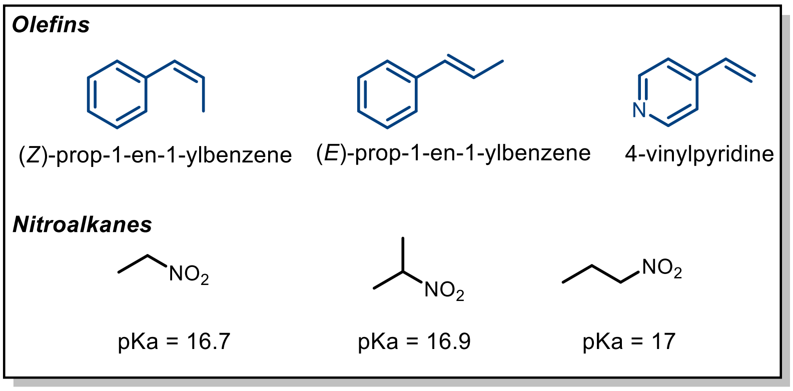

In our report, both cis and trans-1,2-β-methylstyrene did not yield the expected product under the standard conditions. We believe that the lack of reactivity may be attributed to steric hindrance arising from the free rotation of the methyl group, which could obstruct the approach of the radical species. This observation is consistent with our

results for conformationally constrained alkenes such as 1,2-dihydronaphthalene (**3m**, 42%), which readily underwent the reaction and provided the desired product in good yields.

In the case of nitroalkanes, the above-mentioned compounds did not yield the desired products under the standard conditions. According to the optimized conditions, quinuclidine was used as the base; however, it is not sufficiently basic to deprotonate simple alkyl nitro compounds. Effective deprotonation requires the hydrogen α to the nitro group to have a moderately low pK_a, which is typically achieved through the presence of an electron-withdrawing group, as in ethyl nitroacetate, or a phenyl group, as in phenyl nitromethane.

8. Mechanistic Studies

8.1 TEMPO-trapping experiment

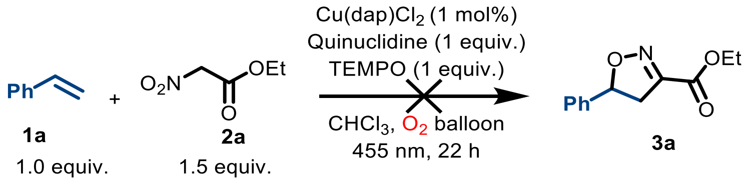

Scheme S1: Radical trapping experiment with TEMPO.

A glass reaction vial (5 mL), equipped with a Teflon-coated magnetic stirring bar, was charged with the styrene (22.9 µL, 0.2 mmol), [Cu(dap)Cl_2] (1.14 mg, 1 mol%), TEMPO (50 mg, 1.0 equiv.), quinuclidine (25.4 mg, 0.2 mmol, 1.0 equiv.) followed the addition of 1.5 mL $CHCl_3$. Ethyl nitroacetate (33.27 µL, 0.3 mmol, 1.5 equiv.) were added to the vial *via* a microsyringe. The vial was irradiated with a blue LED (λ_{max} = 455 nm, 6 W optical power) under an oxygen atmosphere (O_2 balloon), and the solution was stirred at room temperature (25 ℃) maintained by continuous cold-water flow through the "LED Box" connected to a thermostat. No formation of product **3a** was observed instead, the TEMPO-adduct **3ag** was identified and characterized by HRMS analysis (Figure S6).

HRMS (EI+): m/z $[M + H]^+$ calcd. for $C_{13}H_{25}N_2O_5$ 289.1763, found: 289.1757.

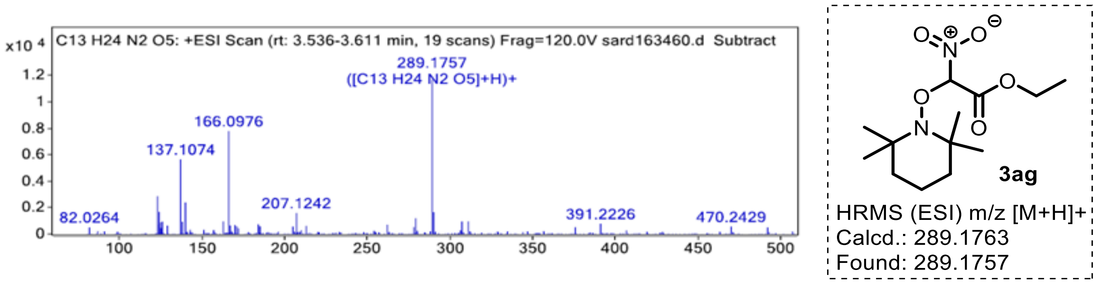

Figure S6. HRMS (ESI-TOF) reports of the adduct obtained.

8.2 Radical clock experiment

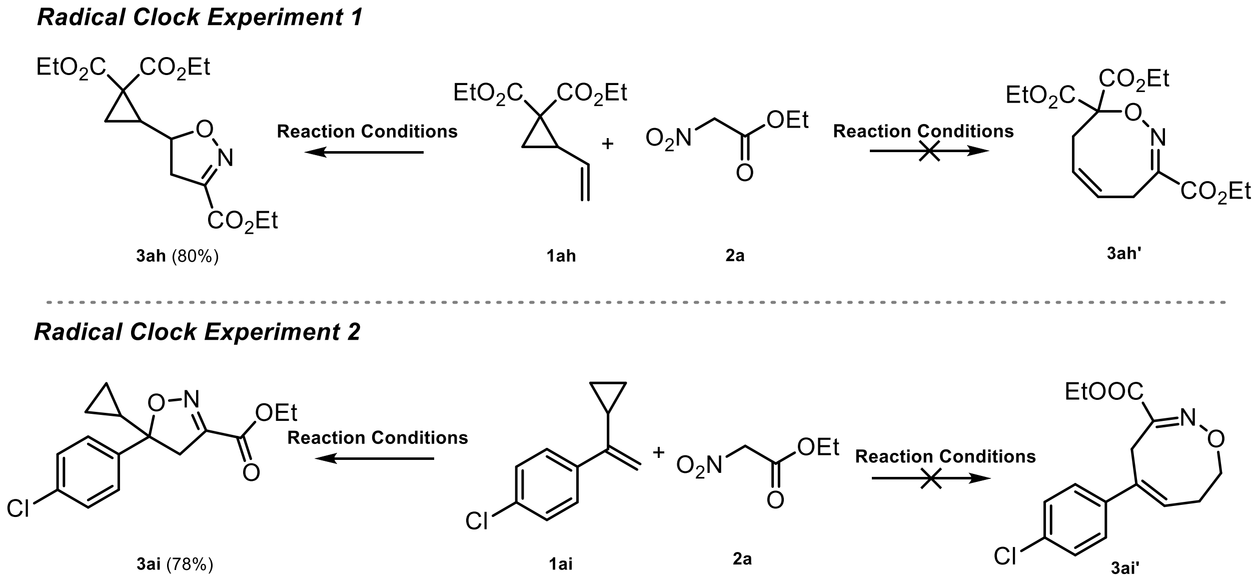

Scheme S2: Radical clock experiment

Experimental Procedure:

Radical clock experiment 1

A glass reaction vial (5 mL) equipped with a Teflon-coated magnetic stirring bar was charged with diethyl 2-vinylcyclopropane-1,1-dicarboxylate (**1ah**, 0.2 mmol, 1.0 equiv.), [Cu(dap)Cl_2] (1.14 mg, 0.01 equiv., 1.0 mol%) and quinuclidine (25.4 mg, 0.2 mmol, 1.0 equiv.) followed by the addition of 1.5 mL $CHCl_3$ (reaction setup Figure S1). Ethyl nitroacetate (33.27 µL, 0.3 mmol, 1.5 equiv.) was added to the reaction mixture *via* a microsyringe. The vial was irradiated with a blue LED (λ_{max} = 455 nm, 6 W optical power) under an oxygen atmosphere (O_2 balloon) at room temperature (25 °C), maintained by continuous cold-water flow through the "LED Box" connected to a thermostat. Upon completion (~ 22 h, monitored by TLC analysis the reaction mixture was diluted with DCM (10 mL), transferred to a round-bottomed flask, concentrated *in vacuo*, and the crude residue was purified on silica (eluent: EtOAc/n-hexane = 1:5) to afford the isoxazoline **3ah** as yellow liquid in 80% yield.

ethyl 5-methyl-5-phenyl-4,5-dihydroisoxazole-3-carboxylate (**3ah**)

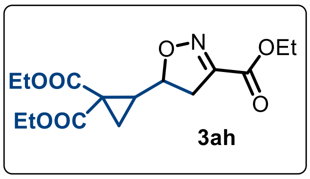

^{1}H NMR (400 MHz, $CDCl_3$) δ [ppm] 4.54 (ddd, J = 11.0, 8.3, 7.4 Hz, 1H), 4.35 (q, J = 7.2 Hz, 2H), 4.21 (dtd, J = 16.1, 7.2, 3.3 Hz, 4H), 3.35 (dd, J = 17.9, 11.0 Hz, 1H), 3.13 (dd, J = 17.9, 7.5 Hz, 1H), 2.05 (td, *J* = 8.7, 7.2 Hz, 1H), 1.63 – 1.52 (m, 2H), 1.37 (t, J = 7.1 Hz, 3H), 1.28 (dt, J = 14.3, 7.1 Hz, 6H).

^{13}**C NMR** (101 MHz, $CDCl_3$) δ [ppm] 169.25, 167.27, 160.45, 151.71, 82.78, 62.19, 62.02, 61.92, 39.37, 32.92, 30.33, 19.06, 14.12, 14.07, 14.01.

HRMS (ESI+) m/z: [M+H]+ calcd. for $C_{15}H_{22}NO_7$ 328.1396, found 328.1397.

Radical clock experiment 2

A glass reaction vial (5 mL) equipped with a Teflon-coated magnetic stirring bar was charged with 1-chloro-4-(1- cyclopropylvinyl)benzene (**1ai**, 0.2 mmol, 1.0 equiv.), [Cu(dap)Cl_2] (1.14 mg, 0.01 equiv., 1.0 mol%) and quinuclidine (25.4 mg, 0.2 mmol, 1.0 equiv.) followed by the addition of 1.5 mL $CHCl_3$ (reaction setup Figure S1). Ethyl nitroacetate (33.27 µL, 0.3 mmol, 1.5 equiv.) was added to the reaction mixture *via* a microsyringe. The vial was irradiated with a blue LED (λ_{max} = 455 nm, 6 W optical power) under an oxygen atmosphere (O_2 balloon) at room temperature (25 °C), maintained by continuous cold-water flow through the "LED Box" connected to a thermostat. Upon completion (~ 22 h, monitored by TLC analysis) the reaction mixture was diluted with DCM (10 mL), transferred to a round-bottomed flask, concentrated *in vacuo*, and the crude residue was purified on silica (eluent: EtOAc/n-hexane = 1:5) to afford the isoxazoline **3ai** as yellow liquid in 78% yield.

ethyl 5-(4-chlorophenyl)-5-cyclopropyl-4,5-dihydroisoxazole-3-carboxylate (**3ai**)

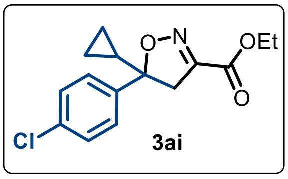

^{1}**H NMR** (400 MHz, $CDCl_3$) δ [ppm] 7.46 – 7.29 (m, 4H), 4.33 (q, J = 7.1 Hz, 2H), 3.43 – 3.25 (m, 2H), 1.43 – 1.32 (m, 4H), 0.62 (dd, J = 6.4, 3.9 Hz, 1H), 0.55 – 0.41 (m, 3H).

^{13}**C NMR** (101 MHz, $CDCl_3$) δ [ppm] 160.69, 151.44, 142.40, 133.87, 128.76, 126.81, 92.44, 62.29, 45.65, 20.62, 14.25, 2.30, 1.69.

HRMS (ESI+) m/z: [M]+H+ calcd. for $C_{15}H_{17}ClNO_3$ 294.0897, found 294.0894.

A detailed mechanistic rationale (DFT) for the formation of the cyclized products **3ah** instead of the ring-opening rearranged products **3ah'** is provided in **Section 8.6**.

8.3 Fluorescence quenching studies

Stern-Volmer quenching studies

Emission intensities were recorded using a spectrofluorometer with the excitation wavelength set at 495 nm. Samples were prepared by mixing a stock solution **A** of Cu(dap)Cl_2 (concentration: 0.02 M) with varying amounts of quinuclidine in $CHCl_3$ within a quartz fluorescence cuvette. The stock solution concentration of quinuclidine in $CHCl_3$ was 0.002 M. For each quenching experiment, 10 µL of the quinuclidine solution was added incrementally to the cuvette containing 10 µL of the Cu(dap)Cl_2 solution, and the total volume was adjusted to 1 mL by addition of $CHCl_3$. Emission intensities were recorded after each addition and plotted in the graph shown below.

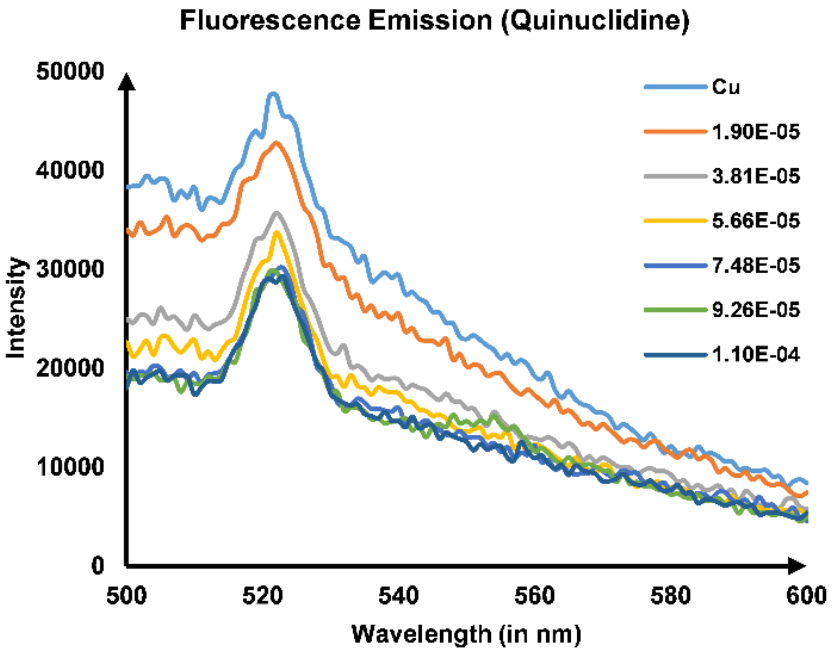

Figure S7: Fluorescence quenching of Cu(dap)Cl_2 in the presence of quinuclidine.

A stock solution **B** of ethyl nitroacetate (**2a**, 0.0015M) and quinuclidine (0.0015 M) was prepared. 10 µL of the stock solution **A** was added to 1 mL of $CHCl_3$, followed by the stepwise addition of 10 µL **B**. Emission intensities were measured after each addition.

A stock solution **C** of ethyl nitroacetate (**2a**, 0.0015 M) was prepared. 10 µL of the stock solution **A** was added to 1 mL of $CHCl_3$, followed by the stepwise addition of 10 µL **C**. Emission intensities were measured after each addition.

A stock solution **D** of styrene (**1a**, 0.0015 M) was prepared. 10 µL of the stock solution **A** was added to 1 mL of $CHCl_3$, followed by the stepwise addition of 10 µL **D**. Emission intensities were measured after each addition.

A stock solution **E** of ethyl nitroacetate (**2a**, 0.0015 M), quinuclidine (0.0015 M) and styrene (0.0015 M) were prepared. 10 µL of the stock solution **A** was added to 1 mL of $CHCl_3$, followed by the stepwise addition of 10 µL **C**. Emission intensities were measured after each addition.

A stock solution **F** of phenyl nitromethane (**2b**, 0.0015 M) and quinuclidine (0.0015 M) were prepared. 10 µL of the stock solution **A** was added to 1 mL of $CHCl_3$, followed by the stepwise addition of 10 µL **F**. Emission intensities were measured after each addition.

A stock solution **G** of ethyl nitro acetate (**2a**, 0.0015 M), quinuclidine (0.0015 M) and styrene (0.0015 M) was prepared. 10 µL of the stock solution **A** was added to 1 mL of $CHCl_3$, followed by the stepwise addition of 10 µL **G**. Emission intensities were measured after each addition.

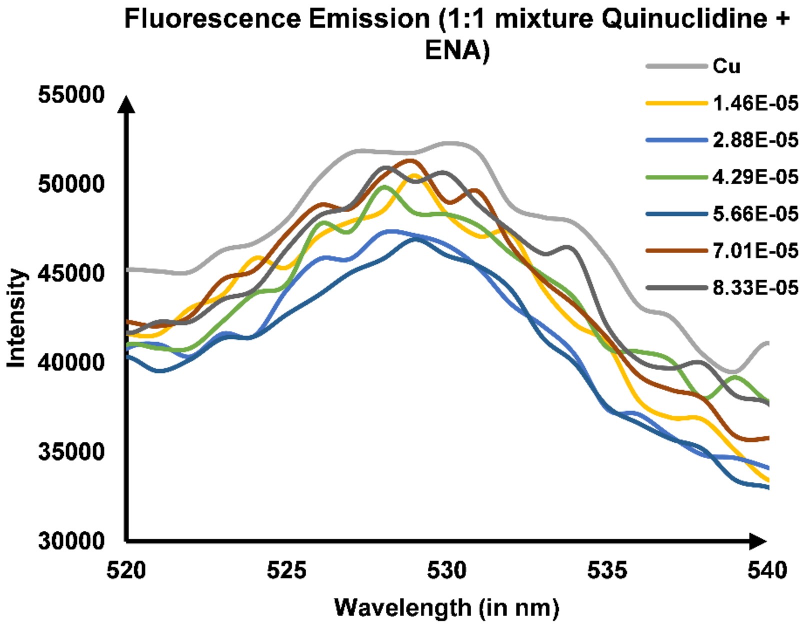

Figure S8: Fluorescence quenching of $Cu(dap)Cl_2$ by 1:1 mixture of quinuclidine and ethyl nitroacetate (**2a**).

Fluorescence quenching of the photocatalyst by quinuclidine, ethyl nitroacetate (**2a**), a 1:1 mixture of quinuclidine and ethyl nitroacetate (**2a**), styrene (**1a**), and the full reaction mixture was analyzed by plotting [I_0/I] versus C (Figure S9). The same was performed for phenyl nitromethane (**2b**).

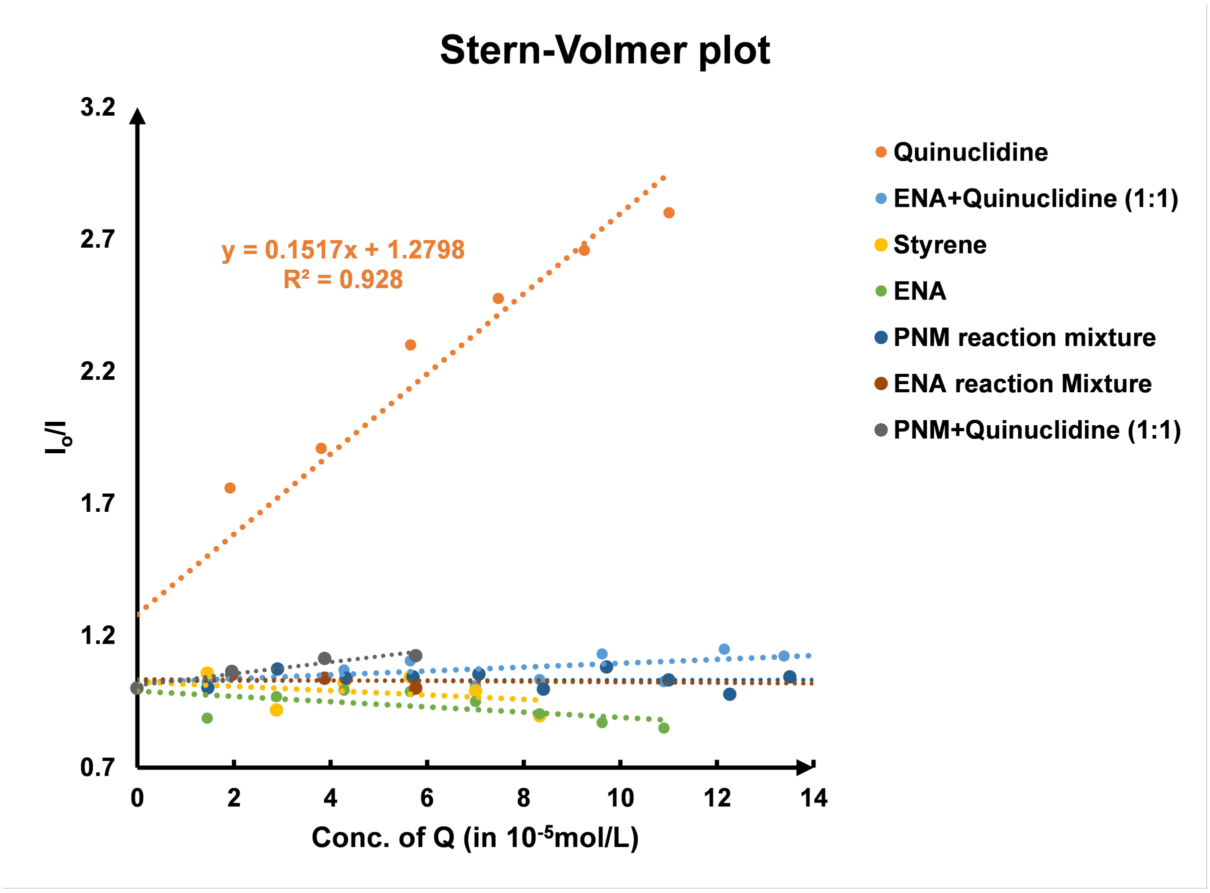

Figure S9: Stern-Volmer plot. ENA (ethyl nitroacetate), PNM (phenyl nitromethane).

8.4 UV-Visible and spectroelectrochemistry studies

For spectroelectrochemistry studies:

A stock solution of 2ml of Cu(dap)Cl_2 (0.01 M) was prepared in HPLC-grade MeCN with $TBABF_4$ (0.01 M) as supporting electrolyte. Spectroelectrochemistry was performed with a 50 µL aliquot of this solution. The sweep was carried out between −0.4 V to +0.45 V, and the initial scan in negative direction was started from +0.15 V with in-situ UV-Vis spectra recorded with every 0.05 V change in potential. The 1e^- reduction of Cu(II) in Cu(dap)Cl_2 to form Cu(I) is marked by an increase in absorption in the 440 nm region, and this peak progressively disappears when this Cu(I) is re-oxidized to Cu(II).

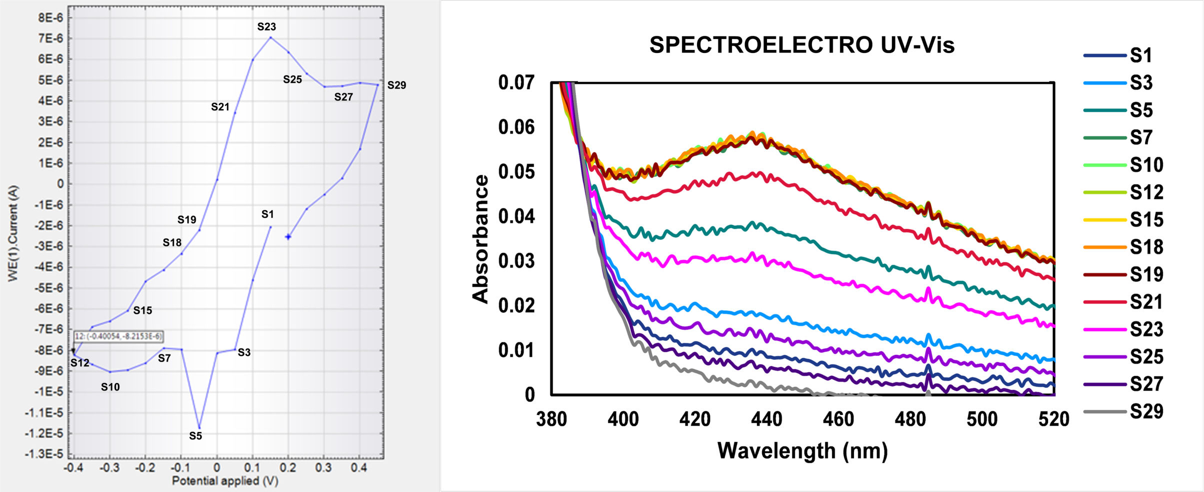

Figure S10: (Left)Points S1, S2, …S12 corresponds to the reduction of Cu(II) to Cu(I), resulting in a rise in the characteristic peak of Cu(I) around 440nm in the UV spectra. (Right) Spectra recorded at points S7-to-S19 have the same absorbance at 440 nm, suggesting the presence of Cu(I). The intensity of this peak starts to decrease at S21 and subsequently disappears in points S23-S29.

For UV-Visible studies:

Three stock solutions were prepared at a concentration of 10 mM each in $CHCl_3$, i.e. Cu(dap)Cl_2 (**D**), quinuclidine (**E**), ethyl nitroacetate (**F**), phenyl nitromethane (**G**), and styrene (**H**). UV-Vis measurements were then conducted to monitor the changes in absorbance throughout the reaction process. Initially, a 2 µL aliquot of the stock solution **D** was added to a cuvette containing 2 mL of $CHCl_3$, and the UV-Vis spectrum was recorded. Subsequently, a 10 µL aliquot of the stock solution **E** was added, and its absorption spectrum was again recorded. Subsequently, a 10 µL aliquot of the stock solution **F** was added, and the UV-Vis spectrum was recorded to obtain the initial spectrum of the reaction mixture. The reaction mixture was irradiated at 455 nm. Absorption spectra were recorded after 5, 20 and 40 minutes to monitor the progress of the reaction. Cyclic voltammetry from spectroelectrochemical studies showed that as the photocatalyst was progressively reduced from the +2 to the +1 state, the absorbance in the region of 440 nm increased. The corresponding rise in characteristic absorption at this wavelength, as confirmed by UV-Vis kinetic measurements, was indicative of the formation of the +1 state of the photocatalyst in the reaction mixture. The study was repeated by adding 10µL aliquot of stock solution **H**, but the characteristics of the curve did not change indicating no-role of styrene in the initiation step. The study was repeated for the full reaction mixture with stock solution **G** instead of **F**, and the same characteristics were observed thus suggesting similar modes of activation.

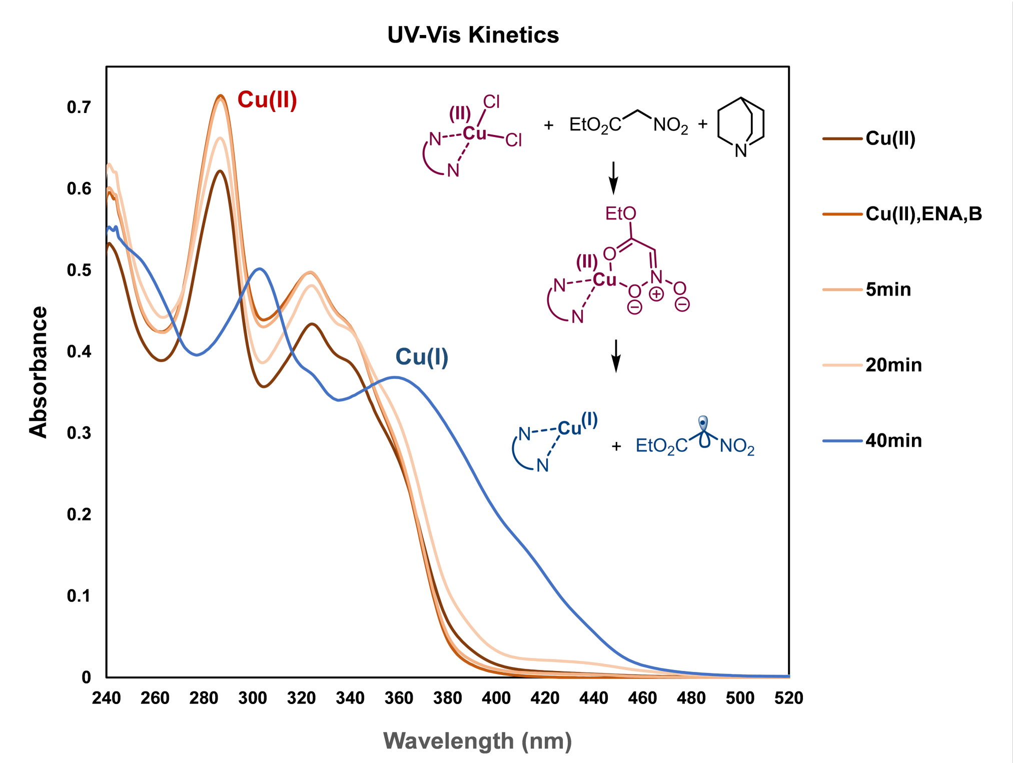

Figure S11: After 40 min of irradiation, the rise of absorption intensity at 440-450 nm is suggestive of formation of Cu(I).

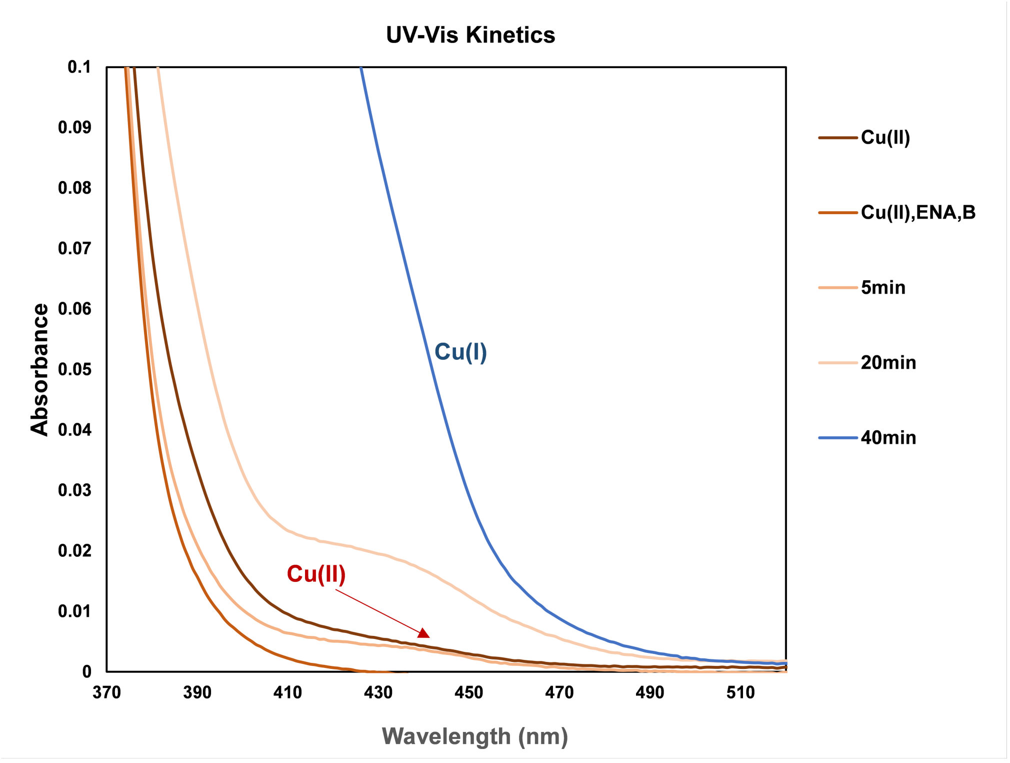

Figure S12: Zoomed-in for 370-520 nm.

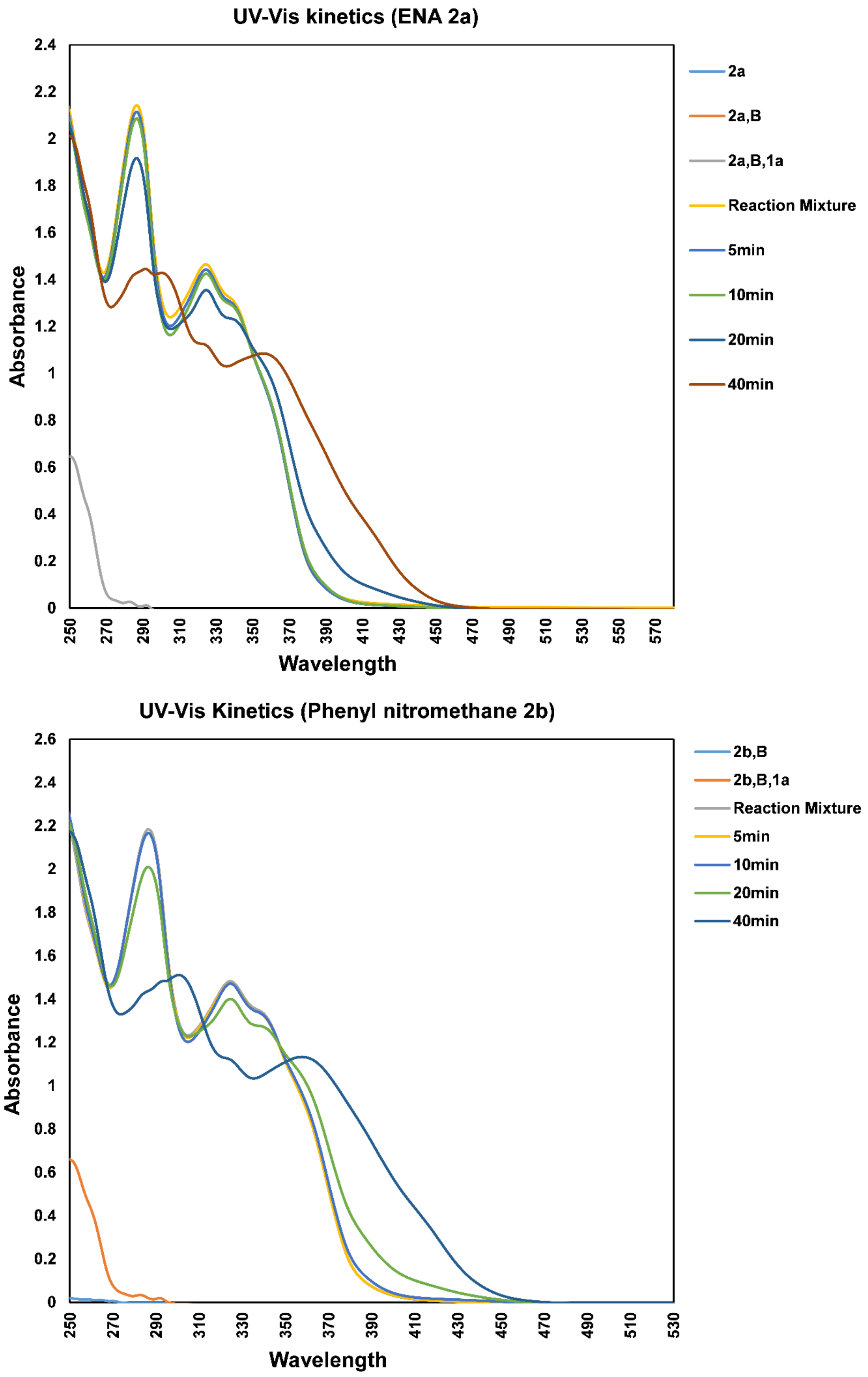

Figure S13: Comparison of UV-vis kinetics of reaction mixtures of ethyl nitroacetate **2a** and phenyl nitromethane **2b**.

UV-Visible spectra of reaction mixture:

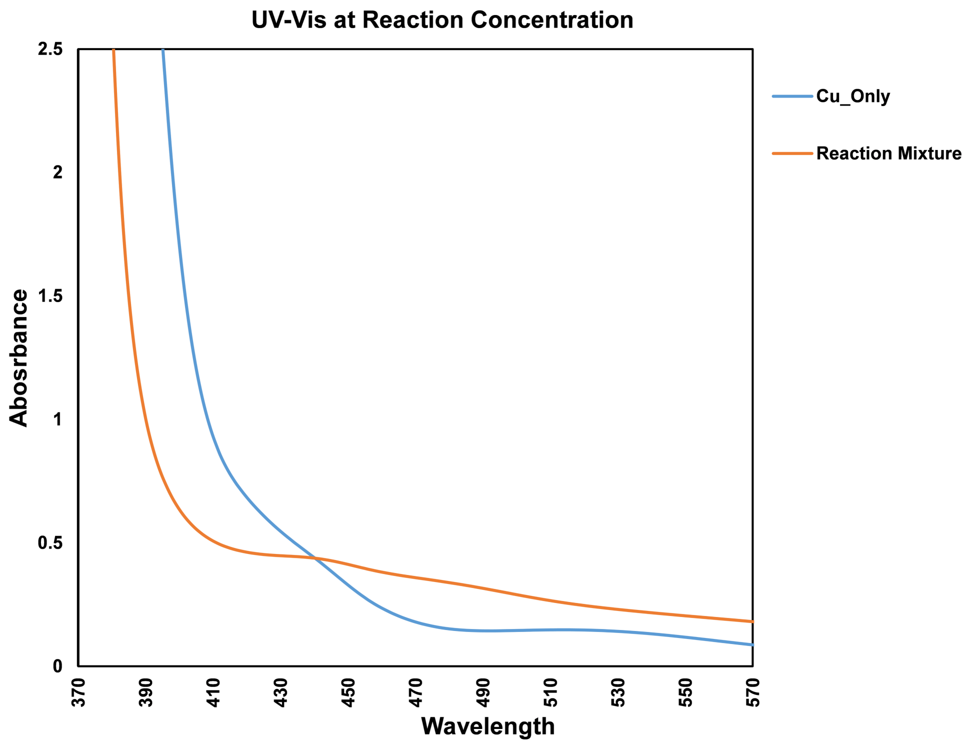

Figure S14: Absorption spectra of the reaction mixture and the Cu-PC alone under the concentration of the reaction conditions.

8.5 Electron Paramagnetic Resonance (EPR) analysis

Continuous wave (CW) EPR spectra were obtained using a Bruker MagnetTech MiniScope MS 400 benchtop EPR spectrometer instrument with X-band of 9.30-9.55 GHz. The spectral data was collected at 77K with the following spectrometer settings: microwave power = 0.48 mW, centre field = 333.59 mT, sweep width = 50.00 mT, sweep time = 30s, modulation frequency = 9.45 GHz, modulation amplitude = 0.1 mT, MW attenuation = 20 db.

Experiment 1 (Figure S15, left): A glass reaction vial (5 mL) equipped with a Teflon-coated magnetic stirring bar was charged with [Cu(dap)Cl_2] (1.14 mg, 0.01 equiv., 1.0 mol%) and quinuclidine (25.4 mg, 0.2 mmol, 1.0 equiv.) followed by the addition of 1.5 mL $CHCl_3$. Ethyl nitroacetate (33.27 µL, 0.3 mmol, 1.5 equiv.) was added to the above reaction mixture *via* a microsyringe. The vial was irradiated at 455 nm (reaction setup Figure S1) at room temperature for 4 h, upon which 500 µL was transferred into the EPR tube, which was cooled down to 77K using a Dewar flask containing liquid nitrogen. The EPR spectra was immediately recorded. At 77K, a characteristic signal of an organic radical with g-value of 2.002 was observed.

Experiment 2 (Figure S15, right): A glass reaction vial (5 mL) equipped with a Teflon-coated magnetic stirring bar was charged with 5,5-dimethyl-1-pyrroline-N-oxide (DMPO, 0.2 mmol, 1.0 equiv.), [Cu(dap)Cl_2] (1.14 mg, 0.01 equiv., 1.0 mol%) and quinuclidine (25.4 mg, 0.2 mmol, 1.0 equiv.) followed by the addition of 1.5 mL $CHCl_3$. Ethyl nitroacetate (22.18 µL, 0.2 mmol, 1.0 equiv.) was added to the above reaction mixture *via* a microsyringe. The vial was

irradiated at 455 nm (reaction setup Figure S1) at room temperature for 4 h, upon which 500 μL was transferred into the EPR tube and the spectra was recorded at room temperature. The EPR signal of the DMPO-trapped radical was observed at room temperature. The radical is carbon-centered according to the hyper-fine splitting constants.

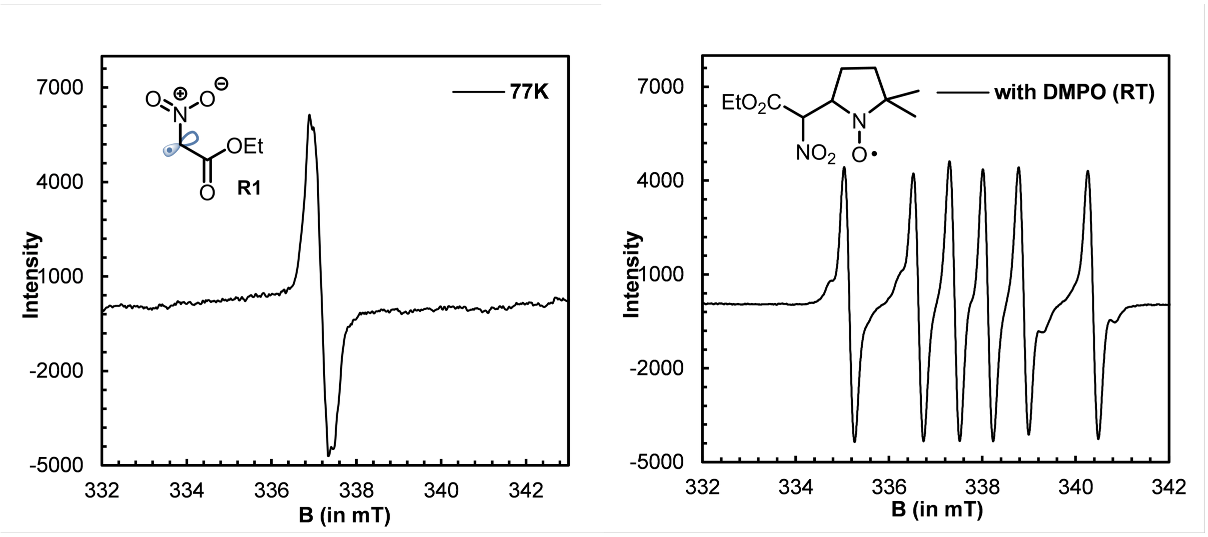

Figure S15: EPR spectrums at 77K (left) and at room temperature with DMPO (right).

8.6 Density Functional Theory (DFT) studies

Computational details:

All the DFT calculations were performed using ORCA 5.0.4 software.[22] Fully optimized structures were characterized to be either local minima or saddle points of first order, i.e. transition state structures by the number of imaginary frequencies. Minimum energy structures feature zero imaginary frequencies, whereas transition states have exactly one. The correct minimum energy pathways associated with the respective imaginary frequency of a given transition state structure was traced by an IRC calculation in the back- and forward direction. Energy values were derived from frequency analysis using the harmonic oscillator approach and include zero-point energy (ZPE) correction. Values of enthalpy (H) and Gibb's free energy (G) are unscaled and are reported for standard conditions if not stated otherwise. All the structures were rendered in CYLView20.[23]

Energy Barriers: Radical addition vs Anionic addition vs Cu-coordinated concerted

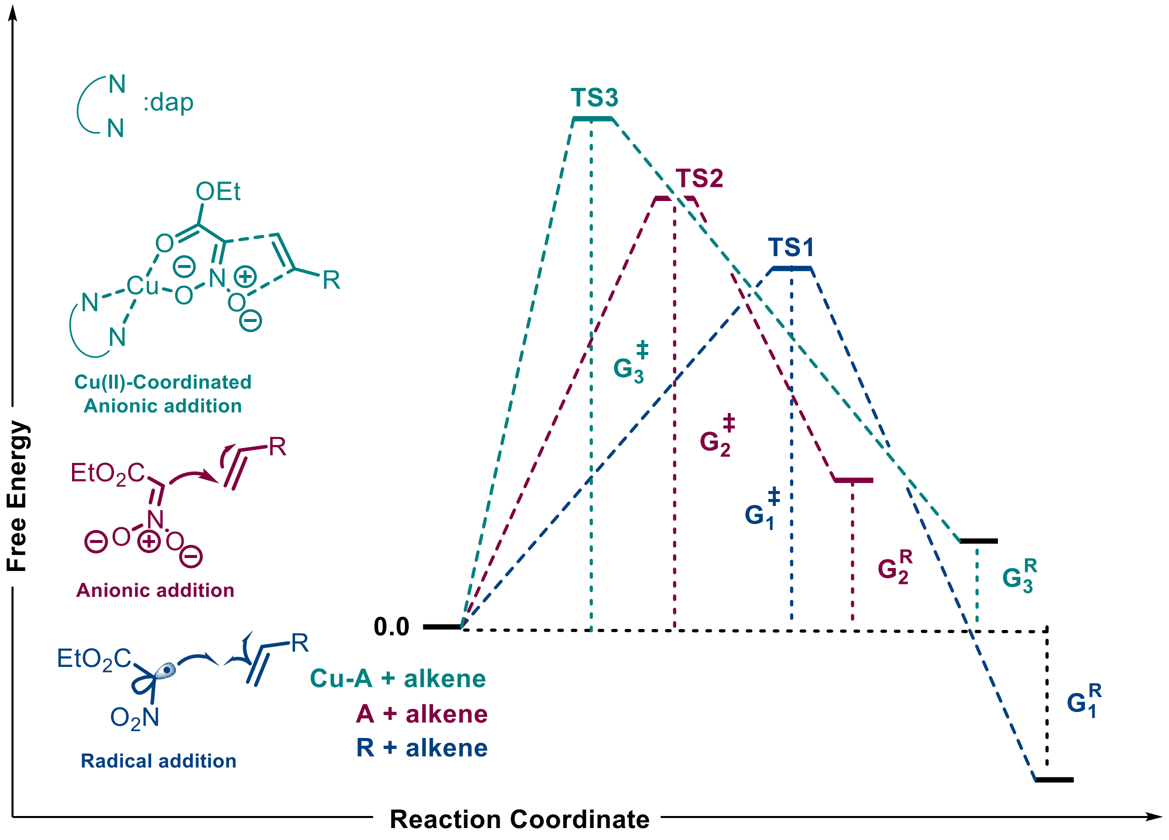

Figure S16: Reaction profile

To establish a trend for this specific reaction, the barriers for radical addition, anionic addition and Cu-coordinated cycloaddition to alkenes were calculated for selected substrates. The geometries were optimized, and frequency analysis was performed at M06-2x/6-31G(d)[24] level of theory in the gas phase and single point calculation in M06-2x/6-31++G (d, p) level of theory to improve the electronic energy. The CPCM model was implemented to account for implicit solvation.[26] Each TS was confirmed by a single imaginary frequency and IRC, and the local minima was confirmed by all positive frequencies.

Due to coordination with Cu (II) the anion **A** is less nucleophilic than the uncoordinated **A**, and hence the barriers for such an addition are high. Hence, for a better comparison the barriers of addition *via* anion **A** and addition *via* radical **R** are computed for electronically different substrates to establish the trend in reactivity. As the alkenes becomes more electron-deficient, the barrier for anion addition ($\Delta G_2^{‡}$) decreases and that of radical addition ($\Delta G_1^{‡}$) increases. For acrylonitrile (**1ab**), the barrier for the anionic addition is lower than that of radical addition, hence, the reaction with this substrate most likely proceeds *via* this pathway.

The barriers are tabulated in Table S7.

Table S7: Comparing radical addition vs anionic addition vs Cu-coordinated concerted pathway (all energies are in kcal·mol^{-1}).

Alkene/Alkyne	$\Delta G_1^{‡}$	$\Delta G_2^{‡}$	$\Delta G_3^{‡}$ ($\Delta G_4^{‡}$)[a]	ΔG_1^{R}	ΔG_2^{R}	ΔG_3^{R} (ΔG_4^{R})[a]
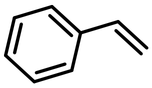 **1a**	8.9	36.6	39.5 (43.8)	-20.1	32.0	2.7 (7.6)
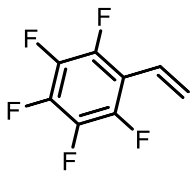 **1j**	9.8	26.4	38.6 (42.6)	-18.6	23.4	4.8 (7.7)
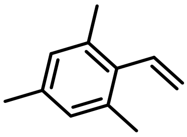 **1k**	9.2	37.8	-	-17.5	36.9	-
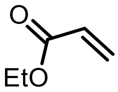 **1aa**	14.1	25.0	36.8 (43.1)	-16.0	20.1	4.2 (7.7)
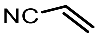 **1ab**	14.1	21.4	35.6 (36.9)	-17.9	16.3	-
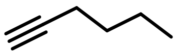 **5d**	12.5	36.6	-	-	-	-

[a]$\Delta G_4^{‡}$ ΔG_4^{R}: Cu-coordinated with acetate as ligand, all the calculations are in gas-phase.

Comparison of HAT vs Deprotonation:

Quinuclidine representing a tertiary amine quenches the photocatalyst *via* oxidation to the corresponding nitrogen centered radical cation, which can effectively abstract the active hydrogen (Hydrogen Atom Transfer or HAT) of ethyl nitroacetate (ENA) to form the ENA radical **R**. It can also act as a base and deprotonate ENA to form ENA anion **A**, which can then quench the photocatalyst. To compare both the two processes, the corresponding activation barriers for HAT and deprotonation were calculated in the gas phase M06-2X/6-31++G(d,p)//M06-2X/6-31G(d)[24] level of theory with CPCM[26] solvation model.

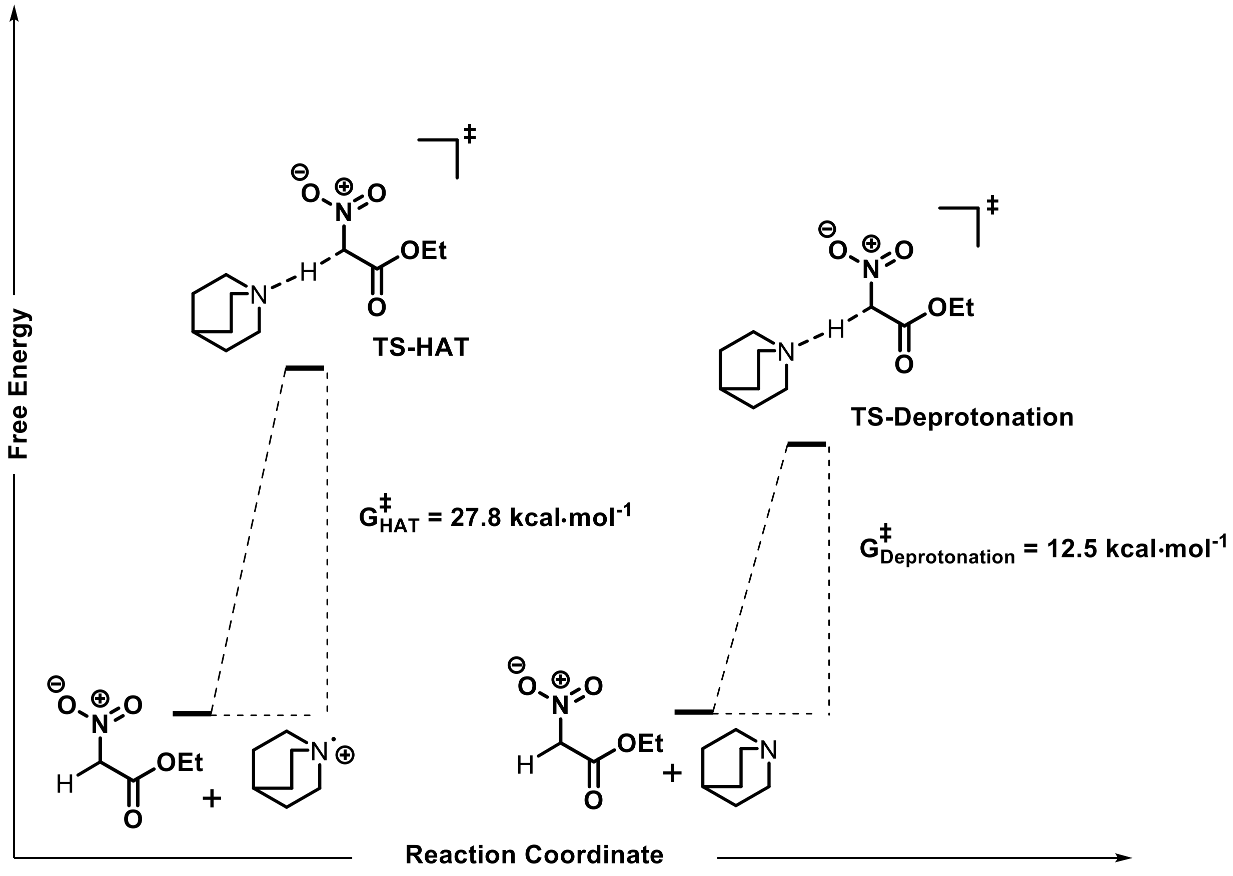

Figure S17: HAT vs deprotonation.

Calculation of redox potentials:

$E_{1/2}$°(Qui/Qui·+) and $E°_{1/2}$ (**A**/**R**) are computed by the method reported by Nicewicz[25] *et al.* where M06-2X/6-31+G(d, p)[24] in CPCM solvation model[26] was found to be one of the best methods for prediction of redox potentials of carbon and nitrogen centered radicals and hence it is the method used for the calculation.

$$E°_{1/2} = \frac{-(G_{reduced} - G_{oxidized})}{nF} - E°_{1/2}\,(\mathrm{SHE})$$

n=1, F = 23.061 kcal·mol^{-1}V^{-1}, $E°_{1/2}$ (SHE) = 4.281 V

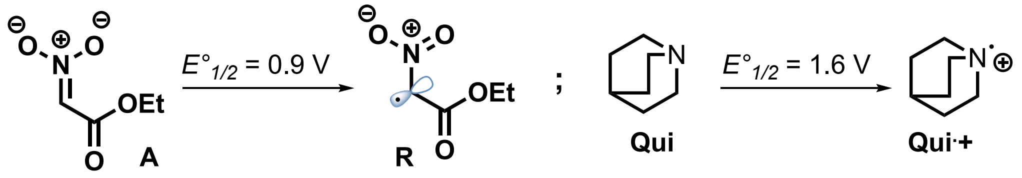

Figure S18: Calculated E°$_{1/2}$.

Radical clock experiment calculation:

The rearranged product **3ah'** that would arise *via* opening of cyclopropyl ring was not observed, instead, the [3+2] anellation at the alkene was observed.

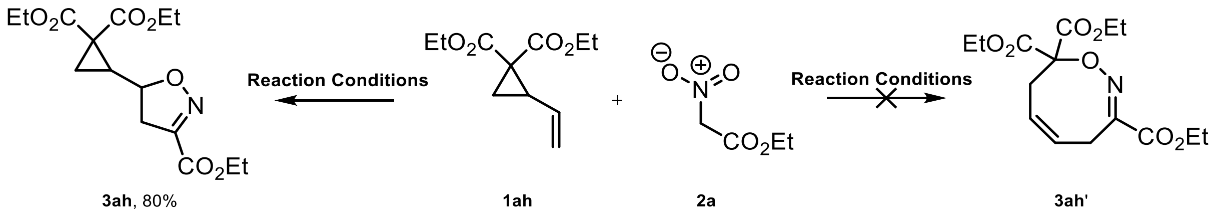

Figure S19: Radical clock reaction.

Upon radical addition to the alkene, intermediate **1ah-I** is formed. From this species, two divergent pathways are possible: (i) ring-opening of the strained cyclopropyl group via **TS1-b$_{1ah}$**, leading to intermediate **1ah-VII**, and (ii) cyclization involving the nitro group's oxygen via **TS1-a$_{1ah}$**, forming **1ah-III**.

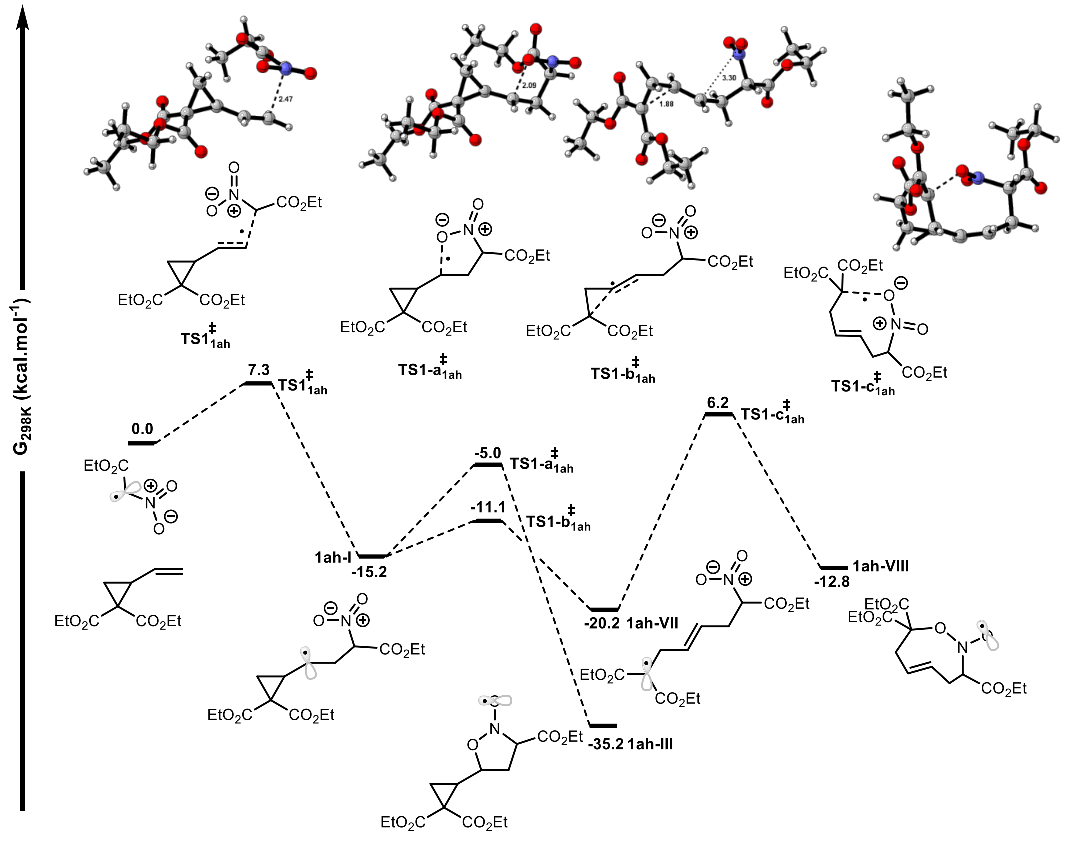

Figure S20: Full reaction profile. Level of theory: M06-2x/6-31++G (d, p)//M06-2x/6-31G(d) (CPCM: Chloroform).

The activation barrier for ring-opening is lower by 6.1 kcal·mol⁻¹, making the formation of **1ah-VII** kinetically favored. However, the subsequent formation of an eight-membered ring **1ah-VIII** has a high free-energy barrier (26.4 kcal·mol⁻¹), whereas reversion to **1ah-I** via reforming the cyclopropyl ring requires only 9.1 kcal·mol⁻¹. As a result, **1ah-VII** and **1ah-I** are likely in rapid equilibrium. In contrast, the formation of **1ah-III** from **1ah-I** via **TS1-a$_{1ah}$** is thermodynamically favored, with **1ah-III** being more stable by 15.0 kcal·mol⁻¹ than **1ah-VII**. Therefore, despite the kinetic preference for **1ah-VII**, the reaction ultimately proceeds through the thermodynamically favored formation of **1ah-III**.

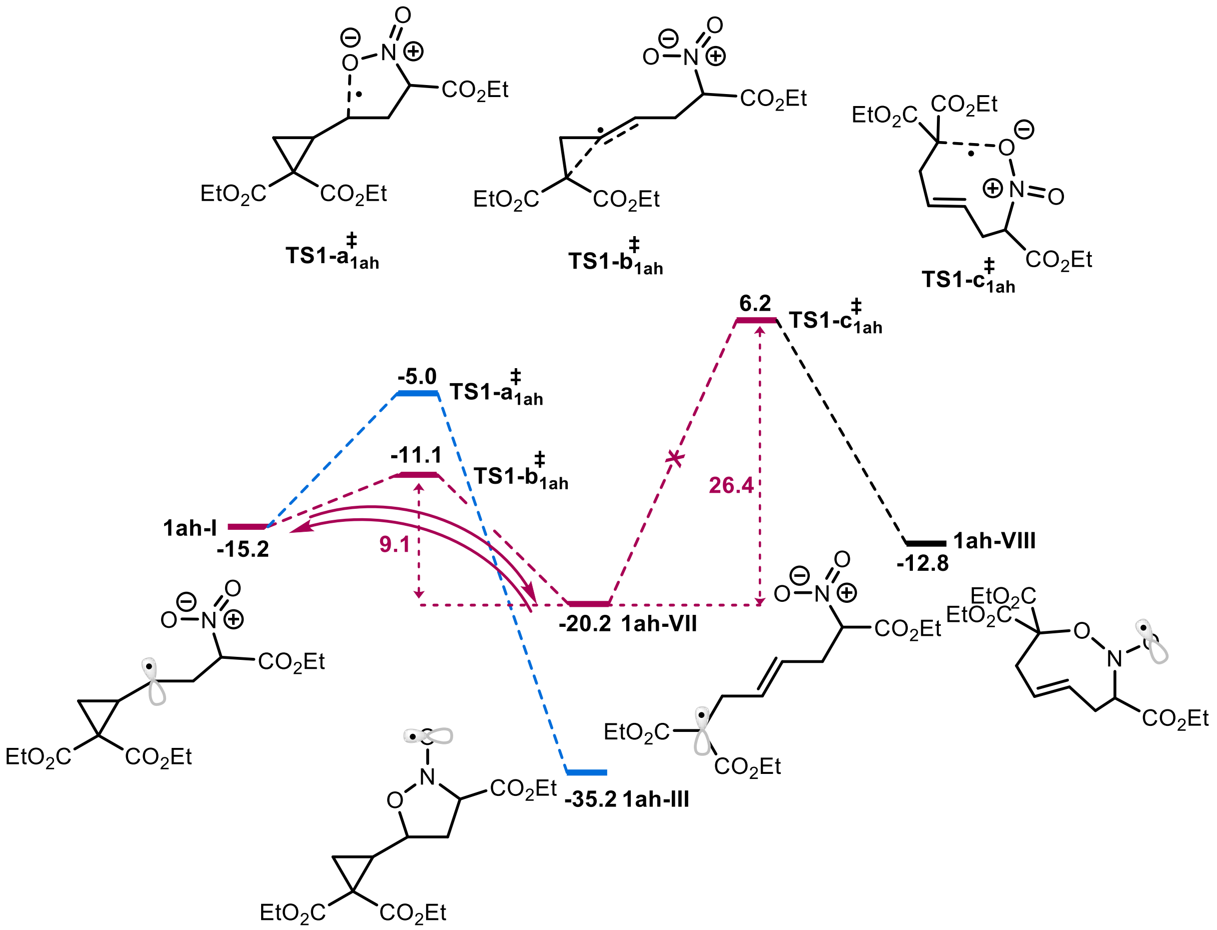

Figure S21: Kinetic vs Thermodynamic pathways.

Global electrophilicity indices (ω):

All the canonical molecular orbital energies were computed using Natural Bond Order (NBO) analysis in M06-2X/6-31++G(d,p)[24] level of theory, based on geometries optimized at the M06-2X/6-31G(d)[24] level in NBO 7.0 program.[27] Global electrophilicities of ethyl nitroacetate and phenyl nitromethane radicals and alkenes **1a**, **1j**, **1aa** and **1ab** were computed according to Domingo[28] et al. using ε_{HOMO} and ε_{LUMO} energies.

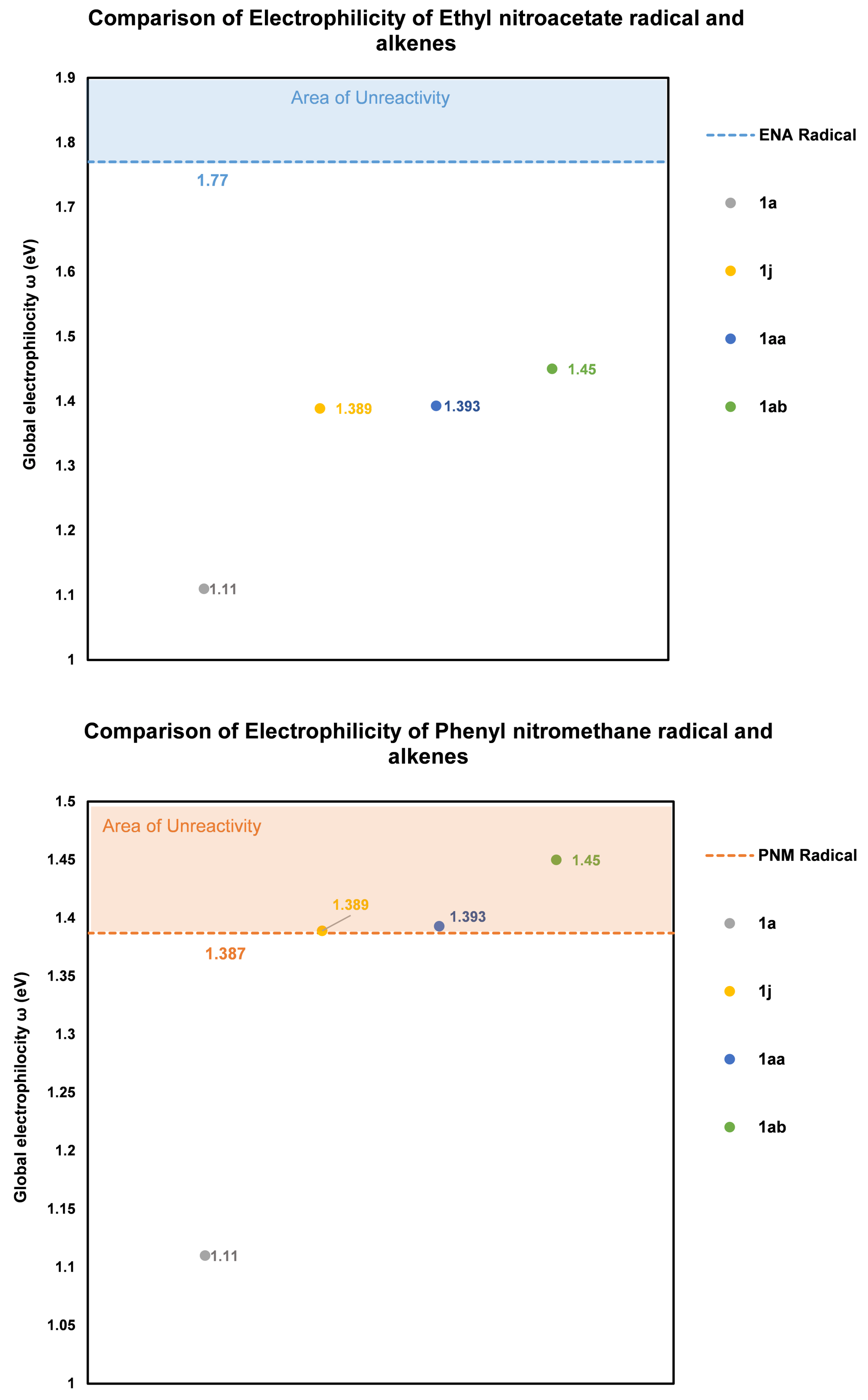

Figure S22: Global Electrophilicity indices of radicals and alkenes in eV.

Role of Dispersion: To check the role of dispersion, the free energies of transition states of anionic ($TS2_{1j}$)and radical addition ($TS1_{1j}$) on pentafluorostyrene (**1j**). The geometries showed negligible changes and the resulting free energy differences did not qualitatively alter the reaction profile. Therefore, we conclude that the inclusion of empirical dispersion corrections does not significantly affect the mechanistic insights presented in this work. The reason could be attributed to the highly parameterized meta-hybrid GGA functional M06-2x, which is designed to account for short- to medium-range dispersion interactions and empirical addition of dispersion could lead to over binding.[29]

Table S8: Comparing free energies with and without dispersion.

**Transition States**	**Free Energy G_1, M06-2x/6-31G(d) (in a.u.)**	**Free Energy G_2, M06-2x-D3/6-31G(d) (in a.u.)**	**$\Delta G = G_2 - G_1$ (in kcal.mol^{-1})**
$TS1_{1j}$	-1316.66495082	-1316.66329474	-1.62
$TS2_{1j}$	-1316.81296404	-1316.81554245	-1.04

Radical Stabilization Energy Calculation: The enthalpy of the reaction that generates a radical by abstraction of hydrogen by a methyl radical is referred to as radical stabilization energy.[30] All the enthalpies are computed in M06-2X/6-31G(d) level of theory.

Table S9: Radical Stabilization Energy.

**Reaction**	$R\text{-}H + \dot{C}H_3 \longrightarrow \dot{R} + CH_4 \quad H_R = RSE$	
Cmpd	**Radical**	**Radical Stabilization Energy (kcal.mol^{-1})**
Ethyl Nitro Radical	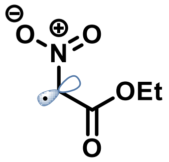	-6.2
Phenyl Nitro Radical	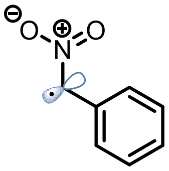	-17.5

Optimized Coordinates and Energies:

Optimized coordinates

Alkenes

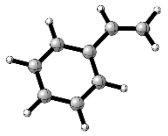	**1a**	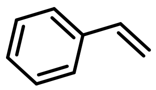

Charge: 0
Multiplicity: 1
[CPCM(CHCl3)M06-2X/6-31G(d)]G_{298K}: -309.3961624
[CPCM(CHCl3)M06-2X/6-31G(d)]H_{298K}: -309.35792174
[CPCM(CHCl3)M06-2X/6-31G(d)]E_{SCF}: -309.50107571
[(CPCM(CHCl3)M06-2X/6-31++G(d, p)]E_{SCF}: -309.52229051

C -1.06719893163684 2.02111974540042 -0.84048589909333
H -1.67358633512991 2.90916596201123 -0.69597660860294
H -1.45383663737619 1.27526031015470 -1.52921888972951
C 0.09556133377310 1.87712839661261 -0.20099916913969
H 0.43056837355360 2.67496129297672 0.46095149868374
C 1.02015701047008 0.73149228095727 -0.30262528093447
C 2.28513466531150 0.82873082185546 0.29064567624285
C 0.68788263953222 -0.45681566000216 -0.96995012865584
C 3.20022140853303 -0.21794170101848 0.20895162836035
H 2.55326767954009 1.74049930602127 0.81878142492784
C 1.59924950552587 -1.50330961724766 -1.05188007745651
H -0.29352783321528 -0.56935133373478 -1.42123689278220
C 2.86064046467435 -1.38882533586599 -0.46487775430844
H 4.17729420240280 -0.11738129573036 0.67166718358723
H 1.32311698307572 -2.41496361296854 -1.57328910223960
H 3.56984894096585 -2.20806333942171 -0.53120538885943

Charge: 0
Multiplicity: 1
[M06-2X/6-31G(d)]G_{298K}: -309.39162508
[M06-2X/6-31G(d)]H_{298K}: -309.35331724
[M06-2X/6-31G(d)]E_{SCF}: -309.49639152
[M06-2X/6-31++G(d, p)]E_{SCF}: -309.51715020

C -1.04311773081173 2.03542290009136 -0.87145790612533
H -1.66510646863599 2.91077320747252 -0.71955159335538
H -1.37941939849920 1.32394281074173 -1.61989914375949
C 0.07998580761477 1.86143627091200 -0.17381881154911
H 0.37423006970935 2.62775106524622 0.54218944618361
C 1.01190735223688 0.72401312365871 -0.28353863719619
C 2.28564364431314 0.83022545283580 0.28625255371964
C 0.67495906289725 -0.46828816196127 -0.93826193102701
C 3.20576975006123 -0.20886794905068 0.18798793764100
H 2.55768219321988 1.74501389025662 0.80691983499468
C 1.59097438354715 -1.50783468344204 -1.03714972276569
H -0.31795736520904 -0.58867071257815 -1.36115784727508

C 2.86227674210692 -1.38233314048478 -0.47732132977373
H 4.19057339067943 -0.10103437781904 0.63188831535133
H 1.31012296089281 -2.42375850487307 -1.54813823520913
H 3.57626907587715 -2.19608497100593 -0.55569070985410

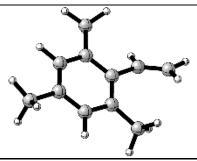	**1k**	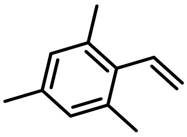

Charge: 0
Multiplicity: 1
[CPCM(CHCl3)M06-2X/6-31G(d)]G_{298K}: -427.20200118
[CPCM(CHCl3)M06-2X/6-31G(d)]H_{298K}: -427.15417962
[CPCM(CHCl3)M06-2X/6-31G(d)]E_{SCF}: -427.38656161
[(CPCM(CHCl3)M06-2X/6-31++G(d, p)]E_{SCF}: -427.41455943

C -0.87429881303461 2.26633647343454 -0.96701633001750
H -1.52259083313501 3.10487418859262 -0.73159079813107
H -0.96080313427650 1.84348249583264 -1.96323323824177
C -0.00301405014913 1.80328631338364 -0.06711352666239
H 0.04837478756466 2.31037914898394 0.89587015447941
C 0.95374599010867 0.68750526851316 -0.24788190831450
C 2.24871576921041 0.84024377956992 0.29159452397378
C 0.61742018514051 -0.51690627311062 -0.90207894603803
C 3.18494766479177 -0.18309486643693 0.15084978051652
C 1.58191588182440 -1.52089157335469 -1.01054811977383
C 2.87121002805583 -1.37572644908250 -0.49970063542683
H 4.18331478303799 -0.04518279018171 0.56126523951656
H 1.31227195887778 -2.45063969600283 -1.50892690942051
C -0.75387386111755 -0.78865169365929 -1.47449565219164
H -1.54379595937371 -0.34951832176167 -0.85951854204361
H -0.86142024514393 -0.37250768593477 -2.48236180252999
H -0.92116555960463 -1.86652976496335 -1.54703063750146
C 3.88278791015117 -2.48560075817987 -0.63569142716422
H 3.57983076081694 -3.36519062219294 -0.05726242946179
H 3.98154947675509 -2.80214714810295 -1.67907413389186
H 4.86617754575707 -2.16860201095041 -0.27910139575003
C 2.64221730296477 2.11032857789845 1.00758059588945
H 2.45176126082620 2.99480115139179 0.39104206704178
H 2.07511788242462 2.23863379534586 1.93705157618047
H 3.70420354752715 2.09284068096798 1.26417243496310

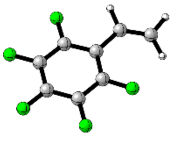	**1j**	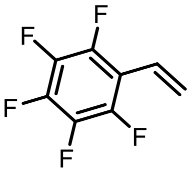

Charge: 0

Multiplicity: 1
[CPCM(CHCl3)M06-2X/6-31G(d)]G_{298K}: -805.43376620
[CPCM(CHCl3)M06-2X/6-31G(d)]H_{298K}: -805.38538935
[CPCM(CHCl3)M06-2X/6-31G(d)]E_{SCF}: -805.49223446
[(CPCM(CHCl3)M06-2X/6-31++G(d, p)]E_{SCF}: -805.53900429

C	-1.14409395265587	1.98020540184724	-0.67611670081721
H	-1.68446608497625	2.91861747181981	-0.60844569947867
H	-1.70149814151035	1.11097724520413	-1.00256845534097
C	0.14952848176535	1.93912060969355	-0.34674269502030
H	0.63653372128370	2.84745538520890	-0.00367835049351
C	1.02911800462335	0.76075907401841	-0.38215474662260
C	2.25437586125579	0.80565828546303	0.29026365103882
C	0.75474010186306	-0.41828349099491	-1.08045993117183
C	3.15383582738715	-0.24888267302426	0.28068921581244
C	1.63630660673055	-1.48931474749352	-1.10186738831736
C	2.84373247518578	-1.40372308352308	-0.42260458804117
F	2.59046650830961	1.90184956015787	0.97668479288193
F	4.30742609761905	-0.16525128838971	0.93926639554748
F	3.70207506708855	-2.41688087948846	-0.44492404166642
F	1.33087814919353	-2.59415835100835	-1.78039702671328
F	-0.37430447316303	-0.54955751949064	-1.77999978159734

Charge: 0
Multiplicity: 1
[CPCM(CHCl3)M06-2X/6-31G(d)]G_{298K}: -805.43376620
[CPCM(CHCl3)M06-2X/6-31G(d)]H_{298K}: -805.38538935
[CPCM(CHCl3)M06-2X/6-31G(d)]E_{SCF}: -805.49223446
[(CPCM(CHCl3)M06-2X/6-31++G(d, p)]E_{SCF}: -805.53900429

C	-1.14409395265587	1.98020540184724	-0.67611670081721
H	-1.68446608497625	2.91861747181981	-0.60844569947867
H	-1.70149814151035	1.11097724520413	-1.00256845534097
C	0.14952848176535	1.93912060969355	-0.34674269502030
H	0.63653372128370	2.84745538520890	-0.00367835049351
C	1.02911800462335	0.76075907401841	-0.38215474662260
C	2.25437586125579	0.80565828546303	0.29026365103882
C	0.75474010186306	-0.41828349099491	-1.08045993117183
C	3.15383582738715	-0.24888267302426	0.28068921581244
C	1.63630660673055	-1.48931474749352	-1.10186738831736
C	2.84373247518578	-1.40372308352308	-0.42260458804117
F	2.59046650830961	1.90184956015787	0.97668479288193
F	4.30742609761905	-0.16525128838971	0.93926639554748
F	3.70207506708855	-2.41688087948846	-0.44492404166642
F	1.33087814919353	-2.59415835100835	-1.78039702671328
F	-0.37430447316303	-0.54955751949064	-1.77999978159734

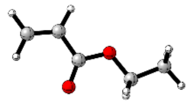	**1aa**	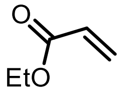

Charge: 0
Multiplicity: 1
[CPCM(CHCl3)M06-2X/6-31G(d)]G_{298K}: -345.53660411
[CPCM(CHCl3)M06-2X/6-31G(d)]H_{298K}: -345.49592378
[CPCM(CHCl3)M06-2X/6-31G(d)]E_{SCF}: -345.63063027
[(CPCM(CHCl3)M06-2X/6-31++G(d, p)]E_{SCF}-345.65345315

C	0.83667492110347	4.64371111447391	-1.25773308269165
O	1.52451344467731	4.06718581675595	-2.07371352957711
O	0.48542974077542	5.93007820450143	-1.36881431289869
C	0.98474187893984	6.61629171140552	-2.52886571296305
H	0.68387210930323	6.06087390446812	-3.42120461923011
H	2.07782103277679	6.62393871919084	-2.48699536486181
C	0.40519479340805	8.01385679572651	-2.50747034295415
H	0.71176680299576	8.54616008541466	-1.60320234430884
H	0.75928909617437	8.57315970376063	-3.37737322925750
H	-0.68694778368263	7.97669063971253	-2.53953385946618
C	0.30255784180712	3.99003028262468	-0.03652952486514
H	0.54797214216821	2.93579994678867	0.04378945144399
C	-0.40404688519359	4.63029611266479	0.89271314752419
H	-0.63567894107697	5.68647558018932	0.79751277094004
H	-0.76748526417638	4.11218814232237	1.77444817316603

Charge: 0
Multiplicity: 1
[M06-2X/6-31G(d)]G_{298K}: -345.53019380
[M06-2X/6-31G(d)]H_{298K}: -345.48947269
[M06-2X/6-31G(d)]E_{SCF}: -345.62441325
[M06-2X/6-31++G(d, p)]E_{SCF}: -345.64622245

C	0.83719185239764	4.64400518564177	-1.26024267999092
O	1.52177560186038	4.07400554940267	-2.07649398052412
O	0.47921773748006	5.93479809185089	-1.36575912559827
C	0.98088846134482	6.61265809361076	-2.52397412418987
H	0.68292655267793	6.05513206468971	-3.41645856301540
H	2.07456770542111	6.61350417545444	-2.48585838443796
C	0.40725501944710	8.01324390132926	-2.50684846395905
H	0.71376259029649	8.54515301756396	-1.60254198779701
H	0.76066480598796	8.57361106335923	-3.37648528772940
H	-0.68489472735582	7.97985224803020	-2.53597978870855
C	0.30556373877869	3.99114312426882	-0.03604874231420

H	0.55831776759369	2.93893004054220	0.04375466276611
C	-0.40452637776434	4.63022608696358	0.88969492093343
H	-0.63964160851457	5.68443139582046	0.78643788329769
H	-0.76739418965114	4.11604272147200	1.77383128126754

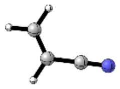	**1ab**	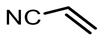

Charge: 0
Multiplicity: 1
[CPCM(CHCl3)M06-2X/6-31G(d)]G_{298K}: -170.73140789
[CPCM(CHCl3)M06-2X/6-31G(d)]H_{298K}: -170.70057299
[CPCM(CHCl3)M06-2X/6-31G(d)]E_{SCF}: -170.75749788
[(CPCM(CHCl3)M06-2X/6-31++G(d, p)]E_{SCF}: -170.76884383

C	1.39626625115481	-2.33343986142955	-0.47627304565361
C	1.29510465607526	-1.10288647754061	0.02956703322980
H	1.12666354034628	-3.21865816848269	0.09085407760790
H	1.56706591301912	-0.22537529737844	-0.54805031576692
H	0.93296816675644	-0.95393805019406	1.04102791191285
C	1.87246546016167	-2.55644297035191	-1.81379266932809
N	2.26360871248642	-2.74694346462276	-2.88661819200193

Charge: 0
Multiplicity: 1
[M06-2X/6-31G(d)]G_{298K}: -170.72437873
[M06-2X/6-31G(d)]H_{298K}: -170.69347353
[M06-2X/6-31G(d)]E_{SCF}: -170.75038691
[M06-2X/6-31++G(d, p)]E_{SCF}: -170.76115580

C	1.39758616192802	-2.33084414043542	-0.47928895904320
C	1.29432620010193	-1.10382148858151	0.03154353234827
H	1.12699293794383	-3.21370004355157	0.09103080817613
H	1.56571198610465	-0.22569790788009	-0.54448923698576
H	0.93235336783145	-0.95438773504508	1.04274855078973
C	1.87292585505064	-2.55859199997926	-1.81653744156695
N	2.26424619103948	-2.75064097452708	-2.88829245371822

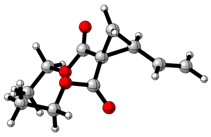	**1ah**	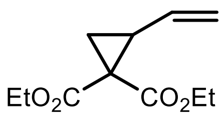

Charge: 0
Multiplicity: 1

[CPCM(CHCl3)M06-2X/6-31G(d)]G_{298K}: -729.15011095
[CPCM(CHCl3)M06-2X/6-31G(d)]H_{298K}: -729.09250327
[CPCM(CHCl3)M06-2X/6-31G(d)]E_{SCF}: -729.37119888
[(CPCM(CHCl3)M06-2X/6-31++G(d, p)]E_{SCF}: -729.41401369

C	0.43490751279613	-1.97758590429297	0.44394590060083
C	1.48985319754699	-1.32685941936812	-0.04280133077147
H	0.40630100393229	-2.27098197347705	1.49160994623471
H	1.53534991744531	-1.02630846111975	-1.08659297786942
H	2.33764307307718	-1.07277128374146	0.58494182818653
C	-0.75462656737499	-2.30976325414911	-0.37326916594719
C	-1.48870632413159	-3.63220616799037	-0.18315064580239
C	-2.11722433727502	-2.33098058362651	0.24504846352861
H	-0.71224476588944	-1.99128352887528	-1.41165323288437
H	-2.95768127329724	-1.98785753326058	-0.34863242707941
H	-2.18506224949207	-2.16296563469584	1.31592169654428
C	-2.03507248268431	-4.23189872947196	-1.43779042274811
O	-2.50033072997282	-3.58592189561425	-2.34996799912291
O	-1.93042119012175	-5.56129866506053	-1.43888748741496
C	-1.03904336220392	-4.62093791435005	0.85860504876318
O	0.11012587418095	-4.90182582565596	1.10596483911103
O	-2.08846207234691	-5.16755263698302	1.47416847121254
C	-2.41052368574809	-6.23396474651733	-2.61937011225066
C	-2.26383871269271	-7.71888637789451	-2.37321277079402
H	-1.81826197440804	-5.89606063126236	-3.47419522383105
H	-3.45095102303770	-5.94337614701778	-2.78628457619200
H	-2.60434259212477	-8.27249014141374	-3.25208042824781
H	-1.21842849306246	-7.97547974388134	-2.18291629664903
H	-2.86376622873667	-8.02651157824024	-1.51253681388747
C	-1.79000919403687	-6.20704848066781	2.42455067416315
C	-3.11092524796143	-6.70141768726118	2.97143067720321
H	-1.14754878619839	-5.79264082018967	3.20624377666099
H	-1.23594461154826	-6.99756685903012	1.91038551880000
H	-2.93320066040326	-7.49280226228153	3.70420806557174
H	-3.65316469141818	-5.88851453955799	3.46148826407684
H	-3.73324232281190	-7.10331557305144	2.16733574083464

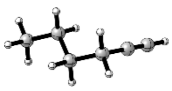	**5d**	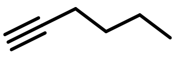

Charge: 0
Multiplicity: 1
[CPCM(CHCl3)M06-2X/6-31G(d)]G_{298K}: -234.35803710
[CPCM(CHCl3)M06-2X/6-31G(d)]H_{298K}: -234.31894809
[CPCM(CHCl3)M06-2X/6-31G(d)]E_{SCF}: -234.47147724
[(CPCM(CHCl3)M06-2X/6-31++G(d, p)]E_{SCF}: -234.49128303

C	1.18109343854613	-1.53351766121520	0.72531867337863
C	1.56361437386936	-2.66399129356499	-0.12865966469516
H	0.68733669369108	-2.99177951766636	-0.69891594376748
H	2.30624584928832	-2.32339936833386	-0.86062471142755
C	2.13839183136376	-3.84292395796012	0.67274799668276
H	1.38376018101723	-4.19448496371144	1.38689212739059
H	2.32934873553787	-4.66862779833523	-0.02319321301512
C	3.42499882251980	-3.49194814800347	1.41721297854391
H	3.22689348736700	-2.65810821698033	2.10139835377120
H	4.17078955702494	-3.13403573075988	0.69572222809513
C	3.98555594884964	-4.68078059151327	2.19368255241012
H	4.21945643657727	-5.51219007065588	1.51989156185305
H	4.90109498277070	-4.41274758573565	2.72899608500056
H	3.25904289429419	-5.04295856289857	2.92896996818768
C	0.87152924203848	-0.61921261659128	1.44995951134760
H	0.59549052524418	0.19903408392558	2.07997849624406

All TSs

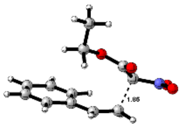	**TS2$_{1a}$**	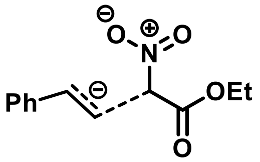

Charge: -1
Multiplicity: 1
[CPCM(CHCl3)M06-2X/6-31G(d)]G_{298K}: -820.76117080
[CPCM(CHCl3)M06-2X/6-31G(d)]H_{298K}: -820.70300006
[CPCM(CHCl3)M06-2X/6-31G(d)]E_{SCF}: -820.96809612
[(CPCM(CHCl3)M06-2X/6-31++G(d, p)]E_{SCF}: -821.02770401

O	-1.66285771781053	0.66839350703723	3.05983827798768
O	-3.07762485593770	2.30938564890317	3.04505683906050
N	-2.46215314983682	1.39454514253973	2.48956778746970
C	-2.58551271774288	1.30656746958267	1.04407978685991
H	-3.48344385368548	1.85593826124828	0.76979318889656
C	-1.28765162891005	2.11338727122816	0.42103511935347
H	-1.42104924629233	3.10845512010816	0.86218267431875
H	-1.56170991402125	2.17776721285414	-0.63895839280513
C	0.03441596285973	1.54890132106579	0.65577583976614
H	0.55167595448478	1.83401490212750	1.56804064529663
C	0.64809426890414	0.59667829833586	-0.17696501189572
C	1.95220020118875	0.07903708152027	0.12471502035510
C	0.07129140313309	0.07866032807473	-1.38435559173360
C	2.59843733544880	-0.83501773287373	-0.68641780788569
H	2.44302246797097	0.43114197444726	1.03075475383627
C	0.73632504950946	-0.83745306081911	-2.18852628043365
H	-0.91646585347179	0.41519661755451	-1.68946717549862

C	2.00884801327250	-1.31785291022542	-1.86486860202205
H	3.58802168571967	-1.18542818469980	-0.39728575027490
H	0.24272164631790	-1.18771585617839	-3.09454670218303
H	2.51866023138500	-2.03768346837987	-2.49711201765748
C	-2.63694298434393	-0.09379278611668	0.50528602323270
O	-2.22511505012627	-1.10509972744789	1.01743023611640
O	-3.20827131031049	-0.05355723485045	-0.71314408985984
C	-3.18185627008284	-1.29753607802046	-1.43567907611279
H	-3.38834561739915	-1.00533038500723	-2.46589916664246
H	-2.16503482123616	-1.69439848929737	-1.37922184496332
C	-4.21878820546937	-2.29545072538456	-0.90902479987395
H	-4.99214203660030	-2.49618590497569	-1.65433691446662
H	-3.75078637404446	-3.24181350691278	-0.62968999124376
H	-4.70732901287295	-1.89255316543803	-0.01843286699722

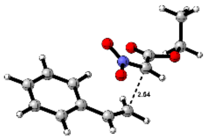	**TS1$_{1a}$**	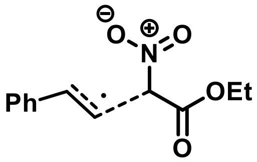

Charge: 0
Multiplicity: 2
[CPCM(CHCl3)M06-2X/6-31G(d)]G_{298K}: -820.62658846
[CPCM(CHCl3)M06-2X/6-31G(d)]H_{298K}: -820.56582393
[CPCM(CHCl3)M06-2X/6-31G(d)]E_{SCF}: -820.83113341
[(CPCM(CHCl3)M06-2X/6-31++G(d, p)]E_{SCF}: -820.87867926

O	-1.17949376532738	-1.34788911834636	1.13452620066337
O	-0.75010009569787	0.40729928404320	2.33191376790861
N	-1.40337634778709	-0.19467634158844	1.48405834192559
C	-2.47458390452931	0.51488351338999	0.88865113060490
H	-2.80456657040200	1.37733525653955	1.44720510922680
C	-1.08204655430616	1.95463249925020	-0.76885084472252
H	-1.71959384657988	2.81975553817705	-0.61689819127968
H	-1.37415686042244	1.25885469971579	-1.55016741808304
C	0.09609109199874	1.84506283016103	-0.11945033168317
H	0.37261956324277	2.61687048992877	0.59675752987295
C	1.05523539364711	0.74880442087177	-0.26466372624385
C	2.31928671528458	0.86718400889402	0.33384584780510
C	0.75386869288969	-0.42292967866747	-0.98123090934161
C	3.26738833454465	-0.14021135375155	0.19823129240953
H	2.55410776991886	1.76236739598265	0.90365091555802
C	1.70267505292797	-1.42929048823747	-1.11451424043855
H	-0.23620595657876	-0.56144254088802	-1.40724980457343
C	2.96268566571077	-1.28959831206171	-0.53139358828389
H	4.24318856025578	-0.03042592152313	0.66074222029212
H	1.45538641677174	-2.32930174012659	-1.66850047763567

H	3.70213866758509	-2.07698895027361	-0.64002493988631
C	-3.26123349381704	-0.05198512635079	-0.21869999871724
O	-2.88800731870066	-0.89328648084319	-1.00558607905758
O	-4.44653476633634	0.56482205347968	-0.26490764594490
C	-5.33532053748031	0.14529431469440	-1.32194840652430
H	-6.04732328335395	0.96544130484520	-1.41575416514983
H	-4.75931911151856	0.05685478183780	-2.24557903851583
C	-6.02454042705259	-1.15759829216200	-0.96138588961895
H	-6.71840823911536	-1.43730880604953	-1.75887743453490
H	-5.29218945326996	-1.95934078117733	-0.84096362539141
H	-6.58913164250205	-1.04729835976393	-0.03187712064032

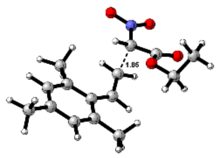	**TS2$_{1k}$**	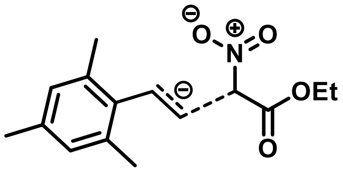

Charge: -1
Multiplicity: 1
[CPCM(CHCl3)M06-2X/6-31G(d)]G_{298K}: -938.56480881
[CPCM(CHCl3)M06-2X/6-31G(d)]H_{298K}: -938.49774563
[CPCM(CHCl3)M06-2X/6-31G(d)]E_{SCF}: -938.85300946
[(CPCM(CHCl3)M06-2X/6-31++G(d, p)]E_{SCF}: -938.91973282

O	4.09763226660794	-1.29726506845033	0.37855146873479
O	2.68000804776594	-2.60997977506162	-0.60975415259439
N	3.01815055561396	-1.49439690873400	-0.18307263425664
C	2.01233884410524	-0.49040964712139	-0.17994719510368
H	1.24744440862415	-0.74365946696660	-0.90641788484841
C	1.21771338544112	-0.68858547120994	1.40669211668388
H	2.03964375088766	-0.38970279280795	2.06407531285482
H	1.13799190625794	-1.77499216751116	1.37702728844665
C	0.03840315804303	0.10330521385553	1.54972839083072
H	0.23304688268362	1.10060876253761	1.93176992989529
C	-1.19713280017840	-0.05061387708915	0.83960378965522
C	-2.05890389085466	1.09014670475909	0.69704133038510
C	-1.66997427377711	-1.26931092608688	0.25630262202079
C	-3.25340378602446	1.00675563260734	-0.00386190813127
C	-2.87732650258878	-1.29556533306639	-0.44539936438464
C	-3.69076775810448	-0.17722410345992	-0.60817561012287
H	-3.86875072067689	1.90305491692657	-0.08950510951364
H	-3.20316910255027	-2.24652136549628	-0.86820254380789
C	2.45466347793773	0.92223427624632	-0.24992154437969
O	3.42754799594956	1.42340247151468	0.26956091489682
O	1.51741845500003	1.63268902252346	-0.90396443225692
C	1.71387203995701	3.05423760361486	-0.94484110009982

H	0.71764935511517	3.46515029312286	-1.11809229265505
H	2.07827166840814	3.39111487191209	0.02851310249147
C	-1.66557310044422	2.41344063872195	1.30215579469212
H	-1.53831117842639	2.34201575357502	2.38990664758188
H	-0.70494033303553	2.76506993420755	0.90281349083168
H	-2.42323943174105	3.17544995652947	1.09770617800317
C	-4.97700001644170	-0.23209961898058	-1.39360407907501
H	-5.79143912070512	0.28226003078058	-0.87126759379225
H	-4.87745311913767	0.24452047805700	-2.37748100094680
H	-5.28903385609909	-1.26749765876789	-1.56411183648138
C	-0.95310554256875	-2.58223840939988	0.45632704972468
H	0.00717021983257	-2.64858970712404	-0.07252721640784
H	-0.73979625429679	-2.75097121217462	1.51947696955656
H	-1.57609592312148	-3.40972878526744	0.10257504762546
C	2.67298163897625	3.43818394017900	-2.05695786460033
H	2.76222256899179	4.52703134494122	-2.11288754697271
H	3.66281946729560	3.01553392611615	-1.86811168027478
H	2.30641372727844	3.06947003204772	-3.01881217420511

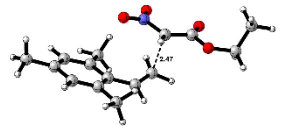	**TS1$_{1k}$**	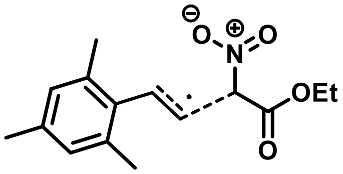

Charge: 0
Multiplicity: 2
[CPCM(CHCl3)M06-2X/6-31G(d)]G_{298K}: -938.43159270
[CPCM(CHCl3)M06-2X/6-31G(d)]H_{298K}: -938.36322095
[CPCM(CHCl3)M06-2X/6-31G(d)]E_{SCF}: -938.71670763
[(CPCM(CHCl3)M06-2X/6-31++G(d, p)]E_{SCF}: -938.77147593

O	-2.94832779347705	-0.46042485649644	-0.71285079285443
O	-1.30487741319941	-1.17358310327197	0.51223005995643
N	-2.19447836165880	-0.36218360461896	0.25076521439910
C	-2.28073563880004	0.74937684425187	1.11622870625713
H	-1.56800733560200	0.73847646251771	1.92626527833562
C	-1.07901119963121	2.20597754260902	-0.68983879549037
H	-1.55066377717839	3.15320642408688	-0.44192474438852
H	-1.61181376579952	1.57275706122803	-1.38909903176248
C	0.14198244905538	1.92774439534422	-0.17551745509803
H	0.53193948237048	2.67330081101458	0.51286489930883
C	0.99001240424270	0.74524166902725	-0.33866714290830
C	1.93688675165073	0.48688076383795	0.68732455536839
C	0.92687143669807	-0.12856147540789	-1.45169991284421
C	2.77073619515678	-0.62155598614102	0.59308994302966
C	1.79696895306481	-1.21702069503785	-1.50907567293671
C	2.71593138258242	-1.49129554210534	-0.49800312612266

H	3.48686473109393	-0.81295559975413	1.38936811764810
H	1.75335063673842	-1.87230527065700	-2.37642544189453
C	-3.40447579464860	1.69666557445272	1.09424238502557
O	-4.30545492043733	1.74478446790350	0.28913010888179
O	-3.25068680034544	2.54288340716508	2.12260313836025
C	-4.25323064203479	3.57164654587542	2.25084869857648
H	-3.77930013209099	4.33198101227759	2.87242581779057
H	-4.45473626208228	3.98647308775524	1.26051448198215
C	-5.51303301758202	3.02564082911183	2.89590886515143
H	-6.23145025066189	3.83705220850062	3.04238614474648
H	-5.97210247412768	2.26582099481561	2.25925514165282
H	-5.28302002874818	2.58559403416955	3.86989123640722
C	2.05185329822634	1.37385903161801	1.90552916131954
H	2.36762654368812	2.38956243117837	1.64210453303868
H	1.09775866101896	1.45680367887632	2.43669761492862
H	2.78948043198861	0.96646374903015	2.60021401909214
C	-0.02769690932188	0.06528082750762	-2.60372100570592
H	-1.03933628049272	-0.26350644647384	-2.33923444624529
H	-0.08628311807623	1.11280259531074	-2.91389971771862
H	0.29651002210631	-0.53084975017850	-3.46013359680071
C	3.60934485567044	-2.70124735696038	-0.56818714411849
H	3.13813087526091	-3.55523999808535	-0.06832966086221
H	3.80259824607070	-2.99045207177255	-1.60469407387269
H	4.56600126931239	-2.51619342250472	-0.07198420963282

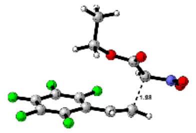	**TS2$_{1j}$**	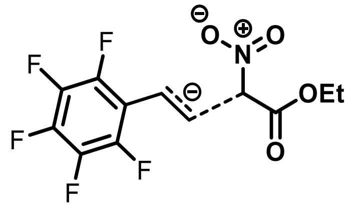

Charge: -1
Multiplicity: 1
[CPCM(CHCl3)M06-2X/6-31G(d)]G_{298K}: -1316.81296404
[CPCM(CHCl3)M06-2X/6-31G(d)]H_{298K}: -1316.74433973
[CPCM(CHCl3)M06-2X/6-31G(d)]E_{SCF}: -1316.97328483
[(CPCM(CHCl3)M06-2X/6-31++G(d, p)]E_{SCF}: -1317.06047954

O	4.66502539982592	-0.08938114607848	0.66013349547188
O	4.29213662259138	-2.03927478889040	-0.20841624203297
N	3.93868615263258	-0.88164600103037	0.05614187366457
C	2.59474992660479	-0.54775626694488	-0.22761853962771
H	2.17600072007618	-1.23655333070332	-0.94834155043517
C	1.68180149544258	-0.96763753932273	1.39324291144183
H	2.51516741944807	-0.76558926166314	2.06752107400034
H	1.57365485370269	-2.02499993343937	1.16337482166002
C	0.52563105641715	-0.19964774227014	1.65959862227032

H	0.64891530853611	0.70776459527565	2.24074870052718
C	-0.73090208251769	-0.33851254761892	1.00600567111831
C	-1.79891999977490	0.54131252092749	1.30067533055404
C	-1.04788008225421	-1.27971704842344	0.00149256256635
C	-3.03766823760943	0.48616485221971	0.69240567549629
C	-2.28198818455760	-1.35052669331100	-0.62010532332456
C	-3.30061597906427	-0.47010432807122	-0.28114985374842
C	2.21703540488703	0.85889748054256	-0.42460710604098
O	2.69805206267124	1.85195624218063	0.07459853383131
O	1.08548599623242	0.87372287581506	-1.15874540471318
C	0.32579607122142	2.08911511045286	-1.13124747302791
H	-0.69863447315986	1.77535735537714	-1.34740696656994
H	0.36281598145632	2.50157745099603	-0.11892815549777
F	-2.49613569488930	-2.27330737308560	-1.56732155673646
F	-0.10963418283373	-2.15207831151850	-0.42329762972876
F	-1.60473639521570	1.50812459386266	2.21966174748516
F	-3.99692055658028	1.35335767793987	1.04187715301917
F	-4.50466442528031	-0.54355069546859	-0.86848362738827
C	0.83467052558903	3.08200509635786	-2.16011019695829
H	0.20331221708920	3.97545305216842	-2.16291296910289
H	1.85982884732444	3.37760880040320	-1.92577182142922
H	0.81286523198867	2.63865030332094	-3.15971775674425

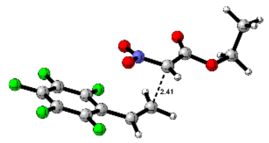	**TS1$_{1j}$**	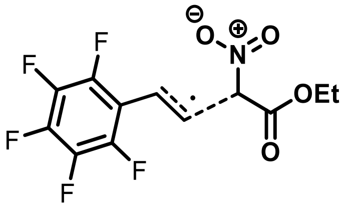

Charge: 0
Multiplicity: 2
[CPCM(CHCl3)M06-2X/6-31G(d)]G298K: -1316.66329474
[CPCM(CHCl3)M06-2X/6-31G(d)]H298K: -1316.59306598
[CPCM(CHCl3)M06-2X/6-31G(d)]ESCF: -1316.82236468
[(CPCM(CHCl3)M06-2X/6-31++G(d, p)]ESCF: -1316.89501175

O	-0.92796804467463	-1.60128285753771	0.08781566361873
O	-0.56712555149609	-0.40885039583013	1.86055738377484
N	-1.23435861920143	-0.71486363460272	0.87490813709093
C	-2.37226135485937	0.09058156481003	0.62084106124081
H	-2.53005197396694	0.87156574800564	1.34944918093518
C	-1.18378513444054	1.45333469698070	-1.07662399159247
H	-1.95580212853897	2.21353939111544	-1.01218486941429
H	-1.33080421231625	0.65129487005874	-1.79080320590957
C	-0.02607838920917	1.62709723682173	-0.39526682082944
H	0.05681008046476	2.47763248088714	0.27489413921544
C	1.12232319723099	0.73030736951067	-0.38480198705167

C	2.09645471735504	0.89337517762375	0.61159544230997
C	1.32902357085245	-0.32099939854722	-1.28862647082381
C	3.20704413438600	0.07438145925116	0.70921552157722
C	2.43496040162236	-1.15127908403668	-1.20975407005056
C	3.37623495547769	-0.95537229587526	-0.20792509648610
C	-3.44755592470170	-0.33093399995013	-0.29325419207608
O	-3.48589675503652	-1.34285603817673	-0.95379551194683
O	-4.38943624518672	0.62111142769461	-0.29071096558418
C	-5.53299917235917	0.38101774965786	-1.13752902511304
H	-5.96958454213549	1.36923590319432	-1.28401421962028
H	-5.18077696642814	-0.00666826513468	-2.09587211155928
C	-6.50990346377072	-0.57031178564394	-0.47332562512741
H	-7.39672394805164	-0.68029272107301	-1.10374068279331
H	-6.05742472176421	-1.55513699756603	-0.33578109195792
H	-6.82170532848163	-0.17998280897371	0.49897645571627
F	1.95570927182838	1.86214892904345	1.51496985876774
F	4.10522611694108	0.26117735996569	1.67237802359879
F	4.43532683629200	-1.74793132866934	-0.12864003667318
F	2.60143128660247	-2.13410470416358	-2.09025561868343
F	0.46544690756620	-0.55065204884004	-2.27475027455306

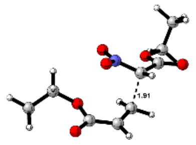	**$TS2_{1aa}$**	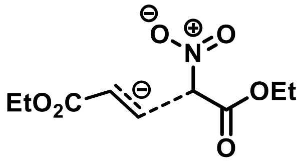

Charge: -1
Multiplicity: 1
[CPCM(CHCl3)M06-2X/6-31G(d)]G_{298K}: -856.92120880
[CPCM(CHCl3)M06-2X/6-31G(d)]H_{298K}: -856.86007279
[CPCM(CHCl3)M06-2X/6-31G(d)]E_{SCF}: -857.11706762
[(CPCM(CHCl3)M06-2X/6-31++G(d, p)]E_{SCF}: -857.17712667

C	1.69172864231007	-2.36194621334743	-0.28112209145195
C	1.25499561875611	-1.06562700584088	0.01749563493879
C	-0.58289397262917	-0.72008767440875	-0.53345303139870
H	1.51897306354388	-3.17741284660400	0.41262576246258
H	1.67344422295221	-0.24042305092258	-0.55767861558313
H	1.08560067877780	-0.82381725828577	1.06660028622559
H	-1.11481926762954	-1.52152933368492	-0.04020657749325
N	-0.50425059165975	-0.95795466117767	-1.92125121651107
O	-0.62447285457260	-2.13970274492705	-2.26900547321434
O	-0.21733975410828	-0.05629980030368	-2.71585771611616
C	-0.88745831308845	0.59915717178805	0.03169985196116
O	-1.39742410054340	0.70542634780564	1.13647334439566
O	-0.44547727446655	1.65373998330783	-0.66805879086921
C	-0.65582997422274	2.93917782271378	-0.06458461449141

H	0.05326264909891	3.59554406094305	-0.57192324797272
H	-0.39989702134559	2.88268525429401	0.99580453479731
C	-2.08410320817045	3.41475259749600	-0.26263596430726
H	-2.78128047804585	2.75113044564651	0.25407876084220
H	-2.19925340911437	4.42501661652771	0.14110590773629
H	-2.33461931402660	3.43432275663131	-1.32682843666341
C	2.16710358856812	-2.73547312378307	-1.57317334497051
O	2.44916820994196	-3.87155745408903	-1.95644890083099
O	2.32217500306663	-1.65702233539531	-2.41013800537856
C	2.43656422086221	-1.95011907503506	-3.79564388874275
H	1.82098847734824	-2.82221929934648	-4.03305132955059
H	2.01706532617295	-1.07811515344435	-4.30524210252405
C	3.88238479163135	-2.18444965059347	-4.20079923832884
H	4.27024786118441	-3.06785710059710	-3.68852412259695
H	3.95906914096633	-2.33905519212956	-5.28194164682588
H	4.49832354844211	-1.32121308323770	-3.93069401753773

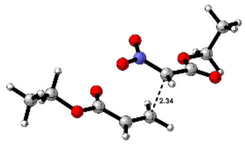	**$TS1_{1aa}$**	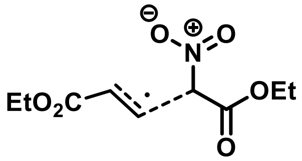

Charge: 0
Multiplicity: 2
[CPCM(CHCl3)M06-2X/6-31G(d)]G_{298K}: -856.76008044
[CPCM(CHCl3)M06-2X/6-31G(d)]H_{298K}: -856.69779902
[CPCM(CHCl3)M06-2X/6-31G(d)]E_{SCF}: -856.95529220
[(CPCM(CHCl3)M06-2X/6-31++G(d, p)]E_{SCF}: -857.00323045

C	1.70860630825174	1.60388905787544	-0.17356186499878
C	0.42476894166458	1.71973711504873	-0.58313552623503
C	-0.63764007078378	0.39897772819203	1.04837340018363
H	2.16551513561192	2.29421953660291	0.52743451130593
H	0.04127445698228	1.04393771105462	-1.34422639355295
H	-0.17636056450197	2.58211441873051	-0.31162548247705
H	-0.40579494240400	1.02886256225261	1.89479741985062
N	0.20263849361838	-0.75272647505234	0.96271672197797
O	1.24831714455878	-0.69869388479085	1.60790633560635
O	-0.12077340868795	-1.69356855340277	0.25517971213468
C	-1.99483281382750	0.50627178988161	0.46988193793826
O	-2.77473152788153	1.31046317606310	0.93853630572669
O	-2.22179303505450	-0.25442968690080	-0.59051384065051
C	-3.54401362576361	-0.15884698529301	-1.16898900965269
H	-3.41978265365867	-0.56119069368937	-2.17410714291035
H	-3.82367326035215	0.89505377896811	-1.22760997098266
C	2.49926816053063	0.42708278516780	-0.61321641989526
O	2.09177610026579	-0.40235994034164	-1.40025431901615

O 3.69192982145948 0.37809706012447 -0.02193127436330
C 4.46331678614344 -0.80745026753169 -0.29238648978451
H 3.84147949420414 -1.67722015464961 -0.06222158977715
H 4.70562160109971 -0.83141686461954 -1.35869773600881
C -4.54392903809575 -0.96400775186047 -0.36162053369233
H -4.66290724772278 -0.54363513746050 0.63981680592285
H -5.51554765457980 -0.94460817847946 -0.86312440857338
H -4.21637127774328 -2.00340850462584 -0.27769409541248
C 5.70111223900698 -0.74740342224568 0.57335324701713
H 5.42782397525987 -0.72285703442556 1.63140000458552
H 6.31641255106905 -1.63259930042709 0.39286024853535
H 6.29427191133045 0.14082311583431 0.34100944719842

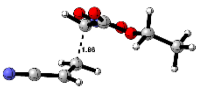	**TS2$_{1ab}$**	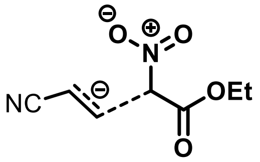

Charge: -1
Multiplicity: 1
[CPCM(CHCl3)M06-2X/6-31G(d)]G_{298K}: -682.11921788
[CPCM(CHCl3)M06-2X/6-31G(d)]H_{298K}: -682.06546056
[CPCM(CHCl3)M06-2X/6-31G(d)]E_{SCF}: -682.24467440
[(CPCM(CHCl3)M06-2X/6-31++G(d, p)]ESCF: -682.29588672

C 1.45645325077289 -1.71986971654301 0.64274225422767
C 1.14331062548329 -1.41968957517176 -0.68909120878454
C -0.65612976780026 -0.75025085270720 -0.97754302070724
H 1.77011275890408 -0.94478287438546 1.33219524902564
H 1.03241880389018 -2.25713804072920 -1.37890990718906
H 1.62978351995205 -0.55449162256889 -1.14150196291203
H -1.28024516813101 -1.56507293460975 -0.63539103244526
N -0.69218832645904 -0.65245236656816 -2.38531581179531
O -1.19988833328590 -1.59924912910515 -3.00358994295355
O -0.10696920216538 0.26960328254956 -2.95954852734387
C 1.14614106337274 -2.97602710235020 1.18858446265072
N 0.90943856437979 -4.02967841897892 1.63919722419620
C -0.70849578719361 0.42310244618965 -0.10293969201788
O -0.97684802502053 0.29907356760156 1.07929076137369
O -0.34631551435305 1.59498520012700 -0.64491215838130
C -0.33790438560895 2.70864032810523 0.25827162909945
H -1.33116458185214 2.81577952364034 0.70410408975435
H 0.37264125495560 2.50764738067643 1.06550664185432
C 0.05413529049351 3.92669368769041 -0.55063294147286
H -0.66368698190559 4.09671277590857 -1.35772156427344
H 0.07328297061021 4.81139210979915 0.09142367886649
H 1.04605431096109 3.79201906142979 -0.99017020077217

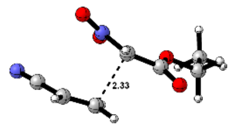	**TS1$_{1ab}$**	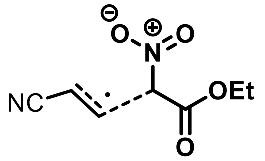

Charge: 0
Multiplicity: 2
[CPCM(CHCl3)M06-2X/6-31G(d)]G_{298K}: -681.95362926
[CPCM(CHCl3)M06-2X/6-31G(d)]H_{298K}: -681.89844342
[CPCM(CHCl3)M06-2X/6-31G(d)]E_{SCF}: -682.07757301
[(CPCM(CHCl3)M06-2X/6-31++G(d, p)]E_{SCF}: -682.11528508

C	1.34405583786580	-2.35926105114018	-0.48011298360731
C	1.15530290781582	-1.10512533270877	0.00282610372309
C	-1.01360208333322	-0.60439540897492	-0.68148589807220
H	1.07734800591746	-3.23788557896499	0.09846471454358
H	1.50345354017306	-0.24378492543459	-0.56156288000724
H	0.85344602887272	-0.94875651836226	1.03366152893243
H	-1.52985114221937	-1.37108171672549	-0.12295264594407
N	-0.77777101423516	-0.96105205391881	-2.05273875723803
O	-1.21120981717659	-2.05418209562120	-2.38878417234702
O	-0.10517261780750	-0.21920488819148	-2.74610721927061
C	1.85183862164873	-2.58636051520029	-1.79502481257195
N	2.27951328330084	-2.77598592232975	-2.85535020668797
C	-1.05633283419696	0.77368853464316	-0.15307379141801
O	-1.22936853281899	0.94579283638673	1.03459005431196
O	-0.88065138566065	1.73495109906310	-1.04881484326803
C	-0.93135016497811	3.07666704938320	-0.52562388092887
H	-1.90303295001460	3.22409866763279	-0.04573027651224
H	-0.15931443551713	3.18055394878461	0.24216361636262
C	-0.71550725384467	4.01324430420360	-1.69289287231288
H	-0.75370820524195	5.04939144599772	-1.34689679694598
H	0.25842536892578	3.83452173877426	-2.15441017046957
H	-1.48940454747530	3.86841291270357	-2.45023983027165

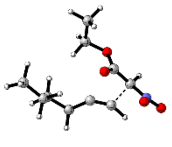	**TS2$_{5d}$**	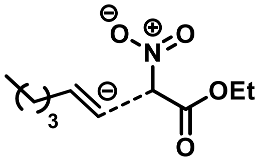

Charge: -1
Multiplicity: 1
[CPCM(CHCl3)M06-2X/6-31G(d)]G_{298K}: -745.70349340
[CPCM(CHCl3)M06-2X/6-31G(d)]H_{298K}: -745.64302224
[CPCM(CHCl3)M06-2X/6-31G(d)]E_{SCF}: -745.91666709
[(CPCM(CHCl3)M06-2X/6-31++G(d, p)]E_{SCF}: -745.97647700

O	4.01363610843478	2.98706404823878	-0.00893246846652
O	5.63970593999200	1.55156128100134	0.01714127400649
N	4.42705574467964	1.82459982297411	-0.01291133507376
C	3.55897719007454	0.71850921649483	0.12086124990628
H	3.99932422980456	-0.15198762377628	-0.35008916465796
C	3.68191158986635	0.31988133863604	1.91234306097054
C	2.61657449231928	0.07979604202644	2.55864723441296
C	2.11851747397864	0.89895093529159	-0.12562992443008
O	1.44608527090777	1.89156389496524	0.03736570244740
O	1.59149771059500	-0.30107473851172	-0.44701803791290
C	0.16093232549204	-0.37763463441728	-0.43507057056968
H	-0.05884052302297	-1.43750279142773	-0.29091714183538
H	-0.21386273053340	0.18444076251990	0.42483626699336
C	-0.43380080103083	0.13669990655557	-1.73380775323507
H	-1.51903949162047	-0.00328168732176	-1.73024692241589
H	-0.21823687789367	1.20075545794404	-1.85319617500321
H	-0.01666016142900	-0.40787260524059	-2.58576974826864
H	4.77163950131210	0.40536907877577	1.98745250043108
C	2.17033017462860	-0.30389519128870	3.92516409894092
H	2.47566635041587	0.46814728136929	4.65024156264667
H	2.67008804676770	-1.22956134202710	4.24891776844809
C	0.64918923457573	-0.49539365161223	4.04240992476033
H	0.33967502650964	-1.26701599388436	3.32326298761022
H	0.38822720533923	-0.86633879835099	5.04415739324320
C	-0.12189626809056	0.78869220410853	3.75167214910487
H	0.24351503029355	1.18714698968239	2.79647597450562
H	0.12310666787648	1.53830803040243	4.51710221748880
C	-1.63278519742559	0.57852212300471	3.69710197013260
H	-2.00996648792107	0.17438456137245	4.64372624831897
H	-2.16535348729721	1.51321521012795	3.49487572015704
H	-1.89419598759881	-0.13479427763266	2.90698293734349

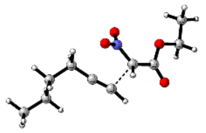	**TS1$_{5d}$**	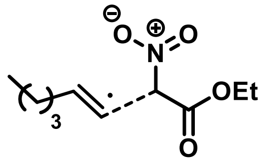

Charge: 0
Multiplicity: 2
[CPCM(CHCl3)M06-2X/6-31G(d)]G_{298K}: -745.58252257
[CPCM(CHCl3)M06-2X/6-31G(d)]H_{298K}: -745.51996286
[CPCM(CHCl3)M06-2X/6-31G(d)]E_{SCF}: -745.79469571
[(CPCM(CHCl3)M06-2X/6-31++G(d, p)]E_{SCF}: -745.84118006

C	1.16635697316899	-1.53981858642124	0.75335489284392
C	-1.27682180643568	-0.14723847425787	0.44412390213276
H	-1.86317507569091	-0.47588115758638	1.29053005600654

N	-1.26234125408220	-1.08358303418185	-0.62721694442030
O	-1.86079197075503	-2.14004207853519	-0.43366514024624
O	-0.59196753494694	-0.85199944255352	-1.62379122567673
C	-1.08482673457134	1.30592202597352	0.27098264568014
O	-1.02179031075481	2.02209038114830	1.25100439181705
O	-1.00692120601693	1.72749302796722	-0.98207268014841
C	-0.83661564399971	3.14976996937144	-1.15427474932414
H	-1.66209730567619	3.66193258212272	-0.65309043339871
H	0.09697338549618	3.44622702965890	-0.66854035275733
C	-0.81871177618179	3.41231312771145	-2.64359730291623
H	-0.69110204424121	4.48244306050688	-2.82516156279872
H	0.00586123634192	2.87570912116304	-3.11949526976933
H	-1.75788802133929	3.08988379860899	-3.10065754319435
C	1.54927213555851	-2.65146367136044	-0.11155737755356
H	0.65102279098412	-2.97440560880994	-0.65782142472650
H	2.26537520726741	-2.29016939992729	-0.85870050344265
C	2.14573266633810	-3.83198392417770	0.67309463743695
H	1.39941416156849	-4.20665405005719	1.38311608845991
H	2.34578078190678	-4.63988565653675	-0.03992124839753
C	3.42981639012745	-3.46517889794286	1.41477036629206
H	3.21580739623447	-2.66254684818739	2.13211018335569
H	4.15561879716182	-3.05908644362346	0.69883572394814
C	4.03448352829608	-4.66309717846145	2.14254229107777
H	4.28870965914893	-5.45923239620782	1.43461507677228
H	4.94530007495486	-4.38468186437523	2.68019622052287
H	3.32608151428060	-5.07582671709585	2.86856775868245
C	0.69510711866519	-0.61311650550704	1.40092596800630
H	0.58179828719207	0.15331236157408	2.14411881573587

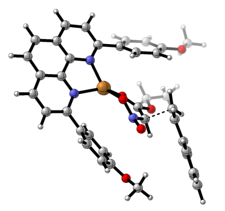	**TS3$_{1a}$**	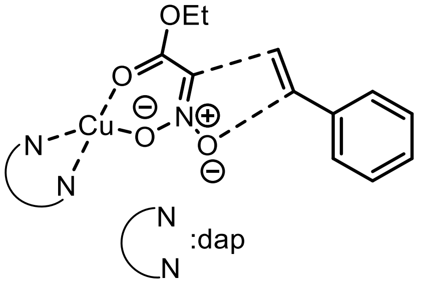

Charge: 1
Multiplicity: 2
[CPCM(CHCl3)M06-2X/6-31G(d)]G_{298K}: -3722.70489740
[CPCM(CHCl3)M06-2X/6-31G(d)]H_{298K}: -3722.59681150
[CPCM(CHCl3)M06-2X/6-31G(d)]E_{SCF}: -3723.29791239
[(CPCM(CHCl3)M06-2X/6-31++G(d, p)]E_{SCF}: -3723.41326425

O	-2.05637343118329	0.35662132905425	2.66672922885382
O	-1.95893897344491	0.27725220697593	0.44907691555683
N	-2.13703839651688	0.97800041408467	1.47446098821158

C	-1.67121389336922	2.25218099476057	1.33638369390807
H	-1.92709959132815	2.67942204859474	0.36962450551698
C	0.20154614854330	1.86473227170019	0.91202958300854
H	0.61674912418622	1.64913092250347	1.89412135781843
H	0.44442568491335	2.85771598912535	0.54122241067326
C	0.09562440906023	0.79473198566520	0.01232435088897
H	0.38779968406404	-0.19891297053981	0.33463665495821
C	-0.13810349538878	0.97606148775849	-1.42215959806563
C	-0.15143464679769	-0.14504076586069	-2.26841825635241
C	-0.42682132749886	2.22962529238569	-1.98667133350836
C	-0.43485670674482	-0.02092906254263	-3.62439785987181
H	0.05822033259977	-1.12448117026408	-1.84512665861123
C	-0.70794391199727	2.35440694831243	-3.34481815523505
H	-0.41985537421006	3.12411824762743	-1.36962048375244
C	-0.71609886463148	1.23189485965436	-4.17109424000136
H	-0.43644411210038	-0.90428550996666	-4.25622033958466
H	-0.92244538956113	3.33527565548420	-3.75944528858639
H	-0.93570152837272	1.33210472807145	-5.22952387869269
C	-1.77549767776424	3.19875816064492	2.45968110494731
O	-2.30558627490005	2.98494619722949	3.55977059766505
O	-1.22248277039109	4.35071620755536	2.18143343243933
C	-1.25572476641939	5.38306044889397	3.20305420959794
H	-2.23864234576982	5.85812625942528	3.14770588310647
H	-1.14298879552296	4.90842836860178	4.17934783344244
C	-0.12795140514315	6.34261772837306	2.90237273395209
H	-0.13095749603131	7.14159219187011	3.64940122203205
H	0.83407528607657	5.82379453529138	2.94840802028630
H	-0.24529720665248	6.79159811932559	1.91316346887276
Cu	-2.87422855068364	1.19124495253435	4.15634174776047
N	-4.53372662888054	0.13107936824426	4.54232747096348
N	-2.78492679514681	1.29914325668916	6.19985919862355
C	-4.67348104734618	-0.13332425603679	5.86021703686753
C	-5.40287848747446	-0.34972122377850	3.65577526583665
C	-3.76195642856774	0.52993589170243	6.75422156446770
C	-1.93588750445647	1.95203356050836	6.99542944571498
C	-5.68316931253656	-0.96627162872187	6.35976470799721
C	-6.40922932335300	-1.25332942443051	4.06850938652009
C	-5.28456195050582	0.18520122014518	2.28625202152833
C	-3.93174613724051	0.37589186212797	8.13678058036951
C	-2.07412857581414	1.88835720975681	8.40411739821253
C	-0.82173784784000	2.70464047202869	6.38335799544678
C	-5.79978513088284	-1.14580389595481	7.77864526866619
C	-6.54185037932750	-1.56051369462971	5.40359806664628
H	-7.09538194980034	-1.65242337585511	3.32990773967639
C	-5.17140413985937	1.57596543280704	2.12414082360902
C	-5.23330032751332	-0.63549213769430	1.15946715954964

C	-3.05314550956672	1.10731426857068	8.96807218674857
C	-4.96537777294441	-0.49016763310988	8.63092508566834
H	-1.37063107598354	2.43229272324376	9.02399090895135
C	-0.45888319288180	3.96526576106995	6.86442591998626
C	-0.07462254295613	2.14519848756512	5.33501709645554
H	-6.57846181708148	-1.80111776227608	8.15635467482982
H	-7.32905578659938	-2.22985200608266	5.73861447929879
C	-4.96179031666808	2.12395310854816	0.87114535690276
H	-5.27118053961687	2.22974085189788	2.98870519050886
C	-4.98374615761621	-0.09638115147953	-0.09894822738340
H	-5.33123848821772	-1.71199134239539	1.26860735909953
H	-3.14926754880374	1.03160964238799	10.04737759886855
H	-5.06523633908741	-0.60795344110383	9.70538600276478
C	0.60366243622843	4.67328221089288	6.30629982062217
H	-1.02295922184168	4.41899021162152	7.67470515572751
C	0.98484307417385	2.83493236379067	4.77268370219607
H	-0.29418695283829	1.13858595761548	4.98569049803283
C	-4.80720207326419	1.28438452933062	-0.24059476454396
H	-4.86428695816019	3.19651586243650	0.73332823453829
H	-4.88368482392317	-0.75877479175878	-0.95031103506272
C	1.33053627281158	4.10745833667094	5.25287488123219
H	0.85111640468070	5.65404742684577	6.69492761672697
H	1.58446928242576	2.39802561446648	3.98107176427079
O	-4.47719265777135	1.89608355371448	-1.39833066288183
O	2.37812657429607	4.70122951547479	4.64307314200886
C	-4.00678125323036	1.08132895143839	-2.46548038191990
C	2.81825869761355	5.95659205690092	5.14256764024011
H	-4.79502032188389	0.41690888626018	-2.83424234337966
H	-3.71234704180939	1.76912522883681	-3.25701358915813
H	-3.13629941745621	0.49720391465285	-2.14408431407931
H	3.10894742181735	5.87684956449303	6.19520050772308
H	3.68368830476689	6.23005357607319	4.54081973227085
H	2.03869073091500	6.71795481416797	5.03152101080409

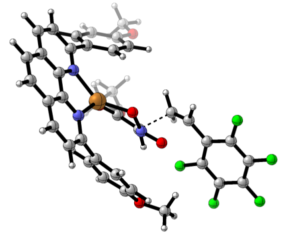	**TS3$_{1j}$**	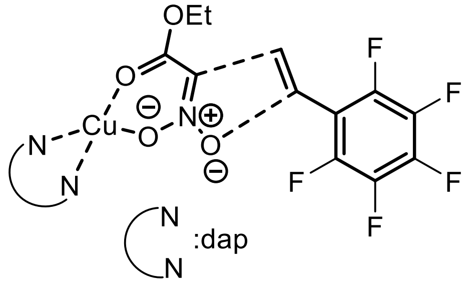

Charge: 1
Multiplicity: 2
[CPCM(CHCl3)M06-2X/6-31G(d)]G_{298K}: -4218.74644500
[CPCM(CHCl3)M06-2X/6-31G(d)]H_{298K}: -4218.62798959
[CPCM(CHCl3)M06-2X/6-31G(d)]E_{SCF}: -4219.29251599
[(CPCM(CHCl3)M06-2X/6-31++G(d, p)]E_{SCF}: -4219.43087510

O	-0.84049482443306	-1.59044318139881	-0.01143172879315
O	-0.64230290770213	-0.35553558812498	1.75859590272286
N	-1.23819063936010	-0.70705229743996	0.74599851423498
C	-2.37248114020217	0.07099593697260	0.37678500826888
H	-2.58157814147936	0.80891037747885	1.14073948058031
C	-1.20448793976471	1.37869981789087	-0.99162793673479
H	-1.98192205510820	2.13636574073708	-0.98441972552327
H	-1.27027758344089	0.62790320624300	-1.76998328912572
C	-0.03312519409562	1.61840659430479	-0.32444211585797
H	0.00659589911495	2.43465069162166	0.38993170662405
C	1.13764169881786	0.76056271306207	-0.34351936933922
C	2.03255865142142	0.80495912306193	0.73691384218953
C	1.44430853550092	-0.14663658588951	-1.36826283400820
C	3.15761618174265	0.00263039968576	0.80387012627779
C	2.56393752675261	-0.95939646489386	-1.31992604992340
C	3.42416071766423	-0.88566867518579	-0.23153360376864
F	1.78723146039719	1.62765553963006	1.75335791177236
F	3.97808474086728	0.06799351631602	1.84717184189859
F	4.49747767761278	-1.66193350830290	-0.18140071000338
F	2.82638184066651	-1.80040677305146	-2.31529654402255
F	0.66660800887649	-0.24312418375069	-2.44499931412310
C	-3.46179039823045	-0.41396078182536	-0.44192016175167
C	-3.42591582041036	-1.55971625833808	-1.26112396861187
C	-4.63123481918965	0.37439824633483	-0.43426003184111
C	-4.53856579130358	-1.90244758942955	-2.01892967060062
H	-2.53652883784260	-2.17391857309481	-1.28391262519811
C	-5.72982265386805	0.02997845807214	-1.20575912693322
H	-4.66600222472683	1.26366302973619	0.19020751169406
C	-5.68935046598356	-1.11575796488361	-2.00050229521117
H	-4.50515987857245	-2.79642496721079	-2.63351297264258
H	-6.62073475340650	0.64922190787153	-1.18469961388470
H	-6.54958453031451	-1.39380567619920	-2.60074510836491

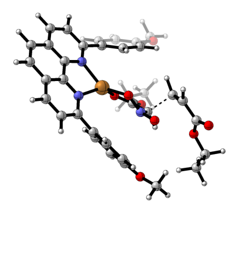	**TS3$_{1aa}$**	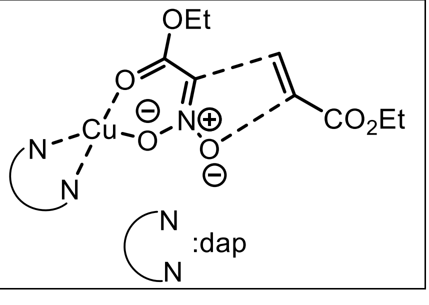

Charge: 1
Multiplicity: 2
[CPCM(CHCl3)M06-2X/6-31G(d)]G_{298K}: -3758.85022547
[CPCM(CHCl3)M06-2X/6-31G(d)]H_{298K}: -3758.73984002
[CPCM(CHCl3)M06-2X/6-31G(d)]E_{SCF}: -3759.43283264

[(CPCM(CHCl3)M06-2X/6-31++G(d, p)]E$_{SCF}$: -3759.54917831

O	-2.15424497023359	0.42402690080340	2.55224875880161
O	-1.96752424396173	0.34518875730834	0.36121760788558
N	-2.11683382953195	1.05201763518965	1.37527343842076
C	-1.51218740019936	2.28272079982332	1.27258277334141
H	-1.67522995422223	2.72501214068594	0.29287506855462
C	0.27783630274876	1.76404365974271	1.02288602903343
H	0.64917155399340	1.62706437707768	2.03739413814557
H	0.60388260886340	2.69051262423756	0.55545260813788
C	0.21987190920826	0.60847047913847	0.22020353467097
H	0.44588080333951	-0.37226625940399	0.61874226758343
C	-1.63225944744006	3.24602831172060	2.38877597082734
O	-2.22721402659120	3.06788866506791	3.46101747423218
O	-1.00804913076780	4.36312212815725	2.13234891911763
C	-1.05693370352136	5.41173035892732	3.14005261487559
H	-2.03719972865586	5.88810963731401	3.05794609119536
H	-0.96648115336968	4.94784514602255	4.12360750600445
C	0.07950661009759	6.36340750874324	2.84858018810373
H	0.06198143067968	7.17151456785238	3.58574880179115
H	1.04046914177326	5.84634658242627	2.92218749551762
H	-0.01859792007620	6.80067221230589	1.85213469468278
Cu	-2.87046652059039	1.30598081312191	4.06955684815543
N	-4.52530135728594	0.23789426387585	4.42693271956033
N	-2.75855001458397	1.34449668484330	6.11197298997788
C	-4.64138698352480	-0.08971012347818	5.73288691951019
C	-5.39164759790390	-0.22713787204462	3.52918459592919
C	-3.71858926978696	0.53534434690055	6.64246595123756
C	-1.90139630579333	1.95918906986552	6.92840926375401
C	-5.62745047232336	-0.96508015705315	6.20684732441124
C	-6.38057768397157	-1.16149614750140	3.91582471436502
C	-5.28429028997389	0.33063664470837	2.16934491398521
C	-3.85586741697030	0.29867012455559	8.01727297515933
C	-2.00872812481598	1.81059612091616	8.33374747720618
C	-0.80329486391007	2.76746468451819	6.35903233349227
C	-5.71254586520634	-1.22410805376674	7.61507825007527
C	-6.49188833692042	-1.52790240682774	5.23707068675870
H	-7.06708891005542	-1.54280503626209	3.16824734231562
C	-5.11857554997335	1.71722057832917	2.01531473034551
C	-5.29387096147685	-0.48097255677746	1.03542077445278
C	-2.96586643960175	0.98678094692627	8.87183543251302
C	-4.86709130137910	-0.60713843289995	8.48457948630143
H	-1.29523060570678	2.32368511269485	8.96820270732962
C	-0.48317726126335	4.00612418845937	6.92005486911597
C	-0.01410118430596	2.28300349823835	5.30367895936260
H	-6.47290117909488	-1.91196174117133	7.97124992454478

H	-7.26176243066993	-2.22627464163557	5.55225723474469
C	-4.91877297609632	2.26755369131301	0.76211592604066
H	-5.17774765353793	2.36873322893255	2.88535015193363
C	-5.06745790969757	0.05897453832444	-0.22713064953333
H	-5.42617784671645	-1.55434103587894	1.13862540193590
H	-3.03720173662739	0.84608658817487	9.94651195351502
H	-4.93925855737163	-0.78752714877786	9.55257719767858
C	0.58470467659992	4.76319136318068	6.44455633268685
H	-1.08361642528569	4.39989935555422	7.73553698545905
C	1.05506148391370	3.02093879404794	4.82525953001991
H	-0.20671043231123	1.29703021546678	4.88559272217882
C	-4.84488454509298	1.43340791001106	-0.36260286898513
H	-4.78700993598047	3.33684319025501	0.62864776374685
H	-5.03257100615117	-0.59901633026368	-1.08666187093523
C	1.36554633859139	4.26547090478754	5.39541909115187
H	0.79772573609975	5.72435057350413	6.89692421959864
H	1.68940639874773	2.64374535935232	4.02962566729897
O	-4.54840103561843	2.04519542268078	-1.52934496411050
O	2.43272211632707	4.90176296661697	4.86964345810851
C	-4.33123596813579	1.22126356392020	-2.66568131627827
C	2.79610005323987	6.15992704775383	5.41995155553049
H	-5.24428481591802	0.68521136460009	-2.94484309253301
H	-4.04195608597281	1.89295324294258	-3.47366523984313
H	-3.52622990868987	0.50368689874308	-2.47053310303543
H	3.04875372138274	6.06341415612647	6.48098718044956
H	3.67149486516109	6.48738575637397	4.86107850466954
H	1.98717938484878	6.88824999093263	5.29750552056195
C	0.06990160137049	0.69167849928548	-1.23002048587026
O	0.20021950140288	-0.23157410850917	-2.01135733497308
O	-0.30400117756383	1.92992612999847	-1.62491633849844
C	-0.56834230651005	2.09561768962204	-3.02400730046408
H	0.37538005987791	2.02819698942825	-3.57432115300881
H	-1.21412054200028	1.28102812763749	-3.36293502305032
C	-1.22824855231652	3.44713682918777	-3.19553305045737
H	-0.56803443064037	4.24818546063239	-2.85185939260201
H	-1.46099028201969	3.61917096281746	-4.24986182269853
H	-2.15878902235259	3.48965054954898	-2.62022481121547

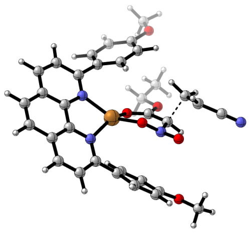	**TS3$_{1ab}$**	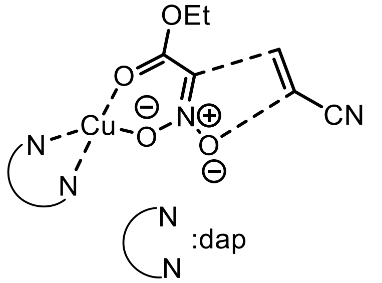

Charge: 1

Multiplicity: 2
[CPCM(CHCl3)M06-2X/6-31G(d)]G_{298K}: -3584.04583267
[CPCM(CHCl3)M06-2X/6-31G(d)]H_{298K}: -3583.94195410
[CPCM(CHCl3)M06-2X/6-31G(d)]E_{SCF}: -3584.55698558
[(CPCM(CHCl3)M06-2X/6-31++G(d, p)]E_{SCF}: -3584.66298519

O	-2.26608654990627	0.47134548607633	2.45860533597996
O	-1.94832490434963	0.41426916328162	0.28937217324105
N	-2.13060988400109	1.11123349558951	1.30195515488829
C	-1.45116962150936	2.31073260257706	1.26216279481491
H	-1.52461280890713	2.77459715399716	0.28309515586026
C	0.30299865400210	1.66701413896009	1.20627401476832
H	0.51255130159601	1.50360203342484	2.26210052319122
H	0.78314873034324	2.55120484870418	0.79228949300479
C	0.26747205328158	0.49847381383582	0.41168034583308
H	0.30905312040297	-0.48604920998071	0.85950675195244
C	-1.60806145962045	3.27672341900553	2.37496548685875
O	-2.23551667118992	3.09854153538771	3.42829993418915
O	-0.96532414719692	4.38582024832557	2.14064801924012
C	-1.03184976784012	5.43271573978463	3.15112194592831
H	-2.01200589436529	5.90632647731831	3.05491627519036
H	-0.95535011223364	4.96467503344793	4.13380672015859
C	0.10670434030432	6.38696847733179	2.87884160647041
H	0.07829299219940	7.19054334032301	3.62059898408073
H	1.06770729230912	5.87140158539738	2.96125526933054
H	0.01973078898282	6.82987182254345	1.88387408060201
Cu	-2.91926708799792	1.35174398737295	4.01336612779622
N	-4.54205949120823	0.24737625047542	4.39103284267940
N	-2.75963035647974	1.36923370311635	6.05758464818932
C	-4.62771642083745	-0.09267748834595	5.69676068072858
C	-5.41048258527670	-0.23533616022998	3.50375574023117
C	-3.70121803953216	0.54192407070342	6.59598277548750
C	-1.89840321782646	1.98589984700091	6.86897410861200
C	-5.58688980202097	-0.99308000669768	6.18017245432956
C	-6.37136860767172	-1.19434553290769	3.90111644934659
C	-5.33943669101764	0.32419512606309	2.14358393188282
C	-3.81340715955505	0.29047117385736	7.97091899027498
C	-1.98464858481149	1.82445346480453	8.27460145494644
C	-0.80579801015383	2.80570752802950	6.30427263132972
C	-5.64590043515101	-1.26463982602954	7.58696608420486
C	-6.45398896032654	-1.56965059230737	5.22116927666856
H	-7.06072132636627	-1.58723231803103	3.16227065965081
C	-5.17886184408780	1.71097158264329	1.98563803926437
C	-5.37692204537765	-0.48670217499945	1.00984248897646
C	-2.92225763842059	0.98386099444868	8.81930945242208
C	-4.80013658736805	-0.63675692801799	8.44780306079349

H	-1.26771249138436	2.34048888508022	8.90264354273992
C	-0.48788207054956	4.03784940981935	6.88101757211528
C	-0.00472931658954	2.32854600899825	5.25473984483416
H	-6.38675539722453	-1.97025975399695	7.94920616962906
H	-7.20288952465948	-2.28643812468268	5.54528543217405
C	-5.01875747929906	2.26304171096417	0.72864918207488
H	-5.21839322834351	2.36297657933779	2.85644141626338
C	-5.19528119534968	0.05524367078962	-0.25966298928503
H	-5.50054198146439	-1.56087081729141	1.11592071362507
H	-2.97597032247214	0.83212379750269	9.89352518694728
H	-4.85125010434640	-0.82610990359748	9.51549386762957
C	0.59004038637134	4.79495657050433	6.42847802065426
H	-1.09675712046747	4.42552707786095	7.69312162237336
C	1.07523526577107	3.06586616968639	4.79990621223890
H	-0.20016123673481	1.34836354339491	4.82346117728898
C	-4.98982657648037	1.43158786458091	-0.40130251448404
H	-4.89852147917000	3.33303643067418	0.59191883916958
H	-5.18871794404083	-0.60013566101736	-1.12203891032145
C	1.38522150719727	4.30226190798022	5.38799499038096
H	0.80119500757437	5.75051096490023	6.89342794996321
H	1.72146823404018	2.69419373768129	4.01110845574310
O	-4.75915809150136	2.05172211409356	-1.57579753857743
O	2.46568075230006	4.93466340288828	4.88612844936883
C	-4.60794023921365	1.24285627238459	-2.73407654096361
C	2.83032225983422	6.18468672281861	5.45436308578475
H	-5.53408198832442	0.70460727153628	-2.96111269912980
H	-4.37488964311316	1.92779468638061	-3.54799078479670
H	-3.78781054747694	0.52906615603253	-2.60205922886385
H	3.06564025124966	6.07517965294141	6.51803572056894
H	3.71660074708958	6.51066637547195	4.91204387054989
H	2.02914870540373	6.92056248671608	5.32652612783532
C	0.49518495744467	0.58301652673173	-0.98835308841494
N	0.66634750311525	0.66587087655457	-2.13459130851434

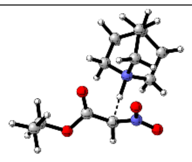	**TS-Deprotonation**	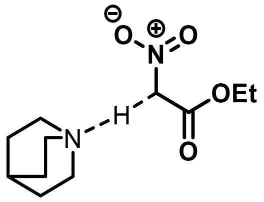

Charge: 0
Multiplicity: 1
[CPCM(CHCl3)M06-2X/6-31G(d)]G298K: -840.86240981
[CPCM(CHCl3)M06-2X/6-31G(d)]H298K: -840.80101551
[CPCM(CHCl3)M06-2X/6-31G(d)]ESCF: -841.13899420
[(CPCM(CHCl3)M06-2X/6-31++G(d, p)]ESCF: -841.19088611

C	-0.82941860411664	-1.19336311831507	-0.64804651806007
C	-3.23607680759322	-1.18491845026180	0.01348313242634
C	-2.16154549920712	-1.97100878459467	-0.75372024900506
H	-0.32282672107188	-1.10146167579086	-1.61302219539844
H	-0.13528060896771	-1.66424083046392	0.05502011609104
H	-2.45947172494679	-2.08435920174194	-1.80159501224050
H	-2.04550948016572	-2.97389709704330	-0.33231693903270
C	-2.13233644397818	0.83215546819705	-0.96445168680852
H	-2.14961521019835	1.89086121727846	-0.69047608258110
H	-1.81477019297822	0.75665678197408	-2.00833476324005
C	-3.49413421024695	0.13916215663060	-0.72293160575232
H	-4.14858953803576	0.77740491453506	-0.12027481512475
H	-3.99673907100862	-0.04338251061587	-1.67731493178397
H	-4.15822877934214	-1.76858484200269	0.07949038769235
H	0.04850200675068	0.85680072002491	-0.20258879198769
C	-2.69982754338895	-0.86918113879678	1.41869635335212
H	-3.48506621325476	-0.42866050393548	2.04016159056444
H	-2.37064449717525	-1.79287993616422	1.90663746248139
C	-1.52034621881026	0.12023942636522	1.27445866153928
H	-1.79633884024929	1.13829516443939	1.56086036066399
H	-0.65009330616287	-0.16813458460776	1.87117155058239
N	-1.08867283995425	0.17622429280905	-0.14459533906506
C	1.24855116501757	1.51827882890788	-0.13429894834247
H	1.59565818106024	1.76205294408188	-1.13301658749563
N	0.90641994969413	2.72509296430761	0.56919806428009
O	0.57734166344328	2.65463501350242	1.74803089559205
O	0.87520817705107	3.77184070331161	-0.07300322191580
C	2.11145731517338	0.55908092880372	0.58826184652103
O	2.33225370159085	0.48782456997061	1.77708811325540
O	2.58636340252660	-0.33507728682967	-0.30664834882947
C	3.39257384036708	-1.39845238814115	0.22669057298234
H	2.94520999501391	-1.75186646337586	1.15871568172995
H	3.33117720681797	-2.18894255200485	-0.52318699597104
C	4.82434000473311	-0.94328738453435	0.43964606168622
H	5.24429878087250	-0.55861577278333	-0.49370589341597
H	4.86581579723339	-0.15847146587530	1.19858780760824
H	5.43586144350710	-1.78597041726071	0.77487906700191

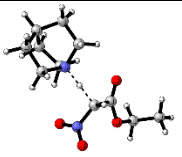	**TS-HAT**	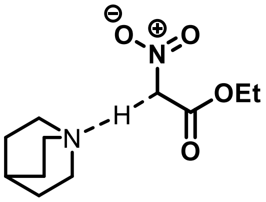

Charge: 1
Multiplicity: 2
[CPCM(CHCl3)M06-2X/6-31G(d)]G_{298K}: -840.62776484

[CPCM(CHCl3)M06-2X/6-31G(d)]H_{298K}: -840.56523487
[CPCM(CHCl3)M06-2X/6-31G(d)]E_{SCF}: -840.90249817
[(CPCM(CHCl3)M06-2X/6-31++G(d, p)]E_{SCF}: -840.94746846

C	-0.98948870764140	0.57344045347253	-0.44463474471996
C	-0.96031247172982	2.96643490355110	0.30243579083046
C	-1.87482780327284	1.77045479654762	0.00445074729174
H	-1.00133731614876	0.43142663299928	-1.52691757640851
H	-1.25681442818964	-0.35927145443088	0.05673941763198
H	-2.57849707249825	2.00238098361455	-0.79848653102571
H	-2.44832819246879	1.49626463275550	0.89407954133698
C	0.97726867488509	1.96305629753118	-0.93583157517737
H	1.96240077756827	2.21090122278045	-0.53623522225404
H	1.07951058382042	1.54290469509584	-1.93839688907407
C	-0.00763767381032	3.16484430575941	-0.88450434271575
H	0.57041405145869	4.08576880151629	-0.77761190069929
H	-0.57274326374037	3.22157609095724	-1.81899245384511
H	-1.55757416402800	3.86729089055653	0.46238690105456
H	1.10659401495717	-0.14363708024495	-0.25968814633616
C	-0.13223451224900	2.64968992918310	1.55485533276972
H	0.64639651912652	3.40388047333516	1.69883638315150
H	-0.76043662039240	2.63224296535077	2.44861716505014
C	0.51685918743318	1.24753633766919	1.37799889498364
H	1.57534728017382	1.24158522755251	1.64560872404339
H	-0.00722470117357	0.46742726896211	1.93295746520228
N	0.40544050554220	0.90683595194042	-0.06296941752597
C	1.72029957627092	-1.27704475774674	-0.54024352088628
H	2.76937151395838	-1.01821580011767	-0.68205218270336
N	1.50417940022469	-2.09914068458966	0.65884628980094
O	0.40115033627668	-2.02707006688300	1.17316034602341
O	2.42817887707493	-2.78330231822490	1.03522181800561
C	0.97505667320377	-1.73124117891824	-1.76961273267019
O	0.83724051732013	-0.97562472624837	-2.70362650122749
O	0.51943173613672	-2.95904226949530	-1.65798714660785
C	-0.22574360035785	-3.48813514276692	-2.79109055114376
H	-0.79275296133074	-4.31279835528714	-2.36164022526931
H	-0.90903207710811	-2.71051153332381	-3.13762357486302
C	0.72016311693338	-3.94914793850079	-3.87999688651037
H	1.27500394722253	-3.10546217602845	-4.29721556193913
H	1.42508907653289	-4.68587221625758	-3.48745617180981
H	0.14237928001924	-4.41460236206656	-4.68288091176383

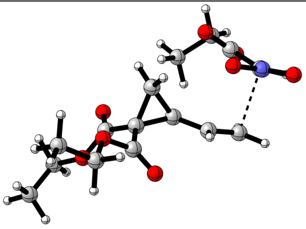	**$TS1_{1ah}$**	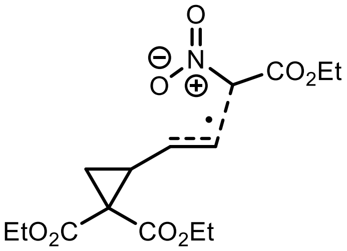

Charge: 0
Multiplicity: 2
[CPCM(CHCl3)M06-2X/6-31G(d)]G_{298K}: -1240.38344397
[CPCM(CHCl3)M06-2X/6-31G(d)]H_{298K}: -1240.30204396
[CPCM(CHCl3)M06-2X/6-31G(d)]E_{SCF}: -1240.70341953
[(CPCM(CHCl3)M06-2X/6-31++G(d, p)]E_{SCF}: -1240.77220686

C	-0.42665829462472	-1.46041318653734	1.26616629104343
C	0.97717393626530	-1.33876925277066	0.78121750590606
C	1.27101840594245	0.12063527203825	0.30040434643071
H	-0.58552437281189	-1.59301676367908	2.32976177262839
H	1.15843843793408	-1.99875864588856	-0.07115070318633
H	1.68506788059050	-1.57565960054289	1.57737368114168
H	2.33196474376712	0.37294447260017	0.33689163400745
O	-0.67689806585326	0.66991557452653	1.27139538099721
O	1.14593953253904	1.52731511729599	2.17939723376826
N	0.55161729563373	0.95054188184031	1.25903647662270
C	0.72851255630848	0.36982949006709	-1.10471190415810
O	0.45167810758375	-0.51043608663822	-1.88420993372117
O	0.63996676252898	1.66944420250944	-1.35324480259847
C	0.16065306534727	2.04521740215320	-2.66514478139931
H	-0.18067117135325	3.07274225521618	-2.53936137743325
H	-0.68929438807344	1.40807080626369	-2.91810976019754
C	1.26901175395048	1.94075384979913	-3.69422658154654
H	1.59461103340929	0.90384287706415	-3.80606399005437
H	0.90239228980494	2.29636253932903	-4.66103846908823
H	2.12381160675539	2.55492762510604	-3.39940325568687
C	-1.51543400693639	-1.84942808312203	0.36198361105403
C	-2.01730725330194	-3.30229493731533	0.36574682595146
C	-2.86456828122263	-2.17541126721586	0.90343883608680
H	-1.47546086650530	-1.42653092179081	-0.63837644459297
H	-3.73356807238260	-1.91373444726561	0.31014877468519
H	-2.98847966086622	-2.13973694733660	1.98185691296556
C	-2.39776565153353	-3.81516946461715	-0.98579878920401
O	-3.04512966804315	-3.17745817413246	-1.78601537002896
O	-1.89918581950373	-5.02836301071387	-1.22158157680381
C	-1.42171066572814	-4.29186738624622	1.32768153608382
O	-0.31119563686844	-4.22210670704159	1.80364028101631
O	-2.29167567183630	-5.26053675622717	1.60756913540853
C	-2.18940385601468	-5.58945788063377	-2.51740747738589

C	-1.55380297291094	-6.96114175009122	-2.55927527528337
H	-1.78357343374407	-4.92100716129090	-3.28129076349560
H	-3.27469393967061	-5.63360351468238	-2.64031833255598
H	-1.74860836533694	-7.42618876566372	-3.52907298708652
H	-0.47228952469179	-6.88828643907584	-2.41922300032121
H	-1.96833719629586	-7.60106255273065	-1.77557824770527
C	-1.81425257023117	-6.30312602619397	2.48024399824453
C	-2.89858207064697	-7.35477986058248	2.55525061811059
H	-1.59823954330538	-5.86323283466023	3.45797723093106
H	-0.88195613772366	-6.70080678361074	2.07034683955451
H	-2.57993224002066	-8.16347829251446	3.21796656462883
H	-3.82537296860577	-6.92805276418360	2.94733104126987
H	-3.09570699171722	-7.77345441081343	1.56462484499684

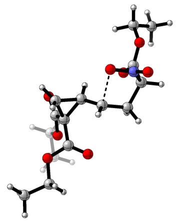	**TS1-a$_{1ah}$**	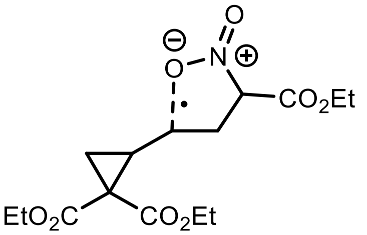

Charge: 1
Multiplicity: 2
[CPCM(CHCl3)M06-2X/6-31G(d)]G_{298K}: -1240.40285576
[CPCM(CHCl3)M06-2X/6-31G(d)]H_{298K}: -1240.32413939
[CPCM(CHCl3)M06-2X/6-31G(d)]E_{SCF}: -1240.72721132
[(CPCM(CHCl3)M06-2X/6-31++G(d, p)]E_{SCF}: -1240.7953747

C	-0.42665829462472	-1.46041318653734	1.26616629104343
C	0.97717393626530	-1.33876925277066	0.78121750590606
C	1.27101840594245	0.12063527203825	0.30040434643071
H	-0.58552437281189	-1.59301676367908	2.32976177262839
H	1.15843843793408	-1.99875864588856	-0.07115070318633
H	1.68506788059050	-1.57565960054289	1.57737368114168
H	2.33196474376712	0.37294447260017	0.33689163400745
O	-0.67689806585326	0.66991557452653	1.27139538099721
O	1.14593953253904	1.52731511729599	2.17939723376826
N	0.55161729563373	0.95054188184031	1.25903647662270
C	0.72851255630848	0.36982949006709	-1.10471190415810
O	0.45167810758375	-0.51043608663822	-1.88420993372117
O	0.63996676252898	1.66944420250944	-1.35324480259847
C	0.16065306534727	2.04521740215320	-2.66514478139931
H	-0.18067117135325	3.07274225521618	-2.53936137743325
H	-0.68929438807344	1.40807080626369	-2.91810976019754
C	1.26901175395048	1.94075384979913	-3.69422658154654
H	1.59461103340929	0.90384287706415	-3.80606399005437
H	0.90239228980494	2.29636253932903	-4.66103846908823

H	2.12381160675539	2.55492762510604	-3.39940325568687
C	-1.51543400693639	-1.84942808312203	0.36198361105403
C	-2.01730725330194	-3.30229493731533	0.36574682595146
C	-2.86456828122263	-2.17541126721586	0.90343883608680
H	-1.47546086650530	-1.42653092179081	-0.63837644459297
H	-3.73356807238260	-1.91373444726561	0.31014877468519
H	-2.98847966086622	-2.13973694733660	1.98185691296556
C	-2.39776565153353	-3.81516946461715	-0.98579878920401
O	-3.04512966804315	-3.17745817413246	-1.78601537002896
O	-1.89918581950373	-5.02836301071387	-1.22158157680381
C	-1.42171066572814	-4.29186738624622	1.32768153608382
O	-0.31119563686844	-4.22210670704159	1.80364028101631
O	-2.29167567183630	-5.26053675622717	1.60756913540853
C	-2.18940385601468	-5.58945788063377	-2.51740747738589
C	-1.55380297291094	-6.96114175009122	-2.55927527528337
H	-1.78357343374407	-4.92100716129090	-3.28129076349560
H	-3.27469393967061	-5.63360351468238	-2.64031833255598
H	-1.74860836533694	-7.42618876566372	-3.52907298708652
H	-0.47228952469179	-6.88828643907584	-2.41922300032121
H	-1.96833719629586	-7.60106255273065	-1.77557824770527
C	-1.81425257023117	-6.30312602619397	2.48024399824453
C	-2.89858207064697	-7.35477986058248	2.55525061811059
H	-1.59823954330538	-5.86323283466023	3.45797723093106
H	-0.88195613772366	-6.70080678361074	2.07034683955451
H	-2.57993224002066	-8.16347829251446	3.21796656462883
H	-3.82537296860577	-6.92805276418360	2.94733104126987
H	-3.09570699171722	-7.77345441081343	1.56462484499684

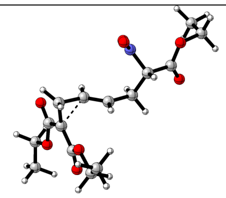

TS1-b$_{1ah}$

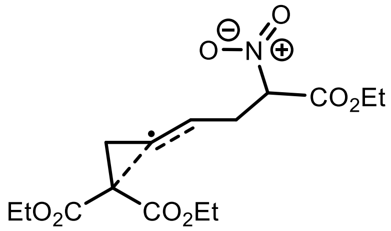

Charge: 0
Multiplicity: 2
[CPCM(CHCl3)M06-2X/6-31G(d)]G_{298K}: -1240.41398952
[CPCM(CHCl3)M06-2X/6-31G(d)]H_{298K}: -1240.33373030
[CPCM(CHCl3)M06-2X/6-31G(d)]E_{SCF}: -1240.73709100
[(CPCM(CHCl3)M06-2X/6-31++G(d, p)]E_{SCF}: -1240.80470397

O	-1.98733505782275	-0.73357779830260	0.62083343317935
O	-1.35150888253081	0.91590539545069	1.86430431116339
N	-1.76985715932034	0.44658980645803	0.82741948802585
C	-1.96405432856518	1.37721803373078	-0.33692235288134
H	-2.00660895234221	2.38090818999817	0.08990770006573

C	-0.77574980189225	1.19468575639130	-1.28663683712009
H	-0.94470790445220	1.88572491784027	-2.12086588001531
H	-0.80611618247866	0.17671541364074	-1.68624285681443
C	0.52740771251307	1.47438446602024	-0.61036715248278
H	0.73862524738745	2.49632782235250	-0.30614245937733
C	-3.29145368154102	1.01722520583287	-0.99815242209847
O	-3.41195631901946	0.80047495878980	-2.17734424509276
O	-4.26534603247146	0.99847760988886	-0.09750776969088
C	-5.55689027220353	0.53208558475740	-0.55385895448138
H	-6.25929200470231	0.92107336025994	0.18278081168336
H	-5.76353330304625	0.97961297570173	-1.52777559639889
C	-5.56882119670877	-0.98296152430201	-0.60866611065863
H	-6.56454801431602	-1.33353327190892	-0.89304813260229
H	-4.84917174991925	-1.34685674620896	-1.34641825607043
H	-5.31494923360721	-1.40010787917311	0.36932014236842
C	1.46313753767084	0.47813324276878	-0.37574711037764
C	3.17830098015864	0.66216207717819	-1.13962387972397
C	2.77437395314649	0.68153149203476	0.29736543380966
H	1.19897455385886	-0.54951110129565	-0.61211624589849
H	3.13170241162080	-0.17177157186073	0.86702942178717
H	2.89679705217891	1.63749426168053	0.79710199859498
C	3.56423285585543	-0.65658341600222	-1.68355256455107
O	3.38391354910439	-1.68997409125539	-1.06246067401729
O	4.12852910182674	-0.62196210439596	-2.89071556351712
C	3.32507387146765	1.91894648639698	-1.90362713368297
O	3.55638153124652	2.01634023948355	-3.08970591205229
O	3.13855974140839	2.99238772403741	-1.11363576936556
C	4.51199669815585	-1.89982864679830	-3.42906623639238
C	5.09751088673043	-1.64695101616727	-4.80071633917546
H	3.62776619057222	-2.54189019497800	-3.46947236193675
H	5.23573157147141	-2.36238804583091	-2.75197538260352
H	5.41251200300991	-2.59435848212551	-5.24611472631166
H	4.35703197237478	-1.18184190240233	-5.45666710905976
H	5.96757546499462	-0.98867969300460	-4.73255639920697
C	3.16793578310300	4.26604807570678	-1.77725258782296
C	2.90375555557494	5.32243320474240	-0.72670623230787
H	2.40906265639286	4.26893146696062	-2.56514508916584
H	4.14624019126310	4.39321442859225	-2.24873502102379
H	2.93534352604548	6.31434056322143	-1.18502416864442
H	1.91883608939590	5.18062019759576	-0.27300568725705
H	3.66132038841089	5.27845852849957	0.06012447920217

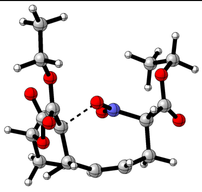	**TS1-c$_{1ah}$**	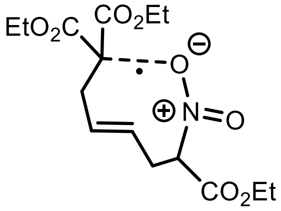

Charge: 0
Multiplicity: 2
[CPCM(CHCl3)M06-2X/6-31G(d)]G_{298K}: -1240.38947408
[CPCM(CHCl3)M06-2X/6-31G(d)]H_{298K}: -1240.31133687
[CPCM(CHCl3)M06-2X/6-31G(d)]E_{SCF}: -1240.71421939
[(CPCM(CHCl3)M06-2X/6-31++G(d, p)]E_{SCF}: -1240.77881288

O	-1.02467442907761	-0.51504487298988	0.61987267167930
O	-0.12091295693239	1.38766742500970	1.25636050666994
N	-0.92314759715238	0.71547751965725	0.52276571835740
C	-1.64292469149507	1.39890253775105	-0.59070177697970
H	-1.63419163516140	2.46195951500142	-0.33653451450177
C	-0.93508108810553	1.14016040424616	-1.93724090522695
H	-1.42636922775519	1.75217322787535	-2.69781296714605
H	-1.05464748130630	0.08485806262923	-2.19933790525858
C	0.49270473063722	1.49181684821233	-1.66138422088876
H	0.72295111368621	2.55177509232592	-1.54585377160450
C	-3.08188211917873	0.88522456023036	-0.58180110187032
O	-3.64634626699498	0.48524397399204	-1.56957323778643
O	-3.61175909228459	0.96452333028400	0.63299665176249
C	-4.93398074450917	0.40322762858558	0.79496897276657
H	-5.30642358434966	0.85267988488278	1.71551103317641
H	-5.55420073587671	0.72247351651972	-0.04490677025239
C	-4.85168535732535	-1.10753223254457	0.89710861373687
H	-5.84616107178857	-1.51863211215016	1.09122545090960
H	-4.47516271532333	-1.53597705610029	-0.03514666389600
H	-4.18638116640333	-1.39617455486685	1.71505082637457
C	1.31521902821363	0.58254593152131	-1.14313867021980
C	1.72620897422765	0.90927813765070	1.24280035919028
C	2.35595203554742	0.98832926557384	-0.14240281089699
H	1.05710805595536	-0.47386531014161	-1.19463790434753
H	2.67734248259872	2.01776133215641	-0.31177701684027
H	3.24171579426557	0.34192351603338	-0.14690160355441
C	2.15214235161420	2.01380268958547	2.17226134588274
O	3.05521240053204	2.77431669971261	1.89109634686683
O	1.44956758244071	2.07833498121341	3.29337367086316
C	1.54477797666697	-0.45386031416259	1.85639263390462
O	1.17231558248159	-0.66449334580887	2.98581423835759
O	1.84540920364823	-1.41702118973028	0.97978864604669
C	1.84452468898844	3.11478550846779	4.21370702979140
C	0.91959601461637	3.02494092291965	5.40661678711185

H	1.77059120346383	4.07966181969811	3.70466345456131
H	2.89061899121169	2.95211094759482	4.48759953577983
H	1.18916887372856	3.79115754018292	6.13812156169452
H	-0.11810605571129	3.18364492311740	5.10188697824110
H	1.00003394722765	2.04402468573768	5.88187977856778
C	1.54720496806972	-2.75671659855483	1.41083260769435
C	1.86383677758066	-3.67757868476743	0.25347285138368
H	0.49085035590195	-2.79332197910737	1.69238966926591
H	2.14755863349812	-2.98289759696792	2.29610501976306
H	1.64263355237662	-4.71063283986247	0.53393025327036
H	1.25893724047904	-3.41769423052195	-0.61952733638791
H	2.92076838707345	-3.60967002009149	-0.01750434601205

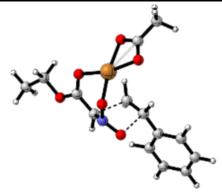	**TS4$_{1a}$**	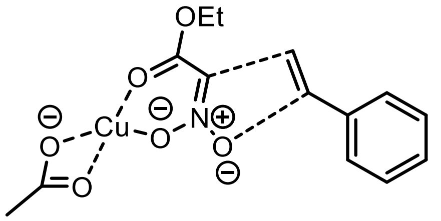

Charge: 0

Multiplicity: 2

[M06-2X/6-31G(d)]G_{298K}: -2689.25693420

[M06-2X/6-31G(d)]H_{298K}: -2689.18411326

[M06-2X/6-31G(d)]E_{SCF}: -2689.51173714

[M06-2X/6-31++G(d, p)]E_{SCF}: -2689.596468272829

O	-0.84049482443306	-1.59044318139881	-0.01143172879315
O	-0.64230290770213	-0.35553558812498	1.75859590272286
N	-1.23819063936010	-0.70705229743996	0.74599851423498
C	-2.37248114020217	0.07099593697260	0.37678500826888
H	-2.58157814147936	0.80891037747885	1.14073948058031
C	-1.20448793976471	1.37869981789087	-0.99162793673479
H	-1.98192205510820	2.13636574073708	-0.98441972552327
H	-1.27027758344089	0.62790320624300	-1.76998328912572
C	-0.03312519409562	1.61840659430479	-0.32444211585797
H	0.00659589911495	2.43465069162166	0.38993170662405
C	1.13764169881786	0.76056271306207	-0.34351936933922
C	2.03255865142142	0.80495912306193	0.73691384218953
C	1.44430853550092	-0.14663658588951	-1.36826283400820
C	3.15761618174265	0.00263039968576	0.80387012627779
C	2.56393752675261	-0.95939646489386	-1.31992604992340
C	3.42416071766423	-0.88566867518579	-0.23153360376864
F	1.78723146039719	1.62765553963006	1.75335791177236
F	3.97808474086728	0.06799351631602	1.84717184189859
F	4.49747767761278	-1.66193350830290	-0.18140071000338
F	2.82638184066651	-1.80040677305146	-2.31529654402255
F	0.66660800887649	-0.24312418375069	-2.44499931412310
C	-3.46179039823045	-0.41396078182536	-0.44192016175167

C	-3.42591582041036	-1.55971625833808	-1.26112396861187
C	-4.63123481918965	0.37439824633483	-0.43426003184111
C	-4.53856579130358	-1.90244758942955	-2.01892967060062
H	-2.53652883784260	-2.17391857309481	-1.28391262519811
C	-5.72982265386805	0.02997845807214	-1.20575912693322
H	-4.66600222472683	1.26366302973619	0.19020751169406
C	-5.68935046598356	-1.11575796488361	-2.00050229521117
H	-4.50515987857245	-2.79642496721079	-2.63351297264258
H	-6.62073475340650	0.64922190787153	-1.18469961388470
H	-6.54958453031451	-1.39380567619920	-2.60074510836491

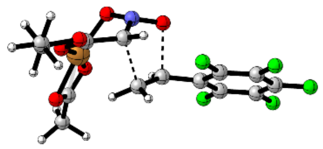	**TS4$_{1j}$**	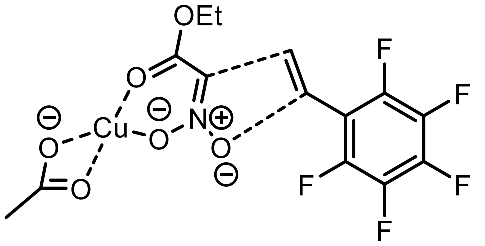

Charge: 0
Multiplicity: 2
[M06-2X/6-31G(d)]G_{298K}: -3185.29892969
[M06-2X/6-31G(d)]H_{298K}: -3185.21759311
[M06-2X/6-31G(d)]E_{SCF}: -3185.50919575
[M06-2X/6-31++G(d, p)]E_{SCF}: -3185.618082577372

O	-0.84049482443306	-1.59044318139881	-0.01143172879315
O	-0.64230290770213	-0.35553558812498	1.75859590272286
N	-1.23819063936010	-0.70705229743996	0.74599851423498
C	-2.37248114020217	0.07099593697260	0.37678500826888
H	-2.58157814147936	0.80891037747885	1.14073948058031
C	-1.20448793976471	1.37869981789087	-0.99162793673479
H	-1.98192205510820	2.13636574073708	-0.98441972552327
H	-1.27027758344089	0.62790320624300	-1.76998328912572
C	-0.03312519409562	1.61840659430479	-0.32444211585797
H	0.00659589911495	2.43465069162166	0.38993170662405
C	1.13764169881786	0.76056271306207	-0.34351936933922
C	2.03255865142142	0.80495912306193	0.73691384218953
C	1.44430853550092	-0.14663658588951	-1.36826283400820
C	3.15761618174265	0.00263039968576	0.80387012627779
C	2.56393752675261	-0.95939646489386	-1.31992604992340
C	3.42416071766423	-0.88566867518579	-0.23153360376864
F	1.78723146039719	1.62765553963006	1.75335791177236
F	3.97808474086728	0.06799351631602	1.84717184189859
F	4.49747767761278	-1.66193350830290	-0.18140071000338
F	2.82638184066651	-1.80040677305146	-2.31529654402255
F	0.66660800887649	-0.24312418375069	-2.44499931412310
C	-3.46179039823045	-0.41396078182536	-0.44192016175167

C	-3.42591582041036	-1.55971625833808	-1.26112396861187
C	-4.63123481918965	0.37439824633483	-0.43426003184111
C	-4.53856579130358	-1.90244758942955	-2.01892967060062
H	-2.53652883784260	-2.17391857309481	-1.28391262519811
C	-5.72982265386805	0.02997845807214	-1.20575912693322
H	-4.66600222472683	1.26366302973619	0.19020751169406
C	-5.68935046598356	-1.11575796488361	-2.00050229521117
H	-4.50515987857245	-2.79642496721079	-2.63351297264258
H	-6.62073475340650	0.64922190787153	-1.18469961388470
H	-6.54958453031451	-1.39380567619920	-2.60074510836491

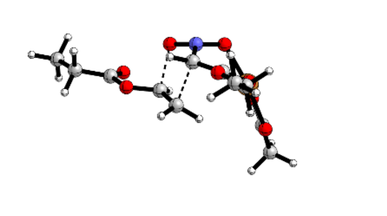

TS4$_{1aa}$

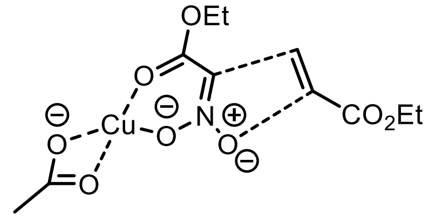

Charge: 0
Multiplicity: 2
[M06-2X/6-31G(d)]G_{298K}: -2725.39618237
[M06-2X/6-31G(d)]H_{298K}: -2725.32052644
[M06-2X/6-31G(d)]E_{SCF}: -2725.63933343
[M06-2X/6-31++G(d, p)]E_{SCF}: -2725.725642762174

O	-0.84049482443306	-1.59044318139881	-0.01143172879315
O	-0.64230290770213	-0.35553558812498	1.75859590272286
N	-1.23819063936010	-0.70705229743996	0.74599851423498
C	-2.37248114020217	0.07099593697260	0.37678500826888
H	-2.58157814147936	0.80891037747885	1.14073948058031
C	-1.20448793976471	1.37869981789087	-0.99162793673479
H	-1.98192205510820	2.13636574073708	-0.98441972552327
H	-1.27027758344089	0.62790320624300	-1.76998328912572
C	-0.03312519409562	1.61840659430479	-0.32444211585797
H	0.00659589911495	2.43465069162166	0.38993170662405
C	1.13764169881786	0.76056271306207	-0.34351936933922
C	2.03255865142142	0.80495912306193	0.73691384218953
C	1.44430853550092	-0.14663658588951	-1.36826283400820
C	3.15761618174265	0.00263039968576	0.80387012627779
C	2.56393752675261	-0.95939646489386	-1.31992604992340
C	3.42416071766423	-0.88566867518579	-0.23153360376864
F	1.78723146039719	1.62765553963006	1.75335791177236
F	3.97808474086728	0.06799351631602	1.84717184189859
F	4.49747767761278	-1.66193350830290	-0.18140071000338
F	2.82638184066651	-1.80040677305146	-2.31529654402255
F	0.66660800887649	-0.24312418375069	-2.44499931412310
C	-3.46179039823045	-0.41396078182536	-0.44192016175167
C	-3.42591582041036	-1.55971625833808	-1.26112396861187

C	-4.63123481918965	0.37439824633483	-0.43426003184111
C	-4.53856579130358	-1.90244758942955	-2.01892967060062
H	-2.53652883784260	-2.17391857309481	-1.28391262519811
C	-5.72982265386805	0.02997845807214	-1.20575912693322
H	-4.66600222472683	1.26366302973619	0.19020751169406
C	-5.68935046598356	-1.11575796488361	-2.00050229521117
H	-4.50515987857245	-2.79642496721079	-2.63351297264258
H	-6.62073475340650	0.64922190787153	-1.18469961388470
H	-6.54958453031451	-1.39380567619920	-2.60074510836491

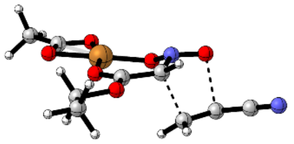	**TS4$_{1ab}$**	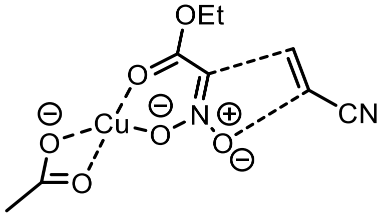

Charge: 0
Multiplicity: 2
[M06-2X/6-31G(d)]G_{298K}: -2550.59852585
[M06-2X/6-31G(d)]H_{298K}: -2550.53148716
[M06-2X/6-31G(d)]E_{SCF}: -2550.77319855
[M06-2X/6-31++G(d, p)]E_{SCF}: -2550.850046348336

O	-0.84049482443306	-1.59044318139881	-0.01143172879315
O	-0.64230290770213	-0.35553558812498	1.75859590272286
N	-1.23819063936010	-0.70705229743996	0.74599851423498
C	-2.37248114020217	0.07099593697260	0.37678500826888
H	-2.58157814147936	0.80891037747885	1.14073948058031
C	-1.20448793976471	1.37869981789087	-0.99162793673479
H	-1.98192205510820	2.13636574073708	-0.98441972552327
H	-1.27027758344089	0.62790320624300	-1.76998328912572
C	-0.03312519409562	1.61840659430479	-0.32444211585797
H	0.00659589911495	2.43465069162166	0.38993170662405
C	1.13764169881786	0.76056271306207	-0.34351936933922
C	2.03255865142142	0.80495912306193	0.73691384218953
C	1.44430853550092	-0.14663658588951	-1.36826283400820
C	3.15761618174265	0.00263039968576	0.80387012627779
C	2.56393752675261	-0.95939646489386	-1.31992604992340
C	3.42416071766423	-0.88566867518579	-0.23153360376864
F	1.78723146039719	1.62765553963006	1.75335791177236
F	3.97808474086728	0.06799351631602	1.84717184189859
F	4.49747767761278	-1.66193350830290	-0.18140071000338
F	2.82638184066651	-1.80040677305146	-2.31529654402255
F	0.66660800887649	-0.24312418375069	-2.44499931412310
C	-3.46179039823045	-0.41396078182536	-0.44192016175167
C	-3.42591582041036	-1.55971625833808	-1.26112396861187
C	-4.63123481918965	0.37439824633483	-0.43426003184111

C	-4.53856579130358	-1.90244758942955	-2.01892967060062
H	-2.53652883784260	-2.17391857309481	-1.28391262519811
C	-5.72982265386805	0.02997845807214	-1.20575912693322
H	-4.66600222472683	1.26366302973619	0.19020751169406
C	-5.68935046598356	-1.11575796488361	-2.00050229521117
H	-4.50515987857245	-2.79642496721079	-2.63351297264258
H	-6.62073475340650	0.64922190787153	-1.18469961388470
H	-6.54958453031451	-1.39380567619920	-2.60074510836491

All Intermediates

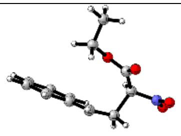	**1a-II**	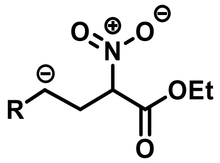

Charge: -1
Multiplicity: 1
[CPCM(CHCl3)M06-2X/6-31G(d)]G_{298K}: -820.76790455
[CPCM(CHCl3)M06-2X/6-31G(d)]H_{298K}: -820.70838685
[CPCM(CHCl3)M06-2X/6-31G(d)]E_{SCF}: -820.97449474
[(CPCM(CHCl3)M06-2X/6-31++G(d, p)]E_{SCF}: -821.03469012

O	-4.41291758921908	2.63218233754600	0.95142211505245
O	-3.97214044526509	1.59929871081590	-0.89798026966962
N	-3.68802081059235	1.94690058889955	0.23866017326221
C	-2.31362439976638	1.63518784972044	0.71235584736294
H	-2.12485533150405	0.60134748163216	0.41449828754437
C	-1.27340809409576	2.56762715199402	0.01838580729951
H	-1.44667045925073	3.57910064738823	0.39958900263230
H	-1.53515435741644	2.56060043064899	-1.05033572053531
C	0.09822235696570	2.08396158749602	0.30723909354465
H	0.64263364488506	2.55472483055643	1.12076483148114
C	0.59168963543043	0.90105059268104	-0.24666750367034
C	1.86949571863440	0.35537061089502	0.13223914595345
C	-0.11507359885954	0.12640173858116	-1.23421032015041
C	2.37400544511506	-0.80604268065206	-0.41543637653977
H	2.45034125970091	0.89272678456087	0.88087721196326
C	0.41463771387830	-1.03968387676960	-1.77233150174320
H	-1.08631209166975	0.46848172286251	-1.58587359674499
C	1.66091628674650	-1.53689507280309	-1.38317322464883
H	3.34756250641573	-1.16358794072408	-0.08267367577167
H	-0.16691977803833	-1.57779193366423	-2.52010188639869
H	2.06175592019196	-2.45214495586410	-1.80656749667342
C	-2.30158277482812	1.79403799282076	2.22406356675121
O	-1.83923420573885	2.73330614530842	2.82392491807631
O	-2.89720181333611	0.75300087689831	2.81078426565163
C	-3.00875031553086	0.82539083728178	4.24274598537189

H	-2.00735736809460	0.94267623559439	4.66574393018292
H	-3.59353106160131	1.71302813868278	4.50102491186860
C	-3.67711686256445	-0.45013765890408	4.70650965782957
H	-3.78319676605876	-0.43538091059452	5.79450899684425
H	-4.67029823910324	-0.54882627398401	4.26065134837321
H	-3.07895963543025	-1.32117591890509	4.42641786550032

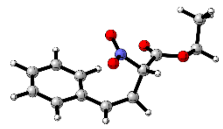	**1a-I**	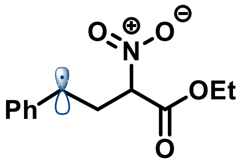

Charge: 0
Multiplicity: 2
[CPCM(CHCl3)M06-2X/6-31G(d)]G_{298K}: -820.67424219
[CPCM(CHCl3)M06-2X/6-31G(d)]H_{298K}: -820.61392927
[CPCM(CHCl3)M06-2X/6-31G(d)]E_{SCF}: -820.88230005
[(CPCM(CHCl3)M06-2X/6-31++G(d, p)]E_{SCF}: -820.92835354

O	2.98036546823536	0.06477601034860	-0.68812673898273
O	1.84032810129782	-1.46013707462222	-1.71049272644955
N	1.94022011697917	-0.49720935734855	-0.97739183120722
C	0.67722047636207	-0.01402558079387	-0.31698557910987
H	-0.09819526785767	-0.09356216987360	-1.08130742007112
C	0.38449753647770	-0.92128628665550	0.88781345901036
H	1.16203529403048	-0.75229767947041	1.63691672041957
H	0.45179251913601	-1.96068123741885	0.54677719226020
C	-0.96577702546552	-0.61580124823204	1.45029511843025
H	-1.01723602375405	0.01028232307876	2.33437754184334
C	-2.17954359419559	-1.04413095584797	0.84980416438941
C	-3.42027348370962	-0.67141151315819	1.42757130462535
C	-2.21324318477043	-1.83988902908506	-0.32468918996581
C	-4.62085588146985	-1.07144815452662	0.86571678481624
H	-3.41428178284014	-0.06145851380333	2.32721714679004
C	-3.42178325349948	-2.23314558506916	-0.88086524685396
H	-1.28558625753078	-2.15214133056493	-0.79726589172854
C	-4.63160904939642	-1.85479586065146	-0.29355008119761
H	-5.55725661016543	-0.77312907716024	1.32765634124210
H	-3.42366390962071	-2.84250749807862	-1.77964113395159
H	-5.57331122670711	-2.16885857988346	-0.73221312460746
C	0.89497985731739	1.44030195870574	0.08521047879703
O	0.95454177566359	1.82097243096377	1.22794949413254
O	1.00636366774906	2.20470048811457	-0.99406784166101
C	1.37830255524551	3.58299506913693	-0.76650990976086
H	1.08447983390872	4.09696502010568	-1.68162049728308
H	0.79261579912946	3.96658223146524	0.07118727764198
C	2.86922363640249	3.68534706498789	-0.51064082324124

H	3.15137754252277	4.73557940375236	-0.39655045597459
H	3.14045171527088	3.15046690789817	0.40313813486089
H	3.42859749954345	3.26062402305482	-1.34818268465466

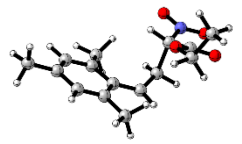	**1k-II**	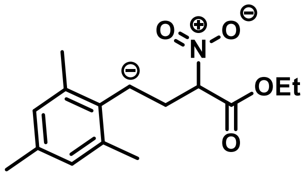

Charge: -1
Multiplicity: 1
[CPCM(CHCl3)M06-2X/6-31G(d)]G_{298K}: -938.56738903
[CPCM(CHCl3)M06-2X/6-31G(d)]H_{298K}: -938.50075391
[CPCM(CHCl3)M06-2X/6-31G(d)]E_{SCF}: -938.85574504
[(CPCM(CHCl3)M06-2X/6-31++G(d, p)]E_{SCF}: -938.92123848

O	3.97867817334707	-0.70502337992067	-0.55228037913673
O	2.73043630436715	-2.46866581257704	-0.49911377692628
N	2.89623679177894	-1.25811723804818	-0.41281980340838
C	1.70840113366548	-0.46725531999579	-0.00872724509795
H	0.89912028676414	-0.78288155601329	-0.67076443379133
C	1.30530040633472	-0.77759488075876	1.46931021199743
H	2.13373199142623	-0.43357988483402	2.09811503402782
H	1.26774598414179	-1.86963962030128	1.54612928880515
C	0.05951588821456	-0.01642335916012	1.78805162470036
H	0.26000496369079	0.92637590983656	2.28643005795752
C	-1.12305668948810	-0.08152234386186	1.01488043671829
C	-2.00276947551974	1.06239361526624	0.92289607327171
C	-1.56324186788922	-1.25247769953638	0.29693306448343
C	-3.13031122781035	1.04196428296332	0.11963587709949
C	-2.70377905833535	-1.20822303974701	-0.50542118913481
C	-3.50578601074759	-0.07613184412461	-0.64134407747676
H	-3.74680379506192	1.94158524493312	0.07389203921143
H	-2.99801807026563	-2.12186284701262	-1.02533107479746
C	1.98050154792385	1.01910595331872	-0.15682048326520
O	2.67639055957735	1.66958809705536	0.58549788349758
O	1.32601815050777	1.51908862008954	-1.20807054375977
C	1.47918537317499	2.93189890233527	-1.43686110943410
H	0.61070183953314	3.20802141953927	-2.03607432254609
H	1.43550990411437	3.44878787038938	-0.47555056616475
C	-1.66550628976753	2.31307708330643	1.68882417933808
H	-1.59863990031885	2.12465444372018	2.76910293324822
H	-0.68378562024456	2.70994128683836	1.39152142864909
H	-2.41721252978322	3.09100459634613	1.52395223211230
C	-4.71533160667245	-0.05297956114900	-1.54158692314984
H	-5.57056448902226	0.43675014036887	-1.06088430711671
H	-4.53038095556785	0.48599742748168	-2.48104278045565

H	-5.02299282684126	-1.06936455779369	-1.81011877242674
C	-0.91225613889014	-2.59608512221490	0.53021499270770
H	0.07730639491757	-2.71798528776933	0.07083763345994
H	-0.77663426230520	-2.76345593562382	1.60764882316110
H	-1.54857917178641	-3.39694952826730	0.13900794787454
C	2.77860185593673	3.22000056895229	-2.16557265149227
H	2.85317174923287	4.28973406707596	-2.38123723803634
H	3.63393733112149	2.92666104061782	-1.55207678460351
H	2.81430440623613	2.67063526125238	-3.11025129437054

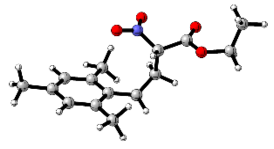	**1k-I**	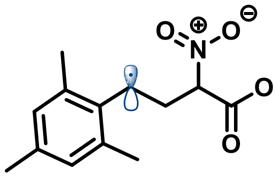

Charge: 0
Multiplicity: 2
[CPCM(CHCl3)M06-2X/6-31G(d)]G_{298K}: -938.47526679
[CPCM(CHCl3)M06-2X/6-31G(d)]H_{298K}: -938.40626562
[CPCM(CHCl3)M06-2X/6-31G(d)]E_{SCF}: -938.76406460
[(CPCM(CHCl3)M06-2X/6-31++G(d, p)]E_{SCF}: -938.81775324

O	-1.96081116575490	-0.71172488015244	0.52700144707386
O	-1.23168695807932	-0.51258388589670	2.54916158553634
N	-1.70862301705207	-0.06653584635100	1.52527914568088
C	-1.96690803426928	1.41104055910337	1.46223079533600
H	-1.62711639842263	1.81010483656873	2.42046907832108
C	-1.14719874049905	1.99155586666188	0.29302928493322
H	-1.29994370204850	3.07500147281313	0.32791049924974
H	-1.57553552330884	1.62243070046950	-0.63560291358355
C	0.29791604293900	1.65773685755721	0.49887592373415
H	0.72704723776776	2.18505506724220	1.34469695002753
C	1.08514649380534	0.60482897104257	-0.05050210179343
C	2.23039480727474	0.18020120800623	0.70397880140292
C	0.83663581642043	-0.04221731698067	-1.30312462977669
C	3.03677856884565	-0.84479699419500	0.23204920146099
C	1.68141864030151	-1.06263417553440	-1.72595517876740
C	2.77996971606337	-1.49405391520922	-0.97913271513504
H	3.89164233096222	-1.15676708432244	0.82867244425066
H	1.48447643546728	-1.53097491922270	-2.68845532435032
C	-3.47149180869448	1.61729522793466	1.33373499628333
O	-4.29494494410023	0.75188314470514	1.49680382021792
O	-3.72771572179603	2.88731148531600	1.04336703158854
C	-5.12467658828681	3.24935048810818	0.93219553262532
H	-5.11753052514664	4.16102284066910	0.33489083932938
H	-5.64202718465470	2.45892683468380	0.38543448223475
C	-5.73114903468426	3.47461721073345	2.30276076590295

H	-6.76965381529297	3.79907475591515	2.19235407362828
H	-5.71728722634391	2.55158242958909	2.88743802782905
H	-5.18158176433975	4.24983198074659	2.84304013823028
C	2.55761282220319	0.79792736265809	2.04083179213406
H	2.77239222261364	1.86956748564833	1.95654941872266
H	1.72213065375664	0.68838133162083	2.74170019763654
H	3.43440909153057	0.31453431140553	2.47754453319732
C	-0.27622186311087	0.37142188790498	-2.23489391507333
H	-1.25213349922339	0.02562797733019	-1.87692867398185
H	-0.32394107835729	1.45895089819671	-2.35090355035144
H	-0.11476431634092	-0.06556256107972	-3.22352468818891
C	3.64734379643376	-2.62897497312195	-1.45436340175950
H	3.25225595374201	-3.59158647669770	-1.10834781309723
H	3.68862402118694	-2.66591459215982	-2.54678299934184
H	4.66686936324134	-2.53537661275638	-1.07001288265439

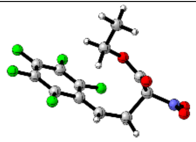	**1j-II**	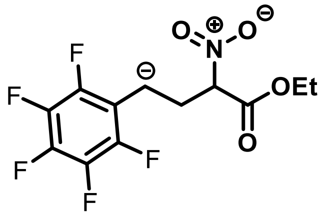

Charge: -1
Multiplicity: 1
[CPCM(CHCl3)M06-2X/6-31G(d)]G_{298K}: -1316.81880309
[CPCM(CHCl3)M06-2X/6-31G(d)]H_{298K}: -1316.74924898
[CPCM(CHCl3)M06-2X/6-31G(d)]E_{SCF}: -1316.97978183
[(CPCM(CHCl3)M06-2X/6-31++G(d, p)]E_{SCF}: -1317.06598585

O	4.52080095898383	-0.46967382091173	0.54323479914961
O	3.99967445416942	-1.81289627896540	-1.06101165255282
N	3.71609988831712	-0.97996290959246	-0.21441093960714
C	2.27628204872047	-0.60765115926940	-0.07197585348443
H	1.74126583837386	-1.20898220119061	-0.80271339661984
C	1.76769754813973	-0.88209407602909	1.38180669890556
H	2.55136926689999	-0.51041029042999	2.04827930423390
H	1.71947573045905	-1.97117556327089	1.48951281518773
C	0.48696169473700	-0.16409732320957	1.67485751304050
H	0.57991602249154	0.77968888888216	2.20013927244351
C	-0.71851286151184	-0.39235324166749	1.02211311222398
C	-1.80690877173526	0.53480713553824	1.10541424908321
C	-1.04346390164248	-1.50301595224841	0.18495846023303
C	-3.02654140270981	0.36908830168070	0.49172210378025
C	-2.26146420890286	-1.67714275050448	-0.43929905530310
C	-3.28846100456474	-0.75060839090501	-0.29673492464537
C	2.06575448813907	0.87266993268740	-0.33900736168213
O	2.69542401918354	1.76672841195643	0.17087336683611

O	1.01420409608052	1.03271799930347	-1.14160666783308
C	0.38874335205307	2.33017126588301	-1.11666502167225
H	-0.62842417724169	2.13854565296773	-1.46325665789875
H	0.34921130653958	2.66139912667038	-0.07468009164191
F	-2.46641112817924	-2.77091600290440	-1.19283757277788
F	-0.09823178369017	-2.45806856727799	-0.03616510087420
F	-1.60350913681705	1.68345870055357	1.79732233573795
F	-3.98086635872084	1.30539691832480	0.63904143659379
F	-4.49648643492981	-0.94283924266450	-0.87096810548590
C	1.10517334924328	3.32336820793549	-2.01159349968712
H	0.54526039632689	4.26292174260600	-2.03557413346824
H	2.11182182351906	3.52291896036465	-1.63908277907306
H	1.17208297798564	2.93505003786383	-3.03167064814274

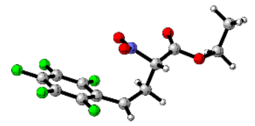	**1j-I**	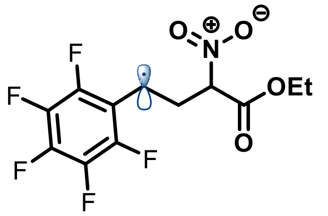

Charge: 0
Multiplicity: 2
[CPCM(CHCl3)M06-2X/6-31G(d)]G_{298K}: -1316.70921622
[CPCM(CHCl3)M06-2X/6-31G(d)]H_{298K}: -1316.63970010
[CPCM(CHCl3)M06-2X/6-31G(d)]E_{SCF}: -1316.87161042
[(CPCM(CHCl3)M06-2X/6-31++G(d, p)]E_{SCF}: -1316.94355441

O	-0.89168011363775	-1.87518550658056	0.35965245174196
O	-2.42699596442808	-1.78942950029649	1.88291953210887
N	-1.83449506427593	-1.35639252688827	0.91717210808432
C	-2.35571241137227	-0.05472306243285	0.36642239747453
H	-2.57640907874662	0.56005595376377	1.24105406174953
C	-1.34911827922454	0.61226387141710	-0.57823539198822
H	-1.84981424724355	1.51689676353767	-0.94724304396171
H	-1.17644692324991	-0.04905314648142	-1.42529995729089
C	-0.08154036015326	0.99564711712983	0.11432804583053
H	-0.16185984100830	1.75438328450985	0.88590992675866
C	1.21996903857171	0.50822387529718	-0.14297871501757
C	2.31246521435337	1.07144948012159	0.56619109848566
C	1.56876222604921	-0.50174390721933	-1.07222065512076
C	3.62107650952499	0.67743075995272	0.37800066349491
C	2.87596685023501	-0.91552972271055	-1.26015695636677
C	3.91200707171158	-0.33078234341872	-0.53843772178489
C	-3.64876521743413	-0.38248913421916	-0.37438348785779
O	-3.94832157534403	-1.47593383742154	-0.77055628471129
O	-4.34713400051934	0.73735117487089	-0.56693562453579
C	-5.55941071733603	0.59955446203836	-1.33906454100710

H	-5.77033194432513	1.61208462792086	-1.68566680607331
H	-5.35189347472006	-0.04470202381470	-2.19654455809851
C	-6.68672350735717	0.04679561953346	-0.48860742458468
H	-7.61098531363045	0.02738535396485	-1.07293406134812
H	-6.45843493786368	-0.97154274860789	-0.16702498410290
H	-6.84671189402345	0.67566639071803	0.39079047619720
F	2.07495564893847	2.04400221734252	1.44904427972247
F	4.60743607420675	1.25719902071572	1.05589310392594
F	5.16605104686833	-0.72055093373741	-0.72430681010874
F	3.14479960143928	-1.87022539156945	-2.14433251254781
F	0.64217826924007	-1.09248924381842	-1.82382797224713

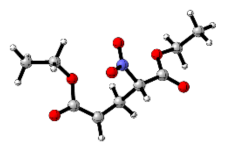	**1aa-II**	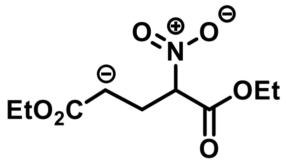

Charge: -1
Multiplicity: 1
[CPCM(CHCl3)M06-2X/6-31G(d)]G_{298K}: -856.92829340
[CPCM(CHCl3)M06-2X/6-31G(d)]H_{298K}: -856.86733687
[CPCM(CHCl3)M06-2X/6-31G(d)]E_{SCF}: -857.12624975
[(CPCM(CHCl3)M06-2X/6-31++G(d, p)]E_{SCF}: -857.18718811

C	1.85600453744938	-2.25650042812265	-0.20286542919538
C	1.21647044389194	-0.94327798080689	0.07219576581785
C	-0.30903636171743	-0.86093327019029	-0.27364166360614
H	1.90488320663065	-3.00197676000927	0.58286818157309
H	1.68516921132626	-0.11612715698973	-0.47053821793759
H	1.26131448886520	-0.69188862590231	1.13919221404279
H	-0.85301558271401	-1.69407555023213	0.16800201943662
N	-0.48121975803221	-1.00724069126386	-1.74797298738990
O	-0.67495773202060	-2.12644974829005	-2.17889986329563
O	-0.38423424181853	-0.00062628592475	-2.43624590876346
C	-0.98861277758400	0.43823579275804	0.13368223553349
O	-2.14684463611664	0.49534408690963	0.48050598816138
O	-0.16588036411252	1.48216419347705	0.08429811678245
C	-0.74894952444643	2.76967591861033	0.35724890853217
H	0.10195231377597	3.40628322476900	0.60164122786930
H	-1.40102799466632	2.68543488424423	1.22939670001608
C	-1.50004629141328	3.27584262940912	-0.85971431150363
H	-2.35151007258789	2.62750334414290	-1.08010501154836
H	-1.87059482142788	4.28786160794270	-0.67326987237966
H	-0.83908718157059	3.29756939140243	-1.73020569521654
C	2.28430400062752	-2.62530505898124	-1.46602763034690
O	2.74910638226638	-3.71261894230967	-1.87139085559511
O	2.17041831919230	-1.56124647949746	-2.38879707640524

C	2.12941964322287	-1.93746450204381	-3.75059435068991
H	1.67197353224099	-2.92663658829396	-3.84899932711761
H	1.47962660848979	-1.20374627253698	-4.24235436436732
C	3.51834355549172	-1.93964100232286	-4.37156261540755
H	4.13130536998059	-2.69367761882779	-3.87206344528997
H	3.47143904616111	-2.16892696553724	-5.44175407705793
H	3.99302465077223	-0.96082909043266	-4.24761730650218

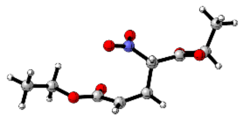	**1aa-I**	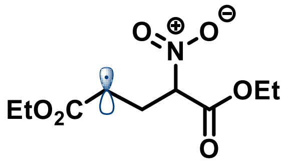

Charge: 0
Multiplicity: 2
[CPCM(CHCl3)M06-2X/6-31G(d)]G298K: -856.80874287
[CPCM(CHCl3)M06-2X/6-31G(d)]H298K: -856.74588599
[CPCM(CHCl3)M06-2X/6-31G(d)]ESCF: -857.00589727
[(CPCM(CHCl3)M06-2X/6-31++G(d, p)]ESCF: -857.05316172

C	1.68258345523702	1.21678333650864	-0.09846552646430
C	0.23472705931932	1.16446077830987	-0.43230821879223
C	-0.64175776721973	0.81657331487413	0.78584264489617
H	2.12948476039160	2.09533820896167	0.35011649419339
H	0.05230299337521	0.41656597544061	-1.20801861157670
H	-0.11369074222473	2.13660545988866	-0.79454285896598
H	-0.46429393937976	1.49087791448617	1.62398413214282
N	-0.23728407651536	-0.54899477073358	1.28031422523336
O	0.72608258008513	-0.59914982420992	2.01743154838237
O	-0.87129254679012	-1.50482107173429	0.87699444772574
C	-2.14104325813315	0.75430737825814	0.50429302739579
O	-2.95957329020244	0.87781885659119	1.38212218507386
O	-2.40401252198577	0.54008979161466	-0.77571134876638
C	-3.79465752813520	0.33099137867824	-1.11508075999955
H	-3.83825359075063	0.50927475873373	-2.18932387749172
H	-4.39425782198675	1.08066119056682	-0.59496684739951
C	2.49448612785694	0.02934163651715	-0.31246926129827
O	2.05076629972401	-1.02403470190144	-0.74135888622317
O	3.77980065455006	0.21480544060986	0.01828475168169
C	4.63373915477437	-0.92907497549379	-0.15035142632469
H	4.23015529519715	-1.75779272781470	0.43837648083171
H	4.62016488153219	-1.22402890195629	-1.20376195845466
C	-4.21070948914078	-1.08188118393490	-0.75551695837456
H	-4.14627424691174	-1.23848758407273	0.32400401504281
H	-5.24320434966236	-1.25292384413889	-1.07210776330556
H	-3.56488044663430	-1.80659193466816	-1.25817534100983
C	6.01841624998628	-0.52643937910963	0.30761938080691
H	6.00724675133153	-0.23913924668916	1.36223738071543

H	6.70649553524366	-1.36654435209877	0.18268363630479
H	6.38819555687041	0.31686450931266	-0.28174037365341

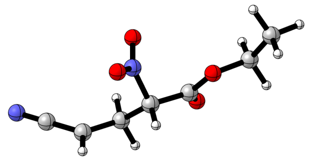	**1ab-I**	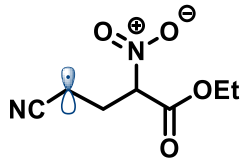

Charge: 0
Multiplicity: 2
[CPCM(CHCl3)M06-2X/6-31G(d)]G_{298K}: -682.00557650
[CPCM(CHCl3)M06-2X/6-31G(d)]H_{298K}: -681.95215967
[CPCM(CHCl3)M06-2X/6-31G(d)]E_{SCF}: -682.13278632
[(CPCM(CHCl3)M06-2X/6-31++G(d, p)]E_{SCF}: -682.16953512

C	0.68338370643292	-2.74369994260258	-0.62102711606933
C	0.75883799456101	-1.34697506618433	-0.09201632714534
C	-0.27573345033733	-0.44236664962082	-0.76110271565867
H	-0.24509097748135	-3.30003151696245	-0.54785844460848
H	1.75135823266794	-0.91714944707091	-0.24455170751970
H	0.54960304223258	-1.32291922056941	0.98418372781705
H	-1.28809353040591	-0.84741564125197	-0.69373145276407
N	0.02699463819688	-0.37116923616338	-2.23385513625899
O	-0.79442375083400	-0.83215651914294	-2.99579162103200
O	1.09857626379826	0.11667203441419	-2.53208644684510
C	1.76422792092684	-3.36745510073108	-1.24554191051302
N	2.67243451374430	-3.89534411270885	-1.75405548496639
C	-0.24761016001916	0.96912249071443	-0.17931800299031
O	0.20427425586448	1.22344356227391	0.90599889439884
O	-0.82728325922114	1.83232800331909	-1.00316719113746
C	-0.88324218741503	3.19961255800141	-0.54717755948389
H	-1.37277369302082	3.21648248096656	0.43042625897895
H	0.14144735718313	3.55921183012345	-0.42067604747989
C	-1.64666873322441	3.98203536128329	-1.59174448323952
H	-1.71726258441800	5.03007998638011	-1.28983515838765
H	-1.13931048342622	3.92999245456050	-2.55793806331848
H	-2.65749747503268	3.58338405123924	-1.70746849272381

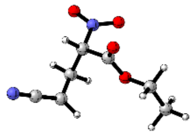	**1ab-II**	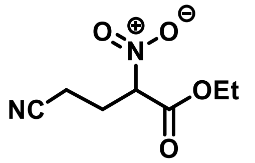

Charge: 0
Multiplicity: 2
[CPCM(CHCl3)M06-2X/6-31G(d)]G_{298K}: -682.12660362
[CPCM(CHCl3)M06-2X/6-31G(d)]H_{298K}: -682.07245301

[CPCM(CHCl3)M06-2X/6-31G(d)]E_{SCF}: -682.25339672
[(CPCM(CHCl3)M06-2X/6-31++G(d, p)]E_{SCF}: -682.30530091

C	1.59703630931145	-1.16107895050156	0.38365547345808
C	1.01502460496396	-1.15431126840527	-0.99246946588410
C	-0.52811875637261	-0.96197393372136	-0.94929512447070
H	1.79961309921399	-0.20902759247864	0.86204755170690
H	1.18738965323293	-2.09236550462146	-1.53258620780212
H	1.42794426237807	-0.34073147555812	-1.59917623078913
H	-0.99353997567205	-1.82522845878551	-0.47286891058976
N	-1.09223423940729	-0.91250319527176	-2.33473020790739
O	-1.04510444900634	-1.94454680282421	-2.98117724778925
O	-1.49184752515966	0.15687461210705	-2.77028710526644
C	1.43599119426296	-2.27220065023592	1.18969906818320
N	1.32057997989937	-3.24001633490605	1.85856941390106
C	-0.95357730838730	0.29865945281035	-0.21333474350811
O	-1.93139446745699	0.35748258633546	0.49471266583281
O	-0.13771279186742	1.32292588161531	-0.44757685315199
C	-0.47855279387540	2.55501358664887	0.20859079919133
H	-1.45454084845904	2.88672220983917	-0.15798687612223
H	-0.56242934013761	2.36663827694179	1.28241926563501
C	0.61739878612494	3.54777225386654	-0.11108507853438
H	0.69397778038471	3.70187278043389	-1.19063087846568
H	0.39730143773834	4.50752746622168	0.36377901241887
H	1.57992783918900	3.18649642842980	0.26059390090386

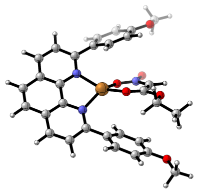	**Cu-1a**	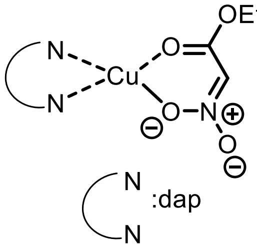

Charge: 1
Multiplicity: 2
[CPCM(CHCl3)M06-2X/6-31G(d)]G_{298K}: -3413.36854505
[CPCM(CHCl3)M06-2X/6-31G(d)]H_{298K}: -3413.27251999
[CPCM(CHCl3)M06-2X/6-31G(d)]E_{SCF}: -3413.82876632
[(CPCM(CHCl3)M06-2X/6-31++G(d, p)]E_{SCF}: -3413.92604746

O	-2.40369959165493	0.62808434246690	2.48912127997727
O	-1.65412623950858	0.56060839773041	0.47186033874713
N	-1.86159552000076	1.22883327811518	1.48009533137532
C	-1.50559595641219	2.53026072276386	1.52310480394914
H	-1.00027612678798	2.89153547847051	0.64254751434043
C	-1.76649427366057	3.40808805865713	2.60704260252546
O	-2.43683348297612	3.17914033453060	3.64636852283817

O	-1.21973347253168	4.59637486918939	2.43190982446617
C	-1.37543836329806	5.57361656497362	3.48662528967000
H	-2.38053893215102	5.99746968979622	3.40856732960189
H	-1.27801007806118	5.06934400216527	4.45032544776621
C	-0.29442138697156	6.61068563957537	3.28380398024815
H	-0.36147004701764	7.36388517818564	4.07387071029386
H	0.69155156334810	6.13852163174508	3.32904806504722
H	-0.40295609727028	7.10791076595248	2.31663834107388
Cu	-3.06601112995668	1.43135807096152	4.12047061818689
N	-4.64321379092535	0.26219140047963	4.50818722183155
N	-2.83115685474419	1.37951335935805	6.15122609117700
C	-4.74964450366112	-0.01847275939945	5.82950152642034
C	-5.50750472340576	-0.25663963533017	3.63556111287630
C	-3.80195028342592	0.60802591252405	6.71314047422332
C	-1.91419873195249	1.95388813355157	6.93270406863360
C	-5.73683746533900	-0.87157806036199	6.34261104754513
C	-6.49863539103257	-1.16814749481152	4.06789769950008
C	-5.37250081394895	0.18552739319151	2.23770233615537
C	-3.90905792958028	0.39119320937087	8.09430313502897
C	-1.98268675898565	1.81706305278021	8.34184773527038
C	-0.79680962657395	2.69117581416391	6.31003097879813
C	-5.80972784524539	-1.08945963915554	7.75818485160972
C	-6.61109995886601	-1.46998428825919	5.40420823667771
H	-7.18174562720584	-1.58619534460252	3.33716570083943
C	-5.18455626280652	1.54978488361223	1.96101027807239
C	-5.30754444841545	-0.73366829536299	1.19168393119415
C	-2.96442879189129	1.04826372430981	8.91532795989745
C	-4.93708758513074	-0.47278735532852	8.60025263219407
H	-1.22206546710961	2.29264140934457	8.95017639475052
C	-0.32675097365789	3.88133565822485	6.87113040582935
C	-0.15384233986122	2.18856413363332	5.16736623428039
H	-6.57885902218767	-1.75283630519407	8.14096023353660
H	-7.38158256510875	-2.15073778959595	5.75444770790835
C	-4.88921487352670	1.97428691112255	0.67919020229925
H	-5.29768066994512	2.28554415900702	2.75538937496059
C	-4.98922828753236	-0.32143096293593	-0.09967103128204
H	-5.44990735829230	-1.79236689738342	1.39044101900355
H	-3.00983268507936	0.91906569945082	9.99281791360320
H	-4.99547264979052	-0.62838547614643	9.67293685419169
C	0.73595196597480	4.57875411384963	6.30133473769910
H	-0.80928138140352	4.29163378718972	7.75396010808661
C	0.90068643227669	2.87041569883325	4.58814344737650
H	-0.45745313032585	1.23260114910694	4.74578689915348
C	-4.75199205003850	1.03368704661016	-0.35410238366210
H	-4.74267141851330	3.02498150399006	0.45085840206309
H	-4.89988548390399	-1.06153979456900	-0.88548522787939

C	1.35417494950915	4.07344640026915	5.15229155358674
H	1.06707362176968	5.50558866855152	6.75425338592231
H	1.40703167765275	2.48171943419522	3.71053375362812
O	-4.40106616908521	1.53416485006083	-1.55533123426112
O	2.38980658426136	4.66712106279542	4.52250722147783
C	-4.13316490336743	0.61357772328484	-2.60297710346380
C	2.95443702431735	5.82726984106931	5.11937464734454
H	-5.02933841285681	0.04133100608652	-2.86429497356879
H	-3.82250060047928	1.21388306892291	-3.45693210764413
H	-3.32768301486047	-0.07148887807711	-2.31759290867304
H	3.30648309749918	5.60940000333727	6.13295312946266
H	3.79627780414675	6.10798432785841	4.48852173890230
H	2.22822982756593	6.64679441109844	5.15077158731515

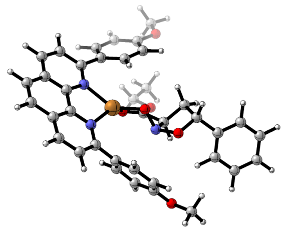	**Cu-1a-IV**	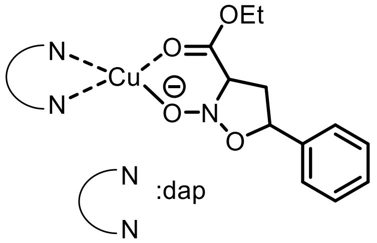

Charge: 0
Multiplicity: 2
[CPCM(CHCl3)M06-2X/6-31G(d)]G_{298K}: -3722.76566682
[CPCM(CHCl3)M06-2X/6-31G(d)]H_{298K}: -3722.65759396
[CPCM(CHCl3)M06-2X/6-31G(d)]E_{SCF}: -3723.36100131
[(CPCM(CHCl3)M06-2X/6-31++G(d, p)]E_{SCF}: -3723.47427111

O	-2.09628003145624	0.58556286825854	2.65932072263304
O	-2.05808130778120	0.46462119037745	0.40736273750455
N	-2.48072704438815	1.25461406073672	1.49461490323244
C	-1.62058243031256	2.44108722023589	1.34519237165108
H	-2.00100490735807	3.01095108218585	0.48754510546967
C	-0.24053114090439	1.86726235913071	1.01470708183090
H	0.24148117306959	1.49793395176835	1.92130582867143
H	0.40915362130406	2.58408668339609	0.51187951339513
C	-0.66606343024480	0.70219691224604	0.09981549229457
H	-0.09033017227393	-0.20011851749102	0.32856740871122
C	-0.55004700240036	1.04050844520189	-1.37102718706690
C	0.67080573374154	0.86052017655814	-2.02663702409582
C	-1.63111965881005	1.57313742478268	-2.07729507390924
C	0.81408780559300	1.21568305438732	-3.36533194118888
C	-1.48827493085002	1.92629534651272	-3.41901497229052
C	-0.26706727941125	1.75039542508910	-4.06610001129345
C	-1.69179719503401	3.36346142931535	2.53289644651876
O	-2.38466176322695	3.19834066715980	3.54062145031688

O	-0.94936844514829	4.42580014492167	2.37264537722052
C	-0.94770629147169	5.40736181718890	3.44420890402994
H	-1.89666572042346	5.94713776694096	3.39348931978136
H	-0.89330009262655	4.87187560848801	4.39370822483434
C	0.24600657198302	6.30530408670401	3.22092050056800
H	0.28941831961777	7.04852379473359	4.02218178378458
H	1.17017338067476	5.72014654538413	3.23397035780024
H	0.16980674117313	6.82866778583686	2.26456241406072
Cu	-2.96328124015175	1.34817188638755	4.12401418212974
N	-4.66462014997117	0.31486610125454	4.51842602206045
N	-2.86376399128014	1.47188639117449	6.16700668924856
C	-4.79620340234912	0.08590620431858	5.84907517713384
C	-5.52372477996633	-0.23235016547437	3.66083429132406
C	-3.86669207444881	0.74496283702519	6.73142200913444
C	-1.95837320171904	2.06087659503703	6.95093607046175
C	-5.79651397988538	-0.74228138841286	6.38070977536145
C	-6.51519488042022	-1.13571088537695	4.11223433954351
C	-5.45453262063936	0.19024884757576	2.24905627722550
C	-4.01838125704711	0.59695360033515	8.11860623446954
C	-2.07664570130240	2.00035360283739	8.36170850695740
C	-0.78316873990157	2.71275120469692	6.33845224611051
C	-5.91112020610437	-0.89278681606741	7.80216988580631
C	-6.64974007910705	-1.38718615410536	5.45576605477977
H	-7.18649826864578	-1.58019024077825	3.38611874979778
C	-5.51037692907196	1.55534231827432	1.93426676757904
C	-5.37074911546094	-0.74405908659341	1.21717691943529
C	-3.09198315564395	1.27995689091133	8.93775586613123
C	-5.06447340353627	-0.23526574184333	8.63842804200396
H	-1.32504786036143	2.48588434283251	8.97297110002071
C	-0.23057567939004	3.86706186089450	6.90151363398227
C	-0.14088388475955	2.13506851255614	5.23069571883163
H	-6.69313436535045	-1.53664604752485	8.19240598760058
H	-7.42308719379841	-2.05670252490258	5.82116553543415
C	-5.46114790636076	1.97233234380710	0.61671613904870
H	-5.61361210137780	2.28927104671408	2.73041570437005
C	-5.28196912329175	-0.33439938532588	-0.10956742548515
H	-5.33762409855891	-1.80492111156777	1.45072685230053
H	-3.17445309429348	1.20258704076389	10.01799693285222
H	-5.15381283906626	-0.33658604963871	9.71545454675628
C	0.92238034374190	4.44888392287514	6.37965046891882
H	-0.71403930064358	4.33994546531849	7.75159524688804
C	1.00601096604416	2.70121462180035	4.70243496015482
H	-0.50934420823988	1.20482144320651	4.80327097165091
C	-5.31496133335282	1.03119285944329	-0.41009739674114
H	-5.50980419425743	3.02445481206848	0.35481064399223
H	-5.17361687766949	-1.07866009163495	-0.88924980467062

C	1.55009448914543	3.86075035464515	5.27640991452612
H	1.31589683553579	5.34951812671405	6.83554361634881
H	1.52410470802061	2.24536922654507	3.86515553358697
O	-5.21376787675337	1.53824076731430	-1.66222925661613
O	2.67699590946263	4.32828839012937	4.70021313046718
C	-5.07737221367809	0.62263476957391	-2.73855795267950
C	3.30146479837728	5.46288408254609	5.28596409747012
H	-5.94055370803874	-0.04946714580939	-2.79141486467779
H	-5.03024584625011	1.22893455301981	-3.64264617922054
H	-4.15708094921876	0.03599332869648	-2.64012384132605
H	3.58396734819820	5.25731808445772	6.32358304952867
H	4.19269376259204	5.65389123920266	4.69045996786743
H	2.64161847022604	6.33652747340188	5.25007800372898
H	-2.58968136569318	1.69564246003260	-1.58033115232932
H	-2.33645589413296	2.33807653402228	-3.95858075749946
H	-0.15833697253115	2.02307899954148	-5.11130061605588
H	1.76820385561209	1.06971698839286	-3.86254398230933
H	1.51232304373026	0.43583285466169	-1.48375729787428

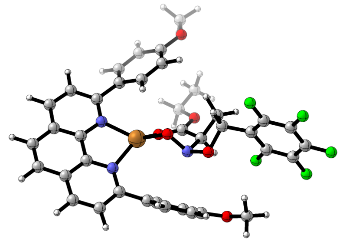	**Cu-1j-IV**	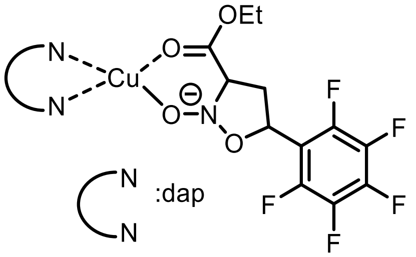

Charge: 1
Multiplicity: 2
[CPCM(CHCl3)M06-2X/6-31G(d)]G_{298K}: -4218.80190044
[CPCM(CHCl3)M06-2X/6-31G(d)]H_{298K}: -4218.68551494
[CPCM(CHCl3)M06-2X/6-31G(d)]E_{SCF}: -4219.35288108
[(CPCM(CHCl3)M06-2X/6-31++G(d, p)]E_{SCF}: -4219.48963055

O	-2.07919146322866	0.69686208689454	2.56516373602798
O	-2.17039333015259	0.68523029699534	0.31214638647650
N	-2.60772487486876	1.36529606499922	1.45912543168634
C	-1.89676285074272	2.64651365812942	1.30975359298010
H	-2.41898866351628	3.20517661191696	0.52586370995997
C	-0.48108477848901	2.27333448520815	0.83500458348781
H	0.19667241006163	2.14250812040158	1.67943147111346
H	-0.06790007383136	3.02652280401872	0.16331986667348
C	-0.74998668203869	0.91952156991602	0.14515314009614
H	-0.19052697730264	0.12094916167570	0.63507141251219
C	-0.49113748823722	0.87594674141011	-1.33635522899298
C	0.33516456630326	-0.08262444866696	-1.91065365967397
C	-1.14080087833700	1.75072957894382	-2.20347051762438
C	0.51635562527412	-0.16988125925174	-3.28592663005813

C	-0.98973104300591	1.67823004361838	-3.57847594952166
C	-0.15366833421039	0.71040488217923	-4.12217352962496
C	-1.95034259831799	3.49500994054889	2.55310268370951
O	-2.57182661452563	3.23152951481812	3.58568534091611
O	-1.27652473628124	4.60528608675692	2.41628018510188
C	-1.25230330457445	5.51468780403629	3.55054396454832
H	-2.22589936478476	6.00930645508805	3.59314975087491
H	-1.11455388598546	4.92130656662022	4.45636468394698
C	-0.11651582327859	6.48308976829816	3.32041140744973
H	-0.05302061231988	7.16597447700507	4.17258619912202
H	0.83141228210585	5.94426453042635	3.23237435767181
H	-0.27801627288208	7.07108136946317	2.41368635980065
Cu	-2.93822987686704	1.30390618874591	4.10965989712880
N	-4.61185290760527	0.22204824801865	4.51428871353881
N	-2.78703492398662	1.34301302520333	6.14641234661096
C	-4.68799090921340	-0.08193813211633	5.83251603830878
C	-5.51024100674650	-0.26804276918081	3.66253530545035
C	-3.74064435219308	0.55426953359054	6.71157028168715
C	-1.87619293557800	1.93337045383925	6.92236495686013
C	-5.65835982380274	-0.94985467815102	6.35484306123861
C	-6.47414241977487	-1.20696606597212	4.09688742890441
C	-5.48795617336513	0.27852157374404	2.29067293022135
C	-3.83551503867914	0.33670573454691	8.09394165037411
C	-1.93456652983097	1.79901699921865	8.33151292055152
C	-0.76463041998305	2.66983962899882	6.28785091482256
C	-5.71096671179098	-1.17736082196269	7.77034148998882
C	-6.54423049209422	-1.54635903415156	5.42745291353267
H	-7.17435093490991	-1.61127717476875	3.37470606973791
C	-5.63043424593609	1.66291222521863	2.11548974910700
C	-5.30603569921475	-0.53507471719538	1.17458407019666
C	-2.89962735321493	1.01208936358351	8.90940831071613
C	-4.84278118894384	-0.54780897773487	8.60678515503781
H	-1.17927012867530	2.28602644366248	8.93722209379004
C	-0.24261183903658	3.82740586091784	6.87295514011249
C	-0.15640391018116	2.17687979675244	5.12147198354742
H	-6.46514105069940	-1.85485793700817	8.15814064658762
H	-7.29552368097634	-2.24548757024120	5.78312480994765
C	-5.53740268303728	2.22388028480621	0.85432335936990
H	-5.82058106448642	2.29744247768422	2.97804677838937
C	-5.17356296571644	0.02290283513404	-0.09261558093718
H	-5.21762700772531	-1.61127376706248	1.29654472088102
H	-2.93681085451940	0.88236957311986	9.98717582552616
H	-4.88926468851265	-0.70686237038478	9.67958555373760
C	0.84439953186187	4.49543396373600	6.31425135219635
H	-0.69873911764145	4.23272480594193	7.77186843020579
C	0.92737940244399	2.82711895571920	4.55838532968151

H	-0.49334420121783	1.24414906093638	4.67437575148086
C	-5.24290907698896	1.41053851632873	-0.24731372693727
H	-5.63422074716505	3.29435450194352	0.70342541940119
H	-4.98259101648639	-0.62528474219564	-0.93928435589646
C	1.43863451761971	3.99167669078768	5.15192000784230
H	1.21436979730603	5.39682983464937	6.78801395670212
H	1.42347789531719	2.43456310207337	3.67680848723167
O	-5.02995856198809	2.05226314479470	-1.41989974018115
O	2.50077179769287	4.54923292955022	4.53465022602840
C	-4.52207769651160	1.28389117058784	-2.49920506472089
C	3.09913043762299	5.68836405880260	5.13918817084361
H	-5.28845470632792	0.61319404599753	-2.90229342313730
H	-4.21664402843308	1.99759987320533	-3.26484201298029
H	-3.65596330786711	0.69529604588074	-2.17649565191491
H	3.44404072043942	5.45437099715390	6.15150530993092
H	3.94919140276077	5.94829927621890	4.51051628040169
H	2.39649272805795	6.52799419739912	5.17453881115218
F	-1.95047784005070	2.69655999531675	-1.71552908053866
F	-1.64532102279474	2.52009129169956	-4.37399661494521
F	0.00303647652165	0.62990091402620	-5.43750223898096
F	1.31575107553568	-1.09914242077010	-3.80239127336676
F	0.98221284475634	-0.96108075208896	-1.14274571312544

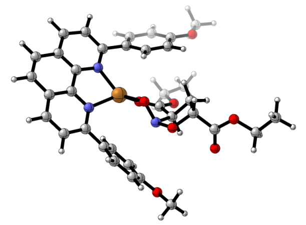	**Cu-1aa-IV**	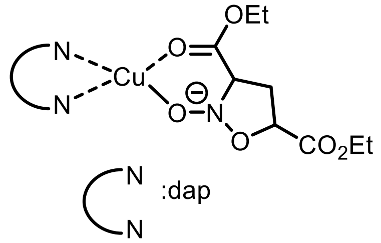

Charge: 1
Multiplicity: 2
[CPCM(CHCl3)M06-2X/6-31G(d)]G_{298K}: -3758.90349524
[CPCM(CHCl3)M06-2X/6-31G(d)]H_{298K}: -3758.79236150
[CPCM(CHCl3)M06-2X/6-31G(d)]E_{SCF}: -3759.48776676
[(CPCM(CHCl3)M06-2X/6-31++G(d, p)]E_{SCF}: -3759.602859126304

O	-2.18943700	0.36058500	2.65307700
O	-2.05785100	0.14831700	0.41400900
N	-2.54478800	0.97471700	1.45106900
C	-1.69324500	2.16399400	1.27322100
H	-2.02536800	2.65906100	0.35313700
C	-0.28441200	1.58896200	1.07542600
H	0.15109400	1.29264700	2.02975600
H	0.38515900	2.27953000	0.56037100
C	-0.64796800	0.34712600	0.23869200

H	-0.08943400	-0.54010900	0.54278900
C	-1.82338200	3.16386200	2.39149400
O	-2.49480200	3.02867900	3.41671600
O	-1.14124500	4.25206500	2.15559100
C	-1.18911000	5.29867200	3.16394600
H	-2.17485900	5.76629200	3.10128700
H	-1.07841600	4.83195400	4.14494600
C	-0.06627900	6.26131200	2.85691100
H	-0.05816400	7.05293600	3.61155100
H	0.89638500	5.74286600	2.88323400
H	-0.19805900	6.71902200	1.87352000
Cu	-2.99628200	1.20042900	4.11426700
N	-4.69592400	0.23583500	4.59028100
N	-2.80776600	1.30764900	6.15638200
C	-4.75619800	-0.06229700	5.90632700
C	-5.62966500	-0.20132800	3.74993600
C	-3.76815200	0.55040100	6.75444600
C	-1.88252900	1.90349800	6.90918600
C	-5.74959500	-0.88780700	6.44876000
C	-6.62550600	-1.09884300	4.20231400
C	-5.59883600	0.38419500	2.39741200
C	-3.84541200	0.35477100	8.14048700
C	-1.92147100	1.79534900	8.32159300
C	-0.78129500	2.63084400	6.24439300
C	-5.77548900	-1.10603400	7.86676200
C	-6.67913700	-1.44062600	5.53398200
H	-7.36191400	-1.46774000	3.49715300
C	-5.47320700	1.77890000	2.28748900
C	-5.67762200	-0.38497900	1.23794300
C	-2.88586500	1.02890500	8.92922700
C	-4.86798100	-0.49823500	8.67872300
H	-1.15355800	2.28966400	8.90540900
C	-0.30598400	3.83896500	6.76130300
C	-0.14364900	2.08534200	5.11819500
H	-6.54281000	-1.75456400	8.27801900
H	-7.45396700	-2.10773600	5.90085400
C	-5.40465300	2.38340500	1.04745700
H	-5.46135900	2.39126600	3.18686800
C	-5.57496900	0.21030900	-0.01635700
H	-5.78124700	-1.46423300	1.31010600
H	-2.90773000	0.91926800	10.00972400
H	-4.89787700	-0.64765400	9.75353500
C	0.76498100	4.50786200	6.17223900
H	-0.78769000	4.28254800	7.62829900
C	0.92734000	2.73437300	4.52836200
H	-0.45382200	1.11856900	4.72589400

C	-5.42165900	1.59738600	-0.11258600
H	-5.31619200	3.46045200	0.94637600
H	-5.59855600	-0.41079800	-0.90368500
C	1.39108200	3.95115500	5.05194400
H	1.10043300	5.44836400	6.59281300
H	1.45047500	2.30462700	3.68022500
O	-5.29072400	2.27111100	-1.27621400
O	2.44295200	4.50326900	4.41134100
C	-5.13004000	1.51215100	-2.46523700
C	2.99353800	5.70067500	4.94258500
H	-6.02494500	0.91934100	-2.68173200
H	-4.97254000	2.23808100	-3.26191100
H	-4.25806400	0.85371600	-2.38666700
H	3.33826500	5.54723300	5.97038400
H	3.83905300	5.95019500	4.30334400
H	2.26003200	6.51397000	4.91670500
C	-0.41104400	0.65333500	-1.23250300
O	-1.15502200	1.32021300	-1.91382800
O	0.75136200	0.15813700	-1.64860100
C	1.13052500	0.48617400	-3.00143800
H	0.35568400	0.11551700	-3.67754700
H	1.17325800	1.57486800	-3.09387700
C	2.47159600	-0.16318500	-3.25799000
H	2.40157400	-1.24788600	-3.14415500
H	2.79795400	0.05931900	-4.27711600
H	3.22263400	0.21664400	-2.56066400

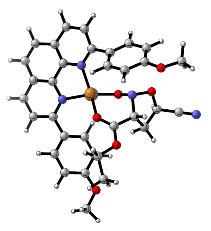	**Cu-1ab-IV**	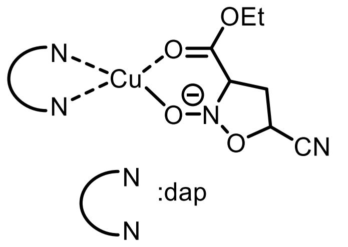

Charge: 1
Multiplicity: 2
[CPCM(CHCl3)M06-2X/6-31G(d)]G_{298K}: -3584.09674927
[CPCM(CHCl3)M06-2X/6-31G(d)]H_{298K}: -3583.99317324
[CPCM(CHCl3)M06-2X/6-31G(d)]E_{SCF}: -3584.61056283
[(CPCM(CHCl3)M06-2X/6-31++G(d, p)]E_{SCF}: -3584.71486274

O	-2.26960813962500	0.49268620860893	2.58339266508000
O	-2.18660641756458	0.33591707709502	0.34374545390158
N	-2.63706246008485	1.14173367981461	1.40898222948988
C	-1.76316901103755	2.31728767347418	1.24498924529403
H	-2.10935949985694	2.85446686008415	0.35504347167878
C	-0.36170039952522	1.73058639578648	1.00009888324092

H	0.14918799974141	1.51855904256417	1.93876449916291
H	0.25756297583748	2.38196357129707	0.38446560971927
C	-0.74483938129918	0.40940014934196	0.29955048695788
H	-0.30249647678534	-0.45646994771581	0.79756232753765
C	-1.84506712328041	3.27936308073497	2.40392915845667
O	-2.50590467213186	3.12505123763089	3.43396933112089
O	-1.13226753127582	4.35166846594599	2.19633394424670
C	-1.12310635452487	5.36267392268621	3.24289642122751
H	-2.08647896657370	5.87692563304848	3.20364249990823
H	-1.02795106381509	4.85480270974638	4.20492265690662
C	0.04173902131430	6.28153959607370	2.96111540818396
H	0.08592739948272	7.04937856319029	3.73870485034779
H	0.98036409242693	5.71988155510986	2.96979946028998
H	-0.07121237394286	6.77336724952814	1.99191642942004
Cu	-3.03822025866924	1.29612907503761	4.09127336832275
N	-4.69436843026322	0.26124351532744	4.56006998332586
N	-2.82614880746823	1.37141354784770	6.13445244278946
C	-4.74388147532364	-0.04186627896878	5.87694290262582
C	-5.60578667914058	-0.22184700725661	3.71944536349580
C	-3.77340404211357	0.59400853314529	6.72855403198598
C	-1.89908699146817	1.96211870598869	6.88954868683494
C	-5.70638844446977	-0.90576233855162	6.41729037106179
C	-6.56398477281100	-1.15977896549782	4.17072716079540
C	-5.60498999391164	0.34565533321422	2.35890314217907
C	-3.84166098524752	0.38412298492006	8.11343454700673
C	-1.93373831888226	1.84297122410819	8.30130602898437
C	-0.78647669266699	2.67929358297262	6.23385446194151
C	-5.72905885861479	-1.12832646413423	7.83418661205511
C	-6.60950045871697	-1.49915984324504	5.50270920745566
H	-7.28025086929766	-1.56172569526233	3.46306929072265
C	-5.58197725253842	1.74301741253965	2.22236357353603
C	-5.63021256026075	-0.44989036785515	1.21484102763564
C	-2.88898940160134	1.06371324322445	8.90565187366821
C	-4.84157168971266	-0.49622695023793	8.64923825135376
H	-1.16542038352516	2.33442693906908	8.88676922783853
C	-0.27178258778894	3.85799755330484	6.78098706140842
C	-0.16075206200793	2.13715970383942	5.09893352382322
H	-6.47542983132266	-1.80306674538271	8.24141947927584
H	-7.35640760845742	-2.19676113238225	5.87034272499837
C	-5.56100815379237	2.32632043410953	0.96956323993232
H	-5.61093075671352	2.37168508343917	3.10990974031311
C	-5.57150827526780	0.12611187267321	-0.05111427329122
H	-5.65808432366474	-1.53223305544149	1.30775969278902
H	-2.90490774232307	0.94348914331773	9.98504245706123
H	-4.86634099230391	-0.65120203668962	9.72336230953237
C	0.82976242228467	4.49839647803901	6.21799962108507

H	-0.74364346358651	4.29959332514975	7.65433332538198
C	0.94069002708584	2.75777775317498	4.53506117345380
H	-0.50250315432359	1.19138824165168	4.68152028461023
C	-5.51737224715187	1.51836869958637	-0.17436741494536
H	-5.55310254462316	3.40470257725261	0.84731640016555
H	-5.55437645865952	-0.51250074236908	-0.92615233458204
C	1.44847968872020	3.94111131747518	5.09380072092574
H	1.19487505594627	5.41642079656086	6.66265310639307
H	1.45646599990006	2.32859698820778	3.68226342664774
O	-5.42856974525127	2.17446212822767	-1.35262881709805
O	2.53338920955292	4.46075171066053	4.48301416287550
C	-5.25608576837376	1.40055545037836	-2.52982091874933
C	3.11593901445305	5.62946100077882	5.04472476257158
H	-6.12962598174723	0.76813457885153	-2.71987182400517
H	-5.13968239255678	2.11619031985146	-3.34261132963896
H	-4.35863365773066	0.77627695249411	-2.45615865114461
H	3.43569581115753	5.44668978529691	6.07572908937536
H	3.98075409749003	5.86009253626301	4.42482506668416
H	2.41153033788194	6.46803227097236	5.01988064034951
C	-0.36906899242206	0.38959810255501	-1.12352395071372
N	-0.07410613517956	0.38346235772196	-2.24025373327137

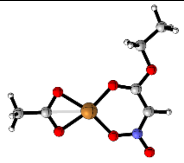	**Cu(OAc)-A**	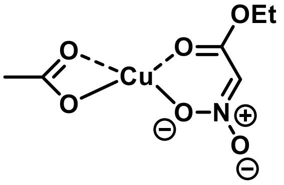

Charge: 0
Multiplicity: 2
[M06-2X/6-31G(d)]G_{298K}: -2379.93100460
[M06-2X/6-31G(d)]H_{298K}: -2379.87281681
[M06-2X/6-31G(d)]E_{SCF}: -2380.05536795
[M06-2X/6-31++G(d, p)]E_{SCF}: -2380.123501064145

O	-3.02908251463222	1.89023377376561	1.14903115686851
O	-3.26629852169126	2.79410708856474	-0.78803153535900
N	-2.93676301438736	2.92226345498387	0.37592048558206
C	-2.48248851593095	4.12063824096224	0.83287436820839
H	-2.43597649425391	4.89755500589198	0.08932795197276
C	-2.08401045475078	4.36710460987615	2.16011571030680
O	-2.08774494121498	3.57433302345376	3.14072150637237
O	-1.66003287694978	5.61209294304076	2.34321166777017
C	-1.20668588916778	5.96528000936235	3.66203391114073
H	-2.04863153097282	5.88717511352920	4.35564403100924
H	-0.44194831962150	5.25069863807809	3.97641150351993
C	-0.66777150556913	7.37597974087031	3.57175264292351
H	-0.30596391060860	7.69758773651398	4.55155326809042

H	0.16150858119271	7.42457557451866	2.86176524644963
H	-1.44736052418672	8.06751652457283	3.24368695134551
Cu	-2.61820447344395	1.75094082546920	3.00205206539155
O	-2.37901242853252	1.15970701107141	4.87166240473186
C	-2.76454955175836	0.00728610492698	4.49726217142826
O	-3.05489711685380	-0.15267708331545	3.27198828351827
C	-2.90352727252173	-1.11485539590112	5.48100546956707
H	-2.84366326782027	-2.07507343082179	4.96925959556307
H	-2.13772317060080	-1.02964110029805	6.25260393597930
H	-3.88301628572334	-1.03244840911598	5.96215120761934

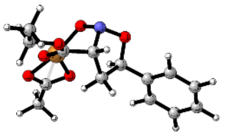	**Cu(OAc)-1a-IV**	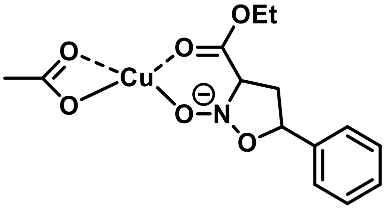

Charge: 0
Multiplicity: 2
[M06-2X/6-31G(d)]G_{298K}: -2689.31812182
[M06-2X/6-31G(d)]H_{298K}: -2689.24671707
[M06-2X/6-31G(d)]E_{SCF}: -2689.57769384
[M06-2X/6-31++G(d, p)]E_{SCF}: -2689.658880702762

O	-3.69934050618664	2.90863308128319	1.74483266835777
O	-2.70860967963961	2.57330904911440	-0.26768458700909
N	-2.90683682112773	3.50004426343922	0.78778024305977
C	-1.49735009148876	3.59330797242682	1.29213924338891
H	-0.96613112529320	4.25169839574055	0.60537556175991
C	-0.94619865427481	2.15331604159609	1.22188198368784
H	-0.91080654072457	1.63957346951504	2.18950622354532
H	0.06885393445917	2.14857610403172	0.82145898158994
C	-1.94877111420339	1.47853873222629	0.24632266377853
H	-2.60309826258148	0.79752594619323	0.79733358610706
C	-1.29389099000822	0.73884201391978	-0.88828969914304
C	-1.04734929949858	-0.62836139610977	-0.75944923815314
C	-0.89527485837977	1.40175719433732	-2.04958180612609
C	-0.39972657820745	-1.32665624079283	-1.77490765630482
H	-1.37547187209584	-1.14435606383909	0.14023183177192
C	-0.25696366087209	0.70135709328117	-3.06916275908162
H	-1.11120165178312	2.46026466479025	-2.15456310274601
C	-0.00519731770682	-0.66259752741735	-2.93391648365712
H	-0.21508749368898	-2.39102655115425	-1.66620940670639
H	0.04011753076109	1.22102914342353	-3.97511557861737
H	0.49057102101316	-1.20602204498192	-3.73224468991730
C	-1.48588730209915	4.20505997092779	2.66811859103680

O -1.87605627935660 3.61863120954420 3.67972298308706
O -1.02603868406273 5.42897221955640 2.70607491289124
C -1.04723291374102 6.09705730833575 3.99005410556998
H -2.05842193245020 6.01926043896082 4.39560572678038
H -0.36709699519554 5.56340523619522 4.65919149351520
C -0.62722173355934 7.52851553031063 3.74709324764024
H -0.62716850452066 8.07695447757814 4.69240972545082
H 0.37680426518916 7.57124123753344 3.31856598605771
H -1.32187638935258 8.01865866018942 3.06080552349603
Cu -3.03598287381482 2.01574511169468 3.22063210998543
O -2.85115590062201 0.97920229095540 4.87779024065451
C -2.88269622003540 -0.09454827260923 4.19331264617453
O -3.02284052250564 -0.00468423967531 2.93866095801665
C -2.77667810439239 -1.43235065123565 4.86380254445608
H -2.17124295273528 -2.10319610617811 4.25249027936527
H -2.35417679747166 -1.32478726550446 5.86234392991773
H -3.77942912774629 -1.86251349760283 4.94309001631909

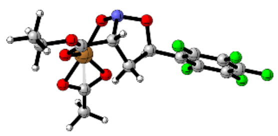	**Cu(OAc)-1j-IV**	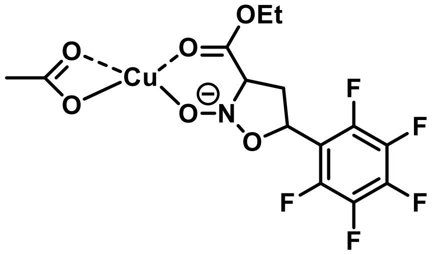

Charge: 0
Multiplicity: 2
[M06-2X/6-31G(d)]G_{298K}: -3185.35751613
[M06-2X/6-31G(d)]H_{298K}: -3185.27651937
[M06-2X/6-31G(d)]E_{SCF}: -3185.57067051
[M06-2X/6-31++G(d, p)]E_{SCF}: -3185.676579893844

O -3.02908251463222 1.89023377376561 1.14903115686851
O -3.26629852169126 2.79410708856474 -0.78803153535900
N -2.93676301438736 2.92226345498387 0.37592048558206
C -2.48248851593095 4.12063824096224 0.83287436820839
H -2.43597649425391 4.89755500589198 0.08932795197276
C -2.08401045475078 4.36710460987615 2.16011571030680
O -2.08774494121498 3.57433302345376 3.14072150637237
O -1.66003287694978 5.61209294304076 2.34321166777017
C -1.20668588916778 5.96528000936235 3.66203391114073
H -2.04863153097282 5.88717511352920 4.35564403100924
H -0.44194831962150 5.25069863807809 3.97641150351993
C -0.66777150556913 7.37597974087031 3.57175264292351
H -0.30596391060860 7.69758773651398 4.55155326809042
H 0.16150858119271 7.42457557451866 2.86176524644963
H -1.44736052418672 8.06751652457283 3.24368695134551

Cu	-2.61820447344395	1.75094082546920	3.00205206539155
O	-2.37901242853252	1.15970701107141	4.87166240473186
C	-2.76454955175836	0.00728610492698	4.49726217142826
O	-3.05489711685380	-0.15267708331545	3.27198828351827
C	-2.90352727252173	-1.11485539590112	5.48100546956707
H	-2.84366326782027	-2.07507343082179	4.96925959556307
H	-2.13772317060080	-1.02964110029805	6.25260393597930
H	-3.88301628572334	-1.03244840911598	5.96215120761934

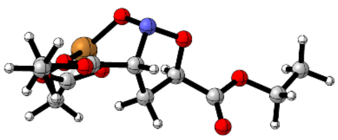	**Cu(OAc)-1aa-IV**	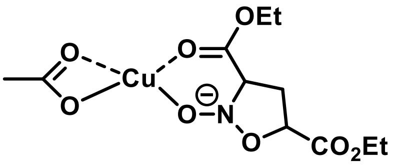

Charge: 0
Multiplicity: 2
[M06-2X/6-31G(d)]G_{298K}: -2725.45605533
[M06-2X/6-31G(d)]H_{298K}: -2725.38168953
[M06-2X/6-31G(d)]E_{SCF}: -2725.70379791
[M06-2X/6-31++G(d, p)]E_{SCF}: -2725.786764807132

O	-3.02908251463222	1.89023377376561	1.14903115686851
O	-3.26629852169126	2.79410708856474	-0.78803153535900
N	-2.93676301438736	2.92226345498387	0.37592048558206
C	-2.48248851593095	4.12063824096224	0.83287436820839
H	-2.43597649425391	4.89755500589198	0.08932795197276
C	-2.08401045475078	4.36710460987615	2.16011571030680
O	-2.08774494121498	3.57433302345376	3.14072150637237
O	-1.66003287694978	5.61209294304076	2.34321166777017
C	-1.20668588916778	5.96528000936235	3.66203391114073
H	-2.04863153097282	5.88717511352920	4.35564403100924
H	-0.44194831962150	5.25069863807809	3.97641150351993
C	-0.66777150556913	7.37597974087031	3.57175264292351
H	-0.30596391060860	7.69758773651398	4.55155326809042
H	0.16150858119271	7.42457557451866	2.86176524644963
H	-1.44736052418672	8.06751652457283	3.24368695134551
Cu	-2.61820447344395	1.75094082546920	3.00205206539155
O	-2.37901242853252	1.15970701107141	4.87166240473186
C	-2.76454955175836	0.00728610492698	4.49726217142826
O	-3.05489711685380	-0.15267708331545	3.27198828351827
C	-2.90352727252173	-1.11485539590112	5.48100546956707
H	-2.84366326782027	-2.07507343082179	4.96925959556307
H	-2.13772317060080	-1.02964110029805	6.25260393597930
H	-3.88301628572334	-1.03244840911598	5.96215120761934

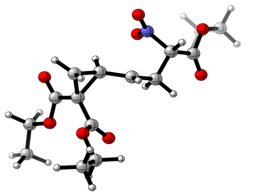	**1ah-I**	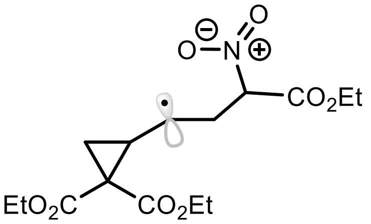

Charge: 0
Multiplicity: 2
[CPCM(CHCl3)M06-2X/6-31G(d)]G_{298K}: -1240.42095237
[CPCM(CHCl3)M06-2X/6-31G(d)]H_{298K}: -1240.33975923
[CPCM(CHCl3)M06-2X/6-31G(d)]E_{SCF}: -1240.74407122
[(CPCM(CHCl3)M06-2X/6-31++G(d, p)]E_{SCF}: -1240.81135527

O	-1.79939690442090	-0.90073329256419	0.05736683234509
O	-1.04330372376227	0.36290056792994	1.63896819905680
N	-1.60986894452260	0.18300290707226	0.58157744226409
C	-2.03557131483567	1.39215800684616	-0.20033185053751
H	-2.05815421531352	2.21592705216344	0.51505661554536
C	-0.99733282363277	1.61748926626215	-1.31367391663980
H	-1.32798701466891	2.50512955543214	-1.86131373701831
H	-1.05835693995650	0.76417011515704	-1.99816762418358
C	0.38682892622872	1.78693249576992	-0.78356598521847
H	0.71799898539303	2.76475642795136	-0.45137351870919
C	-3.42971779238521	1.11985209018147	-0.75628106211486
O	-3.72616878752530	1.27310523517884	-1.91435002821617
O	-4.24649979404846	0.71786797589445	0.20959236907353
C	-5.56575281471951	0.28849759702941	-0.20031525588333
H	-6.15525011430767	0.32050323045939	0.71575115345318
H	-5.96145139684185	1.01312731524466	-0.91435129258604
C	-5.50144873586309	-1.10936305474494	-0.78391983640843
H	-6.51077219264933	-1.45106950806601	-1.02882995838183
H	-4.90063102061165	-1.11930750016960	-1.69687968555668
H	-5.06195423476496	-1.80267361937273	-0.06211561312309
C	1.27211917418376	0.63561157726330	-0.61883709701799
C	2.69815395025033	0.63990761505697	-1.26922882367701
C	2.49570004266789	0.69869061655350	0.22528892240049
H	0.81216642008521	-0.34589030559871	-0.71911247869688
H	2.77522458472758	-0.19754629313800	0.76797399387803
H	2.72619317056777	1.64439141806323	0.70246422088565
C	3.12603437996282	-0.69504639482488	-1.78774244891318
O	2.70344313883223	-1.73829572098083	-1.33413210224056
O	4.04486506792634	-0.62491925018126	-2.74582816788221
C	3.03110614840602	1.87070171851125	-2.05205925995822
O	3.05733092143959	1.92907343560253	-3.25932502333372
O	3.22037463210812	2.93198199117095	-1.26011318100500

C	4.50985521319831	-1.88892172686097	-3.25924310174563
C	5.45406278432182	-1.58576618490102	-4.40126889995866
H	3.64449039165527	-2.47250076783443	-3.58323161633271
H	5.00365976369095	-2.43140451659454	-2.44819889366090
H	5.83522709848174	-2.52163251393920	-4.81831719909642
H	4.93691425179629	-1.03622916370885	-5.19193245400800
H	6.30157533237873	-0.98883512082989	-4.05456615057654
C	3.36832797894462	4.19488627198298	-1.93483935603522
C	3.48308334322138	5.25924997714966	-0.86633680203514
H	2.49712464216563	4.34733035863801	-2.57854677236176
H	4.25771630191941	4.15017825428405	-2.56889961328417
H	3.60337585004117	6.23990930053100	-1.33382978590859
H	2.58429863808509	5.27856124530071	-0.24364529965485
H	4.34909241215029	5.06991680562922	-0.22671974694151

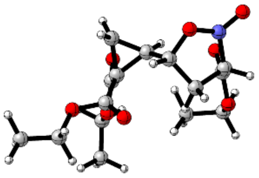	**1ah-III**	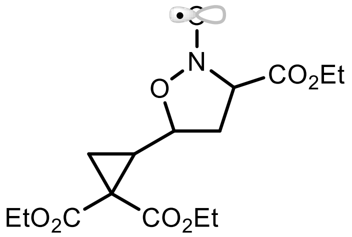

Charge: 0
Multiplicity: 2
[CPCM(CHCl3)M06-2X/6-31G(d)]G_{298K}: -1240.45375827
[CPCM(CHCl3)M06-2X/6-31G(d)]H_{298K}: -1240.37595075
[CPCM(CHCl3)M06-2X/6-31G(d)]E_{SCF}: -1240.78209632
[(CPCM(CHCl3)M06-2X/6-31++G(d, p)]E_{SCF}: -1240.84835872

C	-0.50982934423719	-0.92134561321970	1.76599144120010
C	0.79959679830331	-1.00532860788150	0.97159382501941
C	0.88036292842826	0.38126743689209	0.32697885333999
H	-0.48263429107198	-1.53698964856403	2.66478067182792
H	0.78786246109785	-1.81271389765173	0.23942327468132
H	1.63936373832868	-1.15226854824462	1.65247746999134
H	1.88222276333307	0.81681755078138	0.35992252344829
O	-0.54694148132376	0.43351424144106	2.22256745974531
O	0.21160873370925	2.40212822802425	1.42101922658424
N	-0.01536663087018	1.21322874885885	1.16058131721758
C	0.36910974625694	0.39238438543950	-1.10778875678865
O	-0.62409606976574	0.96524457409145	-1.48533347230078
O	1.16434320480341	-0.35088705764104	-1.87245203856013
C	0.71375162802912	-0.58351783111197	-3.22458239789657
H	1.60399822601639	-0.92898632207802	-3.75024699842387
H	0.38105116829150	0.36443906286363	-3.65203278169130
C	-0.39030581378754	-1.62440940998024	-3.23606826668423
H	-1.28781408168676	-1.24180209126061	-2.74001572542945

H	-0.64778304792237	-1.87657682265994	-4.26913489629778
H	-0.06161405621501	-2.53590411165027	-2.72578562337281
C	-1.74279535862640	-1.20932766358438	0.93524495540021
C	-2.16126429028148	-2.63376975804063	0.62702339270922
C	-2.97708932338820	-1.75689544979275	1.55593352835834
H	-1.89759446904937	-0.52026521715825	0.10606306639365
H	-3.93769974192443	-1.42889429879094	1.17489856945179
H	-2.93980493086943	-2.01782504777452	2.60985769790268
C	-2.72175372025719	-2.82856151716895	-0.74719133223223
O	-3.53296638309085	-2.08477996761958	-1.25171568897208
O	-2.18359373303462	-3.88130310806907	-1.36172666188754
C	-1.43279572401485	-3.80061191162462	1.23259830021589
O	-0.28326984279437	-3.78810998287883	1.61381173275739
O	-2.23357133950440	-4.85882522583527	1.33713458516704
C	-2.66416778445492	-4.15571305801265	-2.69318419826458
C	-1.81679992962154	-5.27844284006858	-3.24809234018045
H	-2.58278497379383	-3.24348020730550	-3.28921955562283
H	-3.72115134359269	-4.42848305841645	-2.62623959176268
H	-2.15467456264766	-5.52943017946916	-4.25675072542337
H	-0.76644600395390	-4.97816254491587	-3.29816661292266
H	-1.89685869704383	-6.17021284726837	-2.62089920115578
C	-1.63529413632070	-6.04288912980237	1.90096026999036
C	-2.72413211894347	-7.08680204014299	2.00465575543585
H	-1.21569071568647	-5.78756678608918	2.87752592595800
H	-0.81674921066502	-6.36040082841700	1.24870235387492
H	-2.31048476923389	-8.00813460050868	2.42260719364173
H	-3.52894033319274	-6.73803407025915	2.65692078481706
H	-3.14159213166202	-7.30762372032908	1.01880597790447

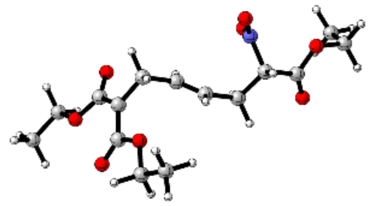

1ah-VII

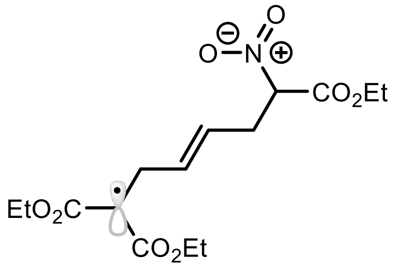

Charge: 0
Multiplicity: 2
[CPCM(CHCl3)M06-2X/6-31G(d)]G_{298K}: -1240.42831243
[CPCM(CHCl3)M06-2X/6-31G(d)]H_{298K}: -1240.34599437
[CPCM(CHCl3)M06-2X/6-31G(d)]E_{SCF}: -1240.75146108
[(CPCM(CHCl3)M06-2X/6-31++G(d, p)]E_{SCF}: -1240.81923899

O	-2.04050389730631	-0.86843545739605	0.39311672522601
O	-1.52128805649363	0.50529118322989	1.97768456892030
N	-1.92300705696152	0.24850824025217	0.86273038482618
C	-2.25824145424202	1.39484140830032	-0.05024038156201

H	-2.21938947041168	2.28889714448087	0.57561345827376
C	-1.23157529007308	1.42244037934422	-1.18183567784103
H	-1.48821311765126	2.28032454889883	-1.81146686437172
H	-1.35820844635787	0.51738548523642	-1.78277243885882
C	0.17267645351626	1.52575156478754	-0.66063650883596
H	0.46177755957090	2.46122860085313	-0.18091385143663
C	-3.67738517242850	1.17595766072541	-0.56952632445229
O	-3.98896566483105	1.31974641308704	-1.72483679402144
O	-4.49370442145002	0.83773969300500	0.42068703133740
C	-5.85104090772812	0.49851723782506	0.05529936428351
H	-6.40820376908845	0.58873950617718	0.98761610647755
H	-6.21424315771044	1.23857496397021	-0.66020515792466
C	-5.90816758572382	-0.90978287895151	-0.50359207610182
H	-6.94746271413805	-1.18076176628409	-0.70812503847141
H	-5.34148499909938	-0.97733211356260	-1.43555780133532
H	-5.49544836539633	-1.62132501489330	0.21606368219940
C	1.04489208020234	0.52110854658738	-0.73429975515527
C	3.38689092922096	0.62496789196052	-1.40339097461691
C	2.46755004303148	0.59860636832001	-0.22306693691230
H	0.76280724272414	-0.41455259688135	-1.21713383201344
H	2.68838515581130	-0.28505342370462	0.38149641616570
H	2.60076569568552	1.49750222448412	0.38081485498751
C	3.75631376138961	-0.68831809581533	-1.97102679857795
O	3.22729803310962	-1.71350054281728	-1.57879935265893
O	4.69541346912106	-0.65967073940472	-2.91216949694990
C	3.72156245636444	1.90138763287246	-2.06572074302714
O	4.37118089468967	2.02694303244268	-3.08306033389155
O	3.20178686695157	2.94375896222706	-1.39886669826249
C	5.03707922213972	-1.93836403581666	-3.47945769200329
C	6.10079210918733	-1.69045561464769	-4.52542485070587
H	4.13267810822865	-2.38066713440721	-3.90671681012281
H	5.38885101502260	-2.59400486856090	-2.67783456924675
H	6.38613002944873	-2.63767747911453	-4.99053006240149
H	5.72651785472947	-1.01834161287173	-5.30172491590659
H	6.98934528443972	-1.24304077159555	-4.07281207681464
C	3.45933379635671	4.23586430575416	-1.97430375462782
C	2.83263423735862	5.26452657215725	-1.05945065023384
H	3.02948995219927	4.26195304846619	-2.97979083626589
H	4.54088141052204	4.36894470275654	-2.06449852074624
H	3.00823298514209	6.26685226373589	-1.45871242141130
H	1.75353381918786	5.10614451990756	-0.98163024901931
H	3.26972544506512	5.20602940702661	-0.05920249049305

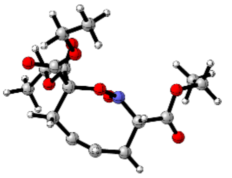	**1ah-VIII**	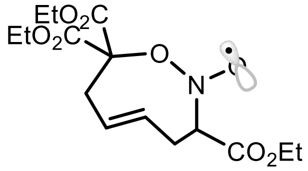

Charge: 0
Multiplicity: 2
[CPCM(CHCl3)M06-2X/6-31G(d)]G298K: -1240.42033345
[CPCM(CHCl3)M06-2X/6-31G(d)]H298K: -1240.34486074
[CPCM(CHCl3)M06-2X/6-31G(d)]ESCF: -1240.74925385
[(CPCM(CHCl3)M06-2X/6-31++G(d, p)]ESCF: -1240.81329360

O	-0.84507968747580	-0.59755353014795	0.37785457203627
O	0.01501079604287	1.34249723494892	1.27887454089395
N	-1.01119964637764	0.61100352647556	0.62200914406732
C	-1.76130510468229	1.44523691424671	-0.34384068904839
H	-1.76657899004505	2.45489882692186	0.07709683806223
C	-1.09764630750509	1.43056431053311	-1.74644241703656
H	-1.61621236216657	2.16429795694978	-2.36890599738878
H	-1.22697808723865	0.43597119748727	-2.18327384797643
C	0.33671359269484	1.73499226579509	-1.45884745903047
H	0.55442984275983	2.75499097676213	-1.13844313376494
C	-3.20130744168039	0.93500487269759	-0.38879644402864
O	-3.81583186806673	0.79830837087856	-1.41836685180876
O	-3.68471433232526	0.71459755022706	0.82768538874950
C	-5.04418061984395	0.22776482412729	0.90200069398178
H	-5.34678794823718	0.44599359231797	1.92625654203681
H	-5.65825187361009	0.80697199080128	0.20930355105466
C	-5.10101740453401	-1.25763596338275	0.60326541023894
H	-6.12013324055332	-1.62301475690685	0.75720543816832
H	-4.81377446084532	-1.45530666122862	-0.43209626706870
H	-4.42946260323187	-1.80439970264175	1.27064670445866
C	1.18757389213153	0.76295092032676	-1.12454891362445
C	1.31960639341323	0.75717827973563	1.31497784537245
C	2.13761325060022	1.00319332890079	0.00252377991733
H	0.95925237161676	-0.27096365806745	-1.37774608604311
H	2.46116176113933	2.04931511982112	0.01149212110203
H	3.02051841702874	0.36294696546903	0.02401676065995
C	2.07300945287387	1.43648546215565	2.46954280810559
O	3.24038964762141	1.18492312148550	2.66024570851508
O	1.34506357055004	2.27560526178261	3.18105242127536
C	1.23637793857316	-0.71814975263074	1.76957862748348
O	0.74626154371664	-1.01967157621310	2.83057763109460
O	1.81645823199537	-1.55666586708345	0.92603954171335
C	2.03042449578368	2.92879755987667	4.27484228442177
C	1.08536597185094	3.96895452724248	4.83187933920455
H	2.95332613915181	3.36896690674963	3.88991792290816

H 2.29131386225243 2.16571946028820 5.01299628310951
H 1.56495142446533 4.48570079434191 5.66700054473741
H 0.83252158717221 4.70639784429293 4.06557484474470
H 0.16457287191732 3.50317216565676 5.19173882159498
C 1.73289797366261 -2.95147616334953 1.28631353913639
C 2.48051288290723 -3.73159171620681 0.22926872053221
H 0.67415252574932 -3.22255652955418 1.33223260250209
H 2.16730438791148 -3.07842824366177 2.28090277046289
H 2.43698325256188 -4.79831456059572 0.46392006250959
H 2.03434578702329 -3.57319139343247 -0.75597959340218
H 3.52939129700526 -3.42571897229254 0.19548761724435

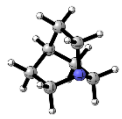	**Qui**	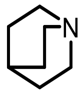

Charge: 0
Multiplicity: 1
[CPCM(CHCl3)M06-2X/6-31+G(d,p)]G_{298K}: -329.01038803
[CPCM(CHCl3)M06-2X/6-31+G(d,p)]H_{298K}: -328.97247544
[CPCM(CHCl3)M06-2X/6-31+G(d,p)]E_{SCF}: -329.17688669

C -1.06752995047803 0.62015347855006 -0.24403119551049
C -0.88375557274883 3.04895688064121 0.32169117553488
C -1.87945125518760 1.90322235968592 0.08899032535192
H -1.23538844817660 0.30848758362049 -1.28060458359972
H -1.36532094468132 -0.21301232729832 0.40117339383850
H -2.56021549555035 2.15632005512596 -0.73124817727715
H -2.49026995458842 1.75672821303616 0.98729654288060
C 0.83204000668342 1.84390388573799 -1.04165927565910
H 1.89846959259421 2.01559081996720 -0.86096180072981
H 0.73319381555427 1.42052144741242 -2.04658892519066
C 0.02460522428061 3.16608122672989 -0.91104343011585
H 0.69713455409689 4.02445697077944 -0.80519245110084
H -0.58800239078070 3.33812390488015 -1.80355339174297
H -1.41856895227612 3.98903429808453 0.48966906450137
C -0.01846485034253 2.70259406022198 1.54216868492486
H 0.74452227338198 3.47609315103977 1.68753132418630
H -0.63622305102964 2.67909896296071 2.44669077326225
C 0.63954330357596 1.31742236776917 1.28802279181853
H 1.72453803298510 1.36654330149620 1.42624855272380
H 0.25339437652036 0.56620677518996 1.98514355162136
N 0.37754868616732 0.83267258436907 -0.07555594971778

Charge: 0
Multiplicity: 1
[CPCM(CHCl3)M06-2X/6-31G(d)]G_{298K}: -328.98864695

[CPCM(CHCl3)M06-2X/6-31G(d)]H_{298K}: -328.95047494
[CPCM(CHCl3)M06-2X/6-31G(d)]E_{SCF}: -329.15637667
[(CPCM(CHCl3)M06-2X/6-31++G(d, p)]E_{SCF}: -329.17695228

C -1.06844768978032 0.60771469681156 -0.19521548623777
C -0.88493499100158 3.04802287575133 0.32113138199365
C -1.87944711809403 1.91308701686493 0.03932738142604
H -1.26053689816179 0.20004593985728 -1.19352523904659
H -1.34591099767779 -0.16227301350778 0.53267525568276
H -2.48664738484573 2.15875460197055 -0.83907749715765
H -2.56422390387433 1.79988029123886 0.88729539022378
C 0.79506941001725 1.82725557545008 -1.07169571080851
H 1.87954140266745 1.95112189635334 -0.98172317369819
H 0.59456112780859 1.41011365962635 -2.06441399999671
C 0.06105735957055 3.18459694318727 -0.87941605277093
H 0.77799093959001 3.99334163455106 -0.69834249220850
H -0.51084169657060 3.45052811927143 -1.77555082128143
H -1.42051146713105 3.98780084144427 0.48937397468965
C -0.05754095626295 2.67944967508889 1.56016220020597
H 0.65962378368827 3.47819101054573 1.78054021842192
H -0.71440448363998 2.58198165618798 2.43192215924103
C 0.67795351589273 1.34176129547237 1.26775410030119
H 1.76356520010978 1.46699723982216 1.34140393123734
H 0.38800319572061 0.56906552863584 1.98798268649763
N 0.37788065197489 0.83176251537642 -0.07642120671470

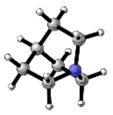	**Qui·+**	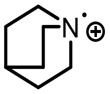

Charge: 1
Multiplicity: 2
[CPCM(CHCl3)M06-2X/6-31+G(d,p)]G_{298K}: -328.79432265
[CPCM(CHCl3)M06-2X/6-31+G(d,p)]H_{298K}: -328.75584450
[CPCM(CHCl3)M06-2X/6-31+G(d,p)]E_{SCF}: -328.96023213

C -1.07489715275125 0.60738608482352 -0.27932877324712
C -0.88568656996366 3.05132558016905 0.32257665480942
C -1.88841375055360 1.91125003113379 0.11602968374279
H -1.20840460304726 0.35617018711657 -1.33176353765859
H -1.34424407981048 -0.23509133402385 0.35937105030926
H -2.58859445334081 2.13436818247826 -0.69179824425458
H -2.45360679376137 1.71743992817676 1.03069848397795
C 0.86972567380567 1.85206418845603 -1.03847561337212
H 1.91516471057801 2.04022209763091 -0.79285379028518
H 0.77729551787589 1.40333675297100 -2.02842455072076
C -0.00300696682825 3.17321525476267 -0.92413274033663

H	0.67562779680977	4.02604724509233	-0.85552907782418
H	-0.61331292506877	3.27594055019696	-1.82449709117084
H	-1.42117516028171	3.99111554688417	0.49126929530052
C	-0.00467930934937	2.73031280579063	1.53497659837700
H	0.80024202135816	3.46164893163824	1.63872381222428
H	-0.58650205800270	2.70949845504972	2.45891991790726
C	0.62707010941685	1.29000887981884	1.31538116475399
H	1.71031719894349	1.31198213521040	1.44124939243494
H	0.17276919683246	0.54967518187027	1.97425516232895
N	0.30611059713891	0.96128331475366	-0.05246079729638

Charge: 1
Multiplicity: 2
[CPCM(CHCl3)M06-2X/6-31G(d)]G_{298K}: -328.77510019
[CPCM(CHCl3)M06-2X/6-31G(d)]H_{298K}: -328.73828394
[CPCM(CHCl3)M06-2X/6-31G(d)]E_{SCF}: -328.94314576
[(CPCM(CHCl3)M06-2X/6-31++G(d, p)]E_{SCF}: -328.96023300

C	-1.08111726033567	0.58247020453805	-0.18835030191344
C	-0.88646044601482	3.05070932717171	0.32207144143497
C	-1.88961029469872	1.93077846053521	0.03138100800355
H	-1.24457691235070	0.17670353953298	-1.18726521827955
H	-1.32404958669433	-0.15869211803754	0.57375141709242
H	-2.46806353883809	2.14318989083780	-0.87035729326024
H	-2.57849130843606	1.79001885662553	0.86713283047210
C	0.80320075355087	1.82020536290875	-1.09884979050040
H	1.88341839333166	1.92441646127468	-0.99283700325172
H	0.55033875966399	1.38766043956767	-2.06729945756675
C	0.06062902459405	3.20513726664786	-0.87089246958300
H	0.81025426299843	3.97672753850612	-0.68196433013886
H	-0.48772707209893	3.46001120179753	-1.78043424247610
H	-1.42219929842492	3.99074318417461	0.48959876973437
C	-0.07421763833243	2.69085277946321	1.56921854565036
H	0.65661581758027	3.46833102769727	1.80229618031817
H	-0.72391034253995	2.54492197834336	2.43507881599400
C	0.70318239983418	1.33716183026715	1.28161556634831
H	1.78339142958674	1.48454405672932	1.30544459633380
H	0.40648493199962	0.55182846285977	1.97761343597985
N	0.30470692562481	0.96148024855884	-0.05276550039187

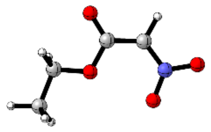	**A** (ENA-)	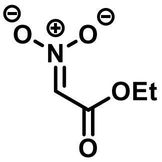

Charge: -1
Multiplicity: 1

[CPCM(CHCl3)M06-2X/6-31+G(d,p)]G_{298K}: -511.46309613
[CPCM(CHCl3)M06-2X/6-31+G(d,p)]H_{298K}: -511.41887807
[CPCM(CHCl3)M06-2X/6-31+G(d,p)]E_{SCF}: -511.53875397

C	-1.19772648092451	-0.57752990429314	-0.74898990899803
H	-1.47970714700916	-1.37903757844891	-0.08600487015810
N	-0.88206379181394	-0.96784062555540	-2.00675882784615
O	-0.95228357810291	-2.20317259566904	-2.27815931167049
O	-0.52605045186569	-0.16020101327407	-2.89888921416545
C	-1.19525632193676	0.75323317502504	-0.21663758189252
O	-1.49189462316920	0.97940494701171	0.96051487856116
O	-0.85728174166053	1.75697509633082	-1.05119300768423
C	-0.87232394026292	3.07199936102480	-0.48282479338914
H	-1.86932503474252	3.27951997485623	-0.08255146036308
H	-0.16073015832226	3.11423169429049	0.34769723561883
C	-0.50120332153672	4.03937638813703	-1.58776520631154
H	-1.21996865962068	3.97872182946271	-2.40924413687459
H	-0.50127070134678	5.06147250377729	-1.19917801243870
H	0.49462333231460	3.81587828732441	-1.97968617238795

Charge: -1
Multiplicity: 1
[CPCM(CHCl3)M06-2X/6-31G(d)]G298K: -511.41675245
[CPCM(CHCl3)M06-2X/6-31G(d)]H298K: -511.37278141
[CPCM(CHCl3)M06-2X/6-31G(d)]ESCF: -511.49363273
[(CPCM(CHCl3)M06-2X/6-31++G(d, p)]ESCF: -511.53864133

C	-1.20279138699979	-0.57135719574150	-0.74841254980117
H	-1.48470232496938	-1.37390656540203	-0.08714306109035
N	-0.87861128668979	-0.96835786681975	-2.01019289953226
O	-0.94477643444193	-2.19776785512223	-2.27120407481808
O	-0.52404000889171	-0.16030733675042	-2.89485055606981
C	-1.19954740682885	0.75309512723375	-0.21642872849120
O	-1.49776025722138	0.99301395169884	0.95441250949520
O	-0.85249029847693	1.75669404795317	-1.06069016948441
C	-0.86846052061973	3.06188853504372	-0.48528500139865
H	-1.86202201036951	3.27055319886436	-0.07600654707366
H	-0.15642919824315	3.10678548848647	0.34553791371466
C	-0.50356567617355	4.03849972429887	-1.58533156473351
H	-1.22589389017970	3.97909989856583	-2.40407823963209
H	-0.50087895343966	5.06015212970224	-1.19508053232090
H	0.48950703354506	3.81494625798866	-1.98491688876376

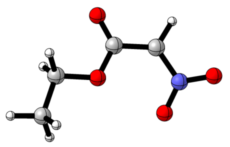	**R** (ENA·)	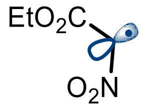

Charge: 0
Multiplicity: 2
[CPCM(CHCl3)M06-2X/6-31+G(d,p)]G_{298K}: -511.27237553
[CPCM(CHCl3)M06-2X/6-31+G(d,p)]H_{298K}: -511.22705281
[CPCM(CHCl3)M06-2X/6-31+G(d,p)]E_{SCF}: -511.34706535

C	-1.19772648092451	-0.57752990429314	-0.74898990899803
H	-1.47970714700916	-1.37903757844891	-0.08600487015810
N	-0.88206379181394	-0.96784062555540	-2.00675882784615
O	-0.95228357810291	-2.20317259566904	-2.27815931167049
O	-0.52605045186569	-0.16020101327407	-2.89888921416545
C	-1.19525632193676	0.75323317502504	-0.21663758189252
O	-1.49189462316920	0.97940494701171	0.96051487856116
O	-0.85728174166053	1.75697509633082	-1.05119300768423
C	-0.87232394026292	3.07199936102480	-0.48282479338914
H	-1.86932503474252	3.27951997485623	-0.08255146036308
H	-0.16073015832226	3.11423169429049	0.34769723561883
C	-0.50120332153672	4.03937638813703	-1.58776520631154
H	-1.21996865962068	3.97872182946271	-2.40924413687459
H	-0.50127070134678	5.06147250377729	-1.19917801243870
H	0.49462333231460	3.81587828732441	-1.97968617238795

Charge: 0
Multiplicity: 2
[CPCM(CHCl3)M06-2X/6-31G(d)]G_{298K}: -511.24208974
[CPCM(CHCl3)M06-2X/6-31G(d)]H_{298K}: -511.19710637
[CPCM(CHCl3)M06-2X/6-31G(d)]E_{SCF}: -511.31793014
[(CPCM(CHCl3)M06-2X/6-31++G(d, p)]E_{SCF}: -511.34685712

C	-1.18214913338143	-0.59548111059993	-0.70793732100372
H	-1.50123838787758	-1.41005424384541	-0.07460283860340
N	-0.83525514279353	-0.97952221051940	-2.03933328460632
O	-1.28881303237809	-2.05524714243533	-2.41046276308863
O	-0.11758007777836	-0.25546805533593	-2.70751077344860
C	-1.14987982809904	0.77551142525185	-0.17403534054979
O	-1.33981307348081	0.94316709883531	1.01356949243127
O	-0.93715246202025	1.73236407796983	-1.06086279678160
C	-0.93113597652583	3.07778261927039	-0.53025849211020
H	-1.90872897139125	3.27075223330278	-0.08038026845240
H	-0.17187339265807	3.13490560112495	0.25385851376472
C	-0.63407758596701	4.00671713928132	-1.68499156779746
H	-0.62905347465878	5.03946536395366	-1.32757651854315

H	0.34444602377256	3.78270357016409	-2.11712068532110
H	-1.39513990476252	3.91023008358182	-2.46318254588960

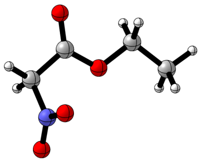	Ethyl Nitro Acetate, **2a**	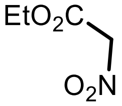

Charge: 0
Multiplicity: 1
[CPCM(CHCl3)M06-2X/6-31G(d)]G_{298K}: -511.89445602
[CPCM(CHCl3)M06-2X/6-31G(d)]H_{298K}: -511.84916084
[CPCM(CHCl3)M06-2X/6-31G(d)]E_{SCF}: -511.98399400
[(CPCM(CHCl3)M06-2X/6-31++G(d, p)]E_{SCF}: -512.01446384

N	-0.63312711420029	-0.78867120088306	-1.95250294736773
O	-1.29920209640318	-1.07693852731213	-2.92497274441232
O	0.57021895413788	-0.61353413667308	-1.94655292100008
C	-1.09217228841363	0.74110220626874	-0.06068408903314
O	-1.01220918372827	0.94496907745075	1.12400913913959
O	-1.04686683021803	1.66507566412609	-1.00956939541177
C	-0.86112408895799	3.02647788771085	-0.55848954699349
H	-1.69328225565839	3.28166274109202	0.10261178427947
H	0.06626037210556	3.07102042386092	0.01799999463440
C	-0.81677814927834	3.90226096328539	-1.78923985138621
H	-0.68228484455571	4.94456155582720	-1.48856994688128
H	0.01611519655701	3.61633152251336	-2.43656712686550
H	-1.74822587331092	3.82057706923051	-2.35499582560607
C	-1.33117608177740	-0.64331834363761	-0.64083528597064
H	-2.39417757675593	-0.81119156621920	-0.81306885785695
H	-0.91470210954235	-1.39660457664079	0.02730194073175

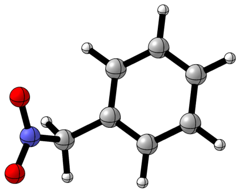	Phenyl Nitro Methane, **2b**	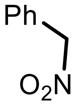

Charge: 0
Multiplicity: 1
[CPCM(CHCl3)M06-2X/6-31G(d)]G_{298K}: -475.75808160
[CPCM(CHCl3)M06-2X/6-31G(d)]H_{298K}: -475.71754133
[CPCM(CHCl3)M06-2X/6-31G(d)]E_{SCF}: -475.86016340
[(CPCM(CHCl3)M06-2X/6-31++G(d, p)]E_{SCF}: -475.88900499

N	-0.51589974701861	-0.82117392953732	-1.85322193360251
O	-1.05303273129640	-1.03669003209396	-2.92174045491975
O	0.68547404575483	-0.78096921567831	-1.67093037843951

C	-1.40271085322115	-0.59027780796053	-0.65434097861257
H	-2.42154360031347	-0.70314608309263	-1.02048666402767
H	-1.14663766073436	-1.39694972840183	0.03583768904158
C	-1.13766241319449	0.76720304460916	-0.06556214764210
C	-1.92664675241956	1.85404929525668	-0.44641937891567
C	-0.09503356485827	0.95031328646808	0.84531572720322
C	-1.67758265030073	3.11761488005261	0.08430912328629
H	-2.73518748702403	1.70806263590351	-1.15805092725242
C	0.15363345116339	2.21335253312156	1.37507387912721
H	0.51599302227619	0.10116001312481	1.13764783068026
C	-0.63802100436221	3.29697755004465	0.99522691612541
H	-2.29460269530498	3.96031304024730	-0.21095892337864
H	0.95890749371416	2.35129997787684	2.08965879375392
H	-0.44817212286031	4.28091432005937	1.41301278757294

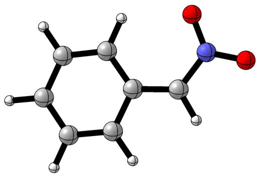	Phenyl Nitro Radical	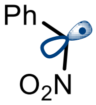

Charge: 0
Multiplicity: 2
[CPCM(CHCl3)M06-2X/6-31G(d)]G_{298K}: -475.12587773
[CPCM(CHCl3)M06-2X/6-31G(d)]H_{298K}: -475.08350838
[CPCM(CHCl3)M06-2X/6-31G(d)]E_{SCF}: -475.21325058
[(CPCM(CHCl3)M06-2X/6-31++G(d, p)]E_{SCF}: -475.24166442

C	-2.92931576351275	-0.88931034384511	-0.07022392453025
C	-1.55701432983827	-0.77950580488169	0.04784843463694
C	-0.93076033409162	0.49415007548230	0.13504662316737
C	-1.74820530894439	1.65549938312751	0.09539246261212
C	-3.12123444732126	1.52613707253021	-0.02519244878523
C	-3.71908376229957	0.26460751922769	-0.10685140334496
H	-3.39085726923926	-1.86887632911695	-0.13648569397282
H	-0.93840046390687	-1.67188323948983	0.07435702903154
H	-1.29386649892396	2.63317638949383	0.16167761569491
H	-3.73790913667522	2.41862539240036	-0.05352673735159
H	-4.79713116455182	0.18062516962461	-0.19947895075685
C	0.47651034096541	0.46278817624476	0.25648282369839
H	1.02117659663404	-0.47121171155854	0.27629486028702
N	1.33780005592229	1.57075522921014	0.36655977978483
O	2.54171302910222	1.31504309149490	0.46535600991880
O	0.89059248668108	2.71857211005578	0.36225620990979

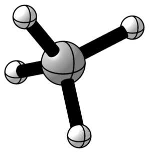	Methane	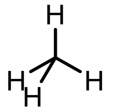

Charge: 0
Multiplicity: 1
[CPCM(CHCl3)M06-2X/6-31G(d)]G_{298K}: -40.45622526
[CPCM(CHCl3)M06-2X/6-31G(d)]H_{298K}: -40.43274979
[CPCM(CHCl3)M06-2X/6-31G(d)]E_{SCF}: -40.48212972

C	-3.24898552121205	0.51115422800242	-0.00000001118097
H	-2.88544137020736	-0.51787349651554	0.00000000297486
H	-2.88534035074907	1.02570729027247	0.89126626193538
H	-2.88534032570938	1.02570729943776	-0.89126627186090
H	-4.34048483212212	0.51112088880287	0.00000001813164

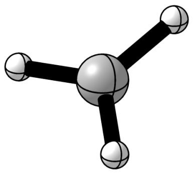	Methyl Radical	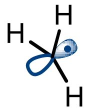

Charge: 0
Multiplicity: 2
[CPCM(CHCl3)M06-2X/6-31G(d)]G_{298K}: -39.79497617
[CPCM(CHCl3)M06-2X/6-31G(d)]H_{298K}: -39.77074906
[CPCM(CHCl3)M06-2X/6-31G(d)]E_{SCF}: -39.80468968

C	-3.33843715978413	0.38486208335363	-0.21837284485542
H	-2.82866872494364	-0.51528105955777	0.09338874315830
H	-2.82899725302706	1.10524375050142	-0.84207998538905
H	-4.35704361224517	0.56542990570272	0.09341258708617

9. Analytical Data of Products

ethyl 4-phenyl-4,5-dihydroisoxazole-3-carboxylate (3a)

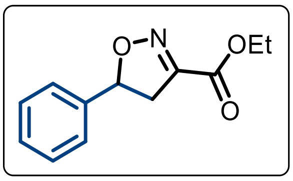

Prepared according to general procedure 7.1 on a 0.2 mmol scale, column chromatography (EtOAc/n-hexane = 1:9) afforded the title compound as a yellow liquid (33.3 mg, 76%).

^{1}H NMR (400 MHz, $CDCl_3$) δ [ppm] 7.4 – 7.2 (m, 5H), 5.7 (dd, *J* = 11.6, 8.8 Hz, 1H), 4.3 (q, *J* = 7.1 Hz, 2H), 3.6 (dd, *J* = 17.8, 11.6 Hz, 1H), 3.2 (dd, *J* = 17.8, 8.9 Hz, 1H), 1.3 (t, *J* = 7.1 Hz, 3H).

^{13}C NMR (101 MHz, $CDCl_3$) δ [ppm] 160.59, 151.12, 139.51, 128.88, 128.66, 125.87, 84.96, 62.17, 41.47, 14.13.

HRMS (EI+) m/z: [M]+ calcd. for $C_{12}H_{14}NO_3$ 219.0895, found 219.08870.

ethyl 4-(o-tolyl)-4,5-dihydroisoxazole-3-carboxylate (3b)

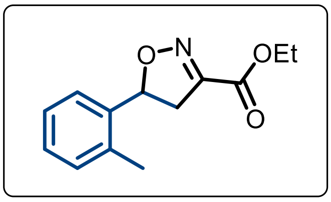

Prepared according to general procedure 7.1 on a 0.2 mmolar scale, column chromatography (EtOAc/n-hexane = 1:9) afforded the title compound as a yellow liquid (25.1 mg, 54%).

^{1}H NMR (400 MHz, $CDCl_3$) δ [ppm] 7.4 – 7.3 (m, 1H), 7.3 – 7.2 (m, 3H), 6.0 (dd, *J* = 11.7, 8.8 Hz, 1H), 4.4 (q, *J* = 7.1 Hz, 2H), 3.7 (dd, *J* = 17.6, 11.7 Hz, 1H), 3.2 – 3.1 (m, 1H), 2.3 (s, 3H), 1.4 (t, *J* = 7.1 Hz, 3H).

^{13}C NMR (101 MHz, $CDCl_3$) δ [ppm] 160.61, 151.07, 137.73, 134.20, 130.72, 128.32, 126.51, 124.95, 82.58, 62.18, 40.75, 19.27, 14.14.

HRMS (ESI+) m/z: [M+H]+ calcd. for $C_{13}H_{16}NO_3$ 234.1130, found 234.1130.

ethyl 4-(2-chlorophenyl)-4,5-dihydroisoxazole-3-carboxylate (3c)

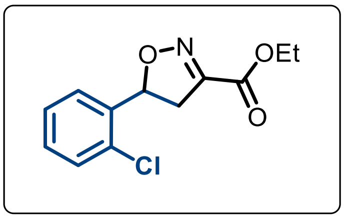

Prepared according to general procedure 7.1 on a 0.2 mmolar scale, column chromatography (EtOAc/n-hexane = 1:9) afforded the title compound as a yellow liquid (28.3 mg, 56%).

^{1}**H NMR** (400 MHz, $CDCl_3$) δ [ppm] 7.5 – 7.4 (m, 1H), 7.4 (dd, *J* = 7.4, 1.8 Hz, 1H), 7.3 – 7.2 (m, 2H), 6.1 (dd, *J* = 11.7, 7.8 Hz, 1H), 4.3 (q, *J* = 7.1 Hz, 2H), 3.8 (dd, *J* = 17.9, 11.7 Hz, 1H), 3.1 – 3.0 (dd, 1H), 1.4 (t, *J* = 7.1 Hz, 3H).

^{13}**C NMR** (101 MHz, $CDCl_3$) δ [ppm] 160.36, 151.34, 137.69, 129.81, 129.71, 129.49, 127.29, 126.43, 81.83, 62.25, 41.36, 14.12.

HRMS (ESI+) m/z: [M+H]+ calcd. $C_{12}H_{13}ClNO_3$ 254.0581, found 254.0581.

ethyl 4-(4-chlorophenyl)-4,5-dihydroisoxazole-3-carboxylate (3d)

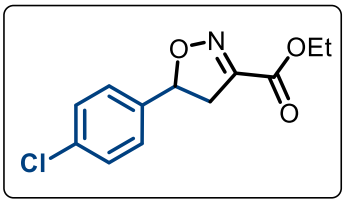

Prepared according to general procedure 7.1 on a 0.2 mmolar scale, column chromatography (EtOAc/n-hexane = 1:9) afforded the title compound as a yellow liquid (28.3 mg, 56%).

^{1}**H NMR** (400 MHz, $CDCl_3$) δ [ppm] δ 7.3 – 7.3 (m, 2H), 7.2 – 7.2 (m, 2H), 5.7 (dd, *J* = 11.6, 8.7 Hz, 1H), 4.3 (q, *J* = 7.1 Hz, 2H), 3.6 (dd, *J* = 17.8, 11.6 Hz, 1H), 3.1 (dd, *J* = 17.8, 8.7 Hz, 1H), 1.3 (t, *J* = 7.1 Hz, 3H).

^{13}**C NMR** (101 MHz, $CDCl_3$) δ [ppm] 160.41, 151.10, 138.03, 134.56, 129.09, 127.23, 84.12, 62.26, 41.54, 14.11.

HRMS (ESI+) m/z: [M+H]+ calcd. for $C_{12}H_{13}ClNO_3$ 254.0581, found: 254.0582.

ethyl 4-(4-bromophenyl)-4,5-dihydroisoxazole-3-carboxylate (3e)

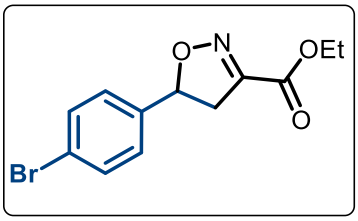

Prepared according to general procedure 7.1 on a 0.2 mmolar scale, column chromatography (EtOAc/n-hexane = 1:9) afforded the title compound as yellow liquid (38.0 mg, 64%).

^{1}**H NMR** (400 MHz, $CDCl_3$) δ [ppm] 7.2 (d, *J* = 8.5 Hz, 2H), 7.1 (d, *J* = 8.5 Hz, 2H), 5.6 (dd, *J* = 11.6, 8.7 Hz, 1H), 4.2 (q, *J* = 7.1 Hz, 2H), 3.5 (dd, *J* = 17.8, 11.6 Hz, 1H), 3.0 (dd, *J* = 17.8, 8.7 Hz, 1H), 1.3 (t, *J* = 7.1 Hz, 3H).

^{13}**C NMR** (101 MHz, $CDCl_3$) δ [ppm] 160.41, 151.11, 138.03, 134.56, 129.09, 127.24, 84.13, 62.27, 41.54, 14.12.

HRMS (ESI+) m/z: [M+H]+ calcd. for $C_{12}H_{13}BrNO_3$ 298.0079, found: 298.0073.

ethyl 5-(4-fluorophenyl)-4,5-dihydroisoxazole-3-carboxylate (3f)

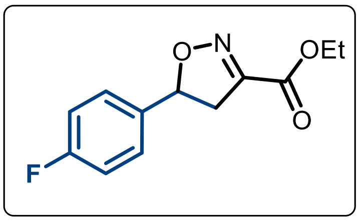

Prepared according to general procedure 7.1 on a 0.2 mmolar scale, column chromatography (EtOAc/n-hexane = 1:9) afforded the title compound as a pale-yellow liquid (38.8 mg, 82%).

^{1}H NMR (400 MHz, $CDCl_3$) δ [ppm] 7.4 – 7.3 (m, 2H), 7.2 – 7.0 (m, 2H), 5.9 (dd, *J* = 11.6, 8.7 Hz, 1H), 4.4 (q, *J* = 7.1 Hz, 2H), 3.7 (dd, *J* = 17.8, 11.6 Hz, 1H), 3.2 (dd, *J* = 17.8, 8.9 Hz, 1H), 1.4 (t, *J* = 7.1 Hz, 3H).

^{13}C NMR (101 MHz, $CDCl_3$) δ [ppm] 162.96 (d, *J* = 248.46 Hz), 160.64, 151.27, 135.44 (d, *J* = 3.0 Hz), 127.91 (d, *J* = 8.3 Hz), 116.01 (d, *J* = 21.7 Hz), 84.46, 62.39, 41.66, 14.28.

^{19}F NMR (377 MHz, $CDCl_3$) δ [ppm] -113.51 ppm.

HRMS (EI+) m/z: [M+] calcd. for $C_{12}H_{12}FNO_3$ 237.0795, found 237.0791.

ethyl 4-(3-nitrophenyl)-4,5-dihydroisoxazole-3-carboxylate (3g)

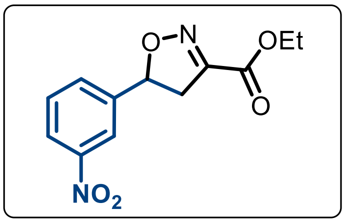

Prepared according to general procedure 7.1 on a 0.2 mmol scale, column chromatography (EtOAc/n-hexane = 1:4) afforded the title compound as a yellow liquid (37.8 mg, 76%).

^{1}H NMR (400 MHz, $CDCl_3$) δ [ppm] 8.3 – 8.2 (m, 2H), 7.7 – 7.5 (m, 2H), 5.9 (dd, *J* = 11.7, 8.3 Hz, 1H), 4.4 (q, *J* = 7.1 Hz, 2H), 3.8 (dd, *J* = 17.8, 11.7 Hz, 1H), 3.3 – 3.2 (m, 1H), 1.4 (t, *J* = 7.1 Hz, 3H).

^{13}C NMR (101 MHz, $CDCl_3$) δ [ppm] 160.16, 151.23, 148.56, 141.86, 131.89, 130.16, 123.60, 120.94, 83.37, 62.47, 41.84, 14.16.

HRMS (ESI+) m/z: [M+H]+ calcd. for $C_{12}H_{13}N_2O_5$ 265.0824, found 265.0821.

ethyl 4-(4-nitrophenyl)-4,5-dihydroisoxazole-3-carboxylate (3h)

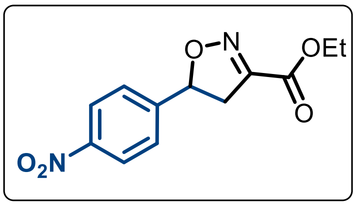

Prepared according to general procedure 7.1 on a 0.2 mmolar scale, column chromatography (EtOAc/n-hexane = 1:4) afforded the title compound as a yellow liquid (32.8 mg, 66%).

^{1}H NMR (400 MHz, $CDCl_3$) δ [ppm] 8.2 – 8.1 (m, 2H), 7.5 – 7.4 (m, 2H), 5.8 (dd, *J* = 11.8, 8.2 Hz, 1H), 4.3 (q, *J* = 7.1 Hz, 2H), 3.7 (dd, *J* = 17.8, 11.8 Hz, 1H), 3.1 (dd, *J* = 17.8, 8.2 Hz, 1H), 1.3 (t, *J* = 7.1 Hz, 3H).

^{13}C NMR (101 MHz, $CDCl_3$) δ [ppm] 160.09, 151.07, 147.99, 146.67, 126.57, 124.18, 83.29, 62.45, 41.86, 14.10.

HRMS (ESI+) m/z: [M+H]+ calcd. for $C_{12}H_{13}N_2O_5$ 265.0824, found 265.0823.

ethyl 4-(4-methoxyphenyl)-4,5-dihydroisoxazole-3-carboxylate (3i)

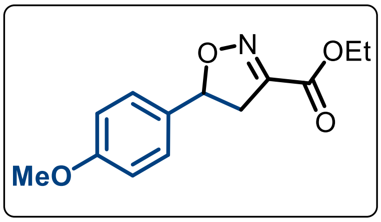

Prepared according to general procedure 7.1 on a 0.2 mmolar scale, column chromatography (EtOAc/n-hexane = 1:3) afforded the title compound as a pale-yellow liquid (19.4 mg, 39%) with the inseparable side product (4-methoxyphenyl)(5-(4-methoxyphenyl)-4,5-dihydroisoxazol-3-yl)methanone (12% according to 1H NMR).[1]

^{1}H NMR (400 MHz, $CDCl_3$) δ [ppm] 7.3 – 7.2 (m, 2H), 6.9 (d, J = 8.7 Hz, 2H), 5.7 (dd, J = 11.5, 9.2 Hz, 1H), 4.3 (q, J = 7.1 Hz, 2H), 3.8 (s, 3H), 3.5 (dd, J = 17.8, 11.5 Hz, 1H), 3.2 (dd, J = 17.8, 9.2 Hz, 1H), 1.3 (t, J = 7.1 Hz, 3H).

^{13}C NMR (101 MHz, $CDCl_3$) δ [ppm] 160.68, 159.94, 151.18, 131.34, 127.50, 114.24, 85.01, 62.13, 55.34, 41.12, 14.14.

HRMS (ESI+) m/z: [M+H]+ calcd. for $C_{13}H_{16}NO_4$ 250.1079, found 250.1079.

ethyl 4-(perfluorophenyl)-4,5-dihydroisoxazole-3-carboxylate (3j)

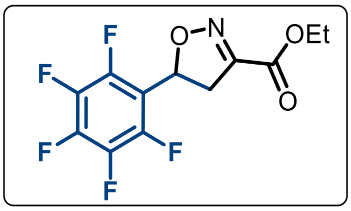

Prepared according to general procedure 7.1 on a 0.2 mmolar scale, column chromatography (EtOAc/n-hexane = 1:7) afforded the title compound as a white solid (45.7 mg, 74%).

^{1}H NMR (400 MHz, $CDCl_3$) δ [ppm] 6.0 (dd, J = 12.5, 8.9 Hz, 1H), 4.3 (q, J = 7.1 Hz, 2H), 3.6 (m, J = 17.9, 12.5, 0.9 Hz, 1H), 3.3 (m, J = 17.8, 8.9, 0.7 Hz, 1H), 1.3 (t, J = 7.1 Hz, 3H)

^{13}C NMR (101 MHz, $CDCl_3$) δ [ppm] 146.25 (ddt, J = 11.7, 7.8, 4.0 Hz), 144.58 (ddt, J = 11.7, 7.8, 4.0 Hz), 143.04 – 142.60 (m), 141.14 (tt, J = 13.4, 5.3 Hz), 138.74 (ddd, J = 17.4, 12.8, 4.9 Hz), 137.49 – 136.66 (m), 74.39, 62.62, 39.66, 14.24.

^{19}F NMR (377 MHz, $CDCl_3$) δ -141.89 – -143.28 (m), -152.12 (tt, J = 21.0, 2.9 Hz), -160.87 – -161.47 (m).

HRMS (ESI+) m/z: [M+H]+ calcd. for $C_{12}H_9F_5NO_3$ 310.0503, found 310.0501.

[1] See NMR spectra for more detail.

ethyl 4-mesityl-4,5-dihydroisoxazole-3-carboxylate (3k)

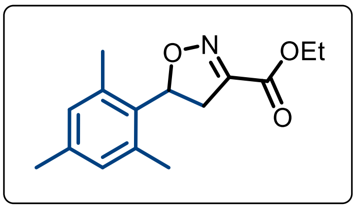

Prepared according to general procedure 7.1 on a 0.2 mmolar scale, column chromatography (EtOAc/n-hexane = 1:9) afforded the title compound as a yellow liquid (37.5 mg, 72%).

^{1}H NMR (400 MHz, $CDCl_3$) δ [ppm] 6.8 (s, 2H), 6.1 (t, J = 12.7 Hz, 1H), 4.4 (qd, J = 7.1, 2.2 Hz, 2H), 3.5 (dd, J = 17.9, 12.5 Hz, 1H), 3.2 (dd, J = 17.9, 12.9 Hz, 1H), 2.3 (s, 6H), 2.3 (s, 3H), 1.4 (t, J = 7.1 Hz, 3H).

^{13}C NMR (101 MHz, $CDCl_3$) δ [ppm] 160.85, 151.56, 138.24, 136.83, 130.64, 130.26, 82.81, 62.13, 38.98, 20.82, 20.13, 14.15.

HRMS (EI+) m/z: [M]+ calcd. for $C_{15}H_{19}NO_3$ 261.1365, found 261.1358.

ethyl 5-methyl-5-phenyl-4,5-dihydroisoxazole-3-carboxylate (3l)

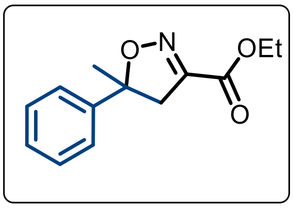

Prepared according to general procedure 7.1 on a 0.2 mmolar scale, column chromatography (EtOAc/n-hexane = 1:9) afforded the title compound as a yellow liquid (27.9 mg, 60%).

^{1}H NMR (400 MHz, $CDCl_3$) δ [ppm] δ 7.4 – 7.3 (m, 4H), 7.3 – 7.2 (m, 1H), 4.3 (q, J = 7.1 Hz, 2H), 3.4 – 3.2 (m, 2H), 1.7 (s, 3H), 1.3 (t, J = 7.1 Hz, 3H).

^{13}C NMR (101 MHz, $CDCl_3$) δ [ppm] 160.77, 151.01, 144.29, 128.63, 127.74, 124.46, 91.21, 62.05, 47.14, 28.19, 14.13.

HRMS (ESI+) m/z: [M+H]+ calcd. for $C_{13}H_{16}NO_3$ 234.1130, found 234.1127.

ethyl 3a,4,5,9b-tetrahydronaphtho[1,2-d]isoxazole-1-carboxylate (3m)

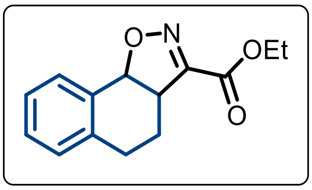

Prepared according to general procedure 7.1 on a 0.2 mmolar scale, column chromatography (EtOAc/n-hexane = 1:9) afforded the title compound as yellow liquid (20.5 mg, 42%).

^{1}H NMR (400 MHz, $CDCl_3$) δ 7.5 – 7.3 (m, 1H), 7.3 (q, J = 3.1 Hz, 2H), 7.2 (s, 1H), 5.6 (d, J = 10.1 Hz, 1H), 4.4 – 4.3 (m, 2H), 3.7 (d, J = 10.2 Hz, 1H), 2.7 – 2.6 (m, 2H), 2.0 – 1.9 (m, 2H), 1.4 (t, J = 7.1 Hz, 3H).

^{13}C NMR (101 MHz, $CDCl_3$) δ [ppm] 160.79, 154.47, 139.10, 130.35, 129.00, 128.36, 126.87, 83.47, 62.01, 44.77, 27.07, 24.38, 14.18.

HRMS (EI+) m/z: [M+] calcd. for $C_{14}H_{15}NO_3$ 245.1052, found 245.1038.

ethyl 4-phenethyl-4,5-dihydroisoxazole-3-carboxylate (3n)

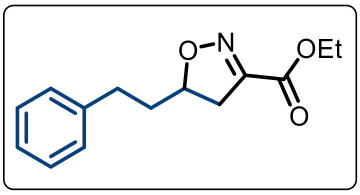

Prepared according to general procedure 7.1 on a 0.2 mmolar scale, column chromatography (EtOAc/n-hexane = 1:9) afforded the title compound as a yellow liquid (39.5 mg, 80%).

^{1}H NMR (500 MHz, $CDCl_3$) δ [ppm] 7.34 – 7.27 (m, 2H), 7.21 (ddd, *J* = 8.3, 7.0, 1.3 Hz, 3H), 4.79 (dtd, *J* = 11.0, 7.9, 5.5 Hz, 1H), 4.35 (q, *J* = 7.1 Hz, 2H), 3.25 (dd, *J* = 17.5, 11.0 Hz, 1H), 2.86 (dd, *J* = 17.6, 8.3 Hz, 1H), 2.82 – 2.69 (m, 2H), 2.09 (dddd, *J* = 13.6, 9.2, 7.6, 5.8 Hz, 1H), 1.90 (dddd, *J* = 13.8, 9.5, 6.8, 5.5 Hz, 1H), 1.37 (t, *J* = 7.1 Hz, 3H).

^{13}C NMR (126 MHz, $CDCl_3$) δ [ppm] 160.99, 151.57, 140.78, 128.70, 128.57, 126.36, 83.22, 62.19, 38.59, 36.97, 31.55, 14.28.

HRMS (EI+) m/z: [M+] calcd. for $C_{14}H_{17}NO_3$ 247.1208, found 247.1202.

ethyl 4-cyclohexyl-4,5-dihydroisoxazole-3-carboxylate (3o)

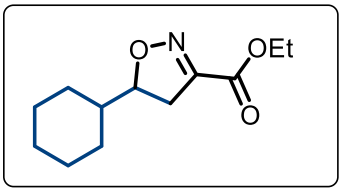

Prepared according to general procedure 7.1 on a 0.2 mmolar scale, column chromatography (EtOAc/n-hexane = 1:9) afforded the title compound as a yellow liquid (25.2 mg, 56%).

^{1}H NMR (400 MHz, $CDCl_3$) δ [ppm] δ 4.5 (ddd, *J* = 11.2, 9.0, 6.7 Hz, 1H), 4.3 (q, *J* = 7.1 Hz, 2H), 3.1 (dd, *J* = 17.6, 11.2 Hz, 1H), 2.9 (dd, *J* = 17.7, 9.1 Hz, 1H), 1.9 – 1.6 (m, 3H), 1.6 – 1.5 (m, 3H), 1.3 (d, *J* = 7.2 Hz, 3H), 1.2 – 1.1 (m, 3H), 1.0 (ddd, *J* = 11.9, 8.9, 3.5 Hz, 2H).

^{13}C NMR (101 MHz, $CDCl_3$) δ [ppm] 160.95, 151.35, 88.32, 61.94, 42.17, 35.83, 28.22, 28.03, 26.21, 25.70, 25.55, 14.14.

HRMS (EI+) m/z: [M+] calcd. for $C_{12}H_{19}NO_3$ 225.1365, found 225.1359.

ethyl 3a,4,5,6,7,8,9,9a-octahydrocycloocta[d]isoxazole-3-carboxylate (3p)

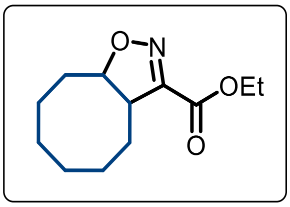

Prepared according to general procedure 7.1 on a 0.2 mmolar scale, column chromatography (EtOAc/n-hexane = 1:9) afforded the title compound as a yellow liquid (43.2 mg, 96%).

^{1}H NMR (400 MHz, $CDCl_3$) δ 4.5 (ddd, *J* = 11.3, 10.0, 3.1 Hz, 1H), 4.3 (qd, *J* = 7.1, 0.7 Hz, 2H), 3.2 (t, *J* = 10.1 Hz, 1H), 2.1 – 1.9 (m, 2H), 1.8 – 1.7 (m, 2H), 1.7 – 1.5 (m, 4H), ,1.5 – 1.4 (m, 2H), 1.3 (t, *J* = 7.1 Hz, 3H), 1.3 – 1.1 (m, 2H).

^{13}C NMR (101 MHz, $CDCl_3$) δ [ppm] 160.91, 156.31, 88.05, 61.83, 48.97, 29.75, 25.54, 25.44, 25.13, 25.09, 24.57, 14.13.

HRMS (EI+) m/z: [M+] calcd. for $C_{12}H_{20}NO_3$ 225.1365, found 225.1363.

ethyl 3a,5,6,6a-tetrahydro-4H-cyclopenta[d]isoxazole-3-carboxylate (3q)

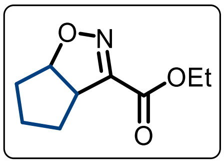

Prepared according to general procedure 7.1 on a 0.2 mmolar scale, column chromatography (EtOAc/n-hexane = 1:9) afforded the title compound as a yellow liquid (21.2 mg, 58%).

^{1}H NMR (400 MHz, $CDCl_3$) δ 5.3 – 5.2 (m, 1H), 4.3 – 4.2 (m, 2H), 3.8 – 3.8 (m, 1H), 2.2 – 2.1 (m, 1H), 2.0 – 1.9 (m, 1H), 1.8 – 1.6 (m, 3H), 1.4 – 1.3 (m, 1H), 1.3 (t, *J* = 7.2 Hz, 3H).

^{13}C NMR (101 MHz, $CDCl_3$) δ [ppm] 160.87, 153.42, 90.30, 61.82, 50.95, 35.59, 31.57, 23.13, 14.13.

HRMS (ESI+) m/z: [M+H]+ calcd. for $C_9H_{14}NO_3$ 184.0974, found 184.0970.

ethyl 4-(benzoyloxy)-4,5-dihydroisoxazole-3-carboxylate (3r)

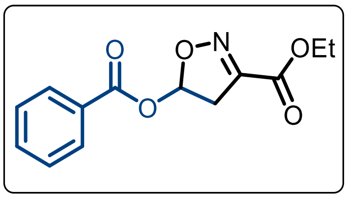

Prepared according to general procedure 7.1 on a 0.2 mmolar scale, column chromatography (EtOAc/n-hexane = 1:6) afforded the title compound as yellow liquid (37.3 mg, 71%).

^{1}H NMR (400 MHz, $CDCl_3$) δ [ppm] 8.1 – 8.0 (m, 2H), 7.6 – 7.6 (m, 1H), 7.5 – 7.4 (m, 2H), 7.1 (dd, *J* = 7.2, 1.9 Hz, 1H), 4.4 (q, *J* = 7.1 Hz, 2H), 3.6 (dd, *J* = 19.0, 7.2 Hz, 1H), 3.4 (dd, *J* = 19.0, 1.9 Hz, 1H), 1.4 (t, *J* = 7.1 Hz, 3H).

^{13}C NMR (101 MHz, $CDCl_3$) δ [ppm] 164.78, 159.80, 152.03, 133.87, 130.01, 128.68, 128.54, 97.24, 62.63, 40.20, 14.11.

HRMS (ESI+) m/z: [M+H]+ calcd. for $C_{13}H_{14}NO_5$ 264.0872, found 264.0867.

5-benzyl 3-ethyl 5-methyl-4,5-dihydroisoxazole-3,5-dicarboxylate (**3s**)

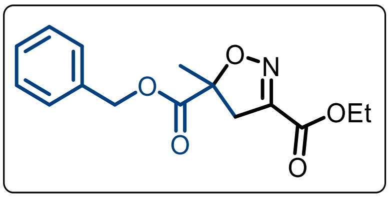

Prepared according to general procedure 7.1 on a 0.2 mmolar scale, column chromatography (EtOAc/n-hexane = 1:5) afforded the title compound as yellow liquid (40.1 mg, 69%).

^{1}**H NMR** (400 MHz, $CDCl_3$) δ [ppm] 7.3 – 7.2 (m, 5H), 5.2 (s, 2H), 4.3 – 4.2 (q, 2H), 3.6 (d, *J* = 18.0 Hz, 1H), 3.0 (d, *J* = 18.0 Hz, 1H), 1.6 (s, 3H), 1.3 – 1.2 (t, *J* = 7.1 Hz, 3H).

^{13}**C NMR** (101 MHz, $CDCl_3$) δ [ppm] 170.43, 160.10, 151.00, 135.00, 128.67, 128.54, 128.10, 88.56, 67.75, 63.27, 62.28, 43.41, 23.41, 14.10, 13.94.

HRMS (ESI+) m/z: [M+H]+ calcd. for $C_{15}H_{18}NO_5$ $[M+H]^+$ 292.1185, found 292.1188

ethyl 5-(2-(oxiran-2-yl)ethyl)-4,5-dihydroisoxazole-3-carboxylate (**3t**)

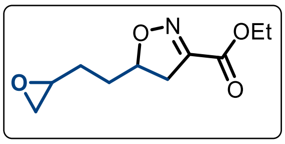

Prepared according to general procedure 7.1 on a 0.2 mmolar scale, column chromatography (EtOAc/n-hexane = 1:5) afforded the title compound as a yellow liquid (29.8 mg, 70%).

^{1}**H NMR** (400 MHz, $CDCl_3$) δ [ppm] 4.9 – 4.7 (m, 1H), 4.3 (qd, *J* = 7.1, 0.5 Hz, 2H), 3.2 (ddd, *J* = 17.6, 10.9, 3.3 Hz, 1H), 2.9 (ddtd, *J* = 10.6, 6.6, 4.0, 2.6 Hz, 1H), 2.8 (ddd, *J* = 17.6, 8.2, 3.7 Hz, 1H), 2.7 – 2.7 (m, 1H), 2.4 (ddd, *J* = 5.0, 2.7, 0.7 Hz, 1H), 1.9 – 1.7 (m, 3H), 1.6 – 1.4 (m, 1H), 1.3 (t, *J* = 7.1 Hz, 3H).

^{13}**C NMR** (101 MHz, $CDCl_3$) δ [ppm] 160.71, 151.40, 83.17, 62.03, 51.40, 47.03, 38.63, 31.74, 27.87, 14.12.

HRMS (ESI+) m/z: [M+H]+ calcd. for $C_{10}H_{16}NO_4$ 214.1079, found 214.1073.

ethyl 4-hexyl-4,5-dihydroisoxazole-3-carboxylate (**3u**)

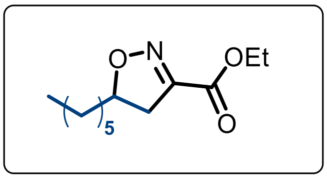

Prepared according to general procedure 7.1 on a 0.2 mmolar scale, column chromatography (EtOAc/n-hexane = 1:9) afforded the title compound as a yellow liquid (32.7 mg, 72%).

^{1}**H NMR** (400 MHz, $CDCl_3$) δ [ppm] 4.79 (ddt, *J* = 10.9, 8.5, 6.5 Hz, 1H), 4.34 (q, *J* = 7.1 Hz, 2H), 3.24 (dd, *J* = 17.5, 10.9 Hz, 1H), 2.83 (dd, *J* = 17.5, 8.5 Hz, 1H), 1.81 – 1.71 (m, 1H),

1.66 – 1.55 (m, 1H), 1.43 (m, 1H), 1.36 (t, J = 7.1 Hz, 3H), 1.35 – 1.23 (m, 7H), 0.92 – 0.84 (m, 3H).

^{13}C NMR (101 MHz, $CDCl_3$) δ [ppm] 160.95, 151.35, 84.20, 61.97, 38.37, 35.07, 31.63, 28.98, 25.05, 22.52, 14.14, 14.03.

HRMS (EI+) m/z: [M+] calcd. for $C_{12}H_{21}NO_3$ 227.1521, found 227.1515.

ethyl 5-decyl-4,5-dihydroisoxazole-3-carboxylate (3v)

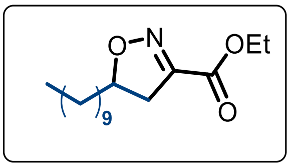

Prepared according to general procedure 7.1 on a 0.2 mmolar scale, column chromatography (EtOAc/n-hexane = 1:9) afforded the title compound as yellow liquid (49.8 mg, 88%).

^{1}H NMR (400 MHz, $CDCl_3$) δ [ppm] 4.7 (ddt, J = 10.9, 8.6, 6.5 Hz, 1H), 4.3 (q, J = 7.1 Hz, 2H), 3.2 (dd, J = 17.5, 10.9 Hz, 1H), 2.8 (dd, J = 17.5, 8.5 Hz, 1H), 1.8 – 1.7 (m, 1H), 1.6 – 1.5 (m, 2H), 1.3 (t, J = 7.1 Hz, 3H), 1.2 (m, 14H), 0.8 (t, J = 6.7 Hz, 3H).

^{13}C NMR (101 MHz, $CDCl_3$) δ [ppm] 160.94, 151.34, 84.19, 61.95, 38.37, 35.06, 31.88, 29.56, 29.50, 29.44, 29.32, 29.30, 25.09, 22.67, 14.13, 14.09.

HRMS (ESI+) m/z: [M+H]+ calcd. for $C_{16}H_{29}NO_3$ 284.2226, found 284.2226.

ethyl 5-(2-hydroxyethyl)-4,5-dihydroisoxazole-3-carboxylate (3w)

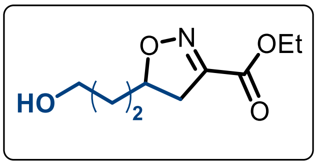

Prepared according to general procedure 7.1 on a 0.2 mmolar scale, column chromatography (EtOAc/n-hexane = 1:2) afforded the title compound as yellow liquid (20.1 mg, 50%).

^{1}H NMR (400 MHz, $CDCl_3$) δ [ppm] 4.9 – 4.8 (m, 1H), 4.4 – 4.3 (m, 2H), 3.7 (t, J = 5.8 Hz, 2H), 3.3 (dd, J = 17.6, 10.9 Hz, 1H), 2.9 (dd, J = 17.6, 8.3 Hz, 1H), 1.8 – 1.7 (m, 5H), 1.4 (t, J = 7.1 Hz, 3H).

^{13}C NMR (101 MHz, $CDCl_3$) δ [ppm] 160.82, 151.46, 83.82, 62.23, 62.05, 38.56, 31.61, 28.28, 14.13.

HRMS (ESI+) m/z: [M+H]+ calcd. for $C_9H_{16}NO_4$ 202.1079, found 202.1074.

ethyl 4-(4-chlorobutyl)-4,5-dihydroisoxazole-3-carboxylate (3x)

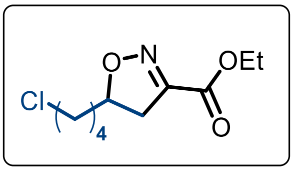

Prepared according to general procedure 7.1 on a 0.2 mmolar scale, column chromatography (EtOAc/n-hexane = 1:9) afforded the title compound as a yellow liquid (33.5 mg, 72%).

^{1}H NMR (400 MHz, $CDCl_3$) δ [ppm] 4.7 (dddd, *J* = 11.0, 8.4, 7.1, 5.2 Hz, 1H), 4.3 (q, *J* = 7.1 Hz, 2H), 3.5 (t, *J* = 6.5 Hz, 2H), 3.2 (dd, *J* = 17.6, 11.0 Hz, 1H), 2.8 – 2.7 (m, 1H), 1.9 – 1.7 (m, 3H), 1.6 – 1.5 (m, 1H), 1.5 (d, *J* = 6.3 Hz, 2H), 1.3 (t, *J* = 7.1 Hz, 3H).

^{13}C NMR (101 MHz, $CDCl_3$) δ [ppm] 160.80, 151.37, 83.71, 62.04, 44.58, 38.46, 34.31, 32.13, 22.53, 14.13.

HRMS (EI+) m/z: [M+] calcd. for $C_{10}H_{16}ClNO_3$ 233.0819, found 233.0813.

ethyl 5-(3-bromopropyl)-4,5-dihydroisoxazole-3-carboxylate (3y)

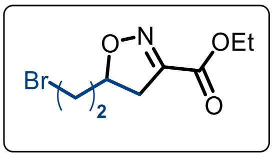

Prepared according to general procedure 7.1 on a 0.2 mmolar scale, column chromatography (EtOAc/n-hexane = 1:9) afforded the title compound as yellow liquid (36.8 mg, 70%).

^{1}H NMR (400 MHz, $CDCl_3$) δ [ppm] 5.0 (dddd, *J* = 10.9, 8.5, 7.6, 4.4 Hz, 1H), 4.3 (q, *J* = 7.1 Hz, 2H), 3.5 – 3.4 (m, 2H), 3.3 (dd, *J* = 17.6, 11.0 Hz, 1H), 2.8 (dd, *J* = 17.6, 7.6 Hz, 1H), 2.3 (ddt, *J* = 14.3, 8.5, 5.6 Hz, 1H), 2.1 (dddd, *J* = 14.9, 8.6, 6.6, 4.4 Hz, 1H), 1.4 (t, *J* = 7.2 Hz, 3H).

^{13}C NMR (101 MHz, $CDCl_3$) δ [ppm] 160.56, 151.52, 81.39, 62.19, 38.58, 38.05, 28.29, 14.12.

HRMS (ESI) m/z: [M+H]+ calcd. for $C_8H_{13}BrNO_3$ 250.0079, found 250.0076, [M+2+] 252.0061.

Ethyl methyl 4,5-dihydroisoxazole-3,4-dicarboxylate (3z)

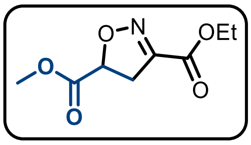

Prepared according to general procedure 7.1 on a 0.2 mmolar scale, column chromatography (EtOAc/n-hexane = 1:9) afforded the title compound as a yellow liquid (31.7 mg, 79%)

^{1}H NMR (400 MHz, $CDCl_3$) δ [ppm] 5.2 (dd, *J* = 11.0, 8.3 Hz, 1H), 4.3 (q, *J* = 7.1 Hz, 2H), 3.8 (s, 3H), 3.5 (d, *J* = 1.5 Hz, 1H), 3.5 (d, *J* = 4.2 Hz, 1H), 1.3 (t, *J* = 7.1 Hz, 3H).

^{13}C NMR (101 MHz, $CDCl_3$) δ [ppm] 169.36, 159.77, 151.09, 79.69, 62.37, 52.97, 37.60, 14.04.

HRMS (ESI+) m/z: [M+H]+ calcd. for $C_8H_{12}NO_5$ 202.0715, found 202.0711.

diethyl 4,5-dihydroisoxazole-3,4-dicarboxylate (3aa)

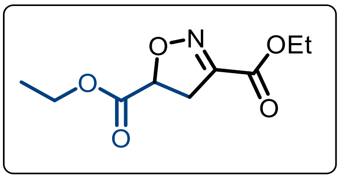

Prepared according to general procedure 7.1 on a 0.2 mmolar scale, column chromatography (EtOAc/n-hexane = 1:8) afforded the title compound as yellow liquid (32.6 mg, 76%).

^{1}H NMR (400 MHz, $CDCl_3$) δ [ppm] 5.2 (dd, *J* = 11.3, 8.1 Hz, 1H), 4.3 (q, *J* = 7.1 Hz, 2H), 4.3 – 4.2 (m, 2H), 3.5 – 3.5 (m, 1H), 3.5 (d, *J* = 6.7 Hz, 1H), 1.4 (t, *J* = 7.1 Hz, 3H), 1.3 (t, *J* = 7.1 Hz, 3H).

^{13}C NMR (101 MHz, $CDCl_3$) δ [ppm] 168.87, 159.87, 151.03, 79.87, 62.40, 62.29, 37.59, 14.09, 14.06.

HRMS (ESI+) m/z: [M+H]+ calcd. for $C_9H_{14}NO_5$ 216.0872, found 216.0871.

ethyl 4-cyano-4,5-dihydroisoxazole-3-carboxylate (3ab)

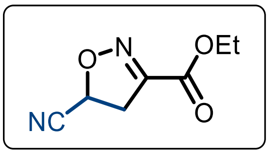

Prepared according to general procedure 7.1 on a 0.2 mmolar scale, column chromatography (EtOAc/n-hexane = 1:5) afforded the title compound as yellow liquid (12.7 mg, 38%).

^{1}H NMR (400 MHz, $CDCl_3$) δ [ppm] 5.3 (dd, *J* = 10.4, 7.8 Hz, 1H), 4.4 – 4.2 (m, 2H), 3.6 – 3.5 (m, 2H), 2.6 – 2.4 (m, 1H), 1.3 (m, *J* = 7.1, 5.7 Hz, 3H).

^{13}C NMR (101 MHz, $CDCl_3$) δ [ppm] 159.07, 151.29, 115.97, 68.17, 63.07, 40.10, 14.17.

HRMS (EI+) m/z: [M+] calcd. for $C_7H_8N_2O_3$ 168.0535, found 168.0529.

ethyl 2-hydroxy-4-oxohexahydro-2H-cyclopenta[d]isoxazole-3-carboxylate (3ac)

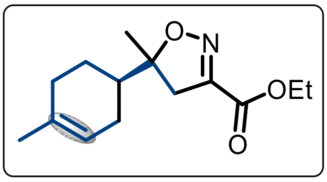

Prepared according to general procedure 7.1 on a 0.2 mmolar scale, coloumn chromatography (EtOAc/n-hexane = 1:6) afforded the title compound as yellow liquid (21.0 mg, 42%).

^{1}H NMR (400 MHz, $CDCl_3$) δ [ppm] 5.3 (dp, *J* = 5.0, 1.6 Hz, 1H), 4.3 (qd, *J* = 7.2, 1.0 Hz, 2H), 3.0 (dd, *J* = 17.7, 1.9 Hz, 1H), 2.7 (dd, *J* = 17.7, 14.1 Hz, 1H), 2.1 – 1.9 (m, 3H), 1.9 – 1.6 (m, 4H), 1.6 (dt, *J* = 2.6, 1.3 Hz, 3H), 1.3 – 1.3 (m, 6H).

^{13}C NMR (101 MHz, $CDCl_3$) δ [ppm] 161.21, 150.81, 134.33, 134.15, 119.73, 93.79, 93.40, 62.00, 42.42, 42.24, 41.91, 40.97, 30.41, 30.34, 26.86, 26.40, 24.18, 23.92, 23.69, 23.43, 23.38, 14.26.

HRMS (ESI+) m/z: [M+H]+ calcd. for $C_{14}H_{22}NO_3$ 252.1600, found 252.1598.

ethyl 5-(4-(((2-(3-benzoylphenyl)propanoyl)oxy)methyl)phenyl)-4,5-dihydroisoxazole-3 - carboxylate (3ad)

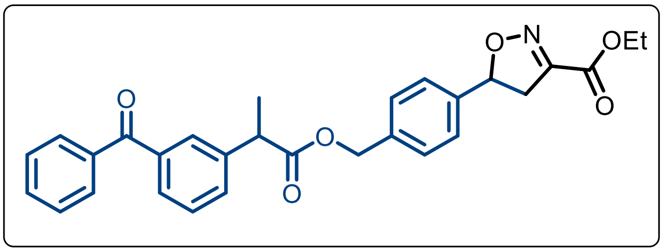

Prepared according to general procedure 7.1 on a 0.2 mmolar scale, column chromatography (EtOAc/n-hexane = 1:8) afforded the title compound as yellow liquid (67.9 mg, 70%).

^{1}H NMR (400 MHz, $CDCl_3$) δ 7.8 – 7.7 (m, 3H), 7.6 (dt, J = 7.6, 1.5 Hz, 1H), 7.6 – 7.5 (m, 1H), 7.5 – 7.5 (m, 1H), 7.4 (dt, J = 14.9, 7.7 Hz, 3H), 7.2 – 7.2 (m, 4H), 5.7 (ddd, J = 11.6, 8.8, 1.6 Hz, 1H), 5.1 (d, J = 1.0 Hz, 2H), 4.3 (q, J = 7.1 Hz, 2H), 3.9 – 3.8 (m, 1H), 3.6 (ddd, J = 17.8, 11.6, 0.8 Hz, 1H), 3.1 (dd, J = 17.8, 8.8 Hz, 1H), 1.5 (d, J = 7.2 Hz, 3H), 1.3 (t, J = 7.1 Hz, 3H).

^{13}C NMR (101 MHz, $CDCl_3$) δ [ppm] 196.41, 173.74, 160.49, 151.11, 140.59, 139.51, 137.94, 137.44, 136.36, 132.53, 131.50, 130.05, 129.22, 129.06, 128.56, 128.39, 128.32, 126.05, 84.54, 66.10, 62.20, 45.39, 41.46, 18.34, 14.13.

HRMS (ESI+) m/z: [M+H]+ calcd. for $C_{29}H_{28}NO_6$ 486.1917, found 486.1917.

ethyl 5-((8R,9S,13S,14S)-13-methyl-17-oxo-7,8,9,11,12,13,14,15,16,17-decahydro-6H-cyclopenta[a]phenanthren-3-yl)-4,5-dihydroisoxazole-3-carboxylate (3ae)

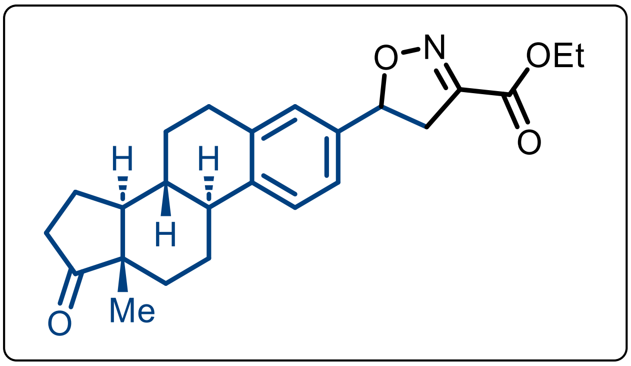

Prepared according to general procedure 7.1 on a 0.2 mmolar scale, column chromatography (EtOAc/n-hexane = 1:5) afforded the title compound as yellow liquid (61.6 mg, 78%).

^{1}H NMR (400 MHz, $CDCl_3$) δ 7.3 (d, J = 7.9 Hz, 1H), 7.1 – 7.0 (m, 2H), 5.7 (dd, J = 11.5, 8.9 Hz, 1H), 4.3 (q, J = 7.1 Hz, 2H), 3.6 (ddd, J = 17.7, 11.6, 0.6 Hz, 1H), 3.2 (dd, J = 17.8, 8.9 Hz, 1H), 2.9 (dd, J = 9.1, 4.4 Hz, 1H), 2.5 – 2.3 (m, 2H), 2.3 (td, J = 10.7, 4.3 Hz, 4H), 2.2 – 1.9 (m, 4H), 1.7 – 1.4 (m, 8H), 1.3 (t, J = 7.1 Hz, 3H).

^{13}C NMR (101 MHz, $CDCl_3$) δ [ppm] 220.84, 160.78, 151.26, 140.55, 137.34, 137.02, 136.99, 126.67, 126.65, 126.03, 123.53, 123.50, 85.04, 84.99, 62.27, 50.61, 48.07, 44.49, 41.32, 38.16, 35.96, 31.68, 29.52, 29.50, 26.51, 26.50, 25.83, 21.71, 14.27, 13.95.

HRMS (ESI+) m/z: [M+H]+ calcd. for $C_{24}H_{30}NO_4$ 396.2175, found 396.2171.

Ethyl-5-(4-(((2-isopropyl-5-methylcyclohexyl)oxy)carbonyl)phenyl)-4,5-dihydroisoxazole-3-carboxylate (3af)

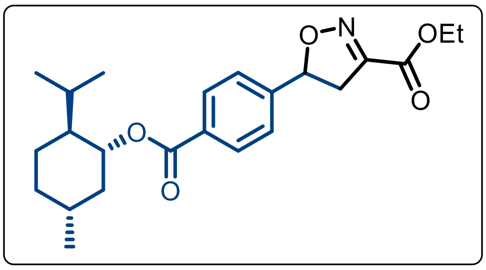

Prepared according to general procedure 7.1 on a 0.2 mmolar scale, column chromatography (EtOAc/n-hexane = 1:5) afforded the title compound as yellow liquid (67.4 mg, 84%).

^{1}H NMR (400 MHz, $CDCl_3$) δ 8.1 – 7.9 (m, 2H), 7.4 – 7.3 (m, 2H), 5.8 (dd, J = 11.6, 8.5 Hz, 1H), 4.9 (td, J = 10.9, 4.4 Hz, 1H), 4.3 (q, J = 7.1 Hz, 2H), 3.6 (dd, J = 17.8, 11.7 Hz, 1H), 3.1 (ddd, J = 17.8, 8.5, 0.9 Hz, 1H), 2.1 – 2.0 (m, 1H), 1.9 (pd, J = 7.0, 2.7 Hz, 1H), 1.7 – 1.6 (m, 2H), 1.6 – 1.4 (m, 2H), 1.3 (t, J = 7.1 Hz, 3H), 1.1 – 1.0 (m, 2H), 0.9 (t, J = 6.7 Hz, 7H), 0.7 (d, J = 7.0 Hz, 3H).

^{13}C NMR (101 MHz, $CDCl_3$) δ 165.51, 160.34, 151.07, 151.06, 144.29, 144.26, 131.10, 130.17, 125.61, 84.17, 75.09, 62.27, 47.25, 41.70, 40.94, 34.29, 31.44, 26.56, 23.67, 22.03, 20.73, 16.55, 14.11.

HRMS (ESI+) m/z: [M+H]+ calcd. for $C_{23}H_{32}NO_5$ 402.2280, found 402.2281.

3,5-diphenyl-4,5-dihydroisoxazole (4a)[2]

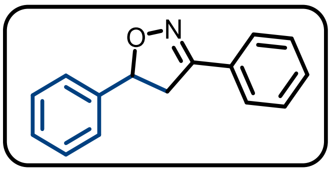

Prepared according to general procedure 7.1 on a 0.2 mmolar scale, column chromatography (EtOAc/n-hexane = 1:99) afforded the title compound as a yellow liquid (33.8 mg, 76%).

^{1}H NMR (400 MHz, $CDCl_3$) δ [ppm] 7.7 – 7.6 (m, 2H), 7.4 – 7.3 (m, 8H), 5.7 (dd, J = 11.0, 8.2 Hz, 1H), 3.8 – 3.7 (m, 1H), 3.3 (dd, J = 16.6, 8.2 Hz, 1H).

^{13}C NMR (101 MHz, $CDCl_3$) δ [ppm] 156.23, 141.09, 130.29, 129.63, 128.92, 128.89, 128.38, 126.90, 126.02, 82.73, 43.35.

[2] The compound was not stable over time and formed precipitate in the NMR-tube which was assumed to appear from imine hydrolysis byproduct.

HRMS (EI+) m/z: [M]+ calcd. for $C_{15}H_{13}NO$ 223.0973, found 223.0991.

3-(2-chlorophenyl)-5-phenyl-4,5-dihydroisoxazole (4b)

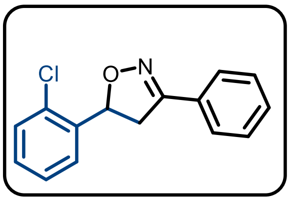

Prepared according to general procedure 7.1 on a 0.2 mmolar scale, column chromatography (EtOAc/n-hexane = 2:98) afforded the title compound as a yellow liquid (28 mg, 56%).

^{1}H NMR (400 MHz, $CDCl_3$) δ [ppm] 7.7 – 7.6 (m, 2H), 7.6 – 7.5 (m, 1H), 7.4 – 7.3 (m, 4H), 7.3 – 7.2 (m, 2H), 6.0 (dd, J = 11.1, 7.0 Hz, 1H), 3.9 (dd, J = 16.8, 11.1 Hz, 1H), 3.2 (dd, J = 16.8, 7.0 Hz, 1H).

^{13}C NMR (101 MHz, $CDCl_3$) δ [ppm] 156.31, 139.17, 131.30, 130.41, 129.66, 129.39, 129.21, 128.88, 127.37, 126.94, 126.80, 79.64, 42.93.

HRMS (EI+) m/z: [M]+ calcd. for $C_{15}H_{12}NOCl$ 257.0607, found 257.0606.

3-phenyl-5-(m-tolyl)-4,5-dihydroisoxazole (4c)[3]

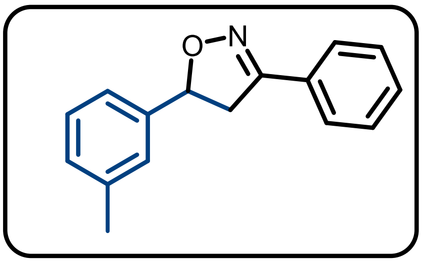

Prepared according to general procedure 7.1 on a 0.2 mmolar scale, column chromatography (EtOAc/n-hexane = 1:99) afforded the title compound as a yellow liquid (21.8 mg, 46%).

^{1}H NMR (400 MHz, $CDCl_3$) δ [ppm] 7.7 – 7.6 (m, 2H), 7.41 (p, J = 3.5 Hz, 3H), 7.2 – 7.1 (m, 4H), 5.7 (dd, J = 11.1, 8.3 Hz, 1H), 3.7 (dd, J = 16.6, 11.0 Hz, 1H), 3.3 (dd, J = 16.7, 8.3 Hz, 1H), 2.4 (s, 3H).

^{13}C NMR (101 MHz, $CDCl_3$) δ [ppm] 156.11, 140.90, 138.57, 130.12, 129.56, 128.99, 128.75, 128.66, 126.77, 126.51, 122.98, 82.64, 43.17, 21.43.

HRMS (ESI+) m/z: [M]+ calcd. for $C_{16}H_{16}NO$ 238.1232, found 237.1225.

3-(4-(tert-butyl)phenyl)-5-phenyl-4,5-dihydroisoxazole (4d)

[3] Residual grease could not be removed from the isolated compound by washing with cold pentane since the compound dissolved in the later.

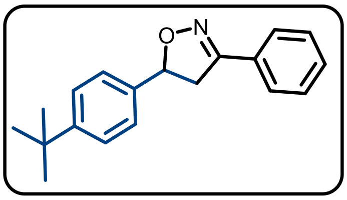

Prepared according to general procedure 7.1 on a 0.2 mmolar scale, column chromatography (EtOAc/n-hexane = 1:4) afforded the title compound as a yellow liquid (33 mg, 59%).

^{1}H NMR (400 MHz, $CDCl_3$) δ [ppm] 7.70 (ddt, *J* = 5.0, 2.5, 1.5 Hz, 2H), 7.45 – 7.36 (m, 5H), 7.36 – 7.32 (m, 2H), 5.73 (dd, *J* = 10.9, 8.4 Hz, 1H), 3.76 (dd, *J* = 16.6, 10.9 Hz, 1H), 3.37 (dd, *J* = 16.6, 8.4 Hz, 1H), 1.33 (s, 9H).

^{13}C NMR (101 MHz, $CDCl_3$) δ [ppm] 156.28, 151.44, 137.92, 130.20, 129.73, 128.85, 126.86, 125.84, 125.80, 82.64, 43.06, 34.73, 31.45.

HRMS (EI+) m/z: [M]+ calcd. for $C_{19}H_{21}NO$ 279.1623, found 279.1612.

ethyl 4-phenylisoxazole-3-carboxylate (6a)

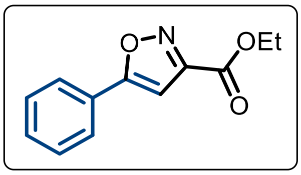

Prepared according to general procedure 7.1 on a 0.2 mmolar scale, column chromatography (EtOAc/n-hexane = 1:5) afforded the title compound as a yellow liquid (31.6 mg, 73%).

^{1}H NMR (400 MHz, $CDCl_3$) δ [ppm] 7.8 – 7.7 (m, 2H), 7.5 – 7.4 (m, 3H), 6.9 (s, 1H), 4.4 (q, *J* = 7.1 Hz, 2H), 1.4 (t, *J* = 7.1 Hz, 3H).

^{13}C NMR (101 MHz, $CDCl_3$) δ [ppm] 171.71, 160.04, 156.96, 130.80, 129.14, 126.65, 125.94, 99.92, 62.24, 14.18.

HRMS (EI+) m/z: [M+] calcd. for $C_{12}H_{11}NO_3$ 217.0739, found 217.0733.

ethyl 5-(o-tolyl)isoxazole-3-carboxylate (6b)

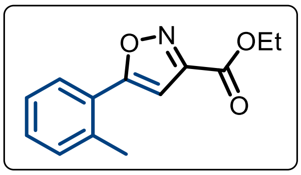

Prepared according to general procedure 7.1 on a 0.2 mmolar scale, column chromatography (EtOAc/n-hexane = 1:5) afforded the title compound as yellow liquid (32.3 mg, 70%).

^{1}H NMR (400 MHz, $CDCl_3$) δ [ppm] 7.6 (dtt, *J* = 4.4, 1.3, 0.7 Hz, 2H), 7.4 (t, *J* = 7.6 Hz, 1H), 7.3 – 7.2 (m, 1H), 6.9 (s, 1H), 4.5 – 4.5 (m, 2H), 2.4 (s, 3H), 1.5 (t, *J* = 7.1 Hz, 3H).

^{13}C NMR (101 MHz, $CDCl_3$) δ [ppm] 171.9, 160.1, 156.9, 139.0, 131.6, 129.0, 126.5, 126.5, 123.1, 99.8, 62.2, 21.4, 14.2.

HRMS (EI+) m/z: [M]+ calcd. for $C_{13}H_{13}NO_3$ 231.0895, found 231.0890.

ethyl 2-hydroxy-4-oxohexahydro-2H-cyclopenta[d]isoxazole-3-carboxylate (6c)

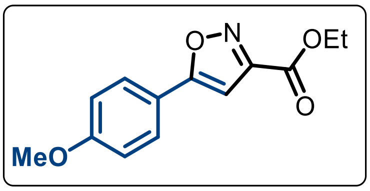

Prepared according to general procedure 7.1 on a 0.2 mmolar scale, column chromatography (EtOAc/n-hexane = 1:3) afforded the title compound as a yellow liquid (33.6 mg, 68%).

^{1}H NMR (400 MHz, $CDCl_3$) δ [ppm] 7.7 – 7.6 (m, 2H), 6.9 – 6.9 (m, 2H), 4.4 (q, J = 7.2 Hz, 2H), 3.8 (s, 3H), 1.4 (t, J = 7.2 Hz, 3H)

^{13}C NMR (101 MHz, $CDCl_3$) δ [ppm] 171.7, 161.6, 160.2, 156.9, 127.6, 119.4, 114.5, 98.5, 62.2, 55.4, 14.2.

HRMS (EI+) m/z: [M+H]+ calcd. for $C_{13}H_{13}NO_4$ 247.0845, found 247.0845.

ethyl 4-butylisoxazole-3-carboxylate (6d)

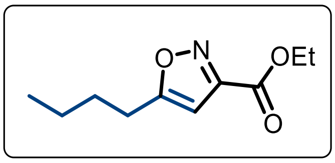

Prepared according to general procedure 7.1 on a 0.2 mmolar scale, column chromatography (EtOAc /n-hexane = 1:5) afforded the title compound as yellow liquid (18 mg, 46%).

^{1}H NMR (400 MHz, $CDCl_3$) δ 6.4 (d, J = 0.8 Hz, 1H), 4.4 (q, J = 7.1 Hz, 2H), 2.8 – 2.7 (m, 2H), 1.7 – 1.6 (m, 2H), 1.4 (t, J = 7.1 Hz, 5H), 0.9 (t, J = 7.3 Hz, 3H).

^{13}C NMR (101 MHz, $CDCl_3$) δ [ppm] 175.69, 160.28, 156.33, 101.39, 62.03, 29.42, 26.39, 22.07, 14.16, 13.61.

HRMS (EI+) m/z: [M+] calcd. for $C_{10}H_{15}NO_3$ 197.1052, found 197.1046.

ethyl 5-cyclohexylisoxazole-3-carboxylate (6e)

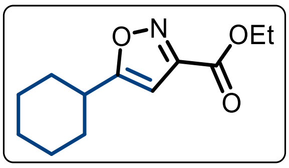

Prepared according to general procedure 7.1 on a 0.2 mmolar scale, column chromatography (EtOAc/n-hexane = 1:5) afforded the title compound as a yellow liquid (25.8 mg, 58%).

^{1}H NMR (400 MHz, $CDCl_3$) δ 6.3 (d, J = 0.9 Hz, 1H), 4.4 (q, J = 7.1 Hz, 2H), 2.8 – 2.7 (m, 1H), 2.1 – 1.9 (m, 2H), 1.8 – 1.7 (m, 2H), 1.7 – 1.6 (m, 2H), 1.5 – 1.4 (m, 2H) 1.4 – 1.3 (m, 3H), 1.2 (m, 2H).

^{13}C NMR (101 MHz, $CDCl_3$) δ [ppm] 179.70, 160.32, 156.13, 99.85, 99.76, 61.97, 37.22, 36.27, 31.00, 25.65, 25.51, 14.14.

HRMS (EI+) m/z: [M]+ calcd. for $C_{12}H_{17}NO_3$ 223.1208, found 223.1207

ethyl 5-(hex-5-yn-1-yl) isoxazole-3-carboxylate (6f)

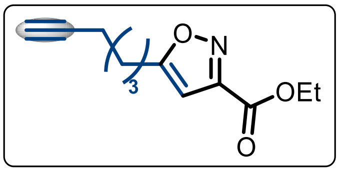

Prepared according to general procedure 7.1 on a 0.2 mmolar scale, column chromatography (EtOAc/n-hexane = 1:5) afforded the title compound as yellow liquid (23.8 mg, 54%).

^{1}H NMR (400 MHz, $CDCl_3$) δ [ppm] 6.4 (s, 1H), 4.4 (q, *J* = 7.1 Hz, 2H), 2.8 (td, *J* = 7.6, 0.8 Hz, 2H), 2.2 (td, *J* = 6.9, 2.7 Hz, 2H), 1.9 (t, *J* = 2.6 Hz, 1H), 1.8 (tt, *J* = 7.6, 6.4 Hz, 2H), 1.6 – 1.5 (m, 2H), 1.4 (t, *J* = 7.1 Hz, 3H).

^{13}C NMR (101 MHz, $CDCl_3$) δ [ppm] 175.05, 160.19, 156.39, 101.60, 83.55, 68.91, 62.07, 29.70, 27.55, 26.34, 18.04, 14.16.

HRMS (ESI+) m/z: [M+H]+ calcd. for $C_{12}H_{16}NO_3$ 222.1130, found 222.1129.

ethyl 5-(cyclohex-1-en-1-yl) isoxazole-3-carboxylate (6g)

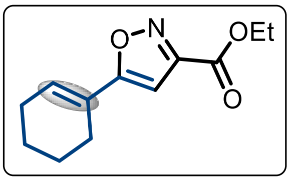

Prepared according to general procedure 7.1 on a 0.2 mmolar scale, column chromatography (EtOAc/n-hexane = 1:5) afforded the title compound as a yellow liquid (29.1 mg, 66%).

^{1}H NMR (400 MHz, $CDCl_3$) δ [ppm] 6.6 (tt, *J* = 4.0, 1.8 Hz, 1H), 6.4 (s, 1H), 4.3 (q, *J* = 7.1 Hz, 2H), 2.3 –2.1 (m, 4H), 1.7 (qq, *J* = 4.8, 2.7 Hz, 2H), 1.6 – 1.5 (m, 2H), 1.3 (t, *J* = 7.1 Hz, 3H)

^{13}C NMR (101 MHz, $CDCl_3$) δ [ppm] 172.85, 160.29, 156.43, 131.48, 124.90, 98.57, 62.05, 25.40, 25.01, 21.93, 21.55, 14.16.

HRMS (ESI+) m/z: [M+H]+ calcd. for $C_{12}H_{16}NO_3$ 222.1130, found 222.1129.

10. References

[18] a) S. Engl, O. Reiser, *ACS Catal.* **2020***, 10,* 9899-9906. b) S. Engl, O. Reiser, *Eur. J. Org. Chem.* **2020***,* 1523-1533.

[19] a) A. Granados, R. K. Dhungana, M. Sharique, J. Majhi, G. A. Molander, *Org. Lett.* **2022**, 24, *26*, 4750–4755; b) A. T. Parsons, M. J. Campbell, J. S. Johnson *Org. Lett.* **2008**, 10, *12*, 2541–2544; c) J. Li, J. Z. Chen, W. Jiao, G. Q. Wang, Y. Li, X. Cheng, G. G. Li, *J. Org. Chem.* **2016**, *81*, 9992–1000; d) M. S. Manna, S. Mukherjee, *J. Am. Chem. Soc.* **2015**, 137, *1*, 130–133.

[20] a) L. Cecchi, F. de Sarlo, F. Machetti, *Eur. J. Org. Chem.* **2006**, *2006*, 4852. b) L. Cecchi, F. de Sarlo, F. Machetti, *Chem. Eur. J.* **2008**, *14*, 7903.

[21] Y. Shen, N. Lei, C. Lu, D. Xi, X. Geng, P. Tao, Z. Su, K. Zheng, *Chem. Sci.* **2021***,12,* 15399-15406.

[22] a) F. Neese, *WIREs Comput. Mol. Sci.* **2022**, *12*; b) F. Neese, *WIREs Comput. Mol. Sci.* **2012**, *2*, 73-78.

[23] *CYLView*, C. Y. Legault, Université de Sherbrooke, **2020**.

[24] a) Y. Zhao, D. G. Truhlar, *Theor. Chem. Acc.* **2008**, *120*, 215-241; b) J. D. Dill, J. A. Pople, *J. Chem. Phys.* **1975**, *62*, 2921-2923; c) M. M. Francl, W. J. Pietro, W. J. Hehre, J. S. Binkley, M. S. Gordon, D. J. DeFrees, J. A. Pople, *J. Chem. Phys.* **1982**, *77*, 3654-3665; d) W. J. Hehre, R. Ditchfield, J. A. Pople, *J. Chem. Phys.* **1972**, *56*, 2257-2261; e) V. A. Rassolov, J. A. Pople, M. A. Ratner, T. L. Windus, *J. Chem. Phys.* **1998**, *109*, 1223-1229.

[25] H. Roth, N. Romero, D. Nicewicz, *Synlett* **2016**, *27*, 714-723.

[26] V. Barone, M. Cossi, *J. Phys. Chem. A* **1998**, *102*, 1995.

[27] *NBO 7.0*, E. D. Glendening, J, K. Badenhoop, A. E. Reed, J. E. Carpenter, J. A. Bohmann, C. M. Morales, P. Karafiloglou, C. R. Landis, and F. Weinhold,Theoretical Chemistry Institute, University of Wisconsin, Madison, **2018**.

[28] L. R. Domingo, M. Ríos-Gutiérrez, P. Pérez, *Molecules* **2016**, *21,* 748.

[29] S. Grimme, *WIREs Comput Mol Sci* **2011**, *1*, 211.

[30] J. Hioe, H. Zipse, *Org. Biomol. Chem.* **2010**, *8*, 3609-3617.

11. Appendix

11.1 NMR-Spectra of Products

^{1}H-NMR (300 MHz) of Compound 3a

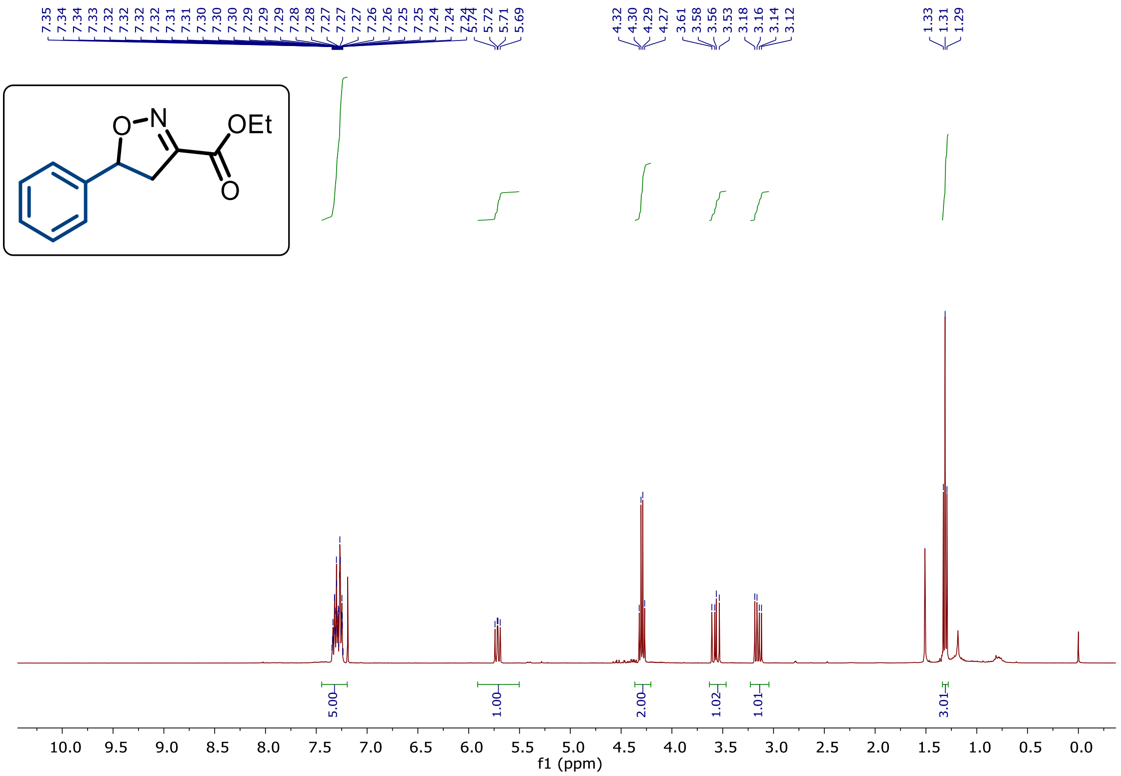

^{13}C-NMR (75 MHz) of Compound 3a

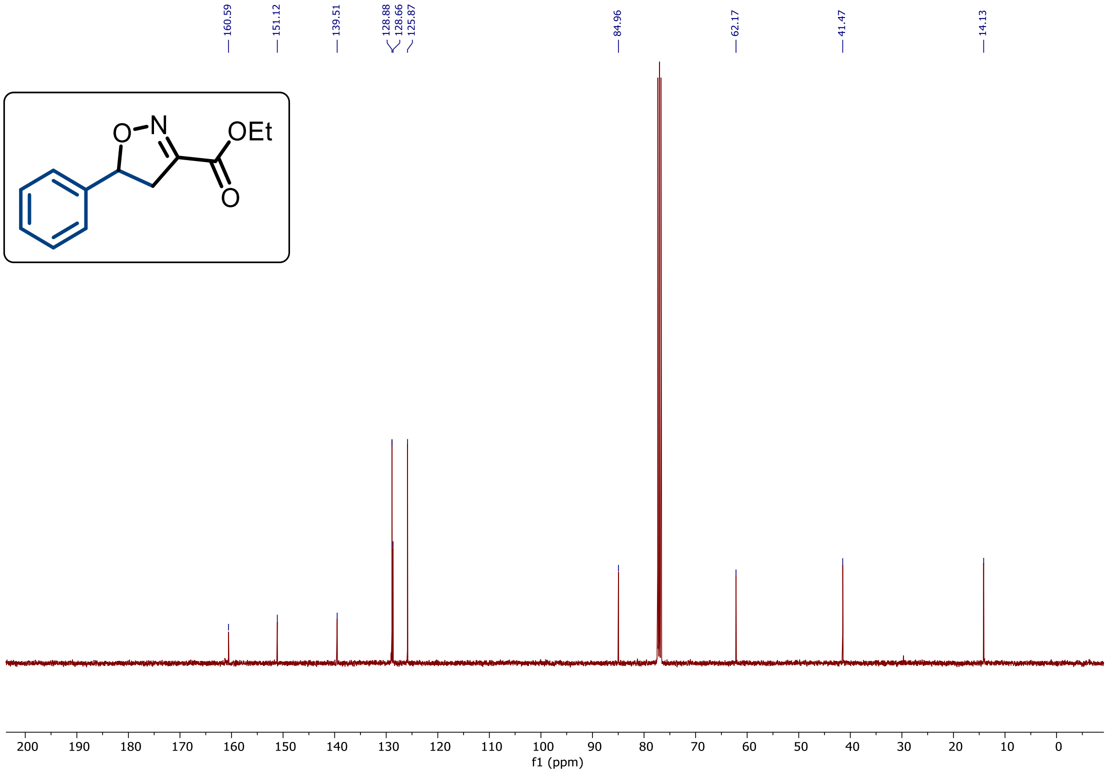

^{13}C-DEPT 135 (75 MHz) of Compound 3a

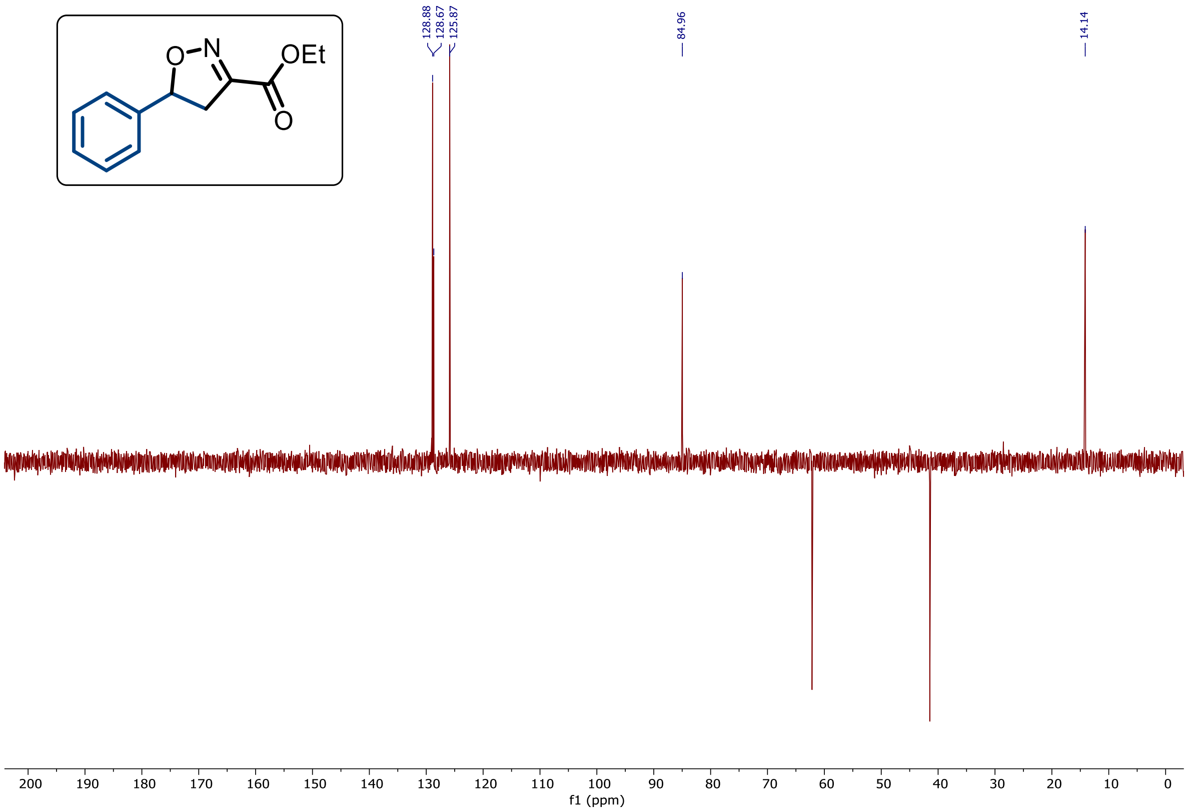

O
N
OEt
O
128.88
128.67
125.87
84.96
14.14
200
190
180
170
160
150
140
130
120
110
100
90
80
70
60
50
40
30
20
10
0
f1 (ppm)

^{1}H-NMR (300 MHz) of Compound 3b

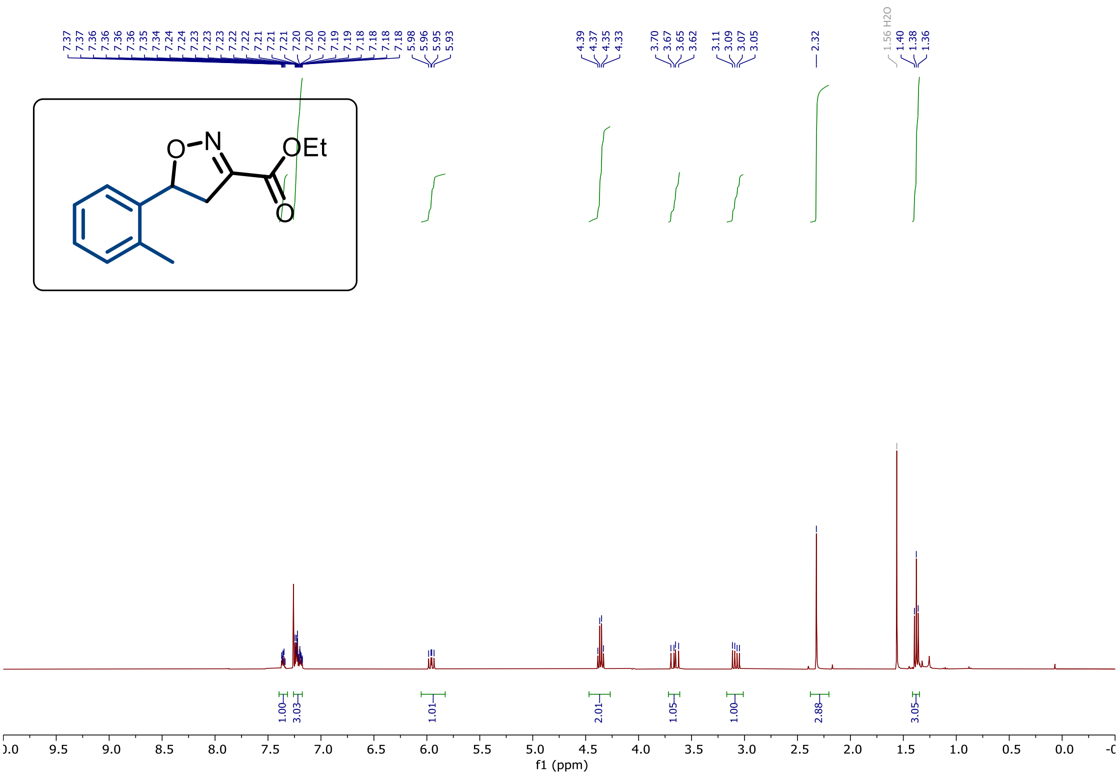

^{13}C-NMR (300 MHz) of Compound 3b

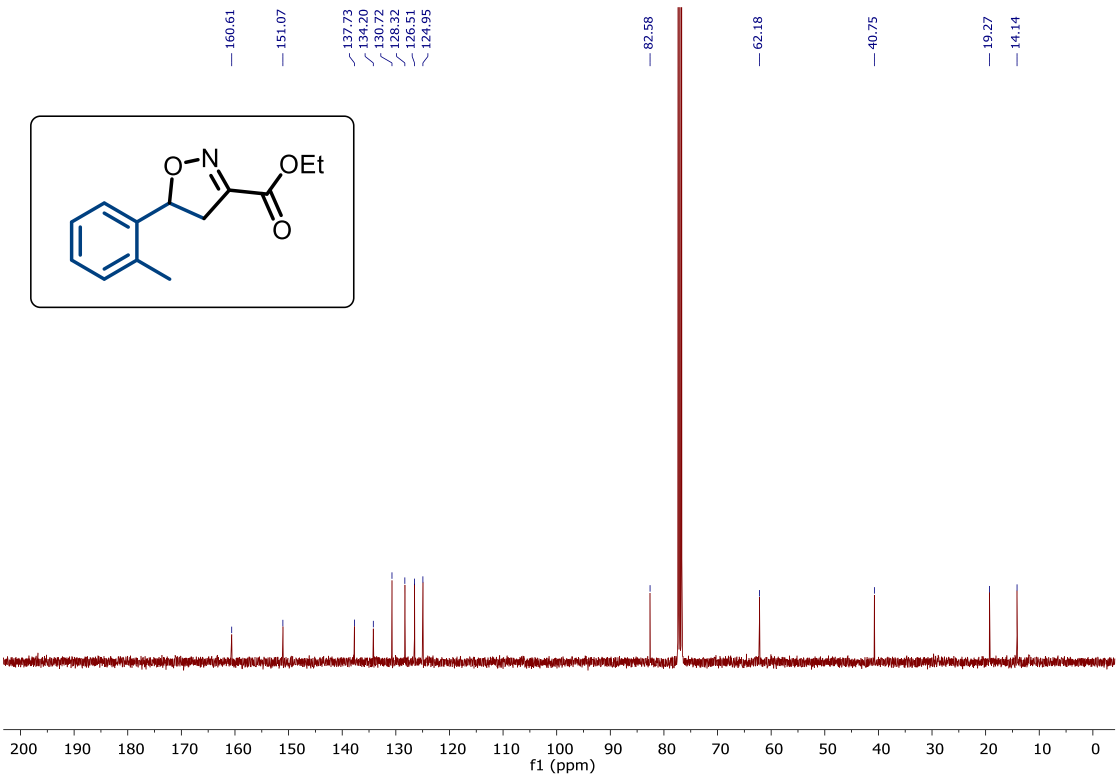

^{1}H-NMR (300 MHz) of Compound 3c

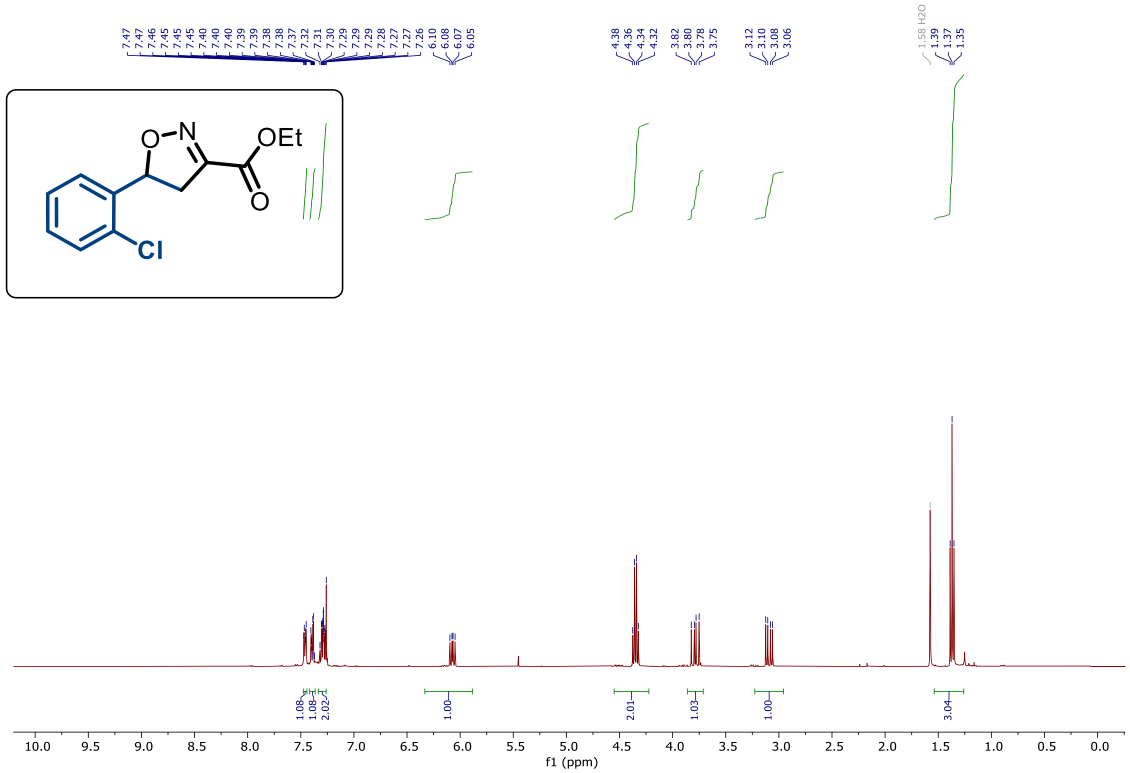

^{13}C-NMR (75 MHz) of Compound 3c

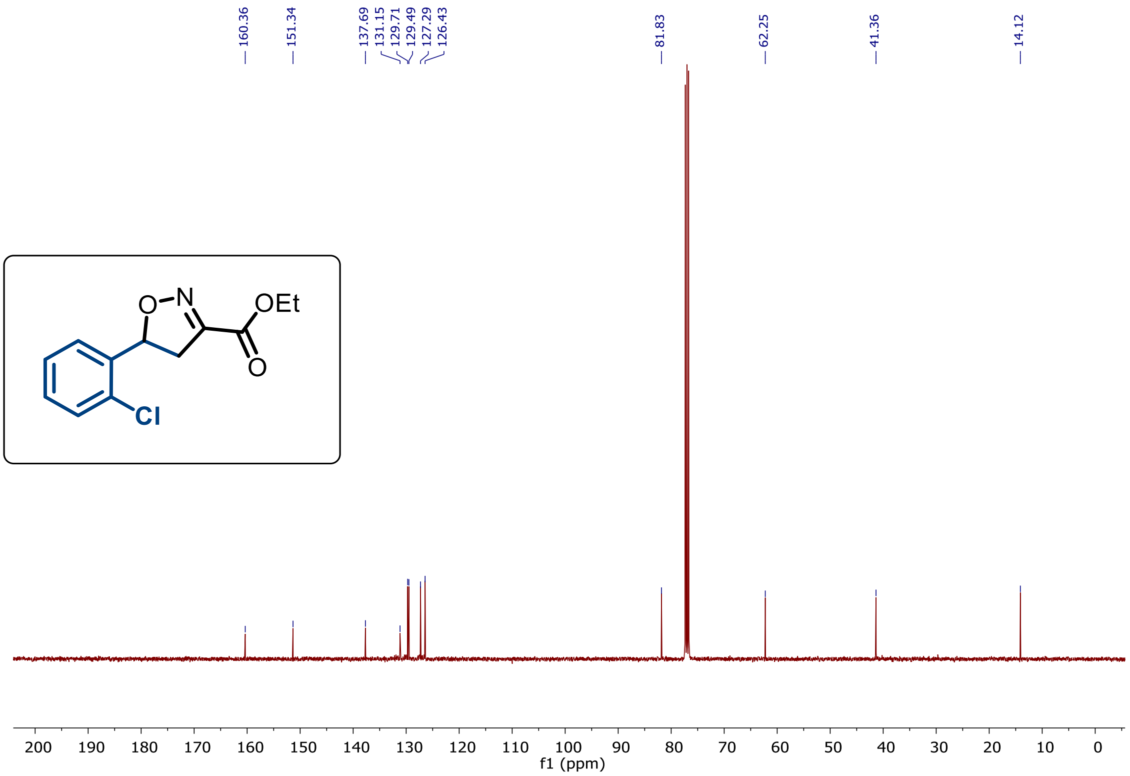

^{1}H-NMR (300 MHz) of Compound 3d

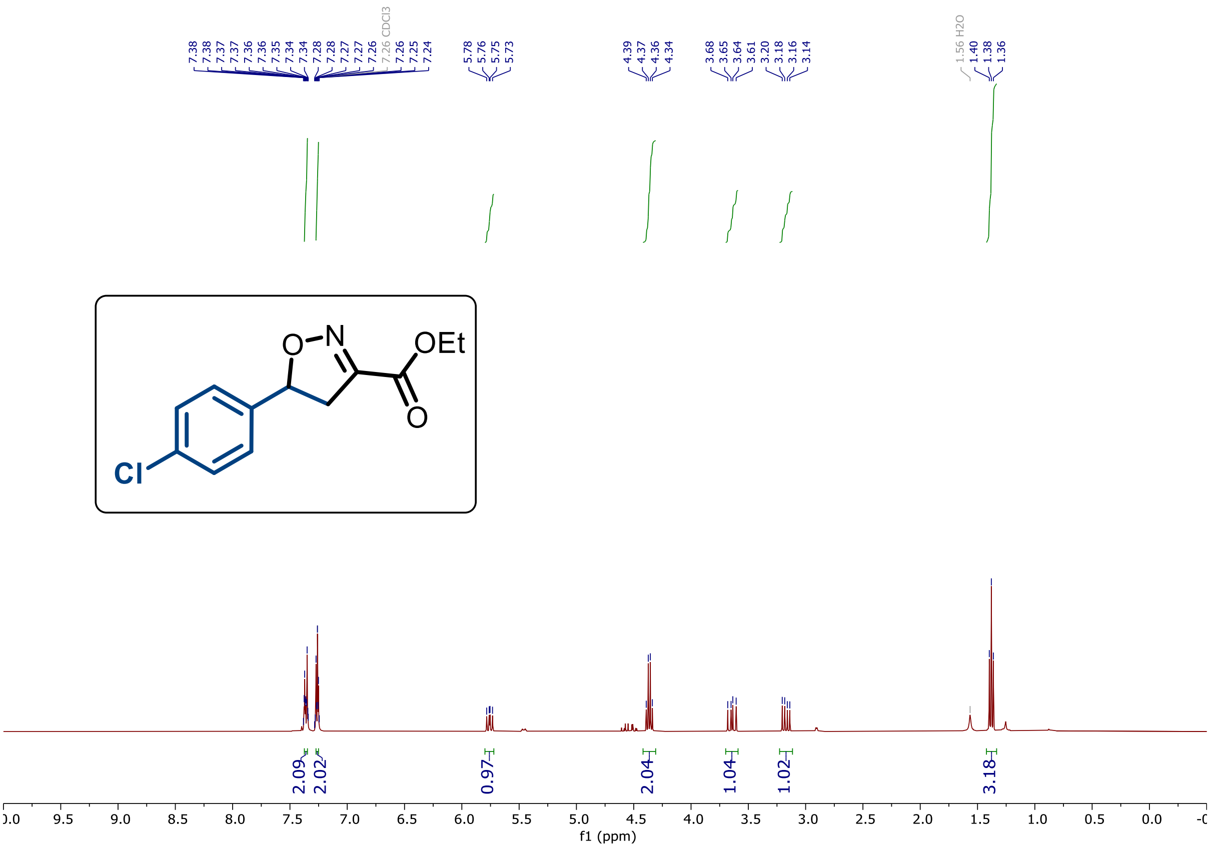

^{13}C-NMR (75 MHz) of Compound 3d

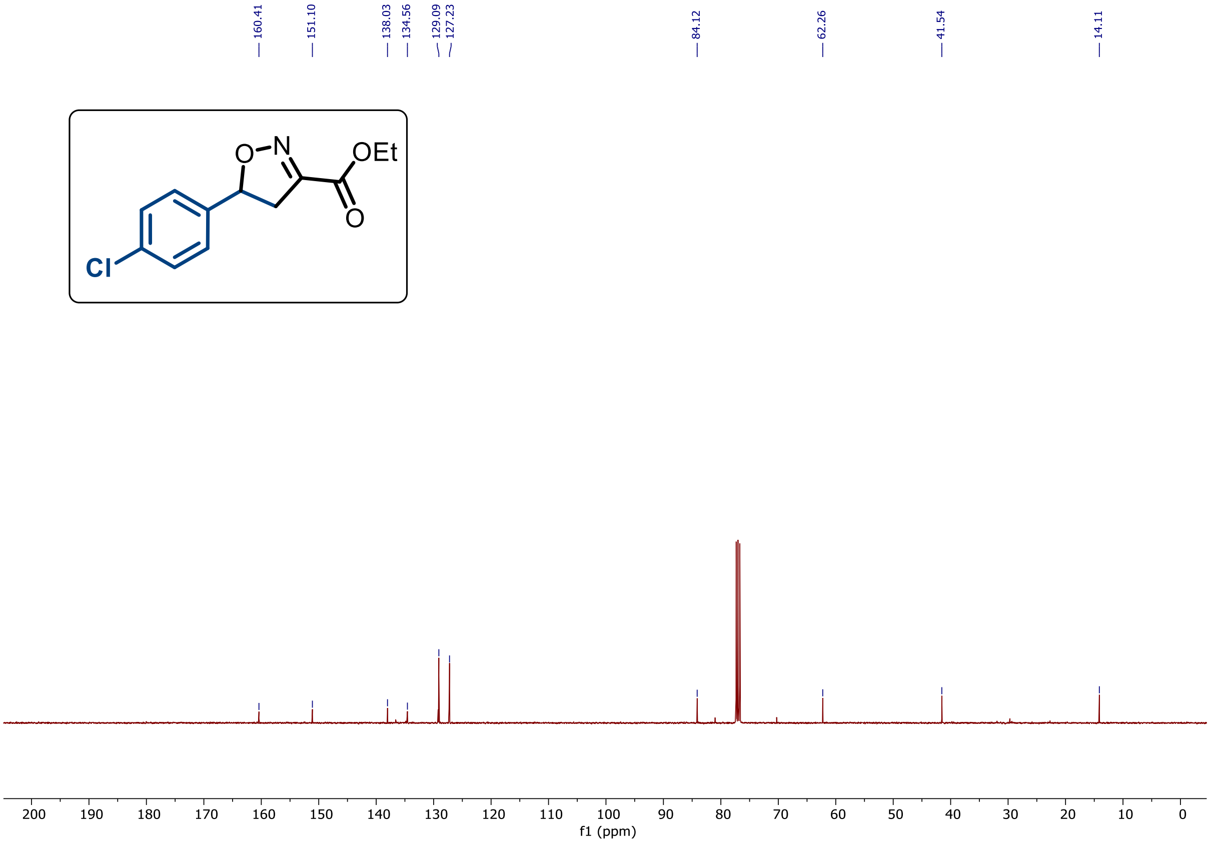

^{1}H-NMR (300 MHz) of Compound 3e

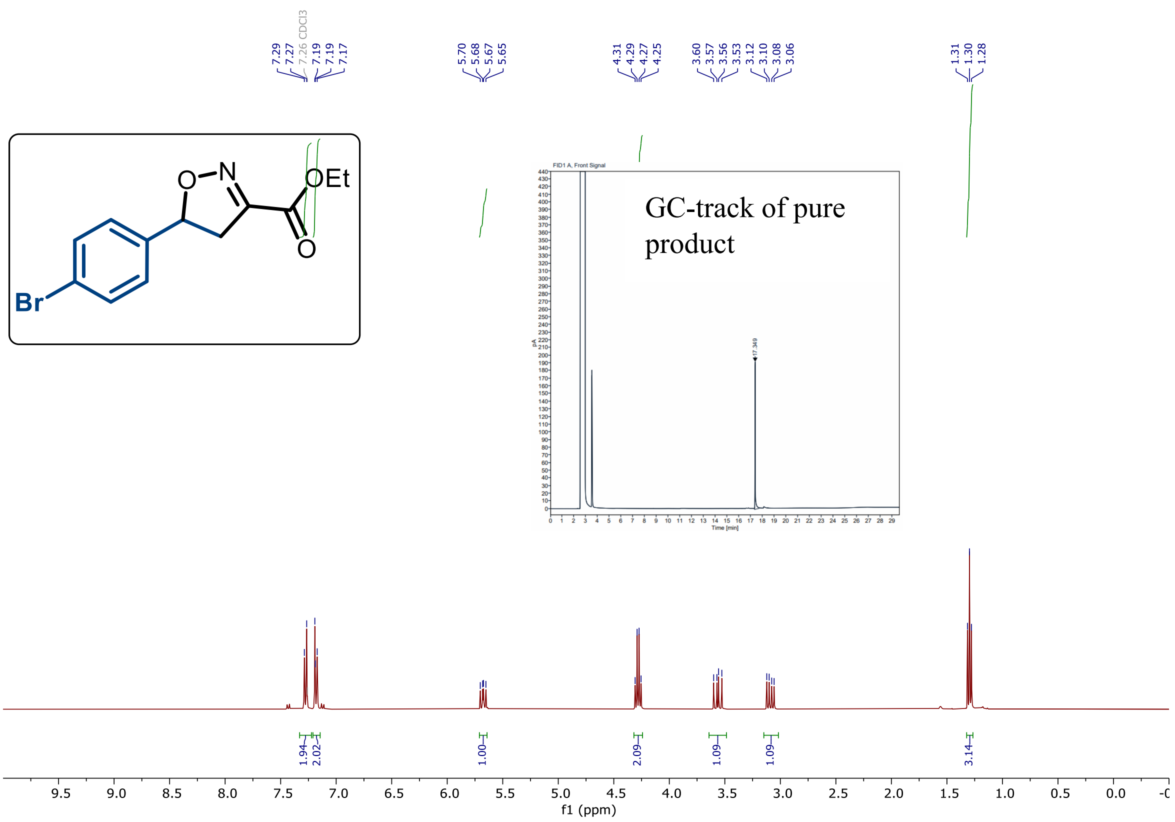

^{13}C-NMR (75 MHz) of Compound 3e

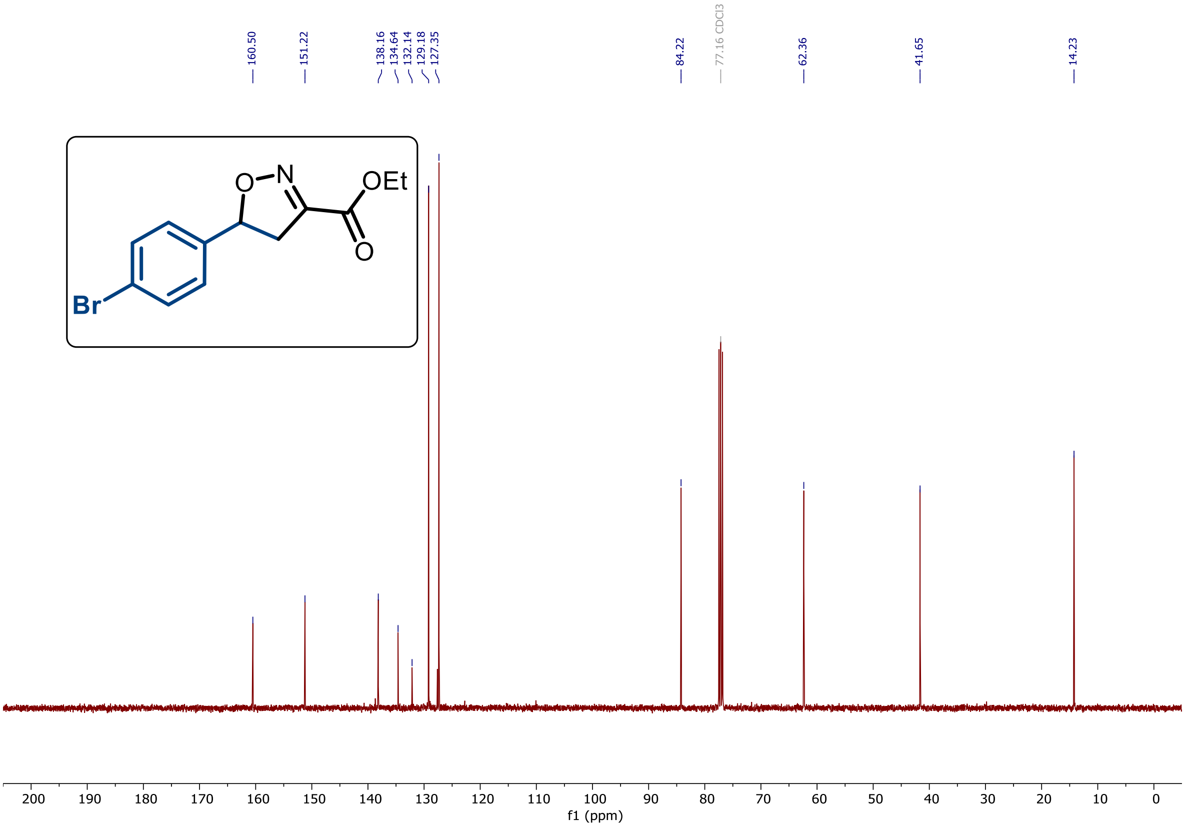

^{1}H-NMR (300 MHz) of Compound 3f

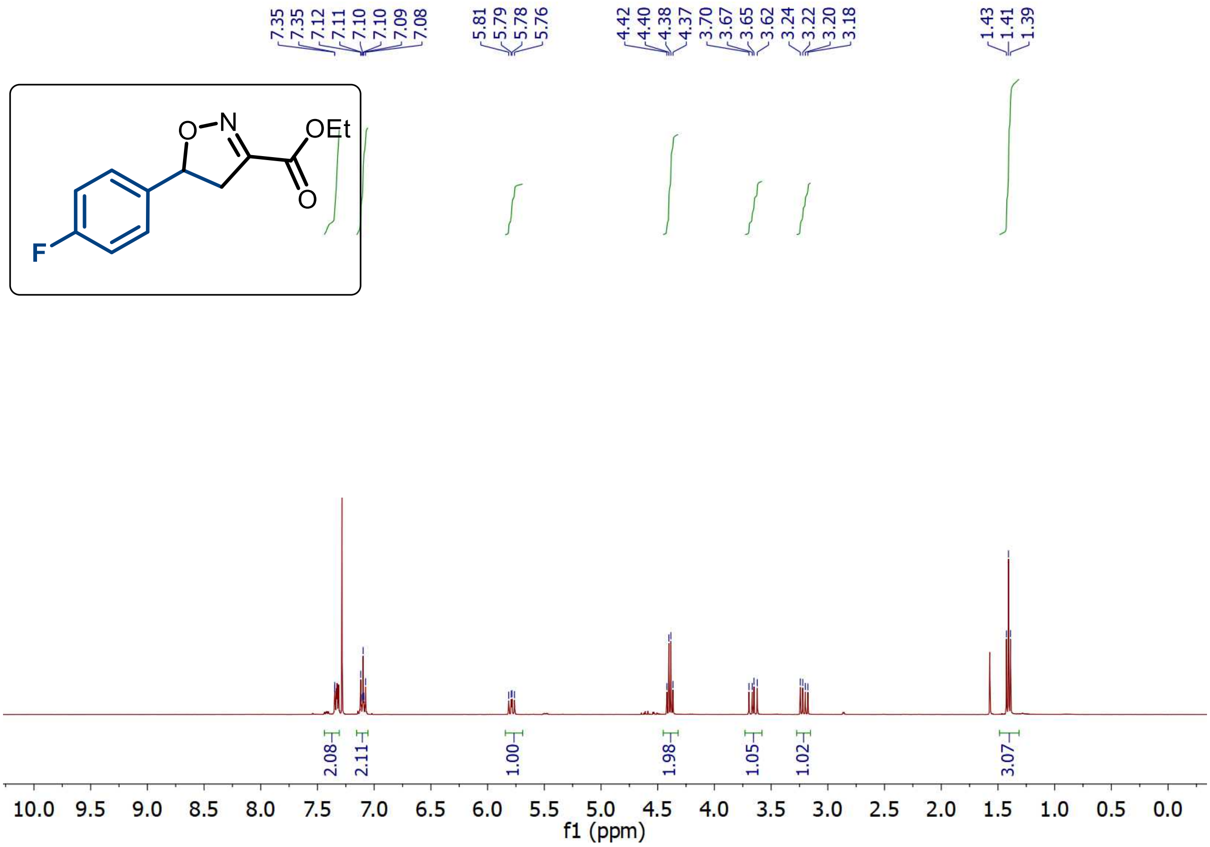

^{13}C-NMR (75 MHz) of Compound 3f

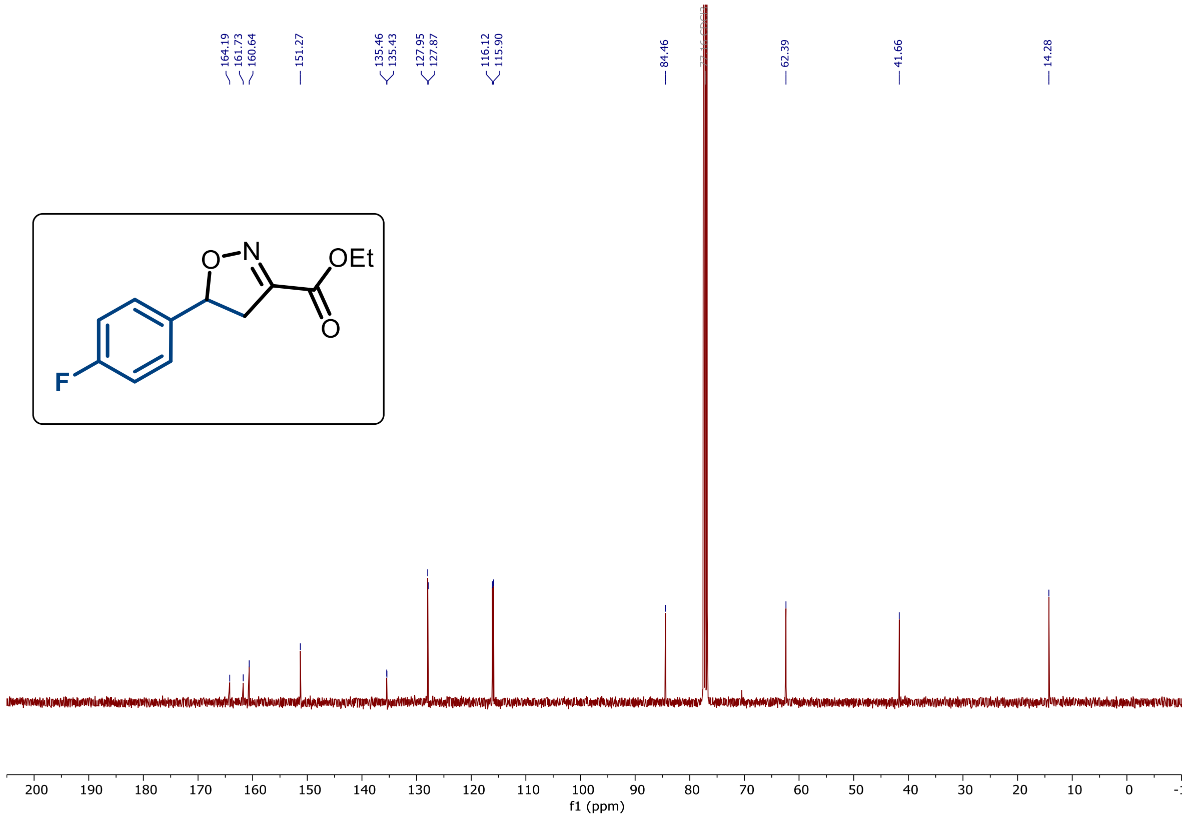

^{19}F-NMR (377 MHz) of Compound 3f

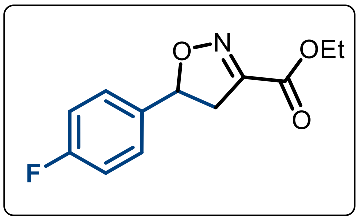

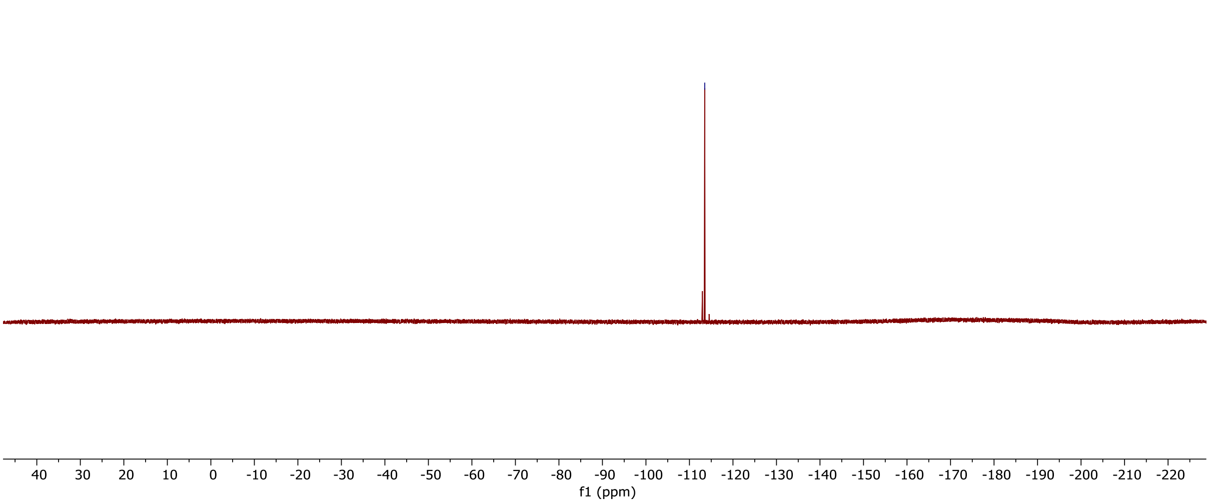

^{1}H-NMR (300 MHz) of Compound 3g

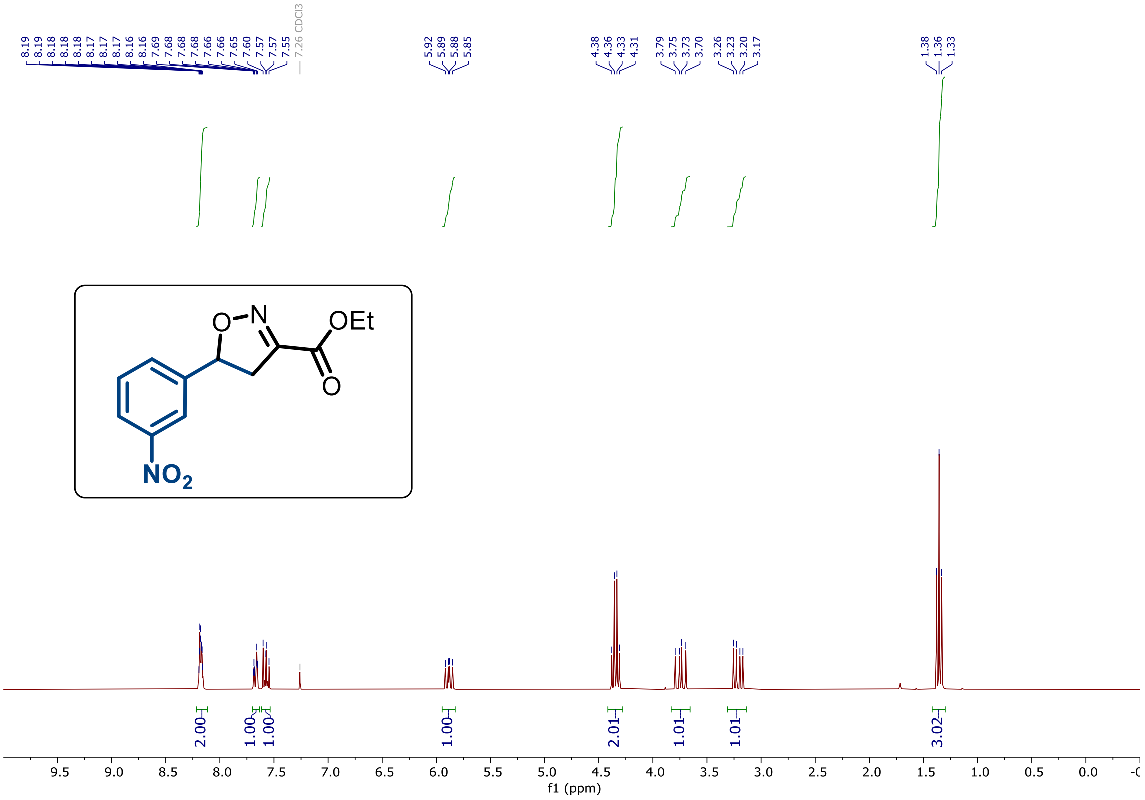

^{13}C-NMR (75 MHz) of Compound 3g

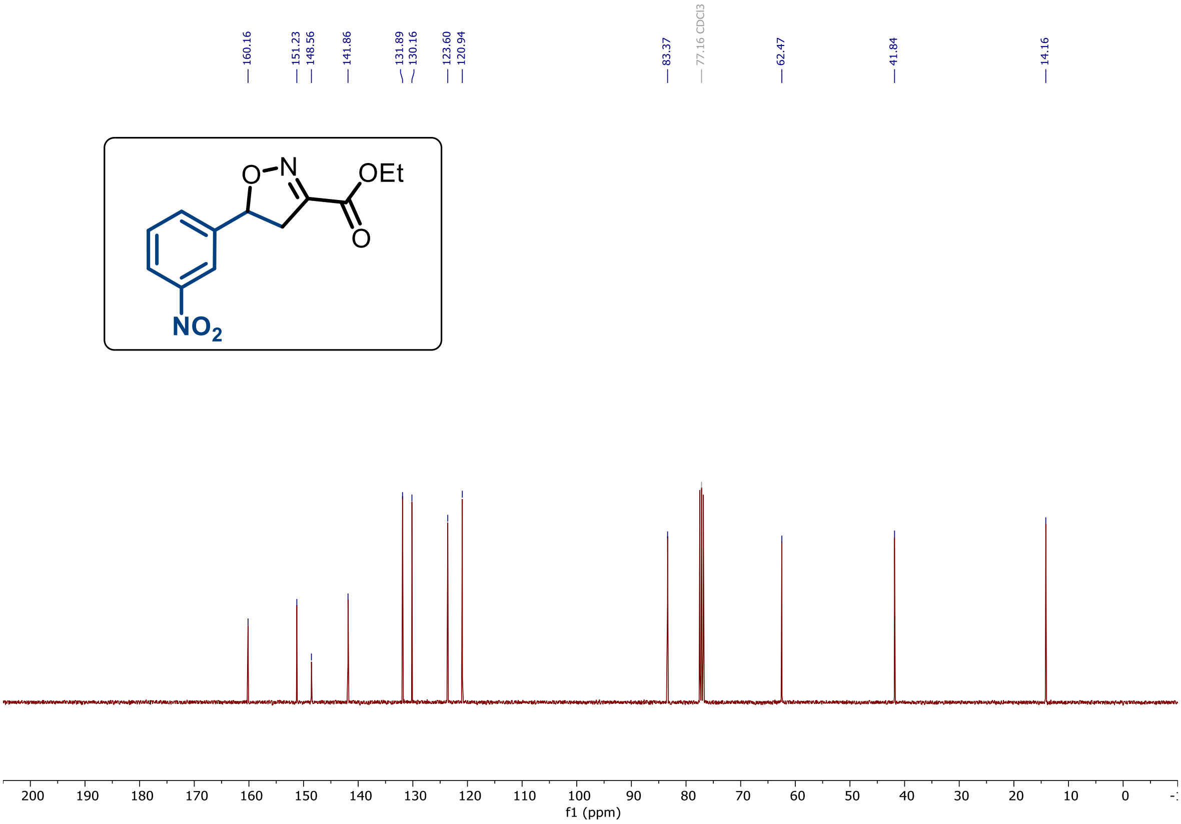

^{1}H-NMR (300 MHz) of Compound 3h

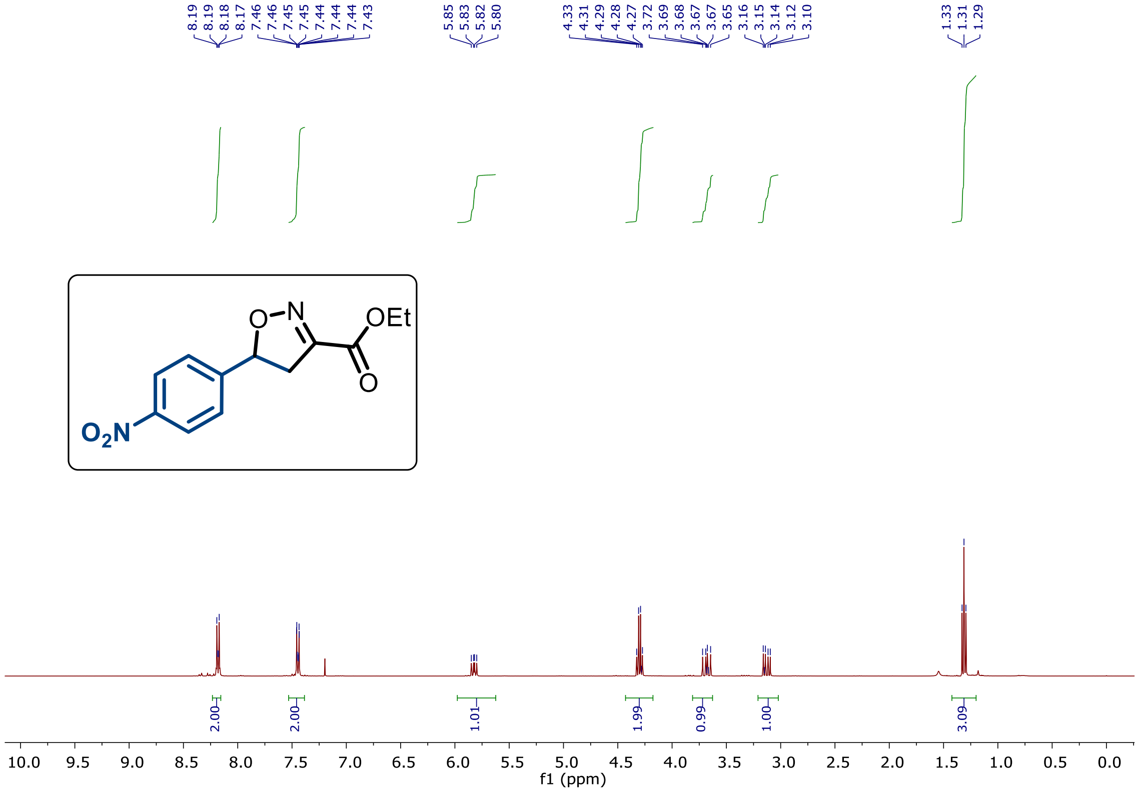

^{13}C-NMR (75 MHz) of Compound 3h

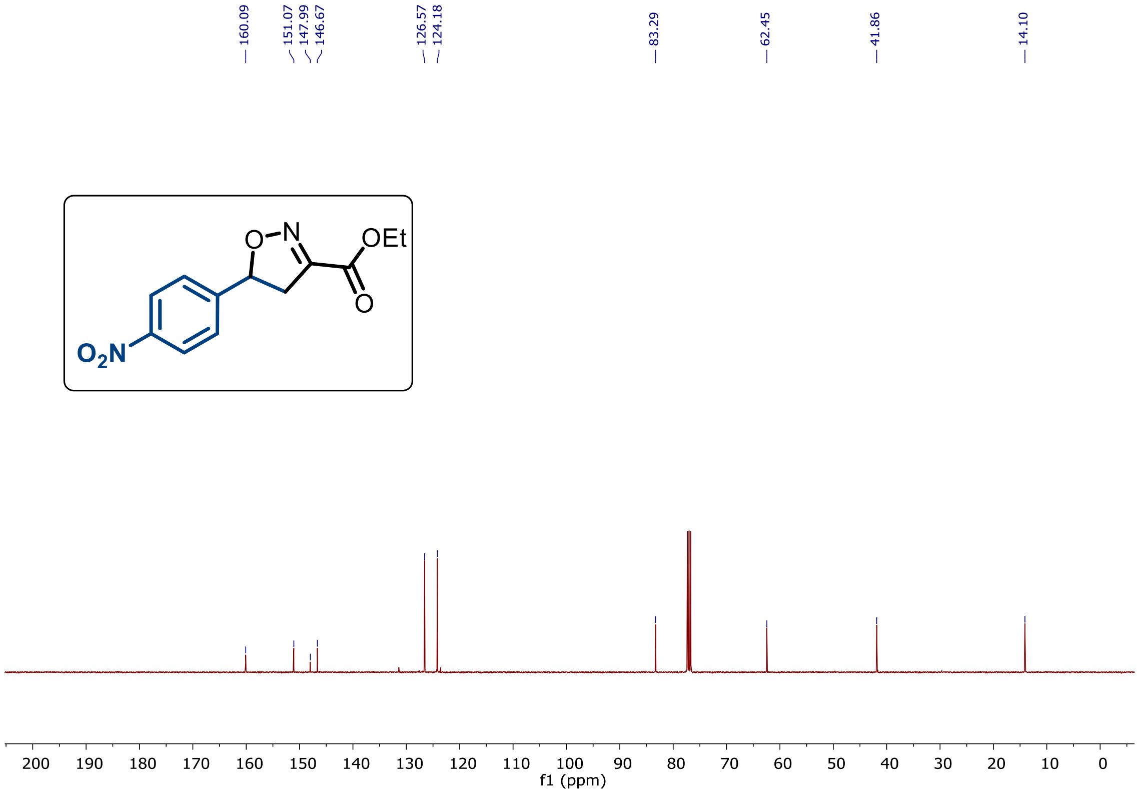

^{1}H-NMR (300 MHz) of Compound 3i

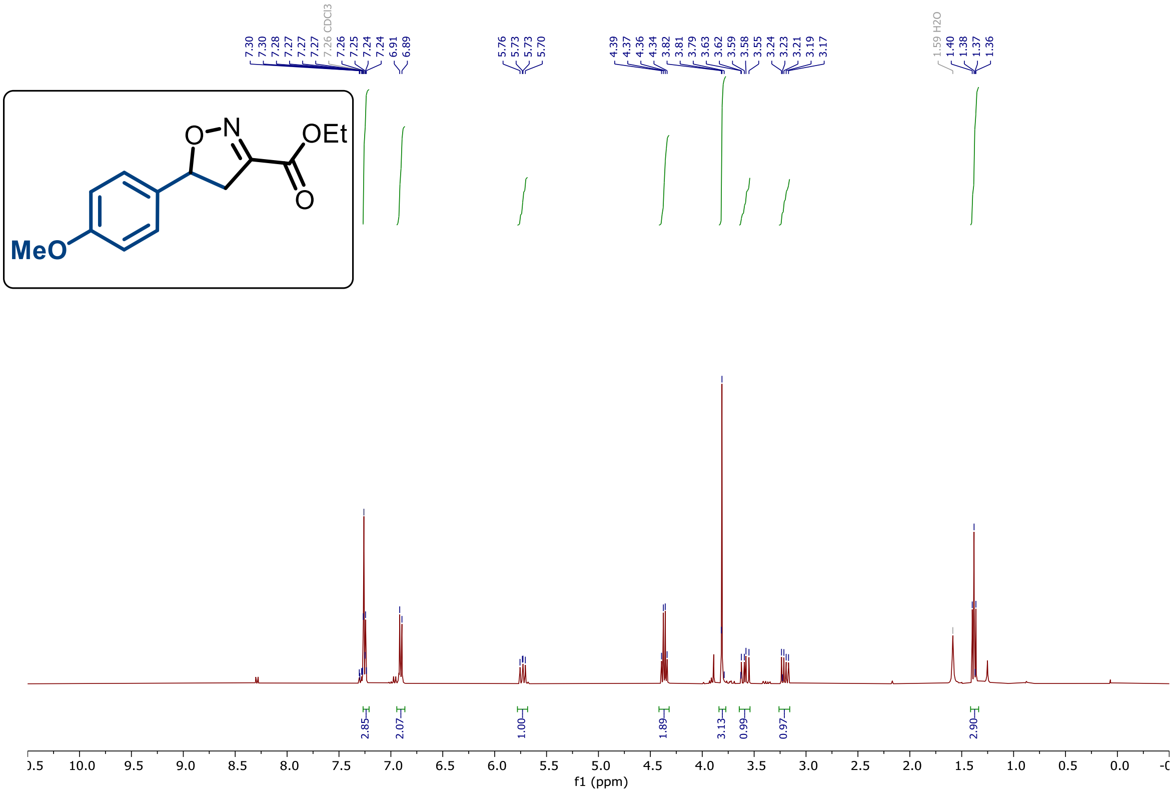

^{13}C-NMR (75 MHz) of Compound 3i

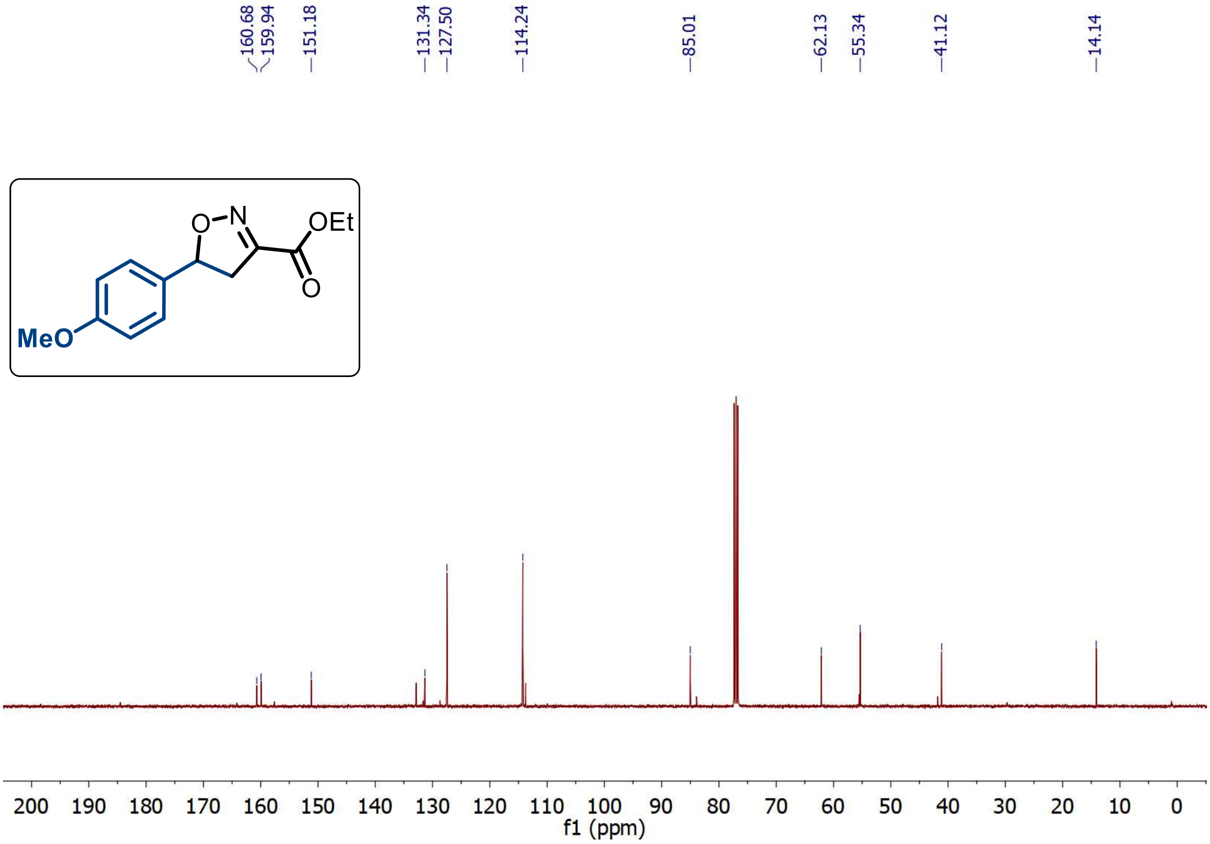

^{1}H-NMR (300 MHz) of Compound 3i showing side product.

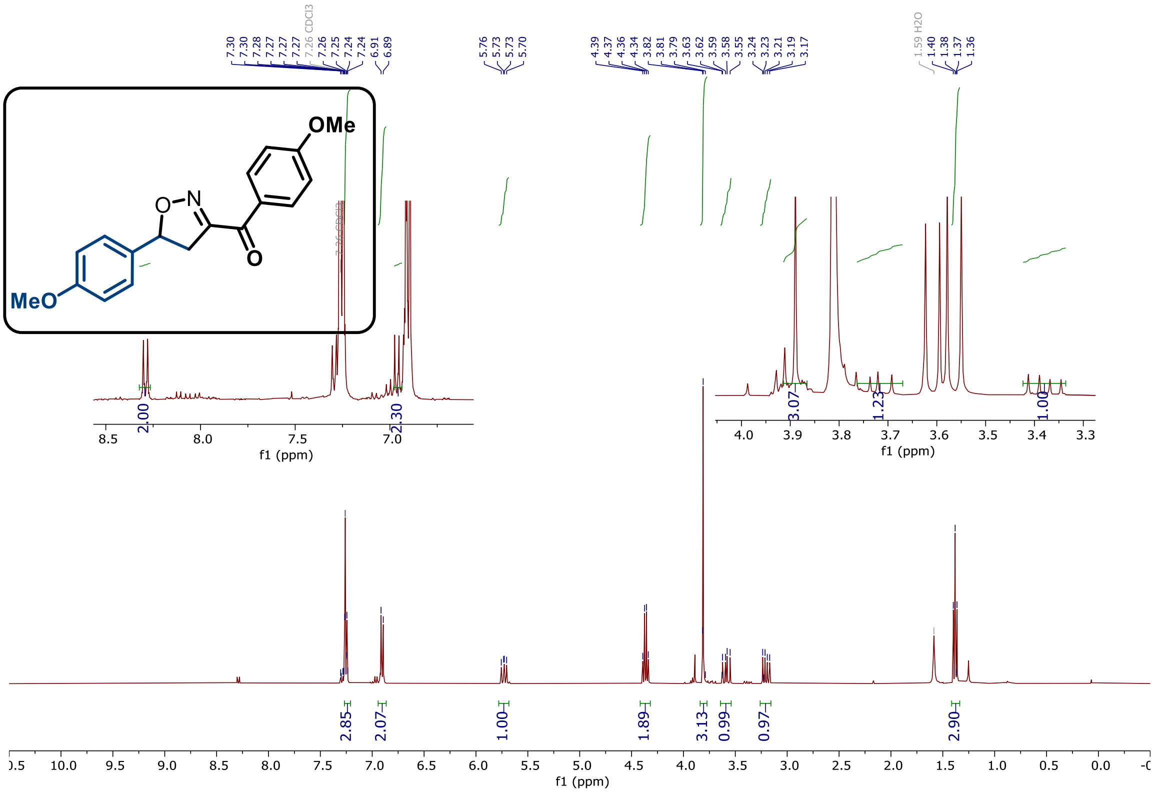

The side product (12% according to 1H NMR) is formed from the electron rich 4-methoxystyrene, which is cleaved under the reaction conditions (in part) to 4-methoxybenzaldehyde that undergoes a nitroaldol reaction with ethyl nitro acetate following decarboxylation. The resulting 1-(4-methoxyphenyl)-2-nitroethan-1-one undergoes in an analogous way to ethylnitroacetate the cycloaddition to give rise to the side product.

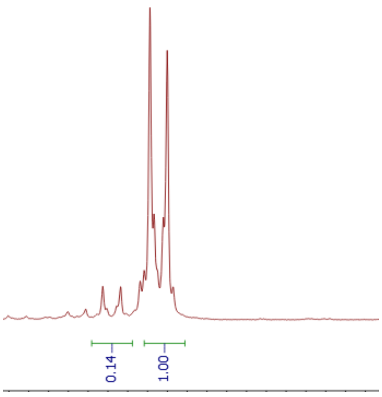

^{1}H-NMR (300 MHz) of Compound 3j

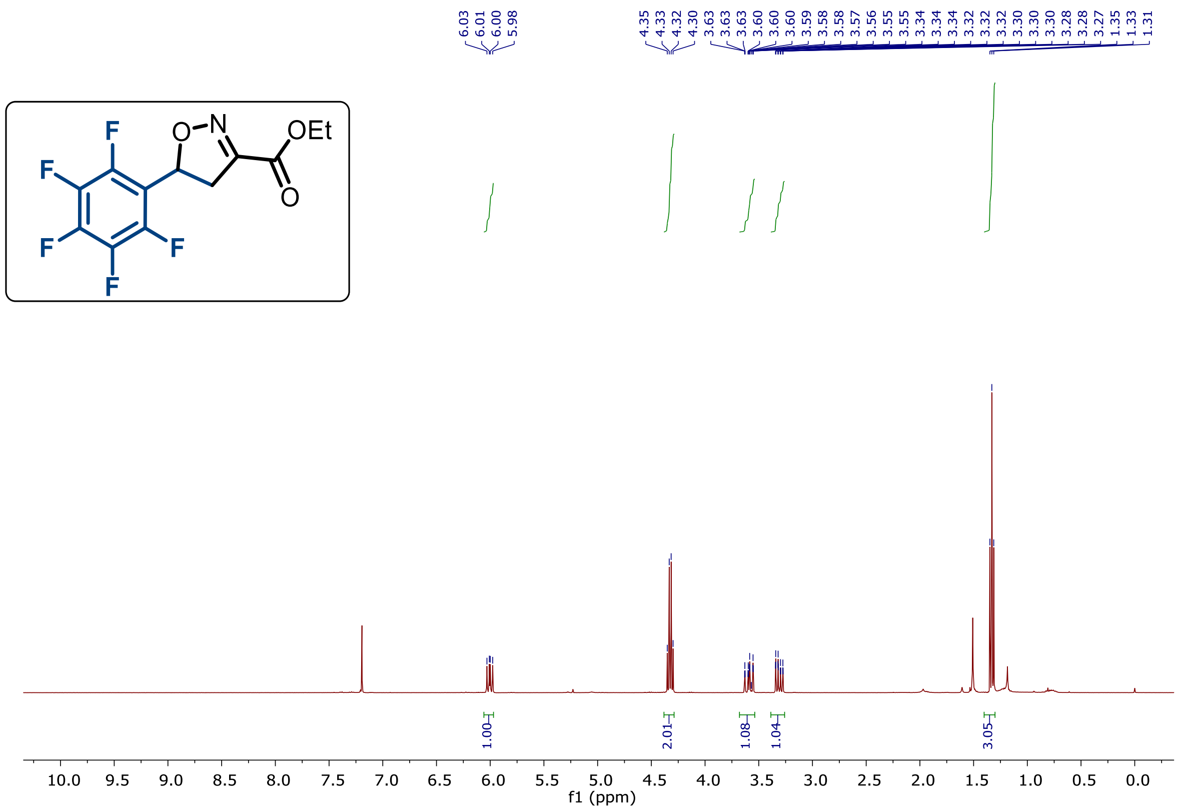

^{13}C-NMR (75 MHz) of Compound 3j

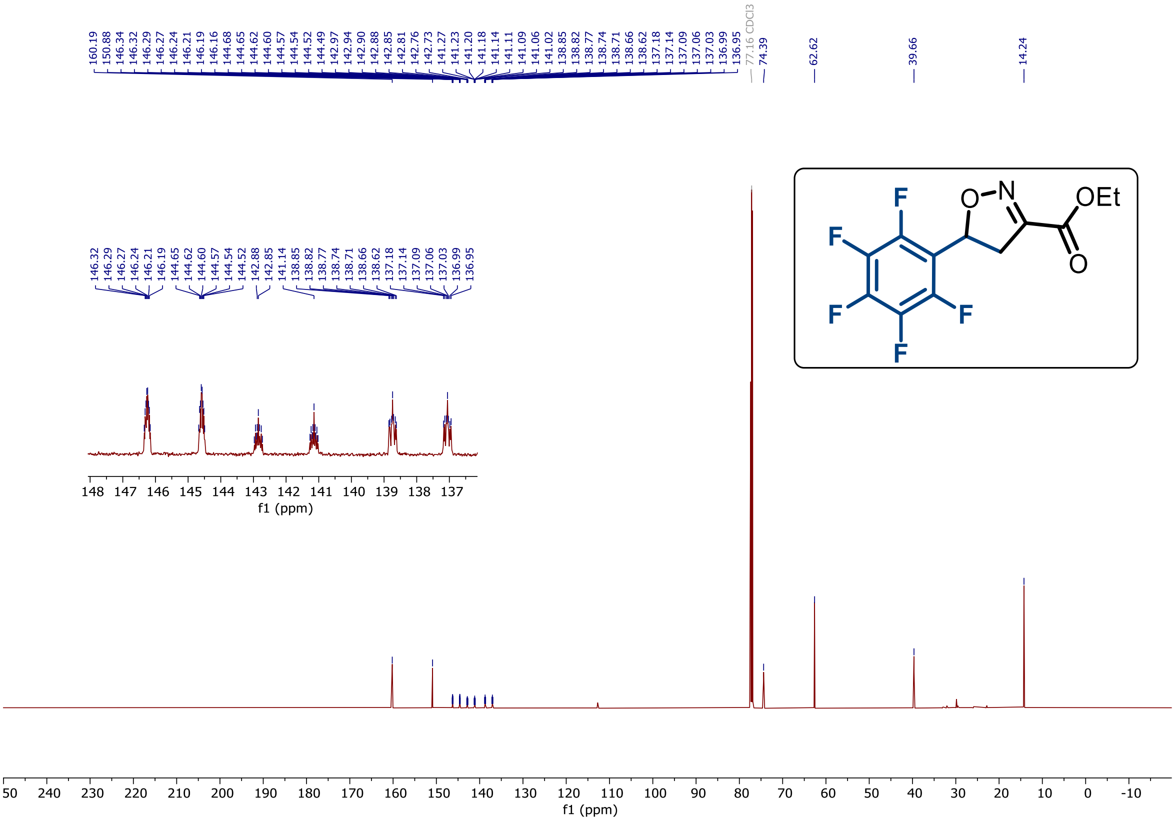

^{19}F-NMR (377 MHz) of Compound 3j

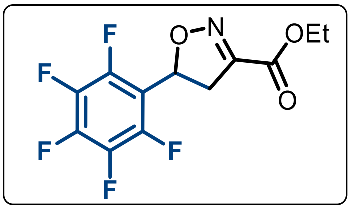

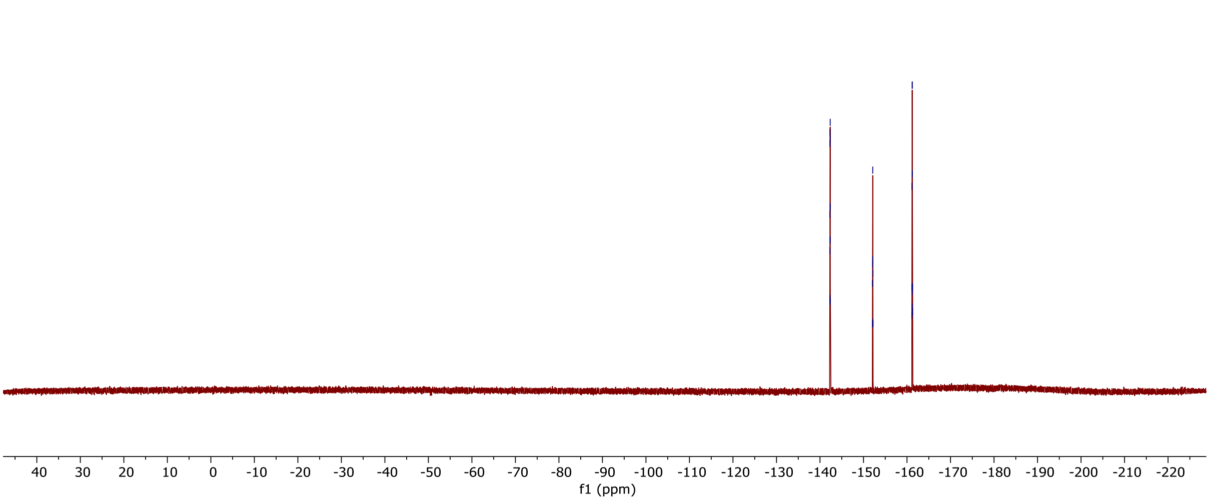

^{1}H-NMR (300 MHz) of Compound 3k

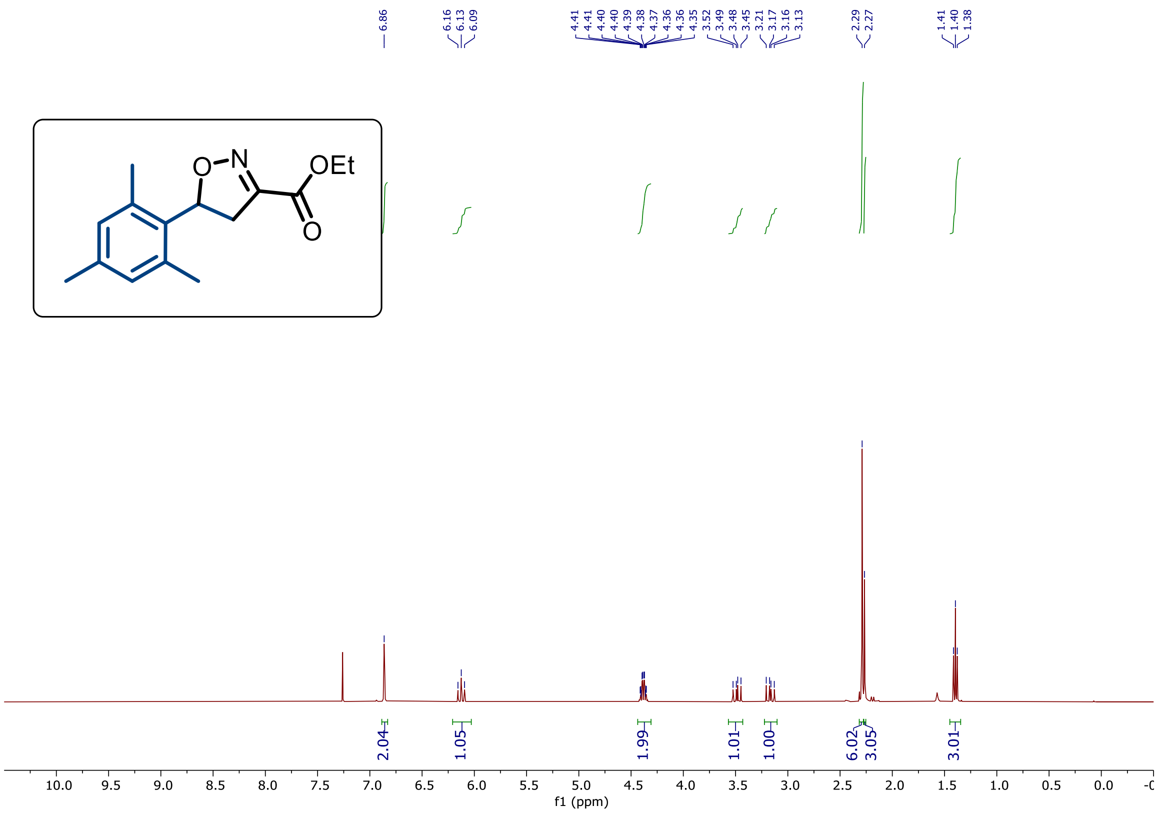

^{13}C-NMR (75 MHz) of Compound 3k

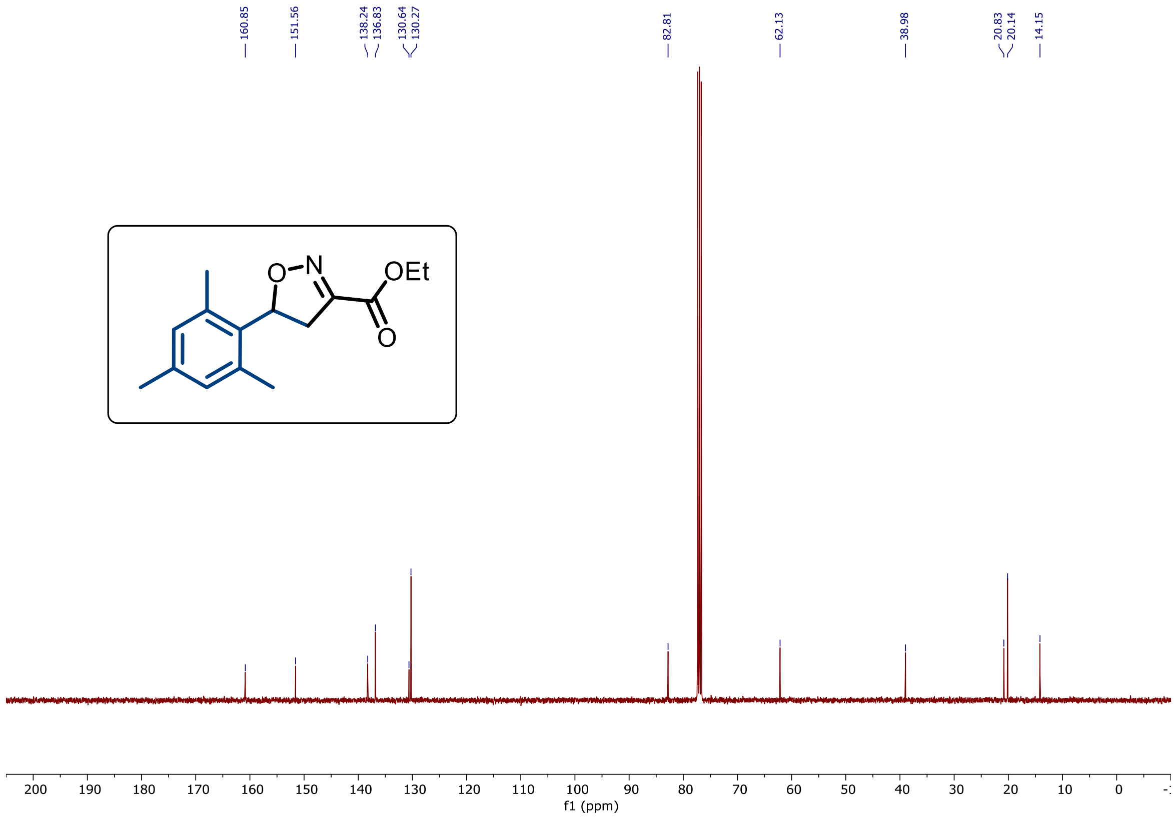

^{1}H-NMR (300 MHz) of Compound 3l

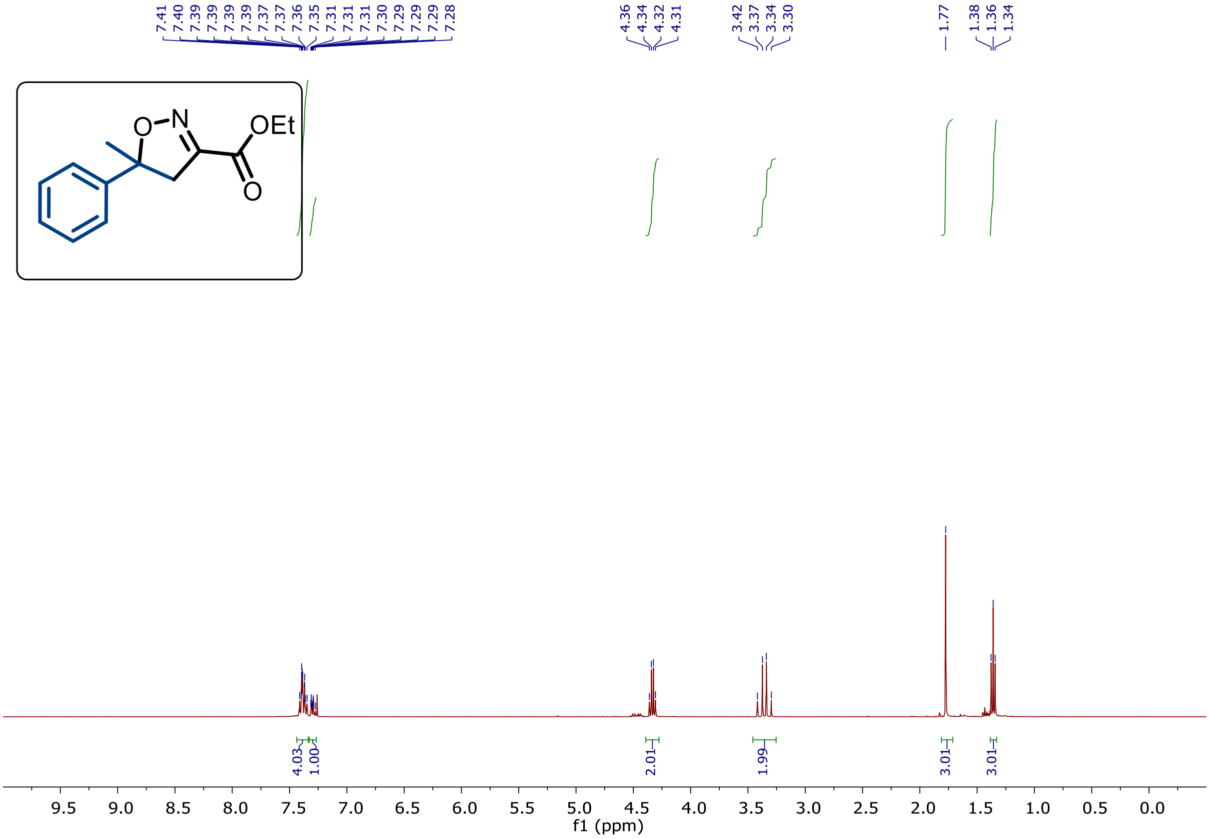

^{13}C-NMR (75 MHz) of Compound 3l

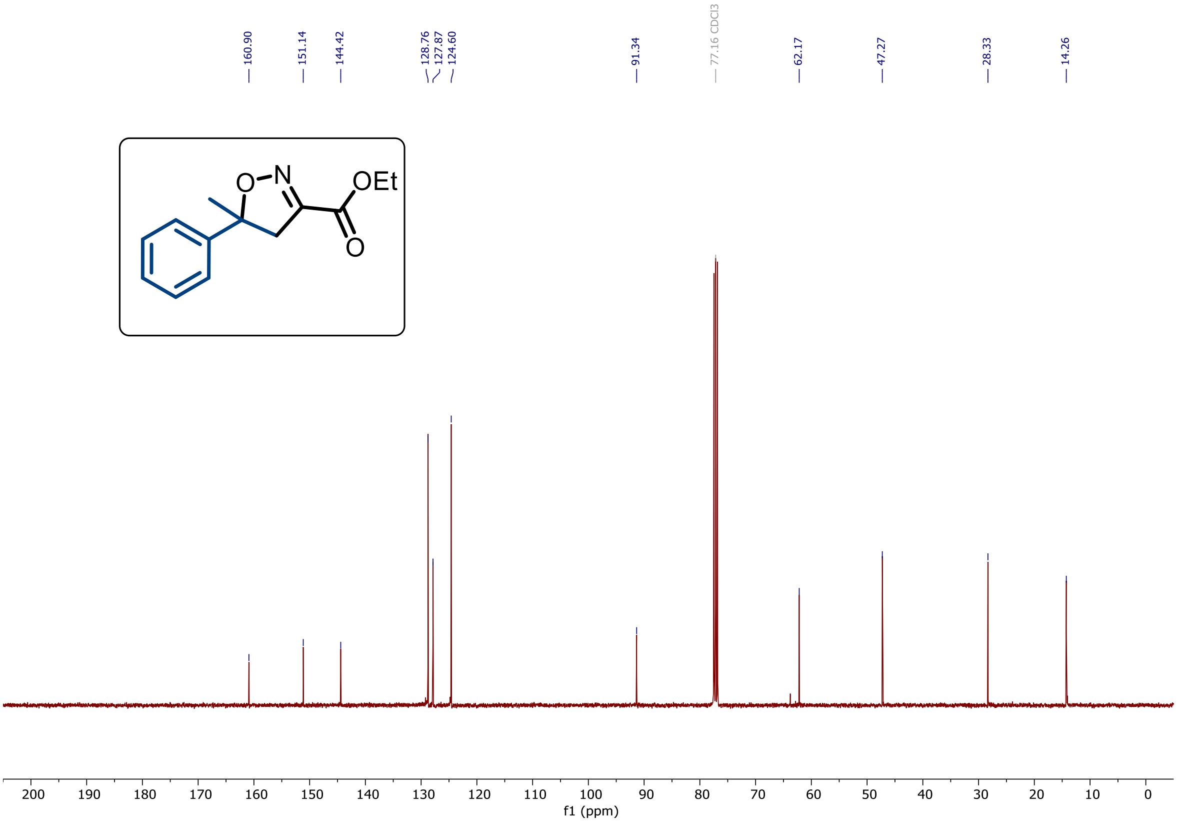

^{1}H-NMR (300 MHz) of Compound 3m

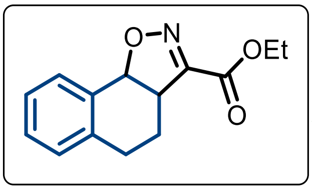

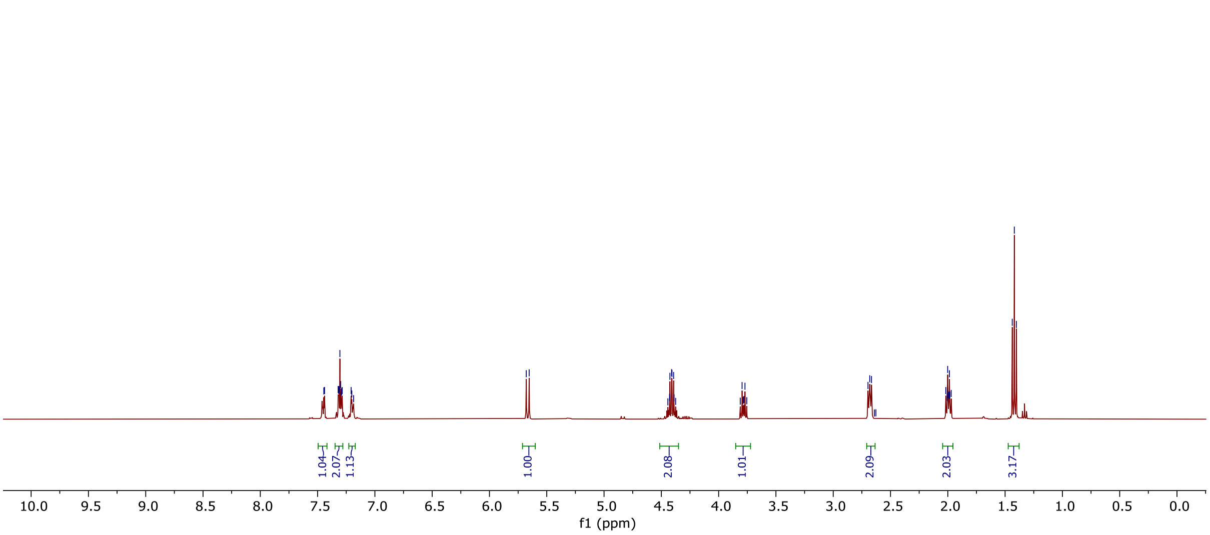

^{13}C-NMR (75 MHz) of Compound 3m

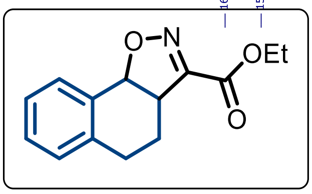

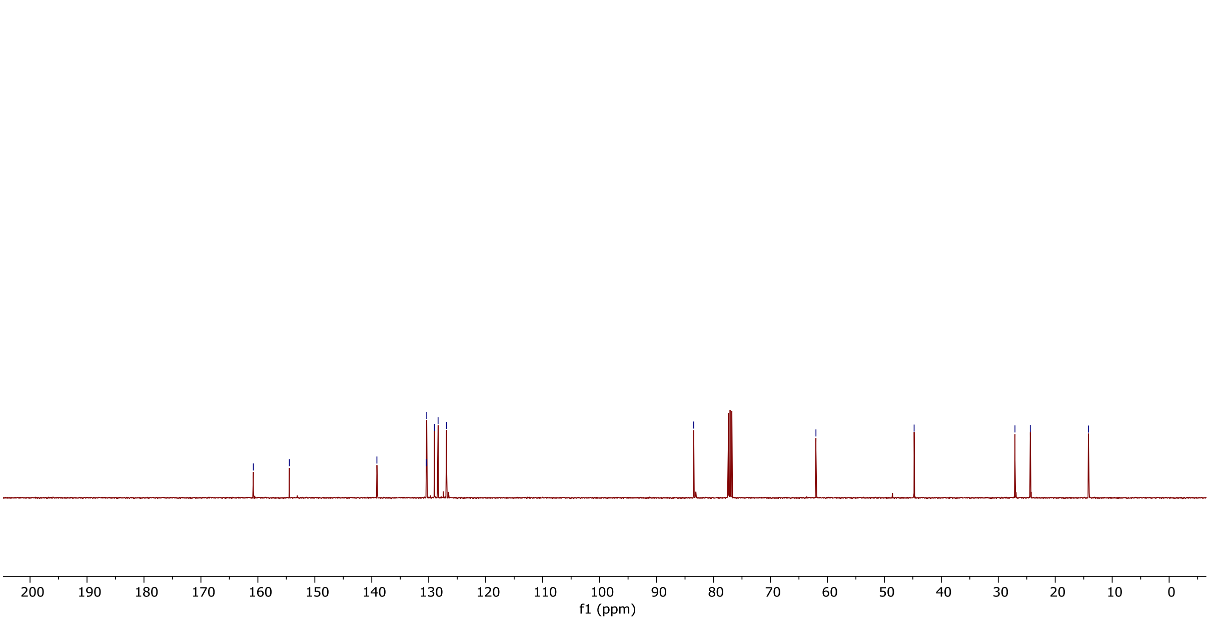

^{1}H-NMR (500 MHz) of Compound 3n

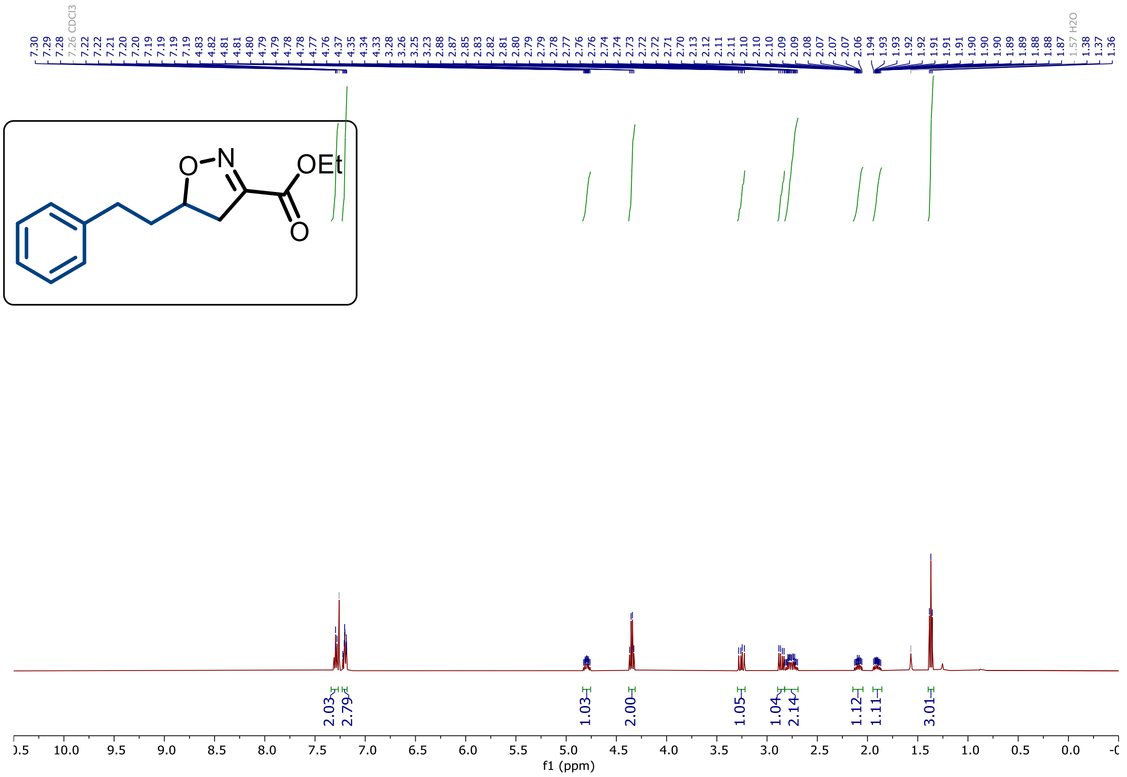

^{13}C-NMR (126 MHz) of Compound 3n

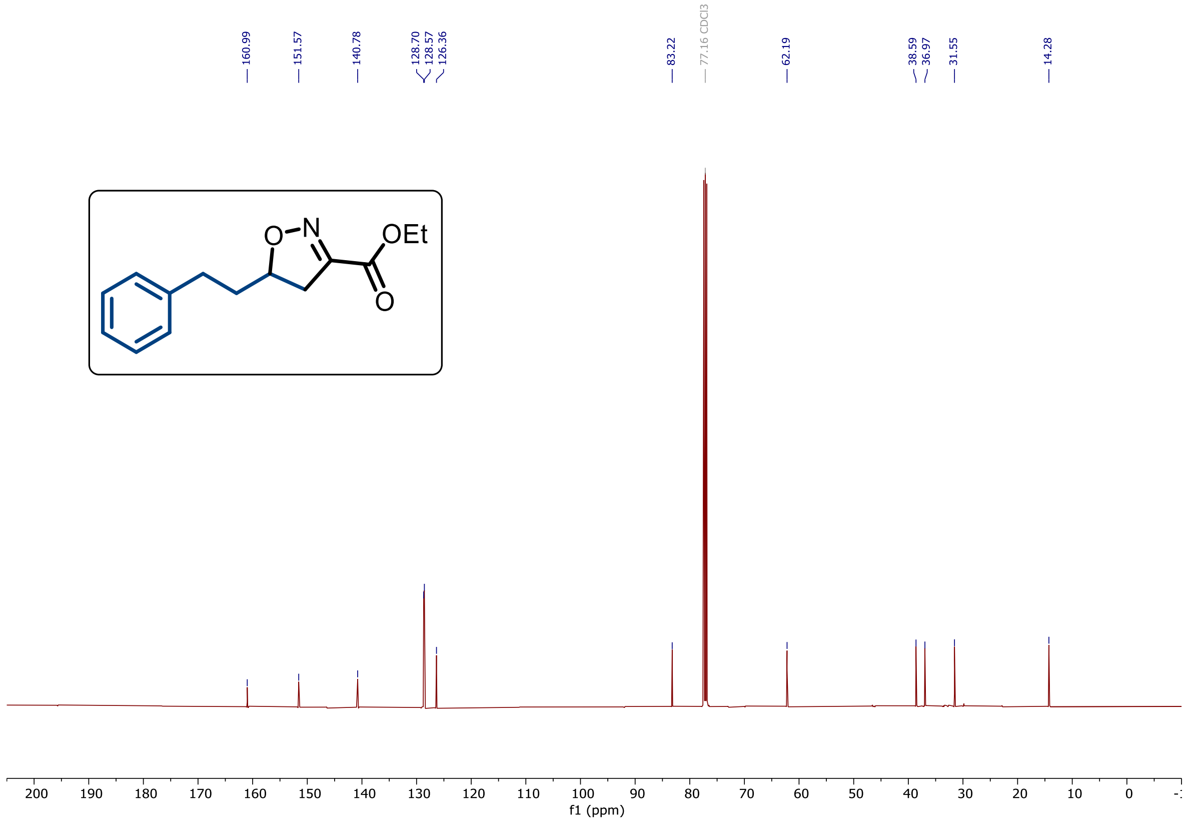

^{1}H-NMR (300 MHz) of Compound 3o

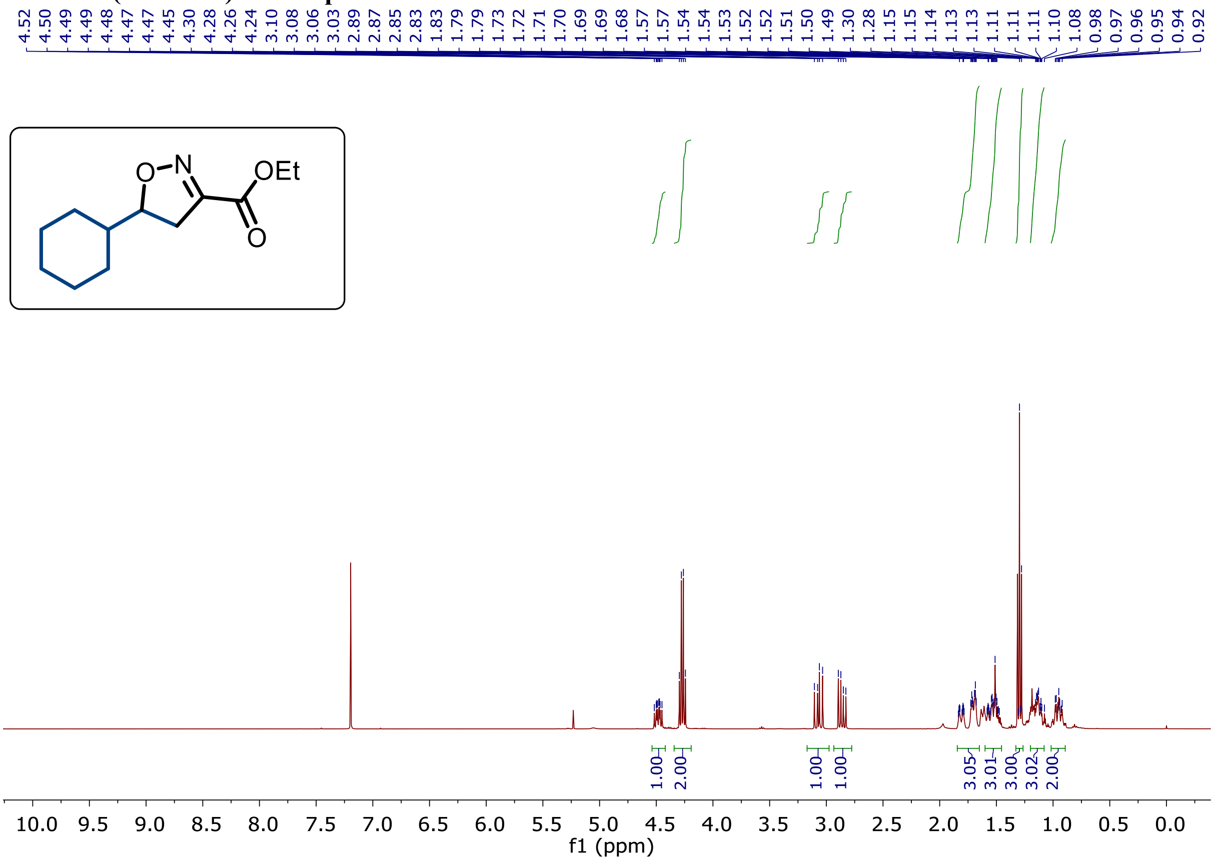

^{13}C-NMR (75 MHz) of Compound 3o

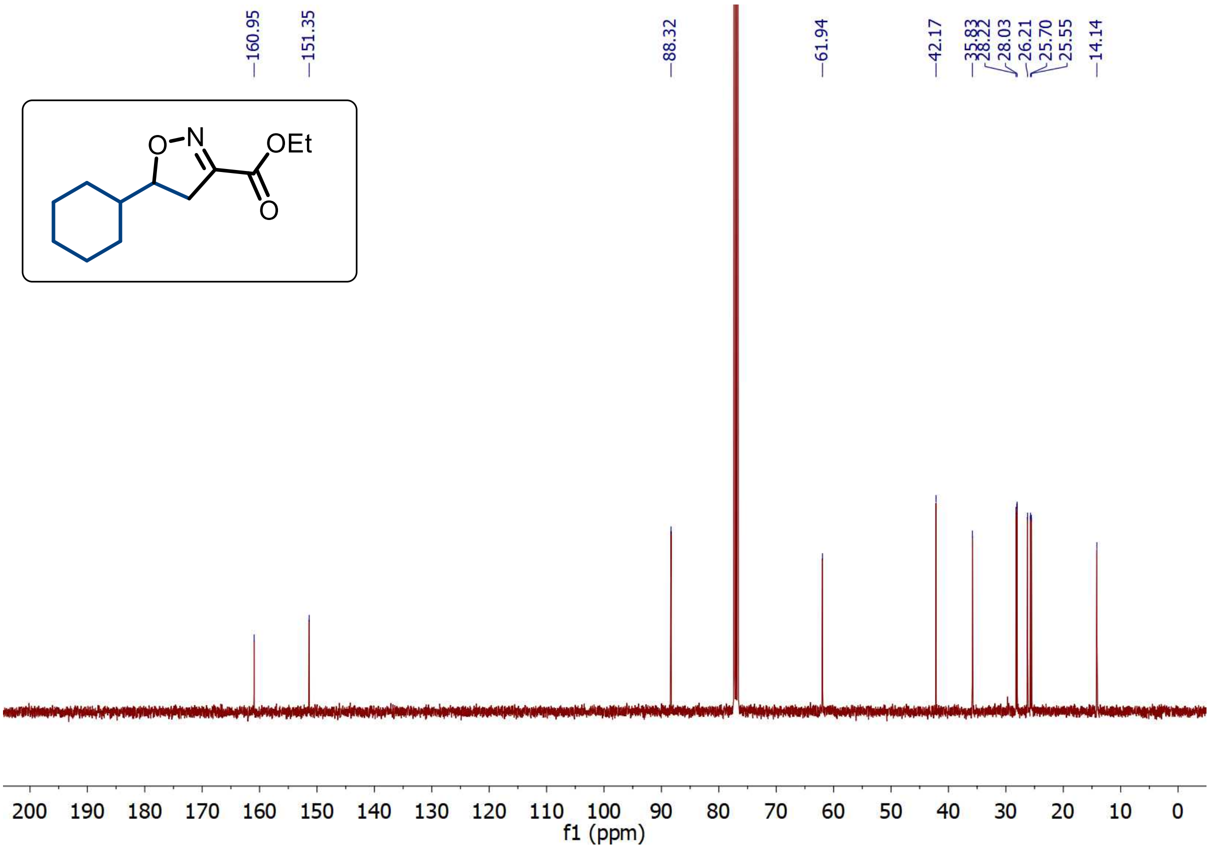

^{1}H-NMR (300 MHz) of Compound 3p

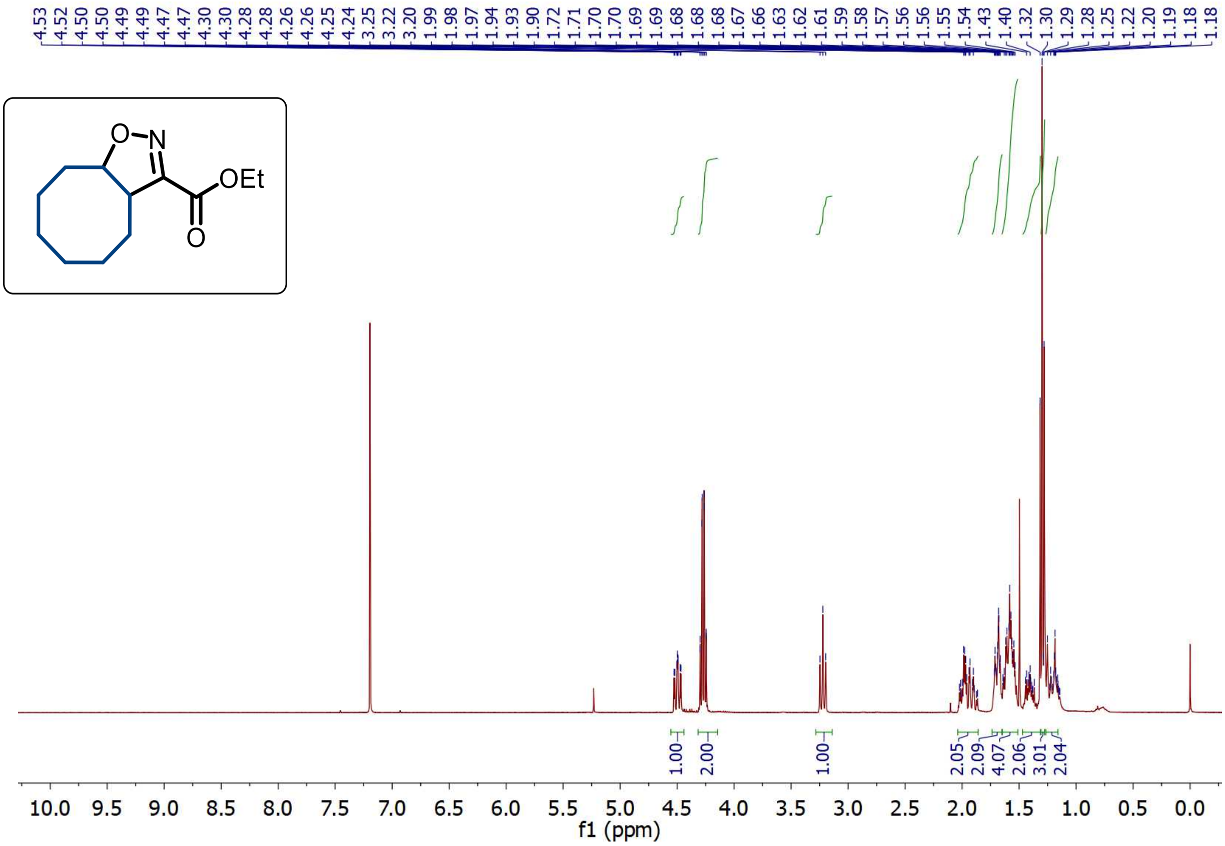

^{13}C-NMR (75 MHz) of Compound 3p

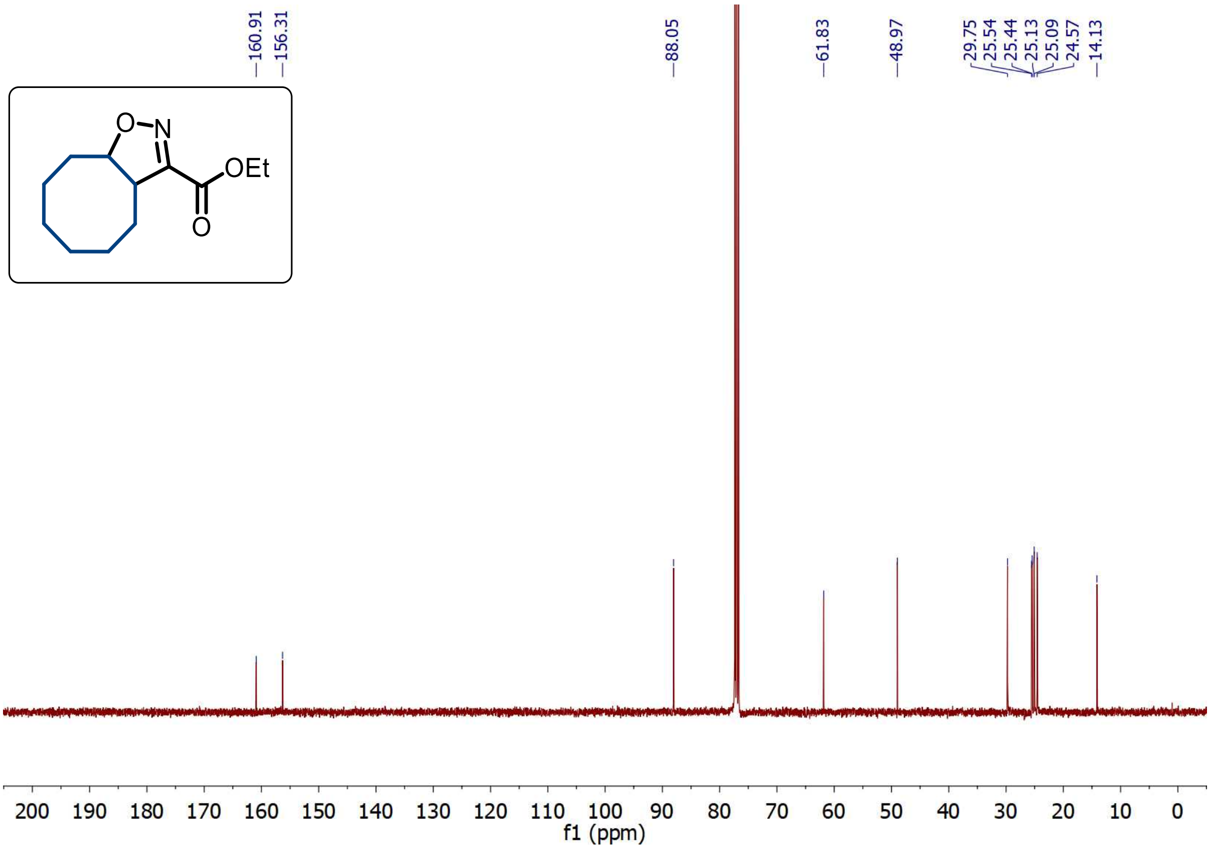

^{1}H-NMR (300 MHz) of Compound 3q

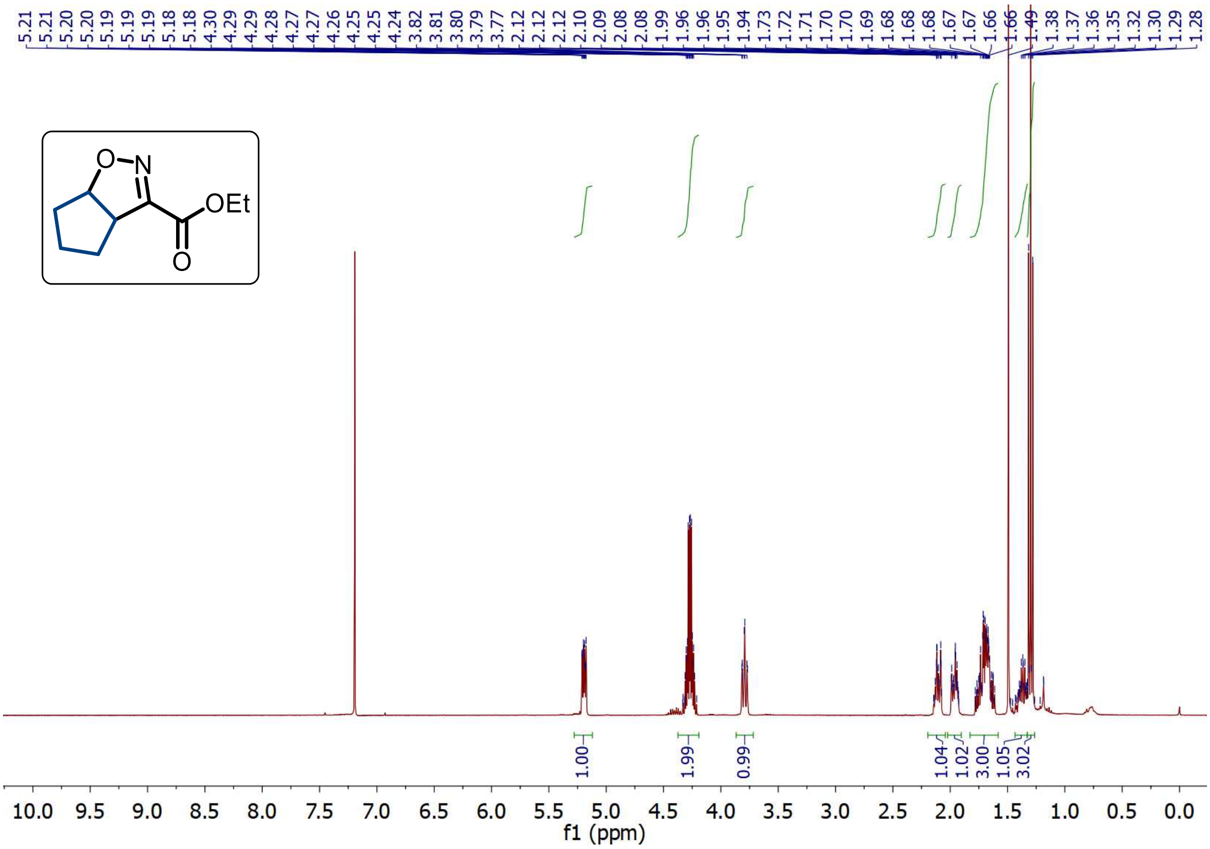

^{13}C-NMR (75 MHz) of Compound 3q

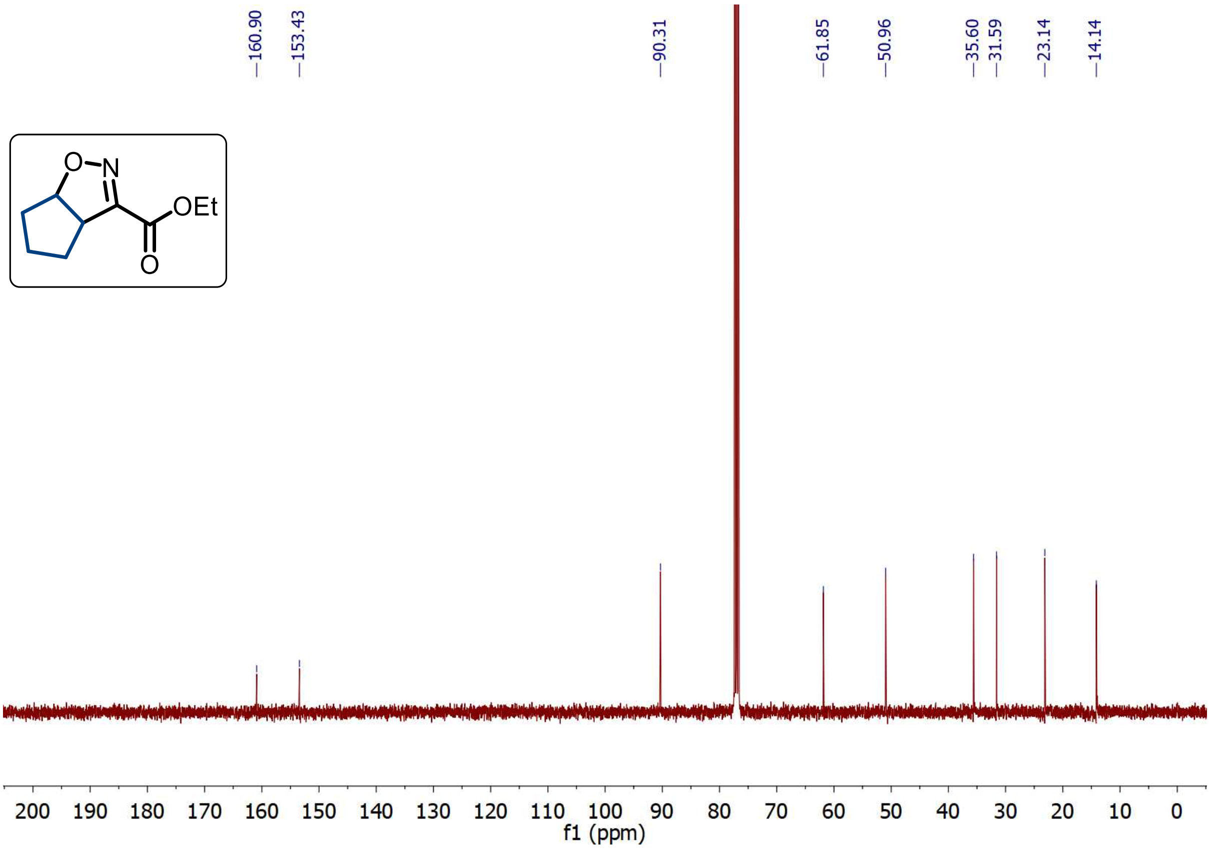

^{1}H-NMR (300 MHz) of Compound 3r

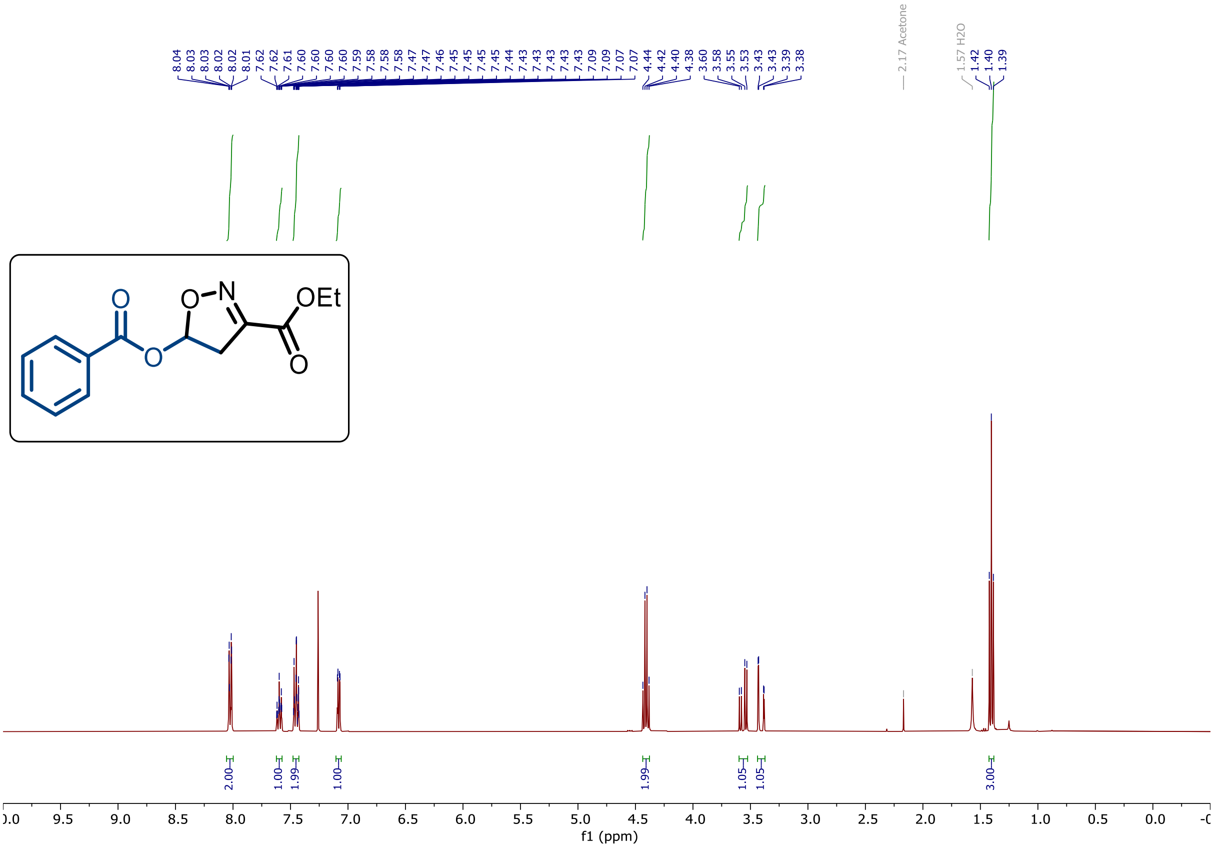

^{13}C-NMR (75 MHz) of Compound 3r

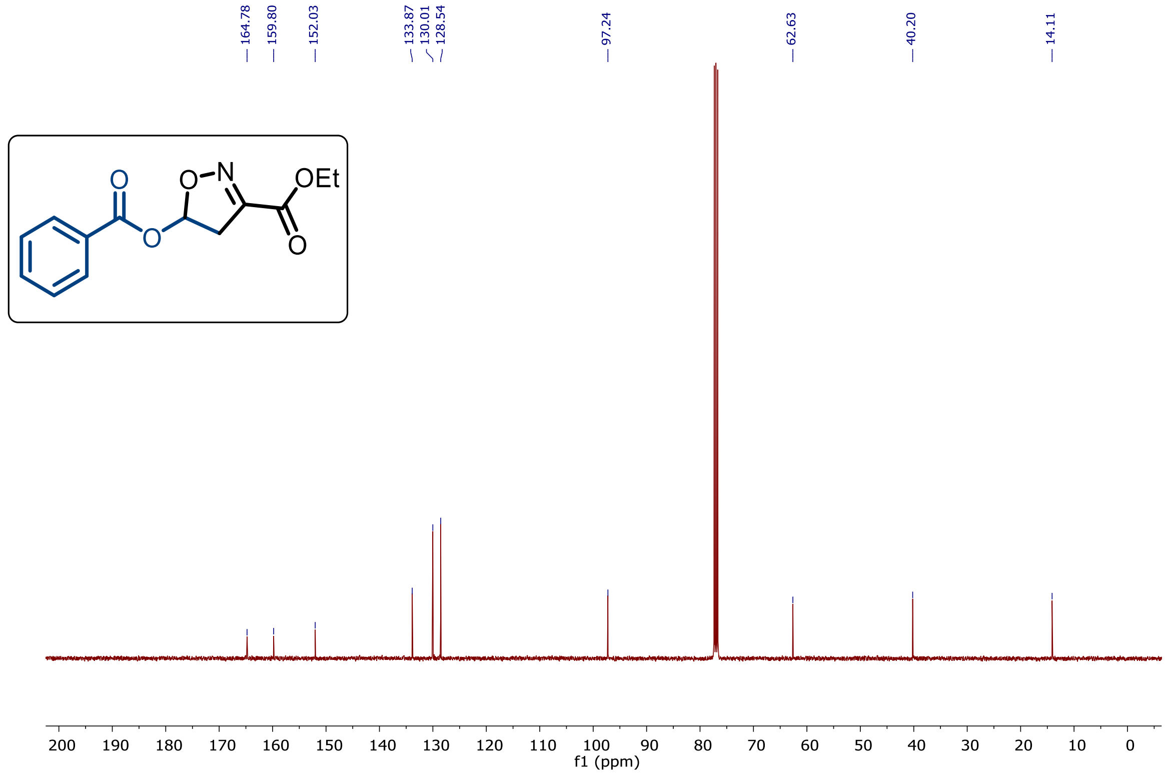

^{1}H-NMR (300 MHz) of Compound 3s

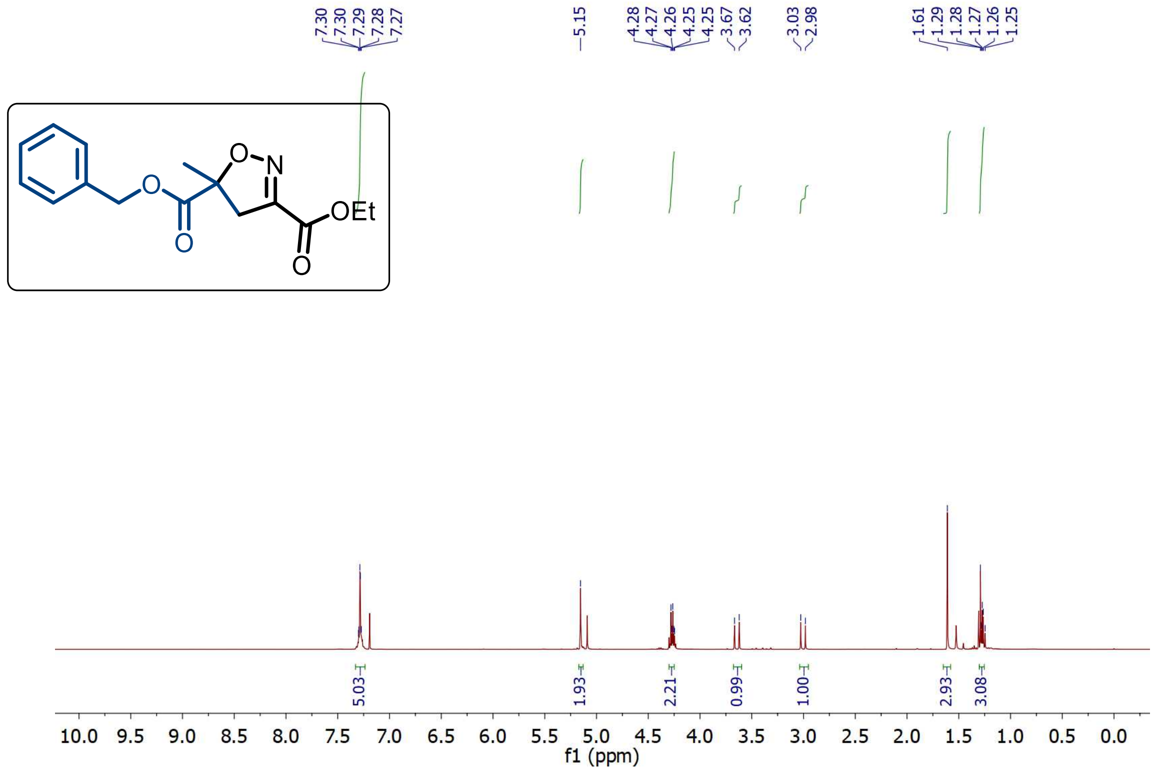

^{13}C-NMR (75 MHz) of Compound 3s

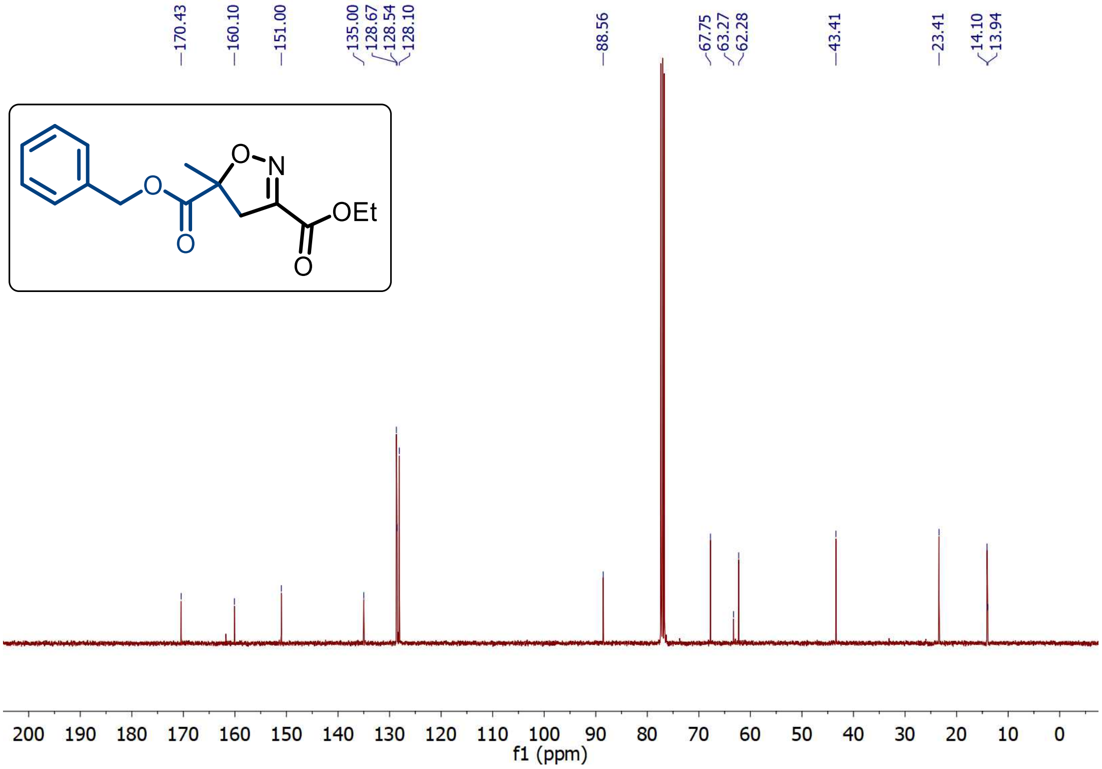

^{1}H-NMR (300 MHz) of Compound 3t

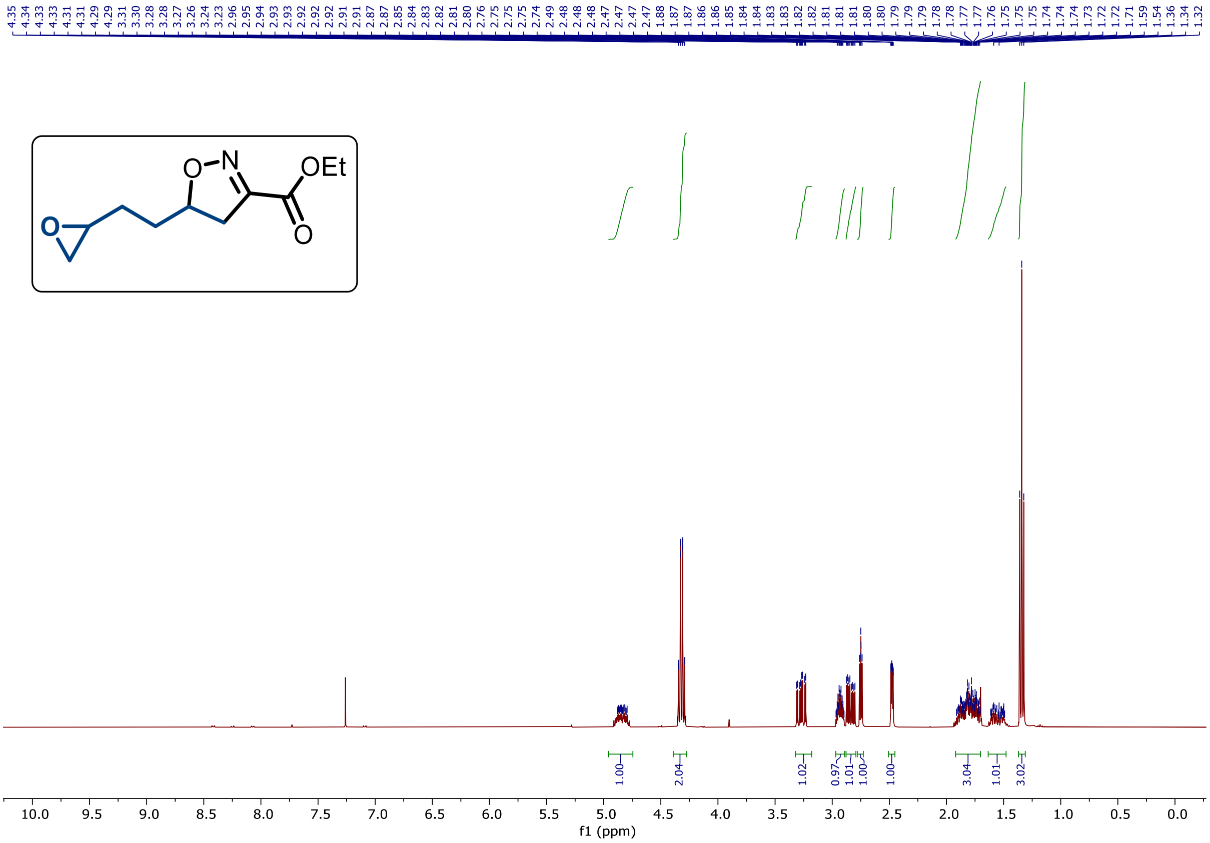

^{13}C-NMR (75 MHz) of Compound 3t

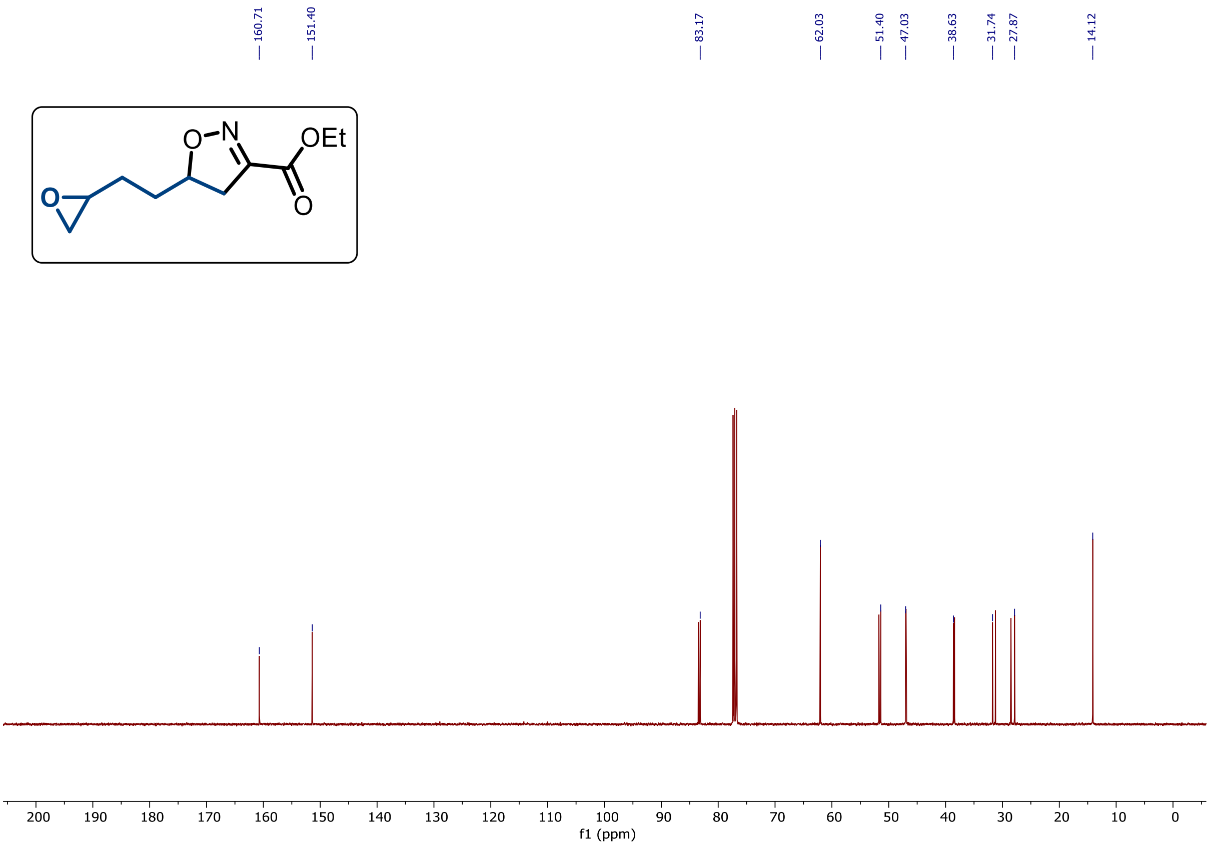

^{1}H-NMR (300 MHz) of Compound 3u

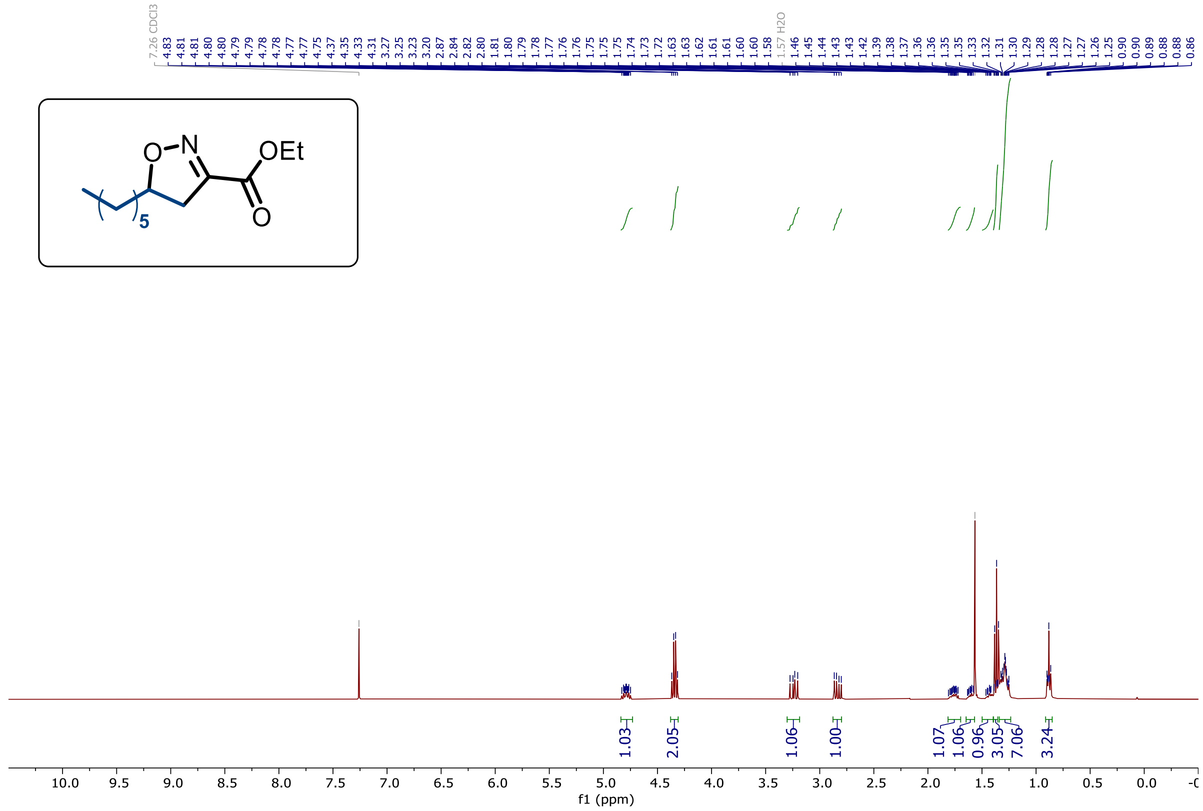

^{13}C-NMR (75 MHz) of Compound 3u

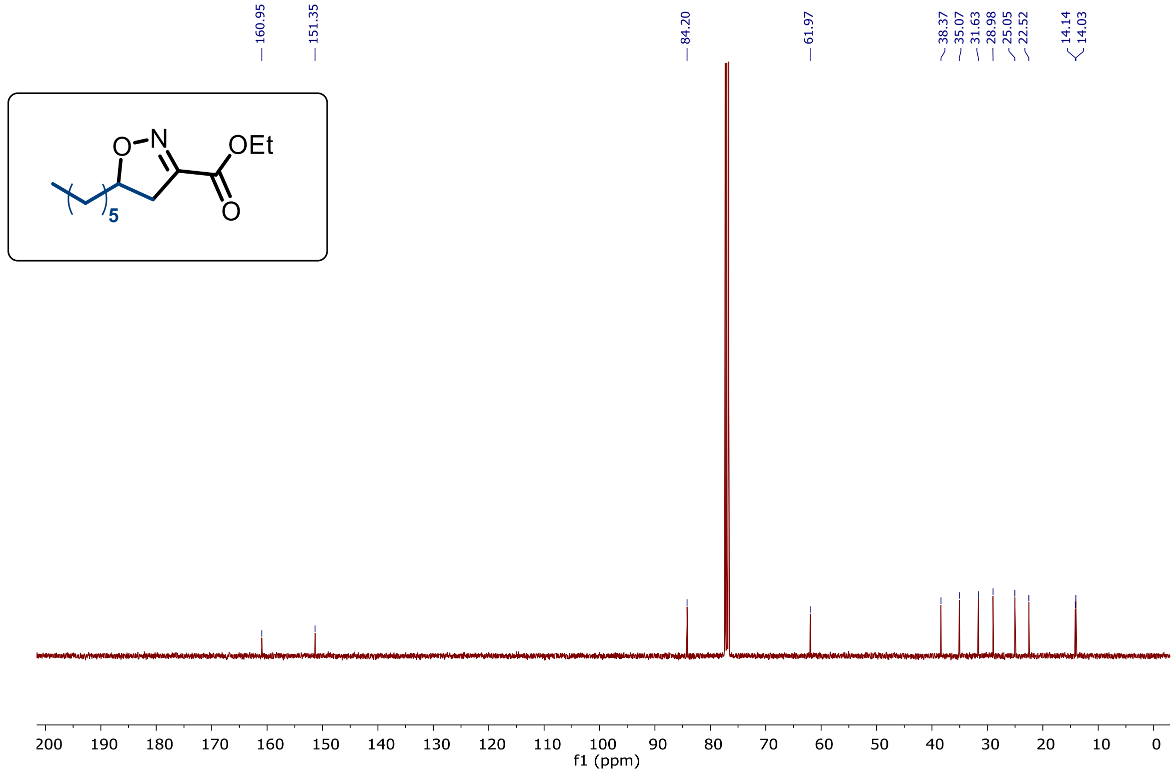

^{1}H-NMR (300 MHz) of Compound 3v

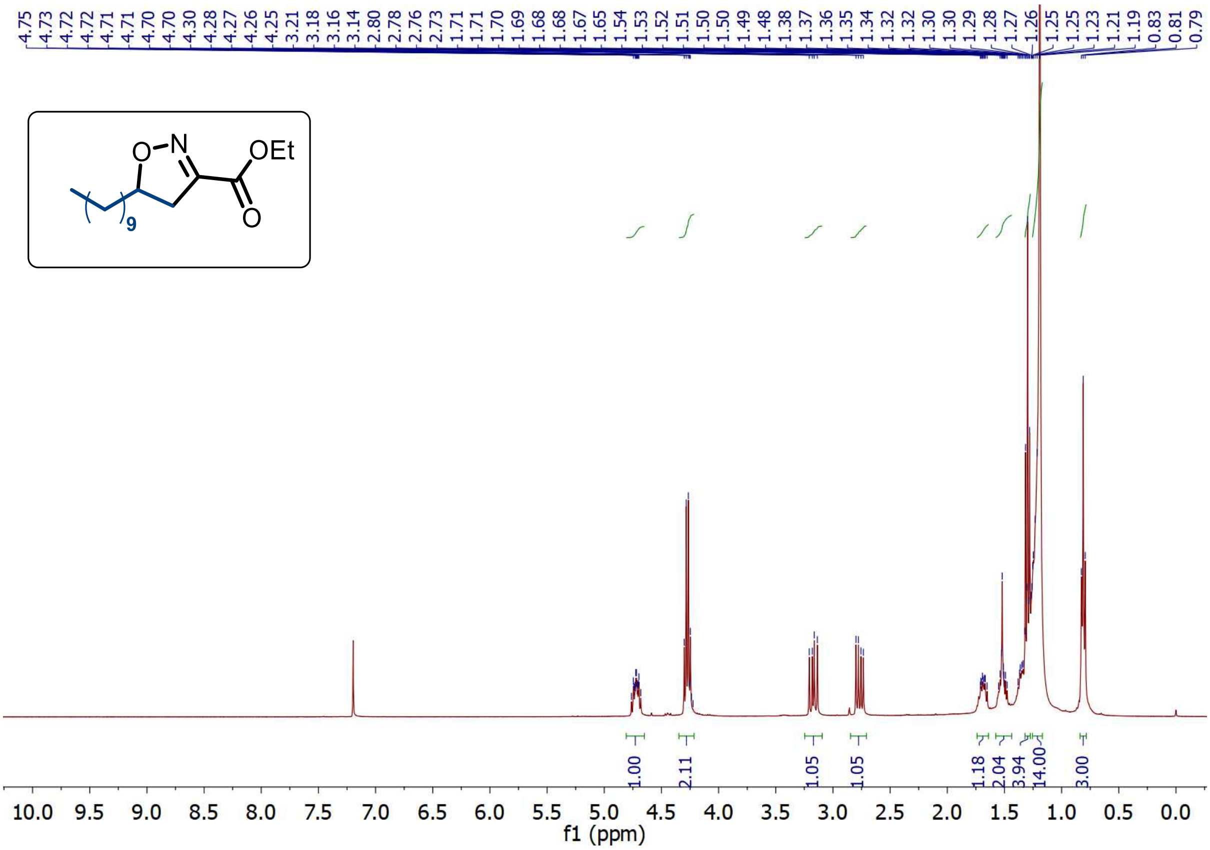

^{13}C-NMR (75 MHz) of Compound 3v

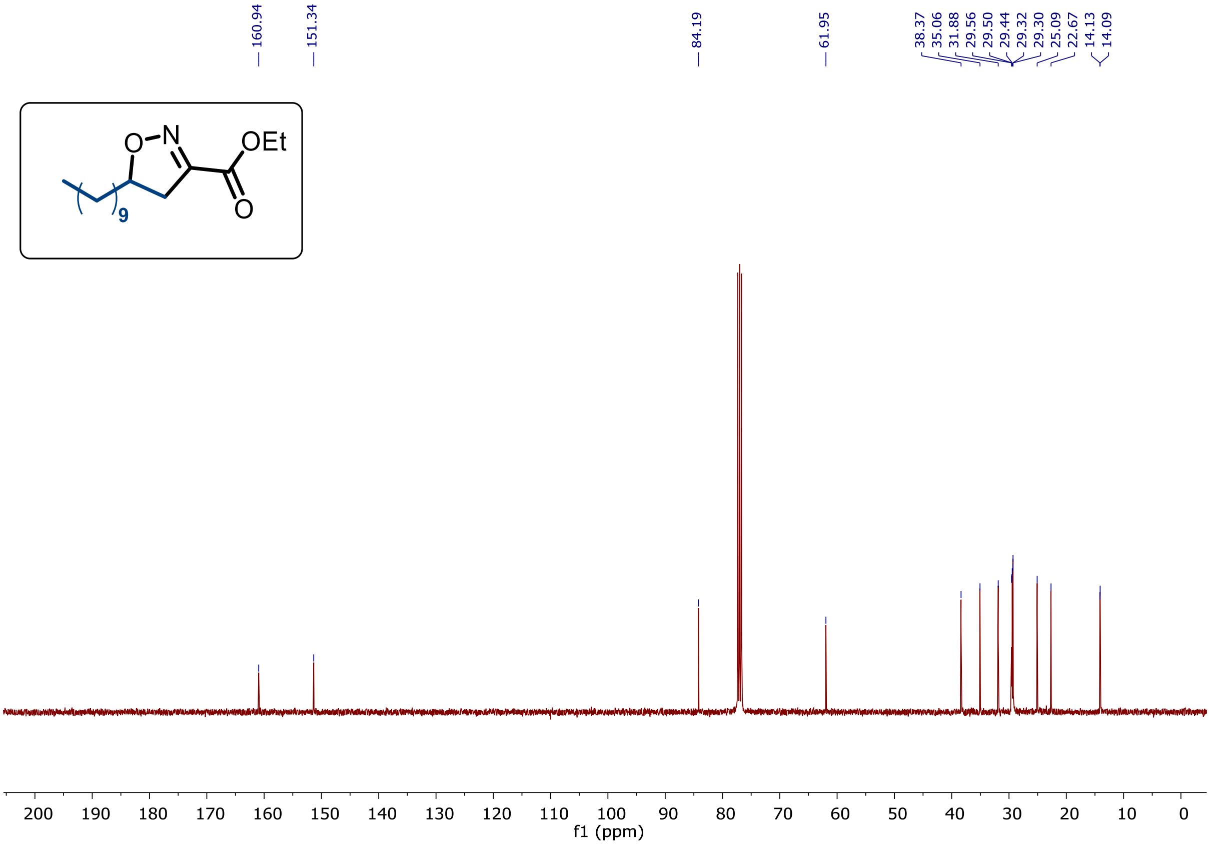

^{1}H-NMR (300 MHz) of Compound 3w

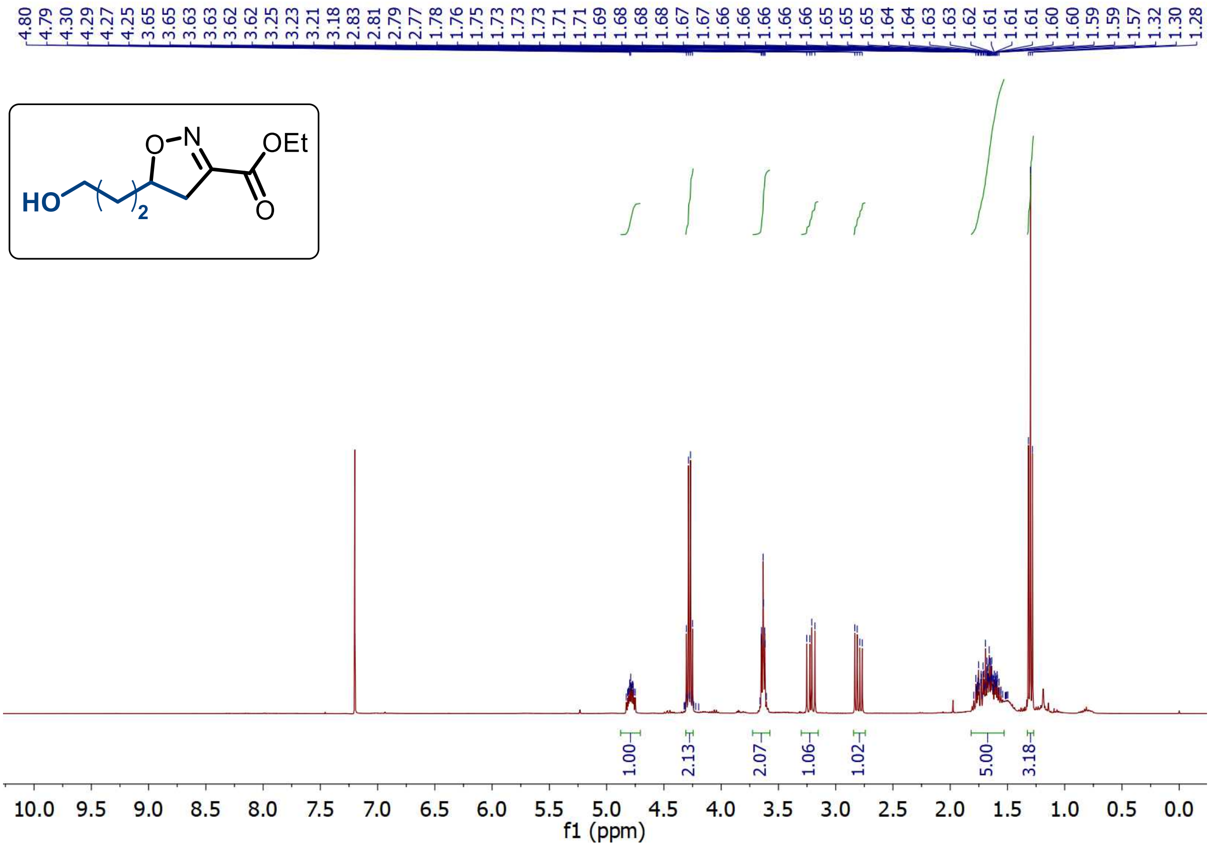

^{13}C-NMR (75 MHz) of Compound 3w

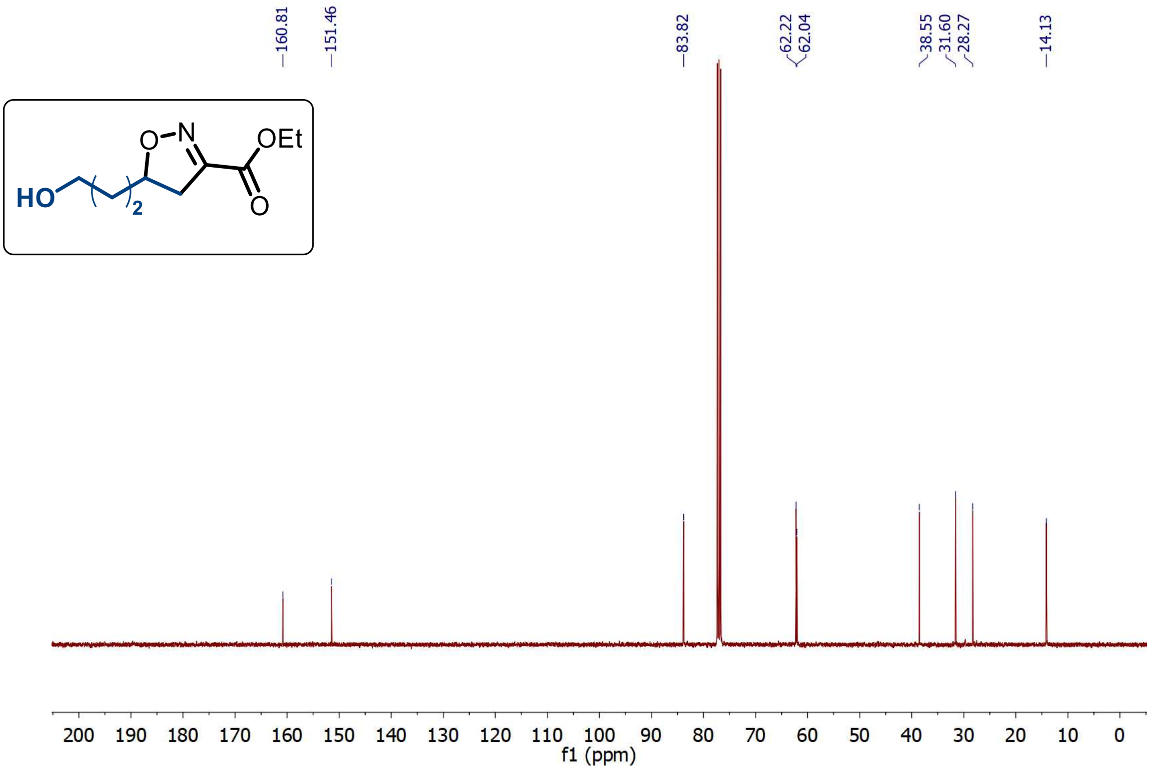

^{1}H-NMR (300 MHz) of Compound 3x

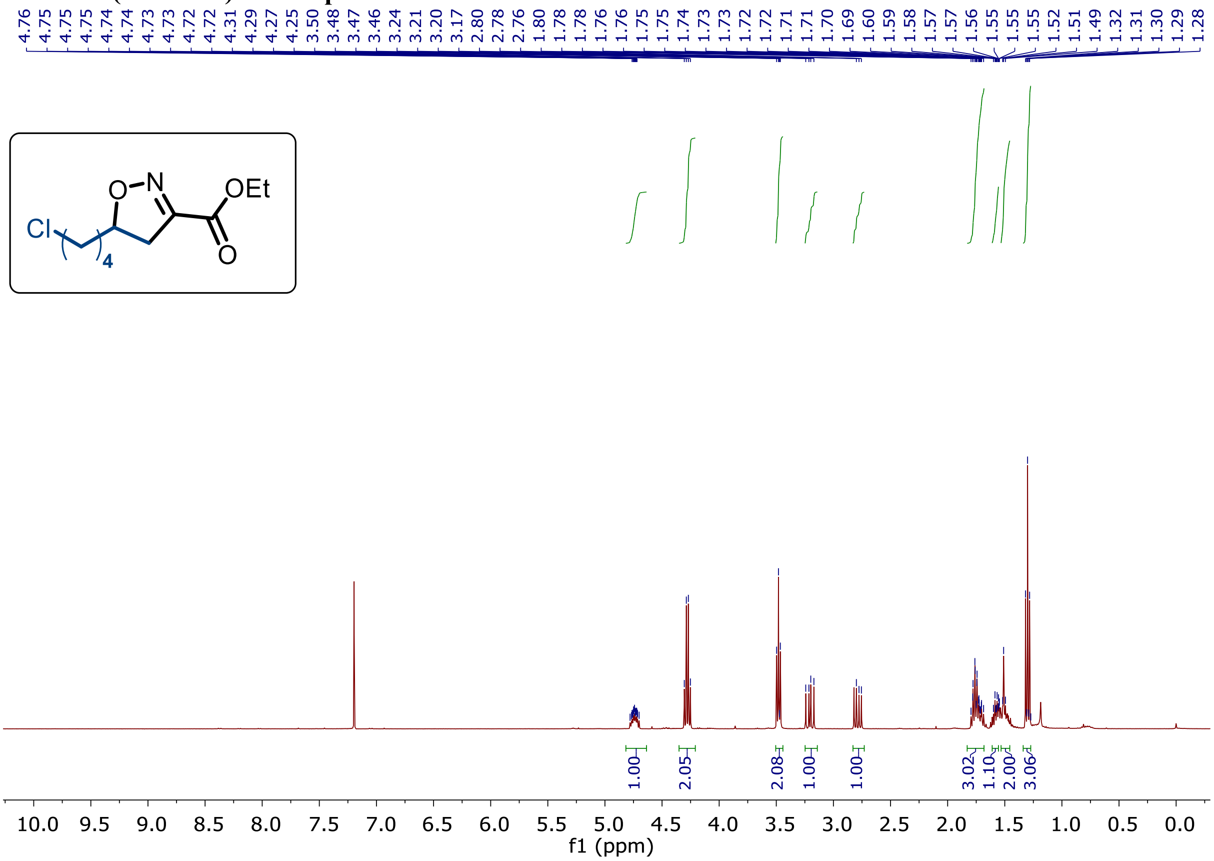

^{13}C-NMR (75 MHz) of Compound 3x

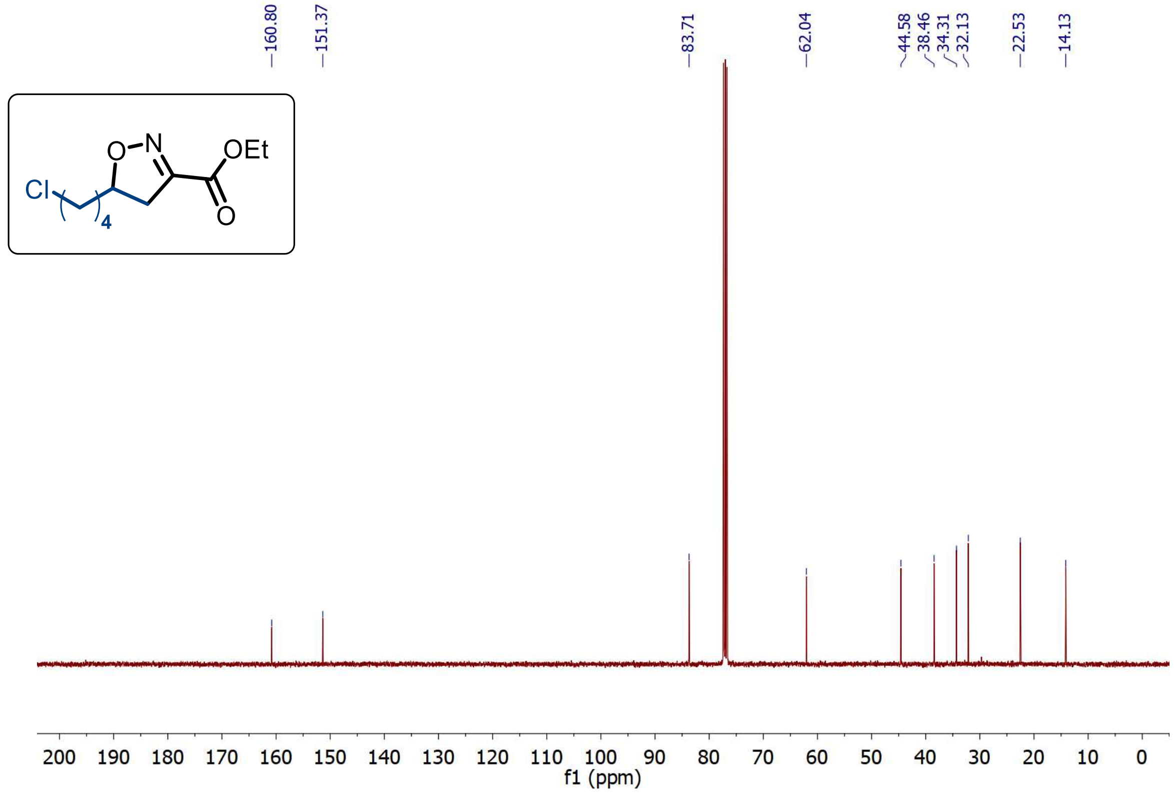

^{1}H-NMR (300 MHz) of Compound 3y

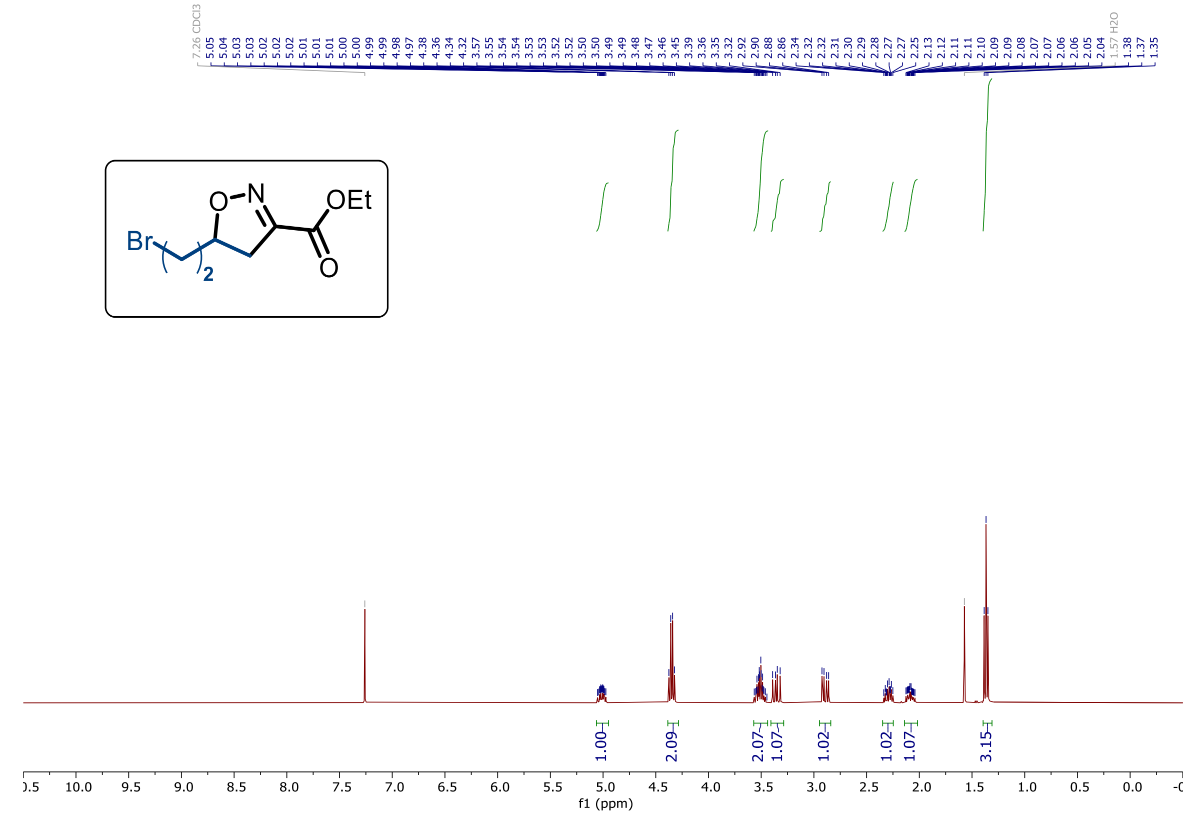

^{13}C-NMR (75 MHz) of Compound 3y

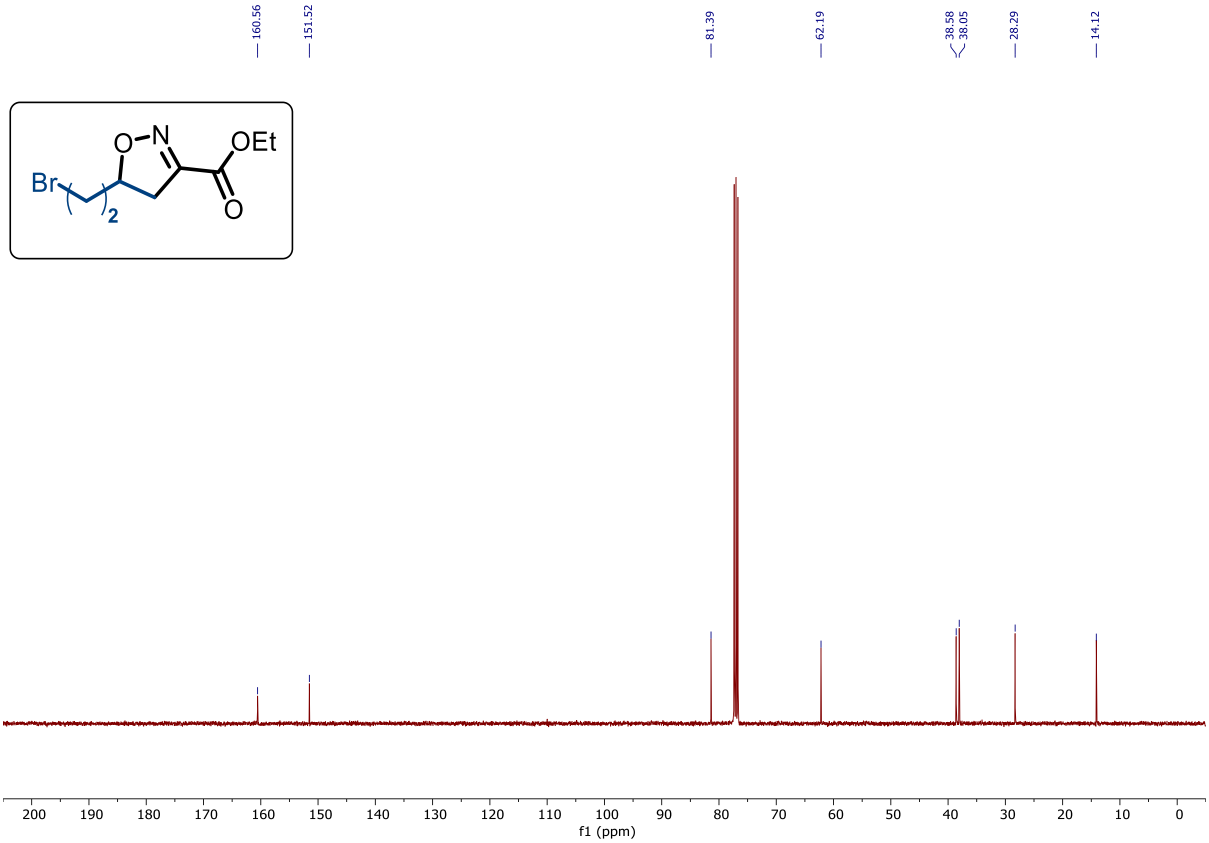

^{1}H-NMR (300 MHz) of Compound 3z

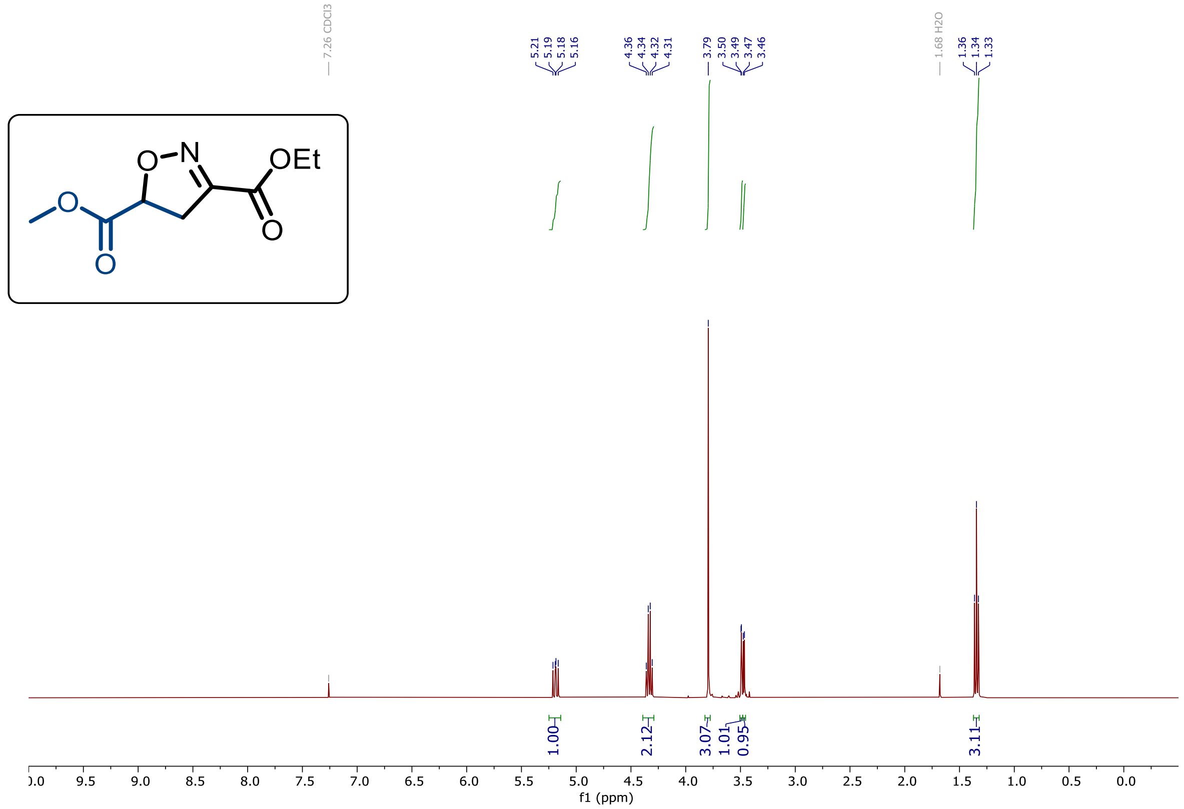

^{13}C-NMR (75 MHz) of Compound 3z

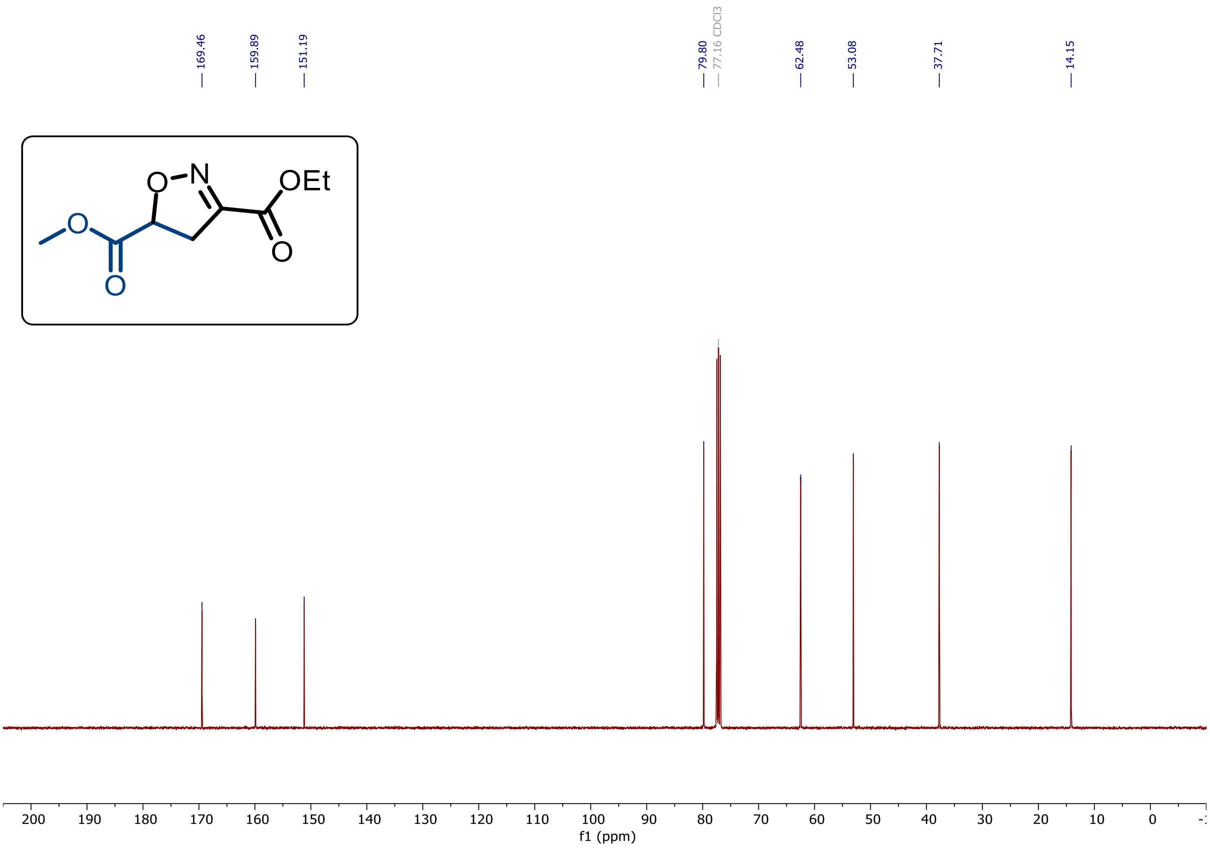

^{1}H-NMR (300 MHz) of Compound 3aa

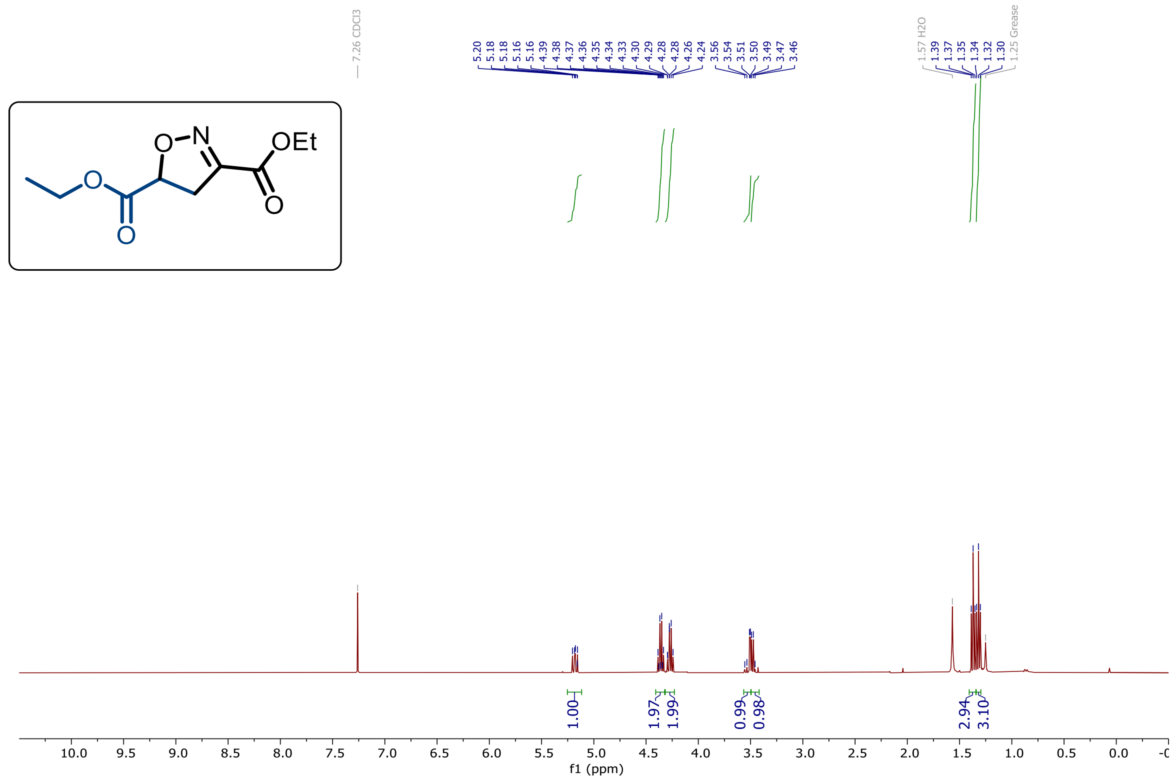

^{13}C-NMR (75 MHz) of Compound 3aa

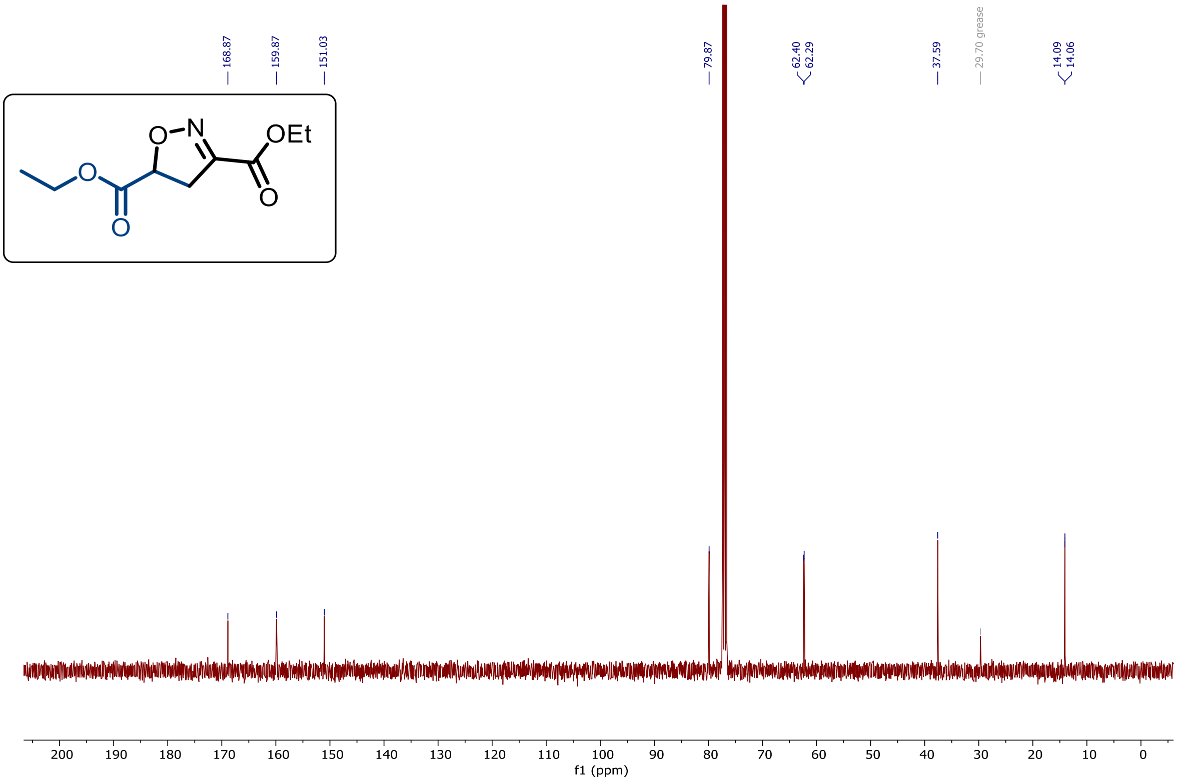

^{1}H-NMR (300 MHz) of Compound 3ab

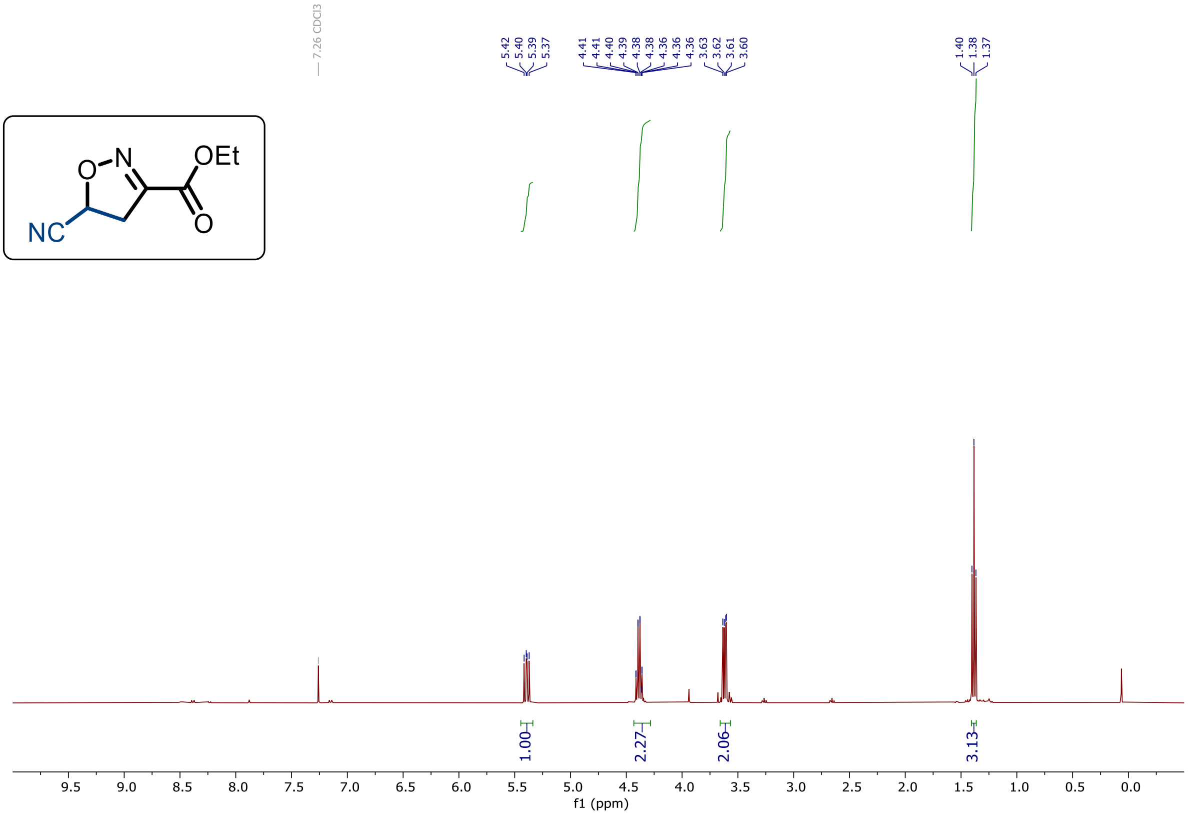

^{13}C-NMR (75 MHz) of Compound 3ab

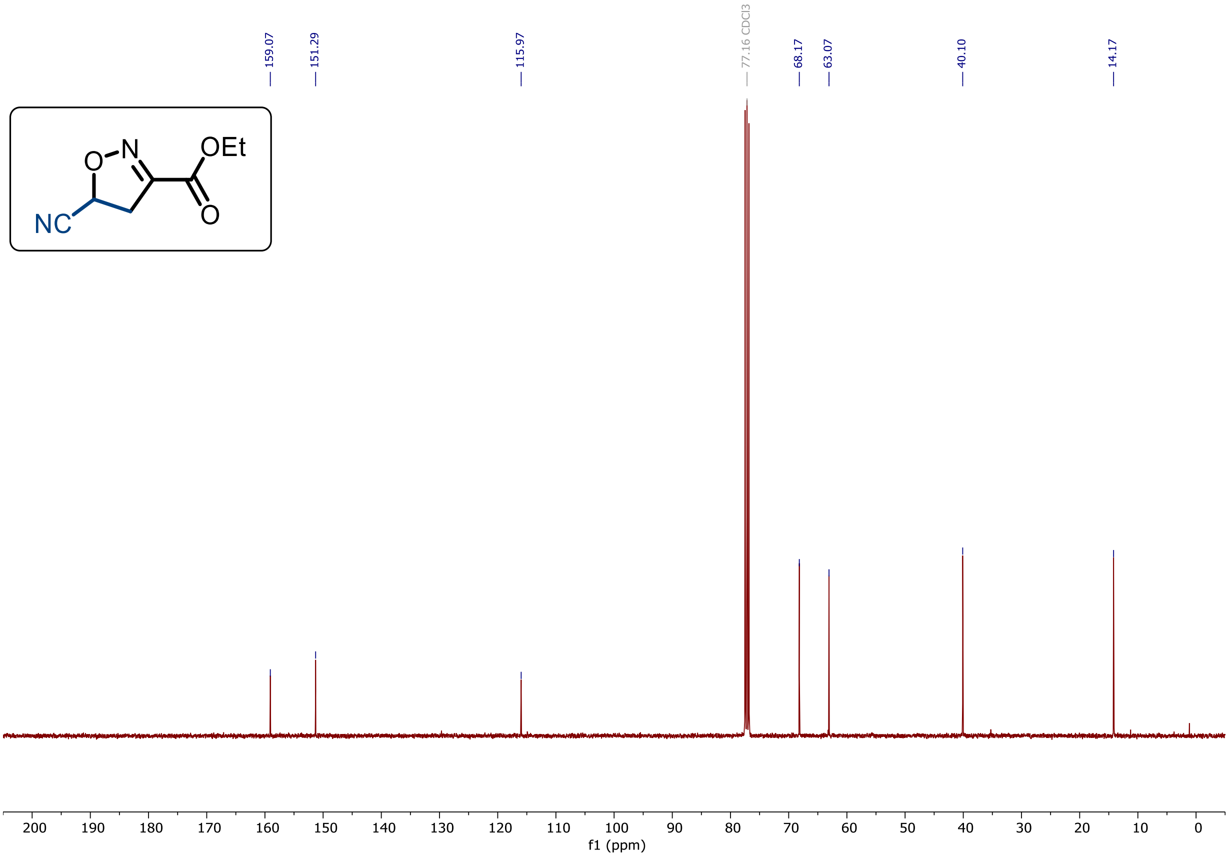

^{1}H-NMR (300 MHz) of Compound 3ac

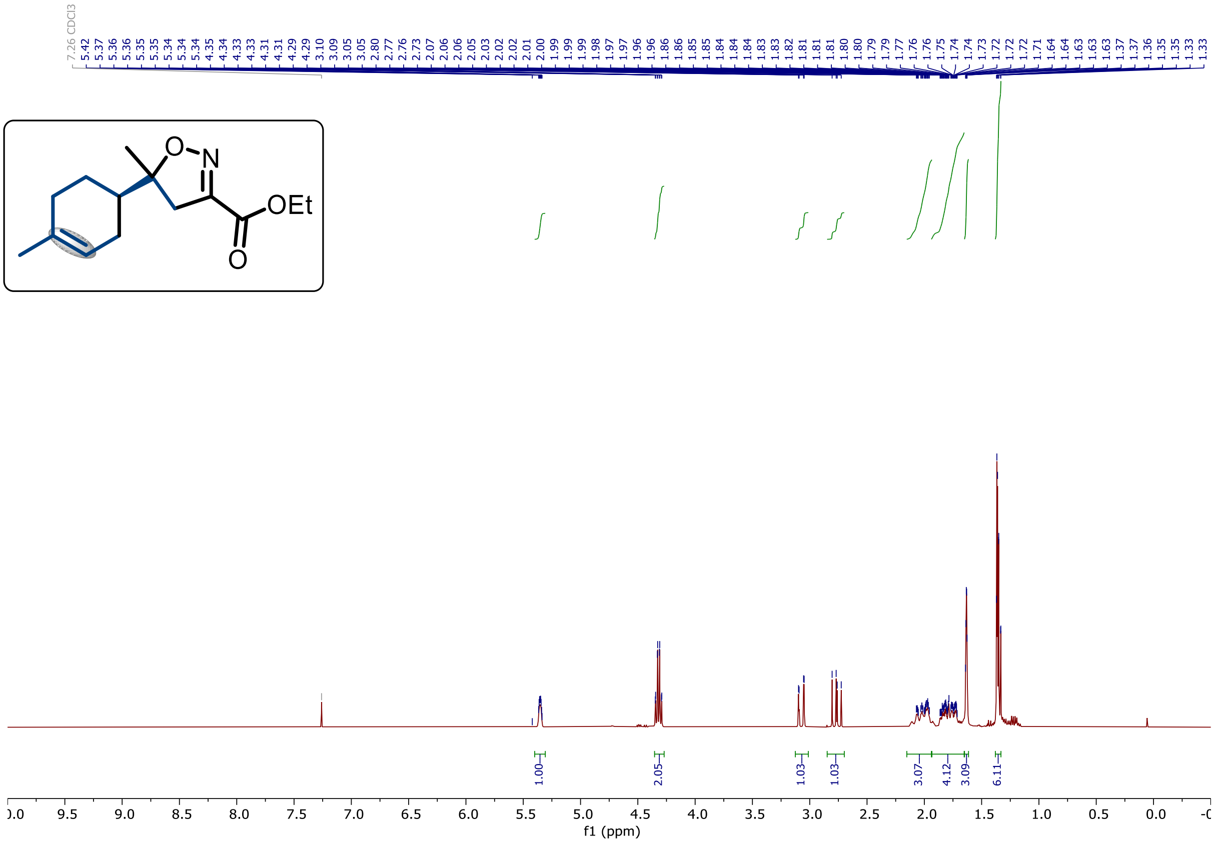

^{13}C-NMR (75 MHz) of Compound 3ac

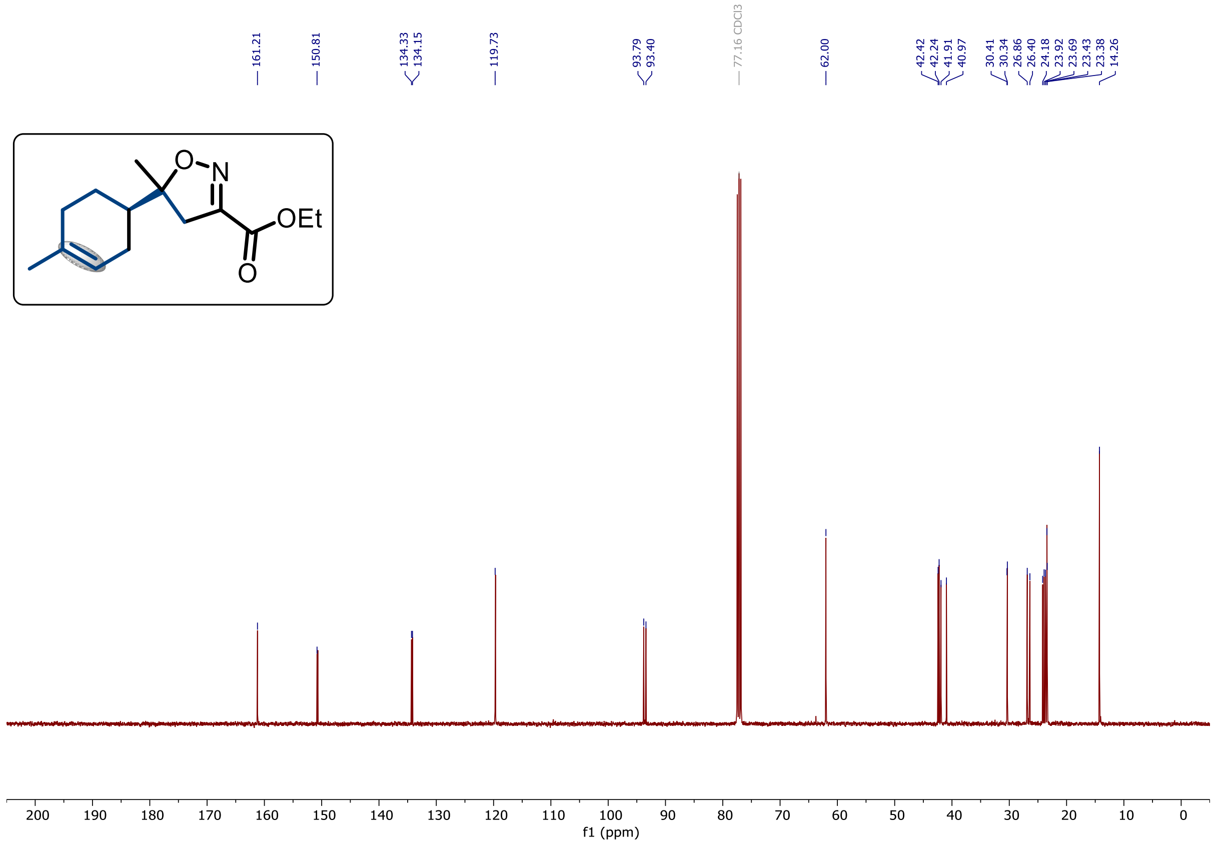

^{1}H-NMR (300 MHz) of Compound 3ad

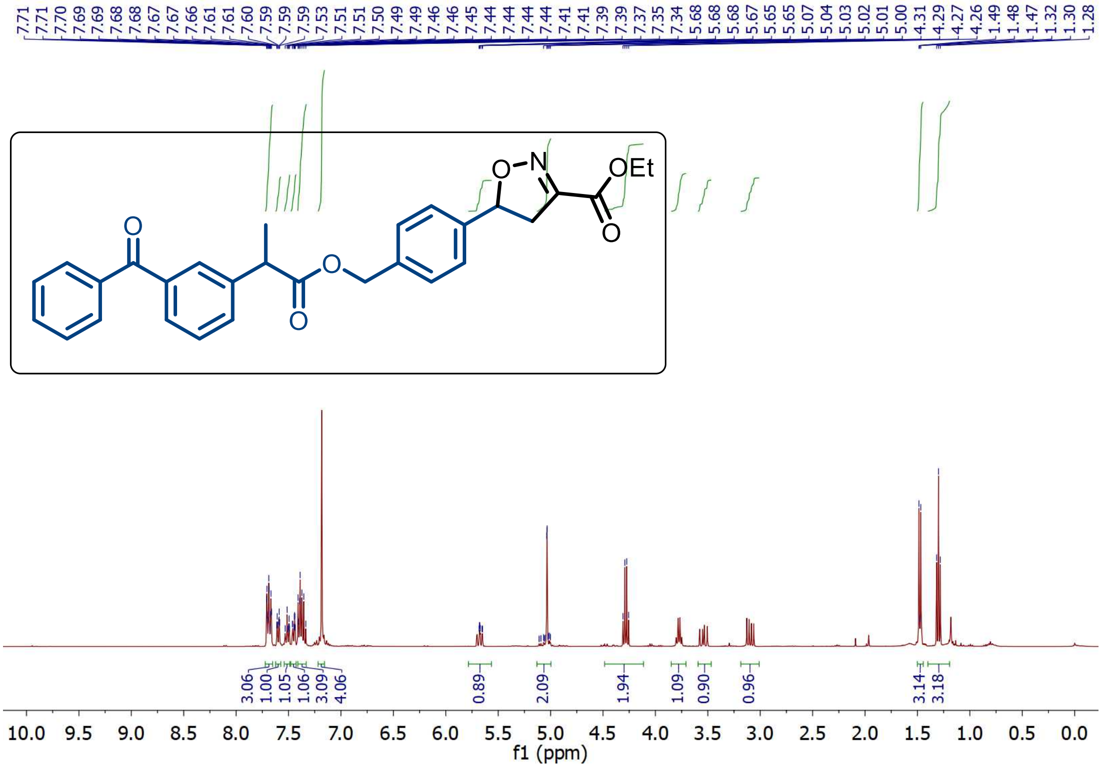

^{13}C-NMR (75 MHz) of Compound 3ad

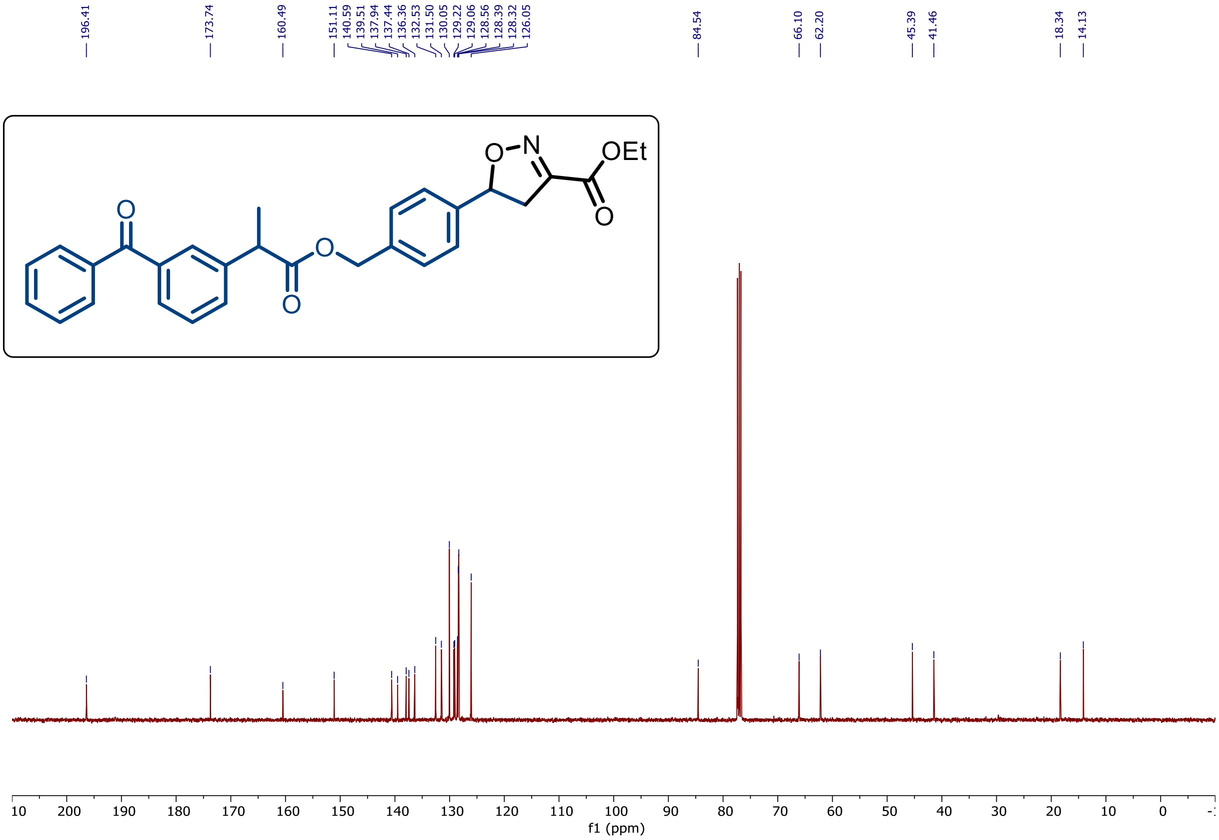

^{1}H-NMR (300 MHz) of Compound 3ae

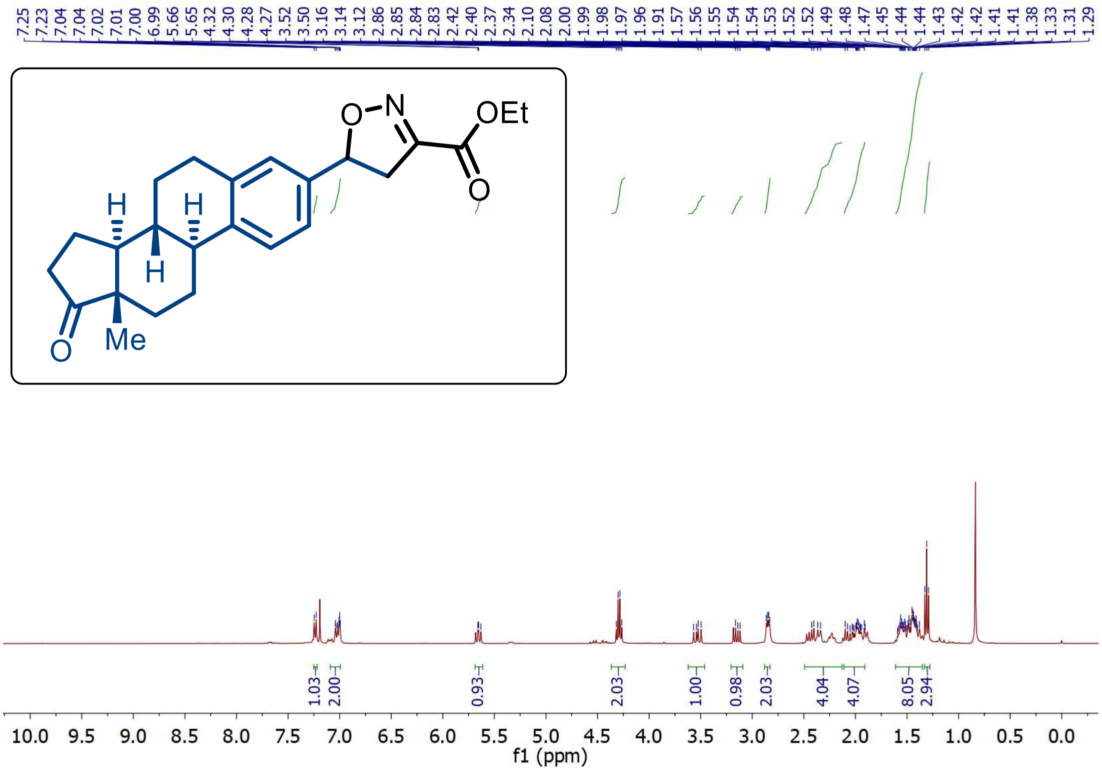

^{13}C-NMR (75 MHz) of Compound 3ae

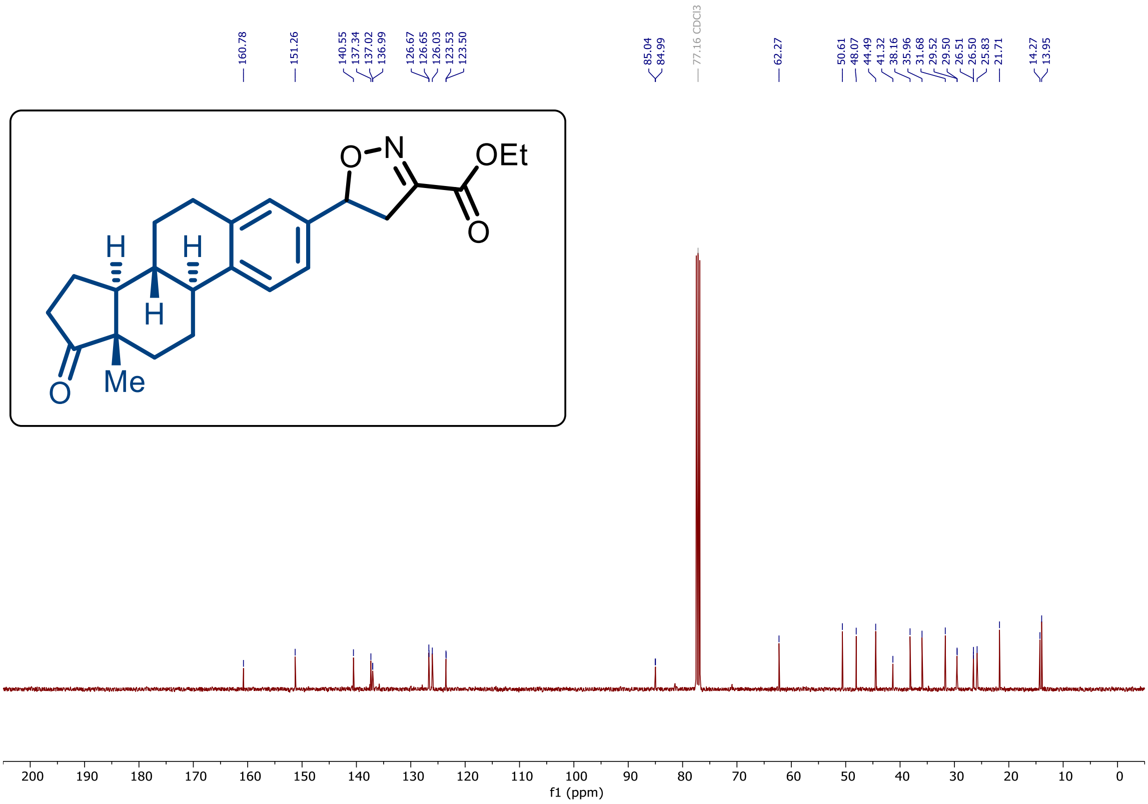

^{1}H-NMR (300 MHz) of Compound 3af

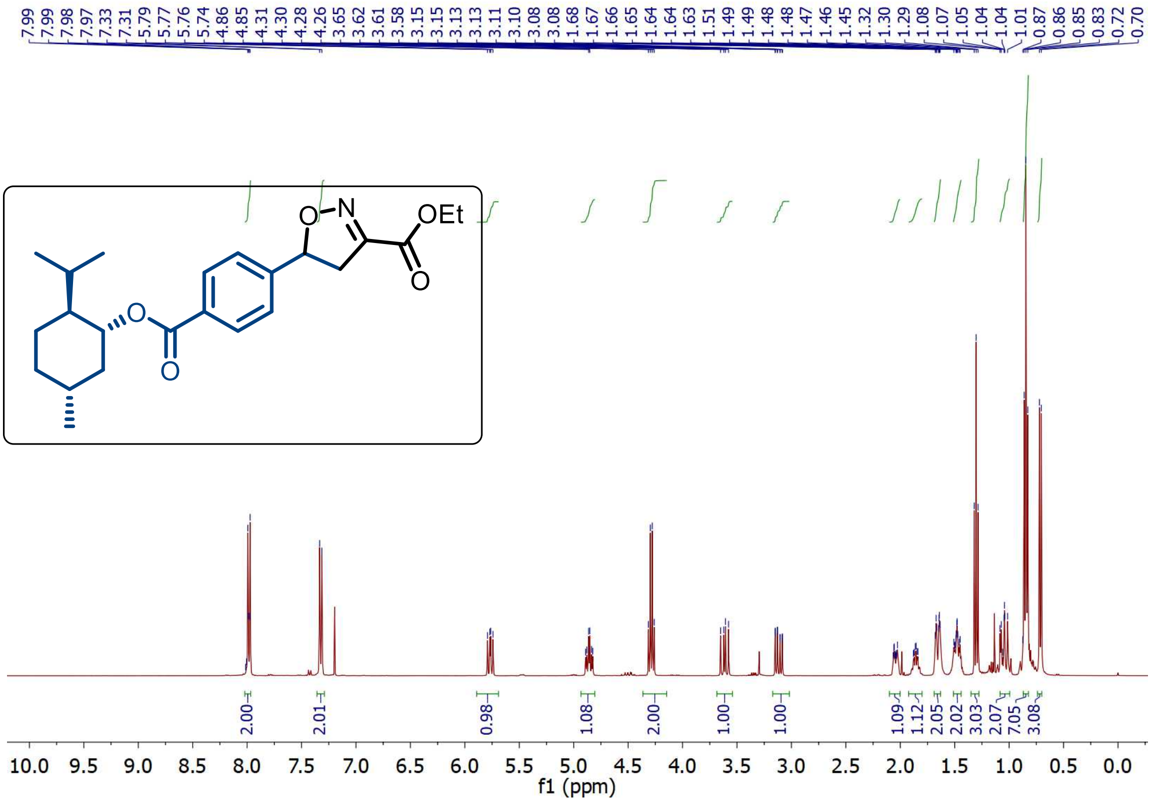

^{13}C-NMR (75 MHz) of Compound 3af

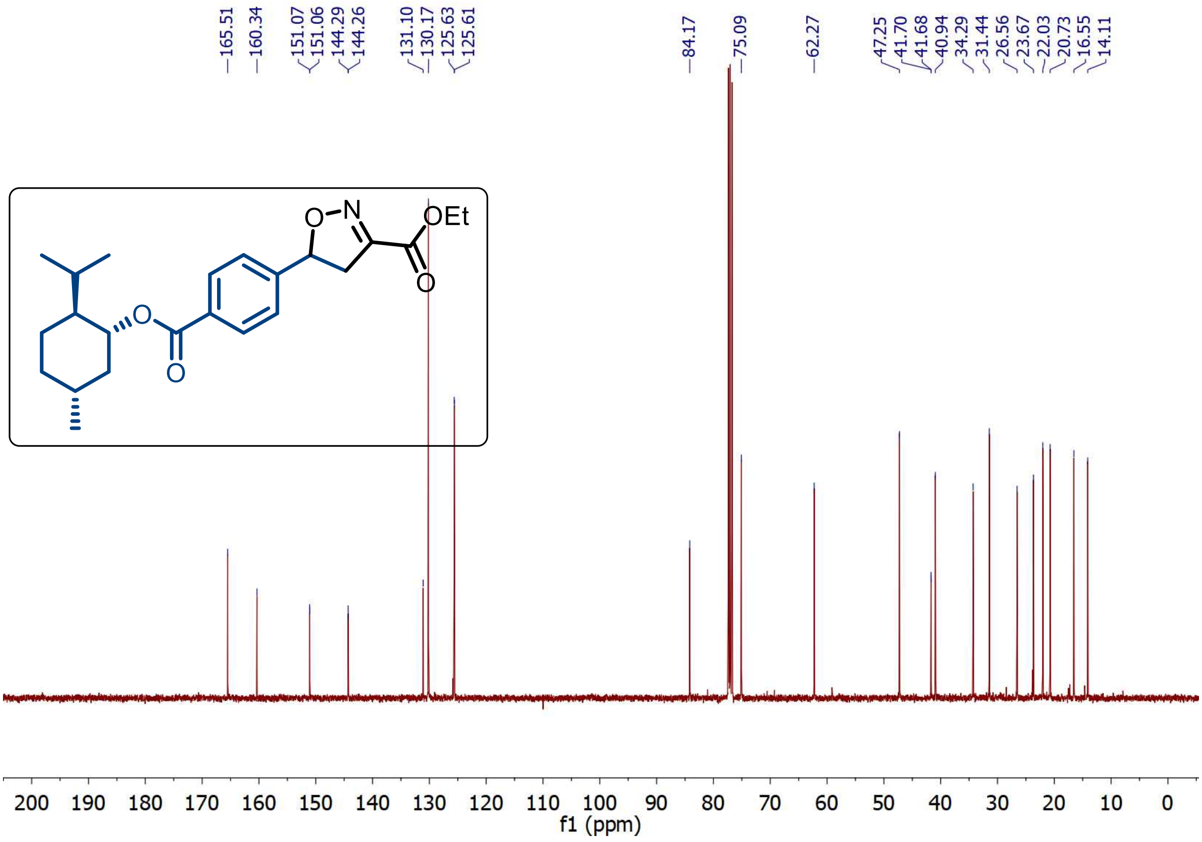

^{1}H-NMR (300 MHz) of Compound 4a

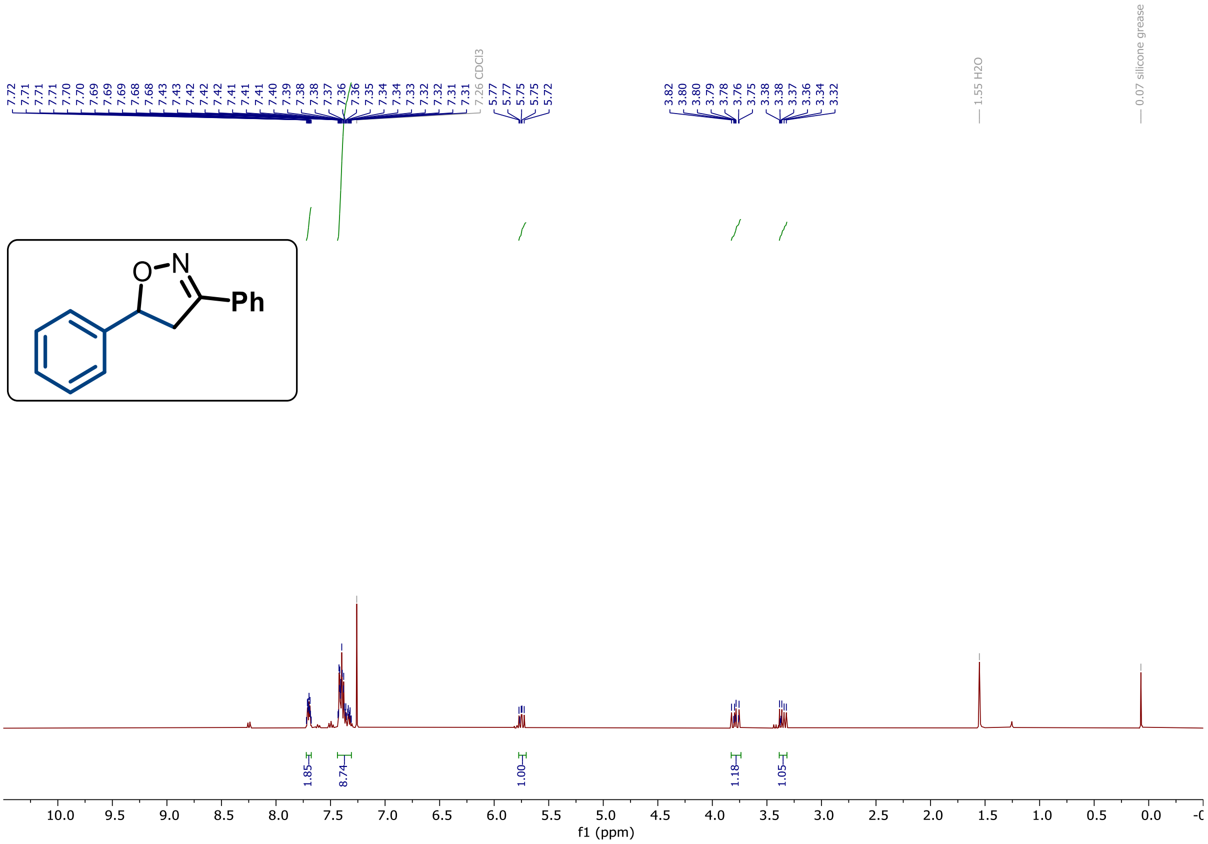

^{13}C-NMR (75 MHz) of Compound 4a

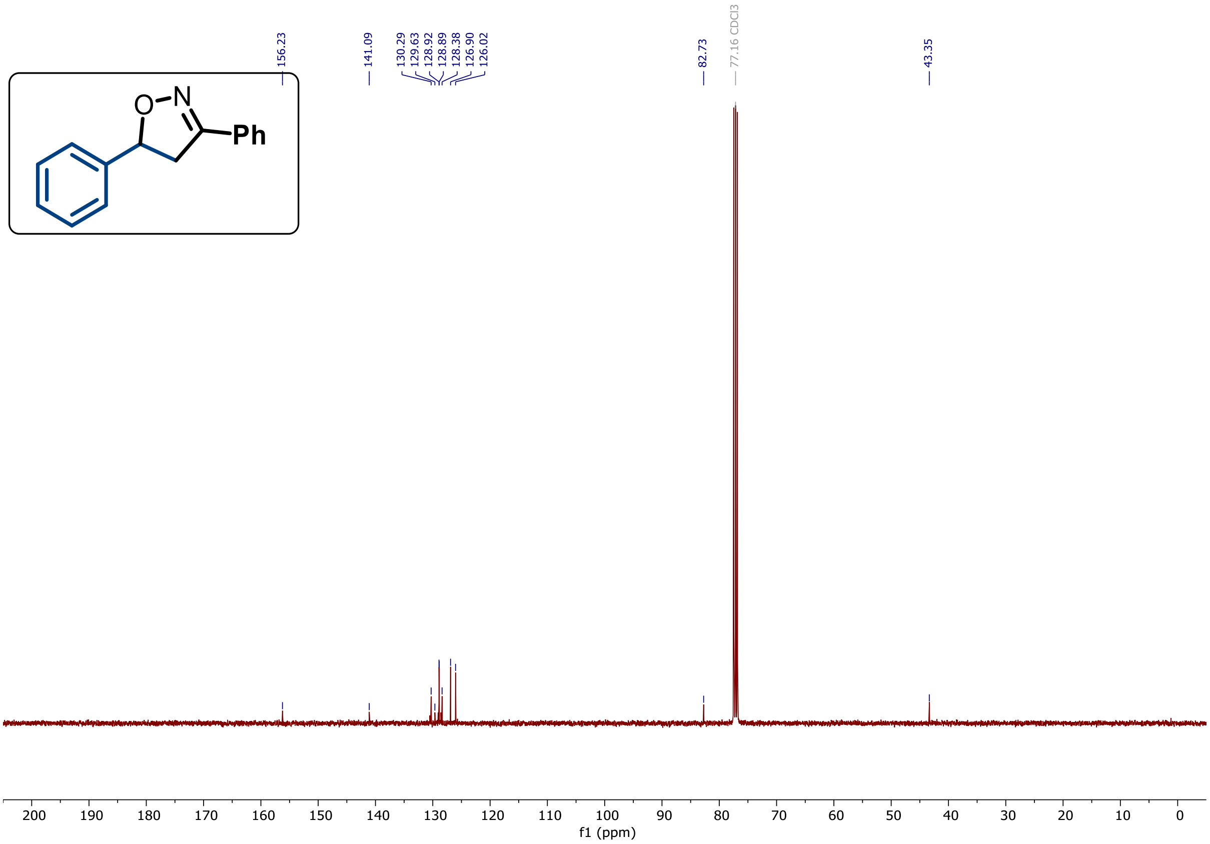

^{1}H-NMR (300 MHz) of Compound 4b

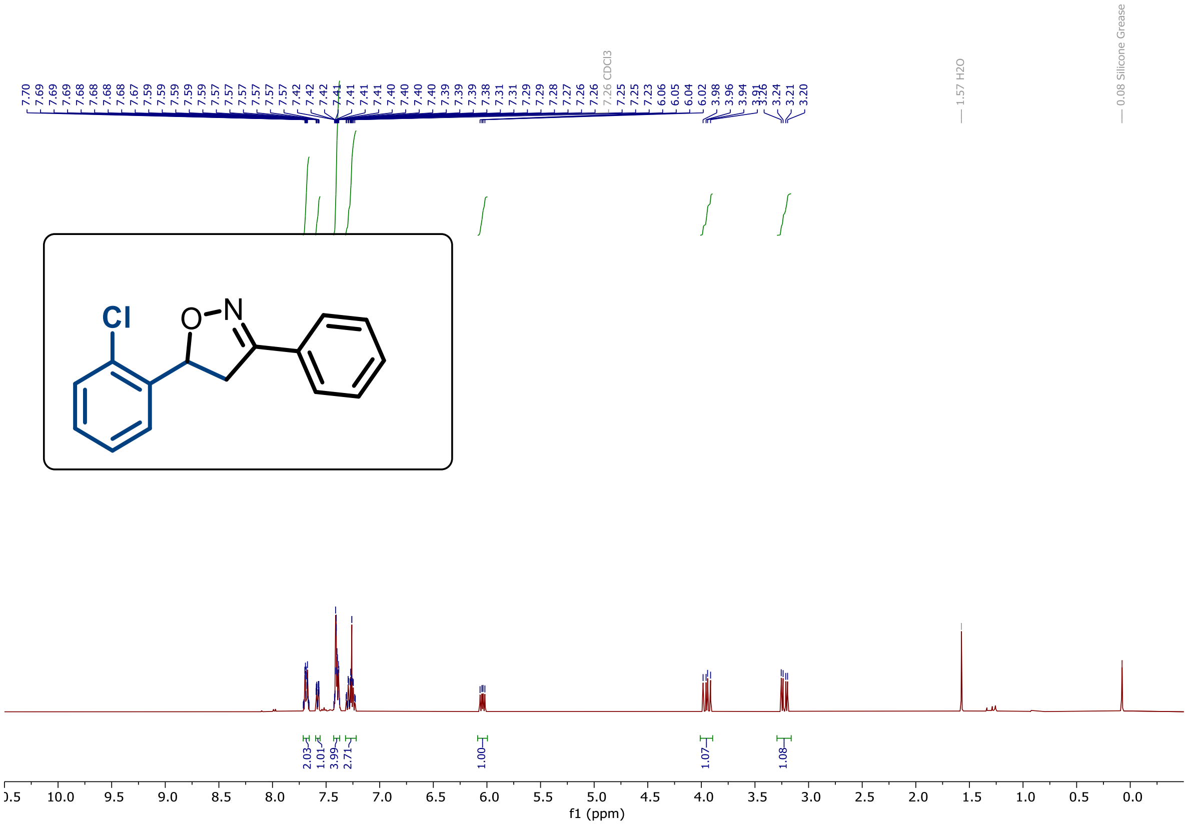

^{13}C-NMR (75 MHz) of Compound 4b

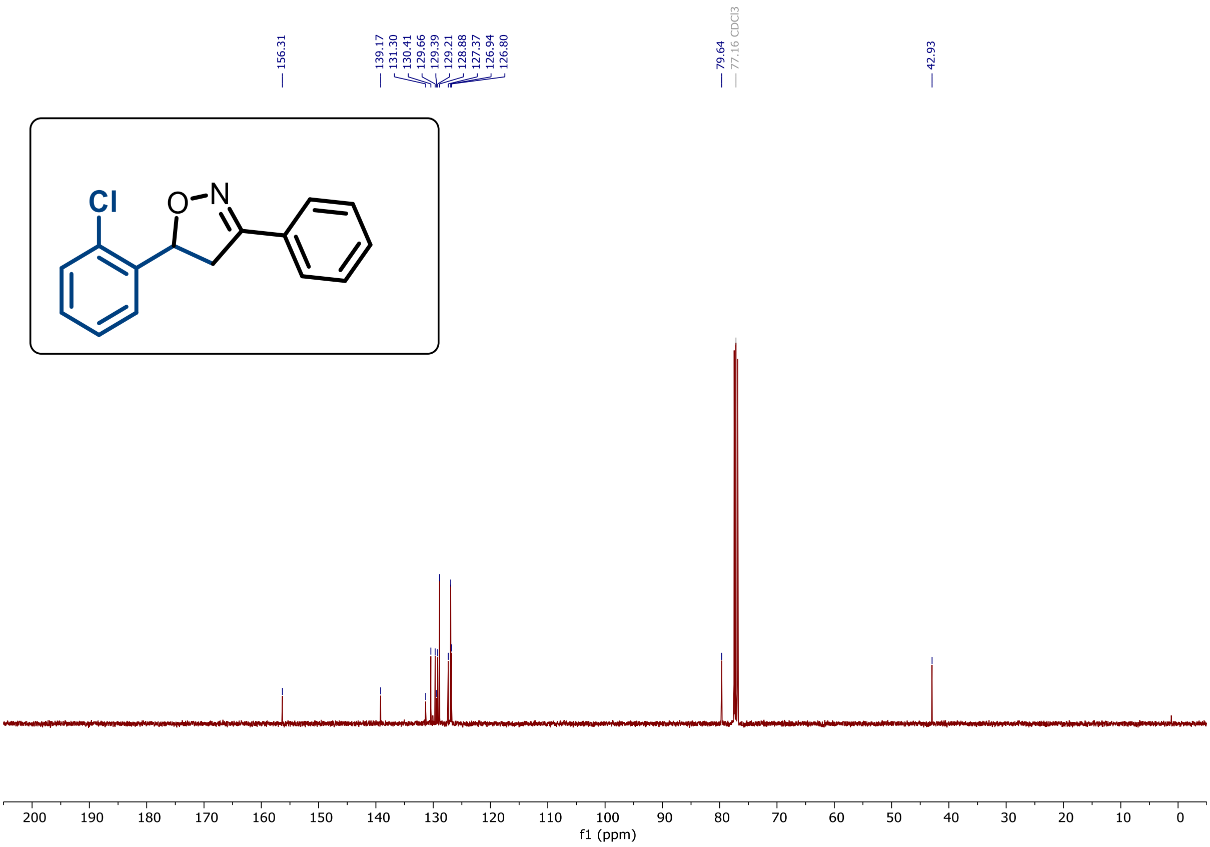

^{1}H-NMR (300 MHz) of Compound 4c

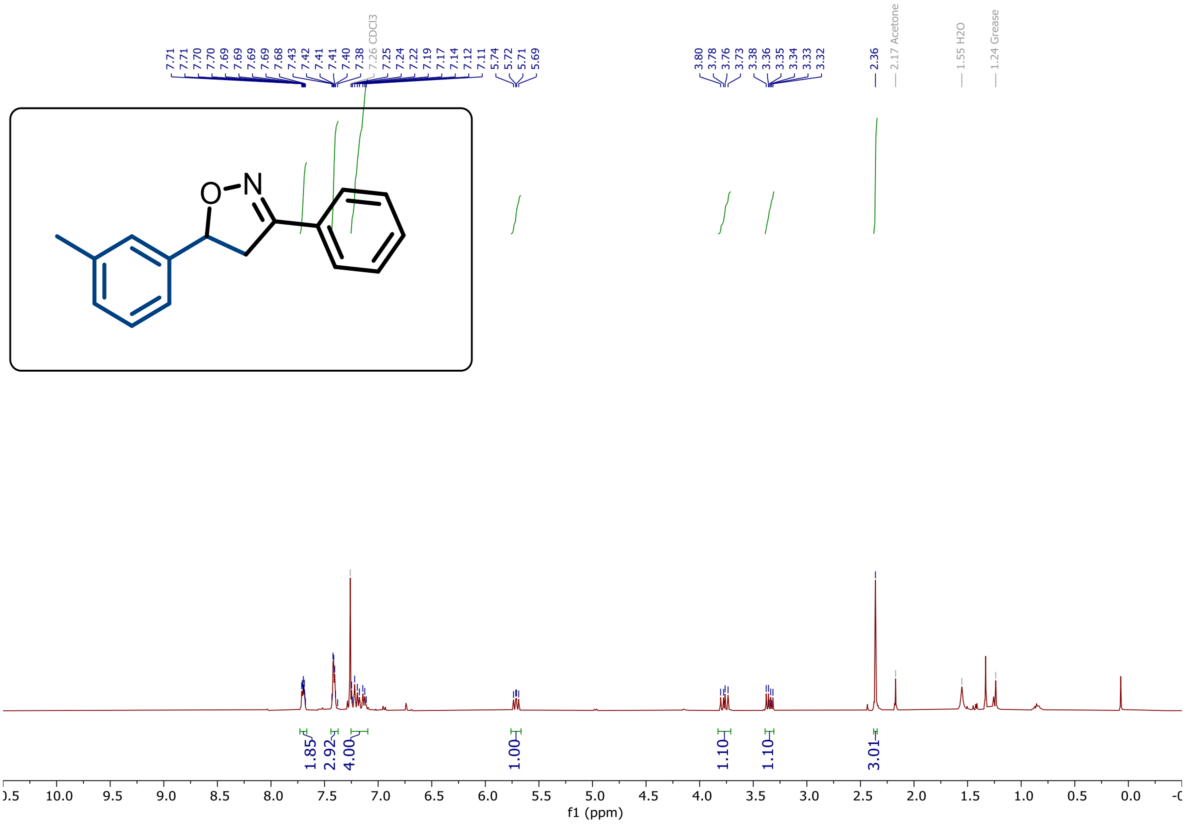

^{13}C-NMR (75 MHz) of Compound 4c

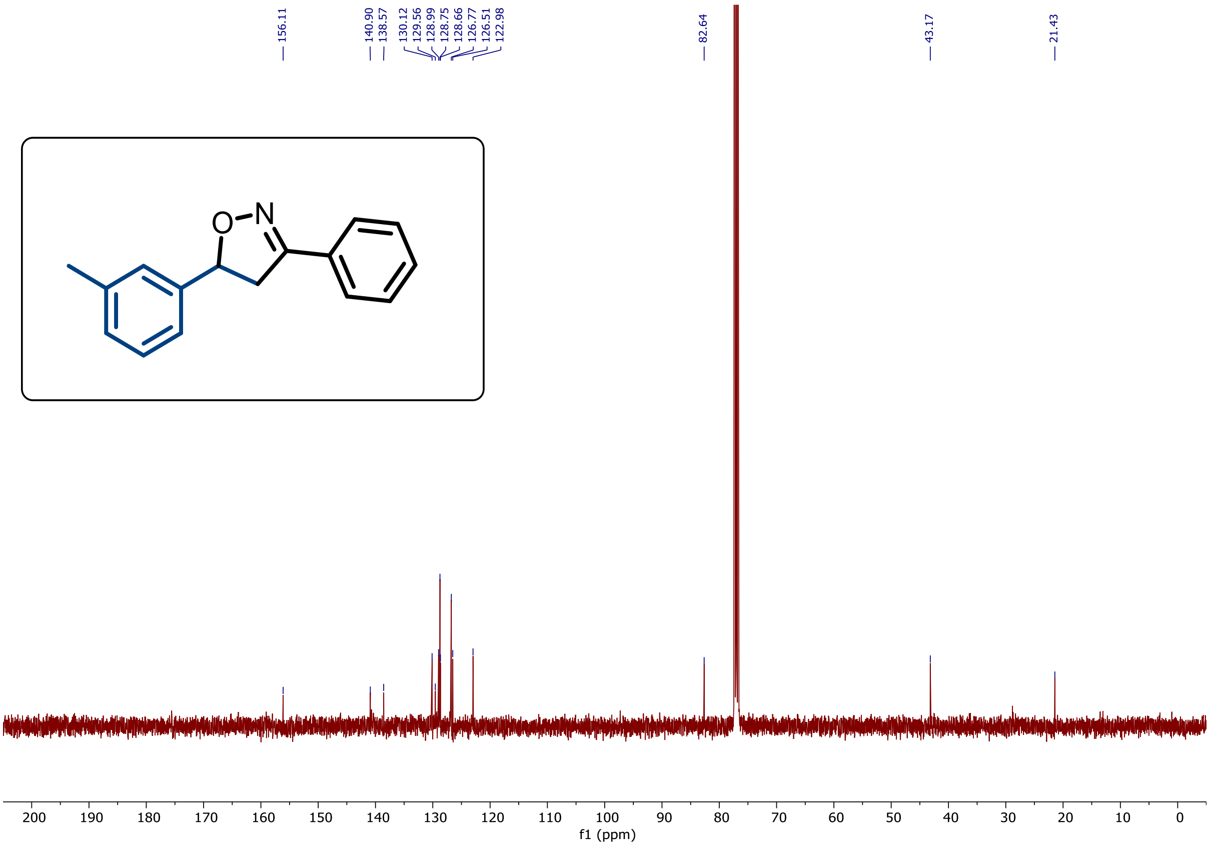

^{1}H-NMR (300 MHz) of Compound 4d

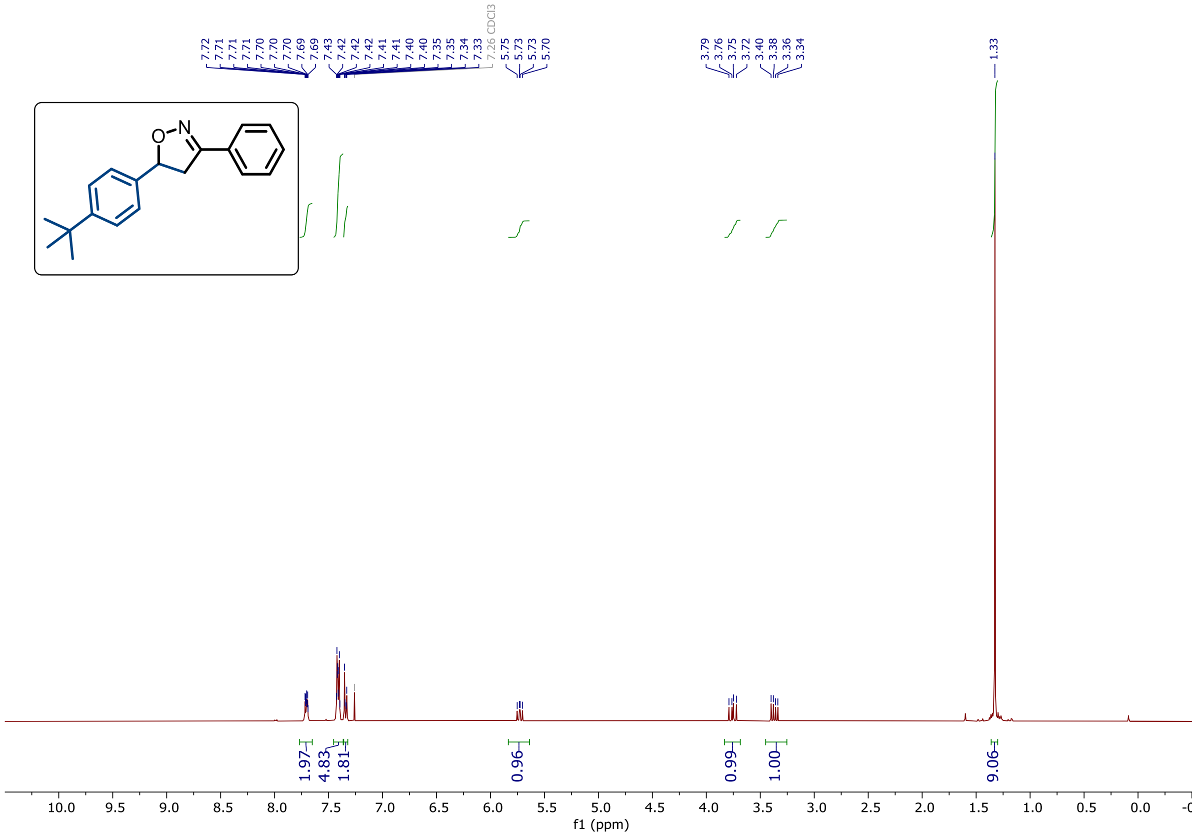

^{13}C-NMR (75 MHz) of Compound 4d

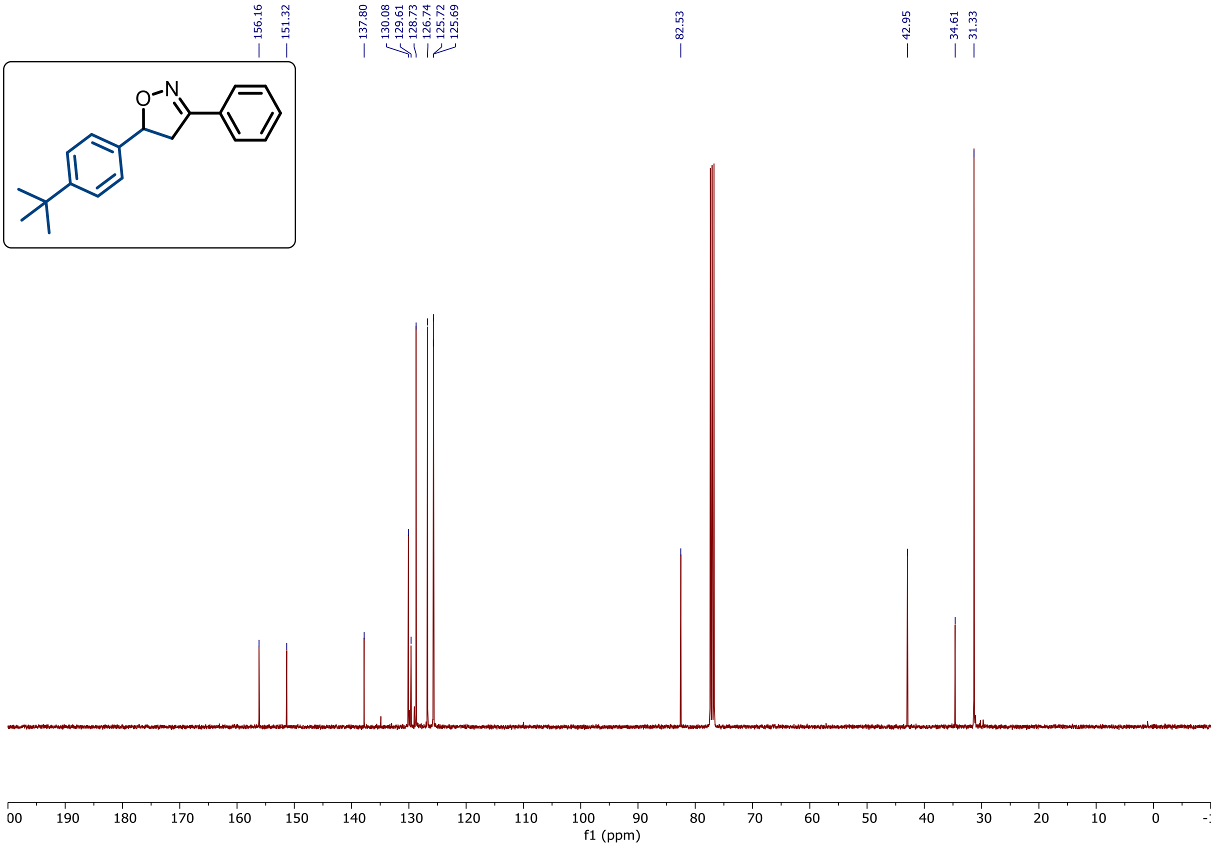

^{1}H-NMR (300 MHz) of Compound 6a

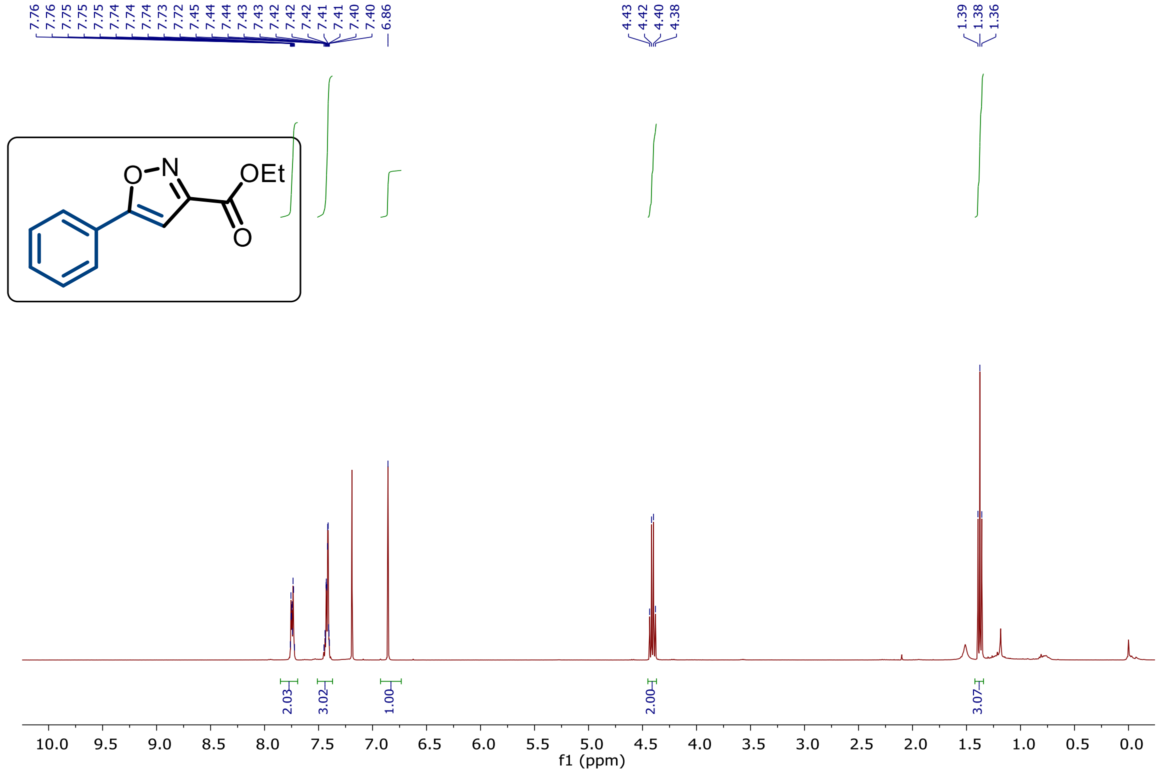

^{13}C-NMR (75 MHz) of Compound 6a

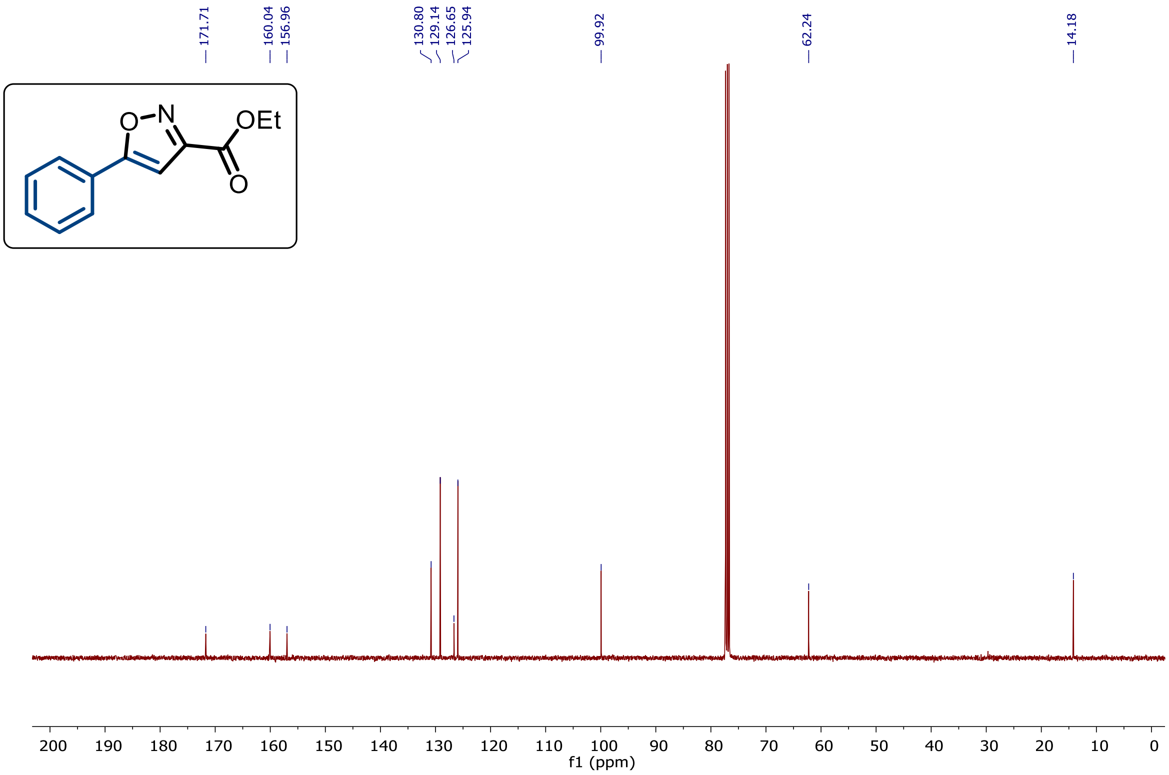

^{1}H-NMR (300 MHz) of Compound 6b

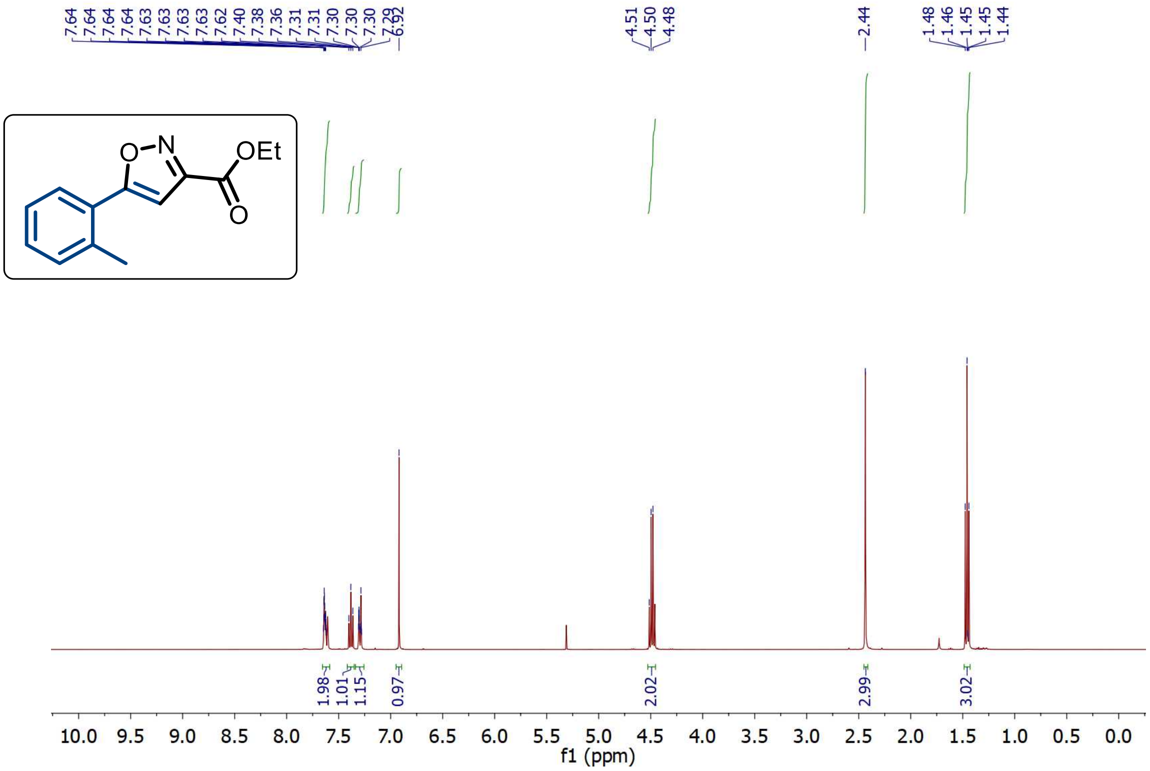

^{13}C-NMR (75 MHz) of Compound 6b

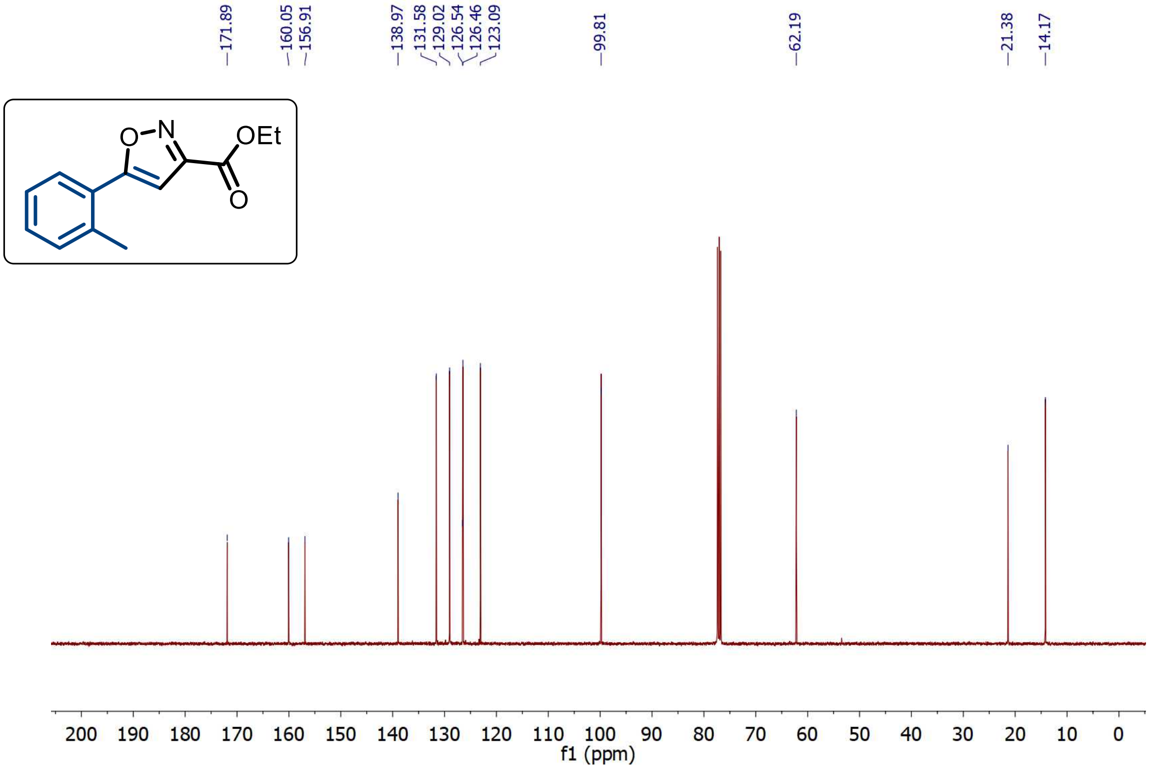

^{1}H-NMR (300 MHz) of Compound 6c

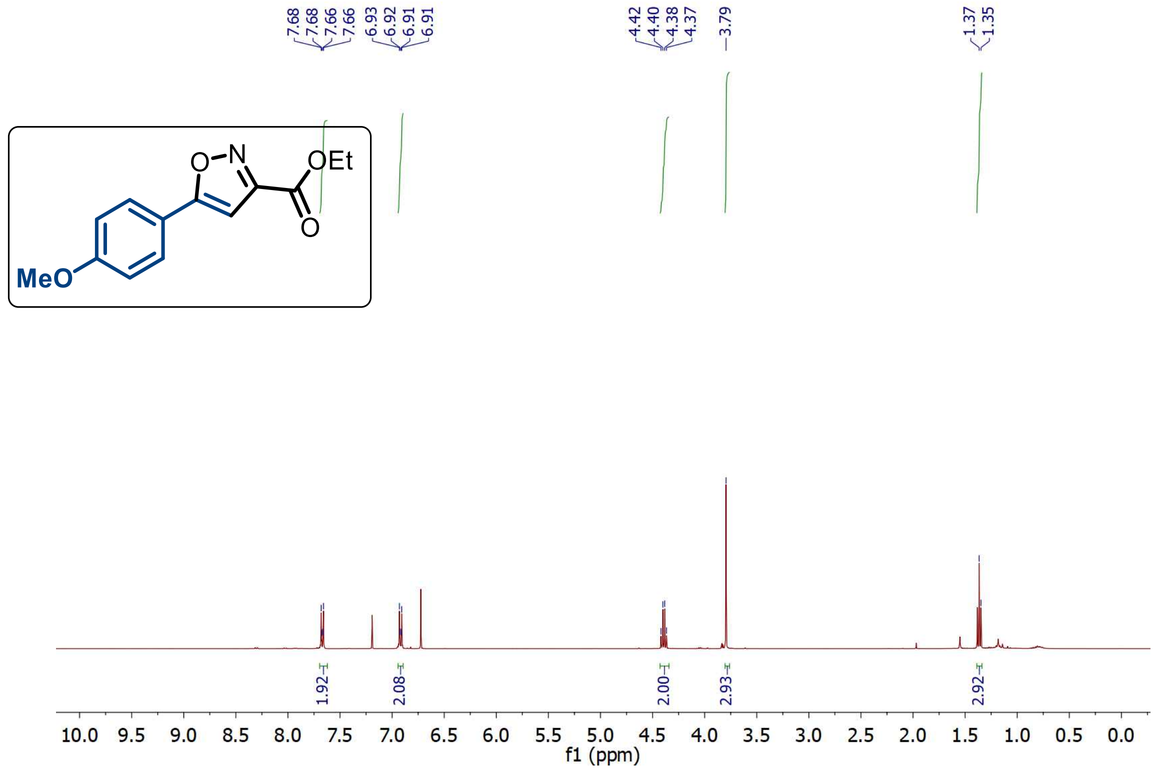

^{13}C-NMR (75 MHz) of Compound 6c

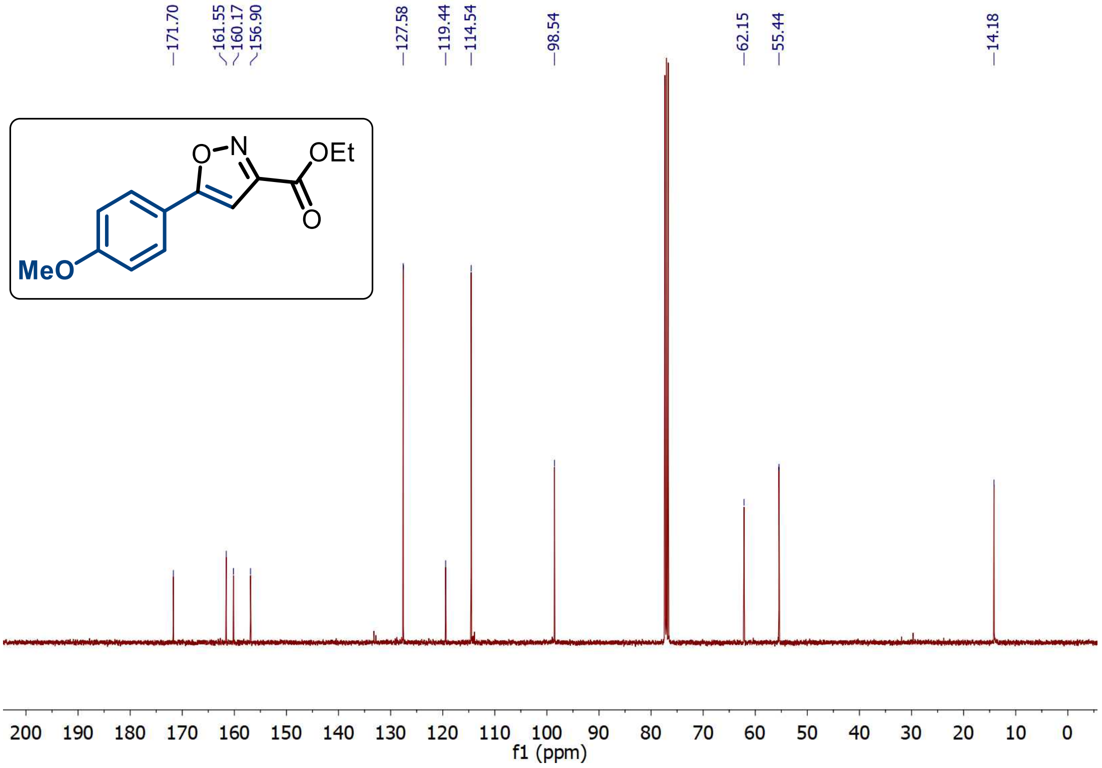

^{1}H-NMR (300 MHz) of Compound 6d

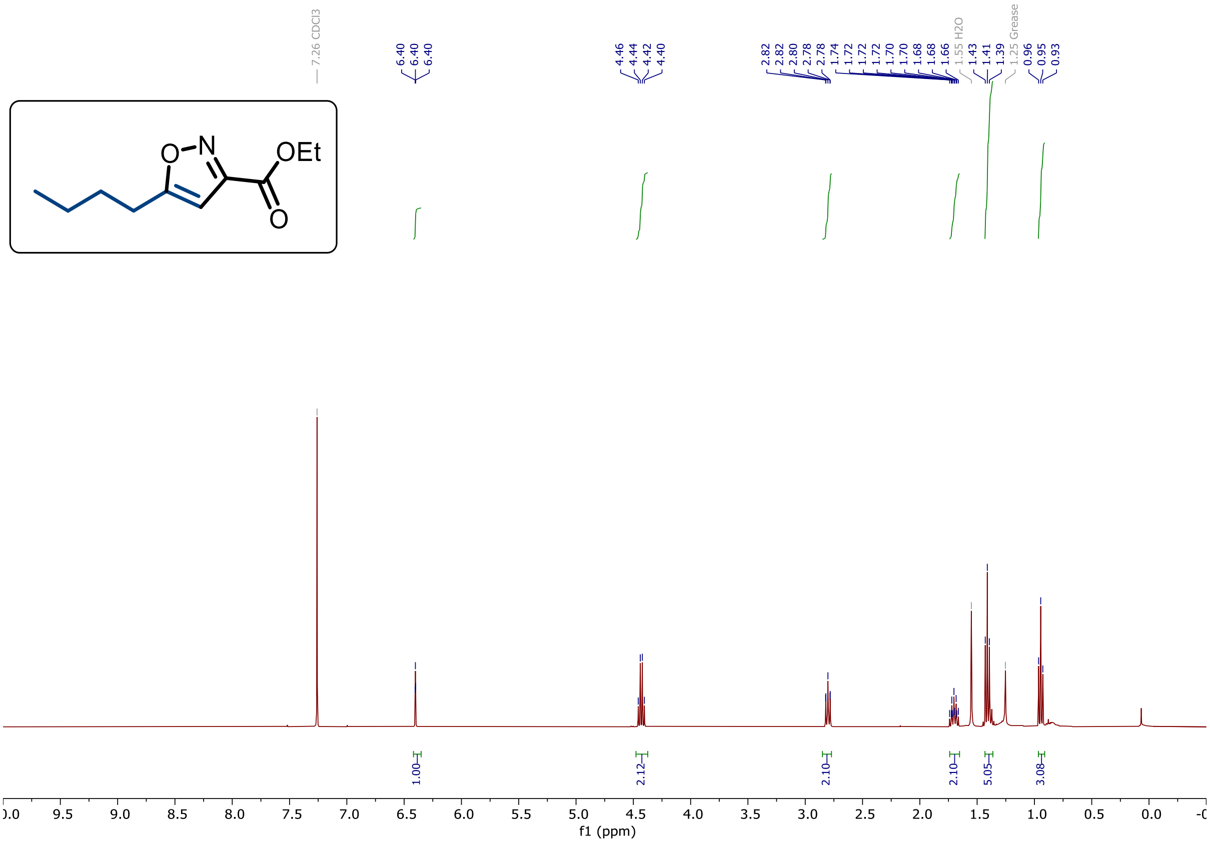

^{13}C-NMR (75 MHz) of Compound 6d

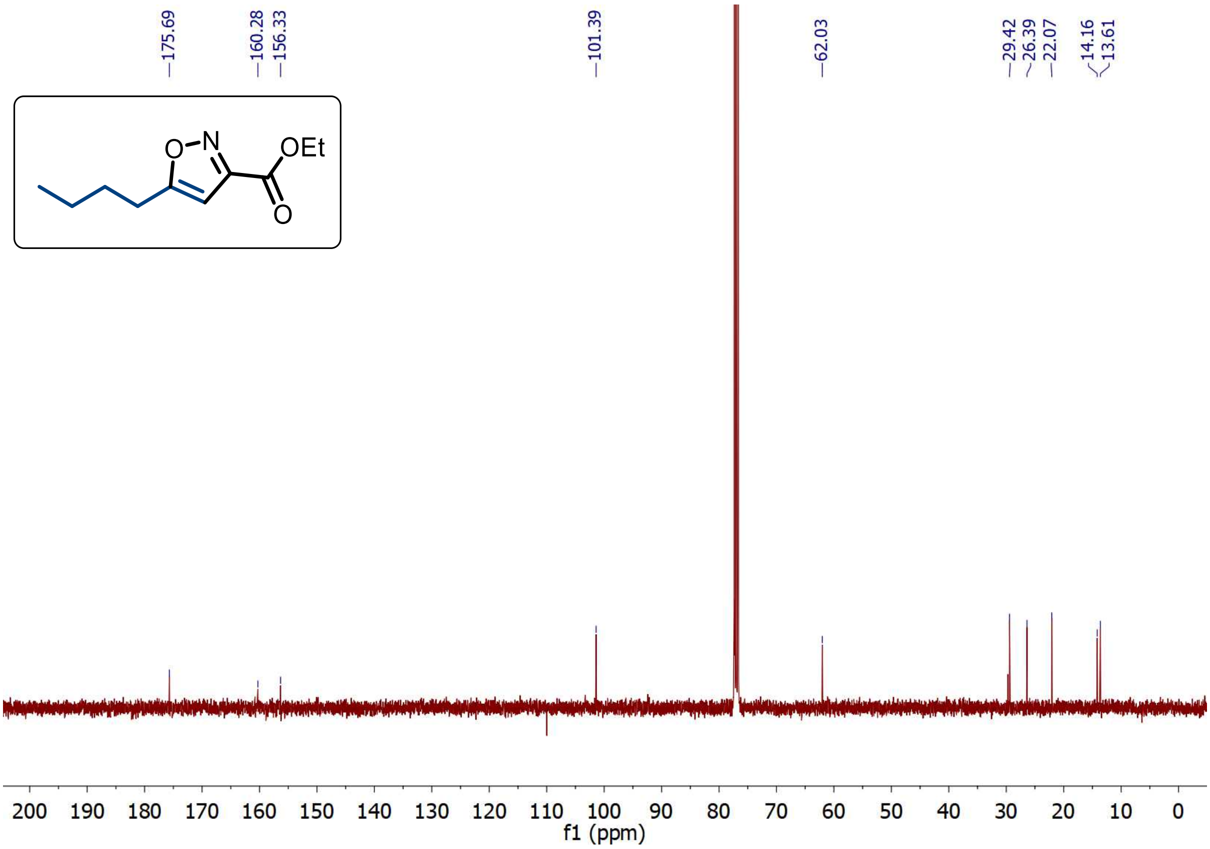

^{1}H-NMR (300 MHz) of Compound 6e

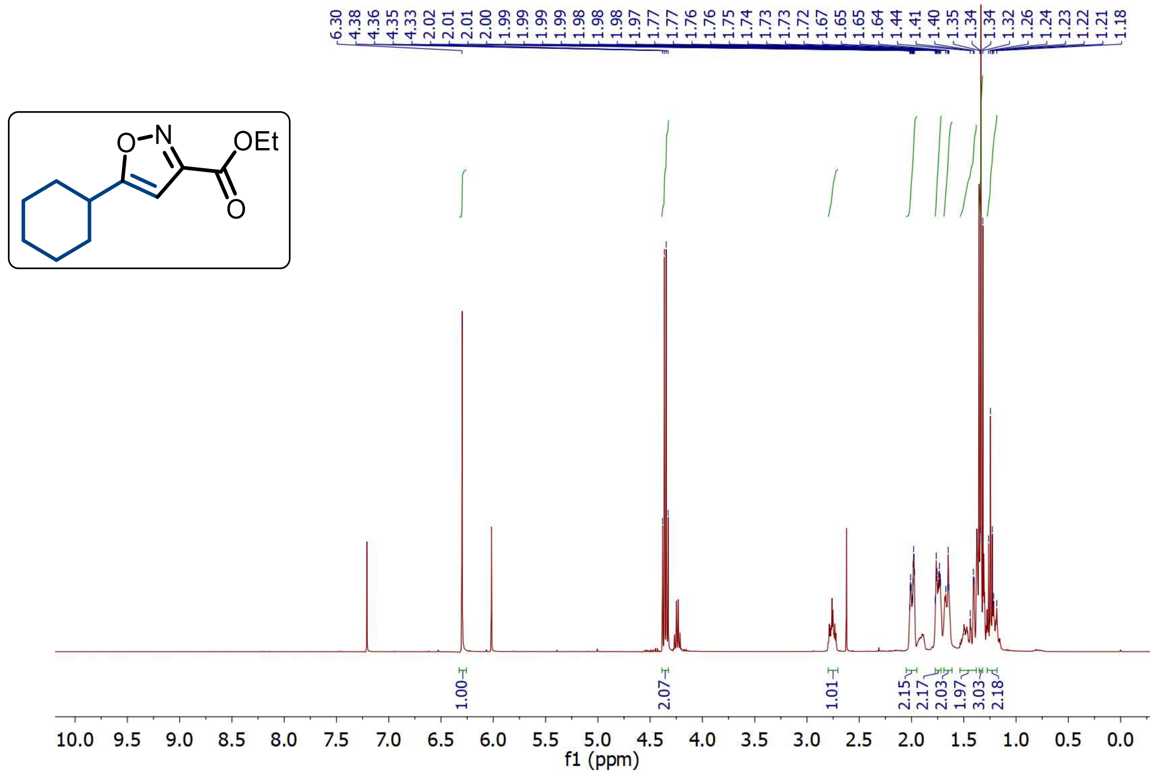

^{13}C-NMR (75 MHz) of Compound 6e

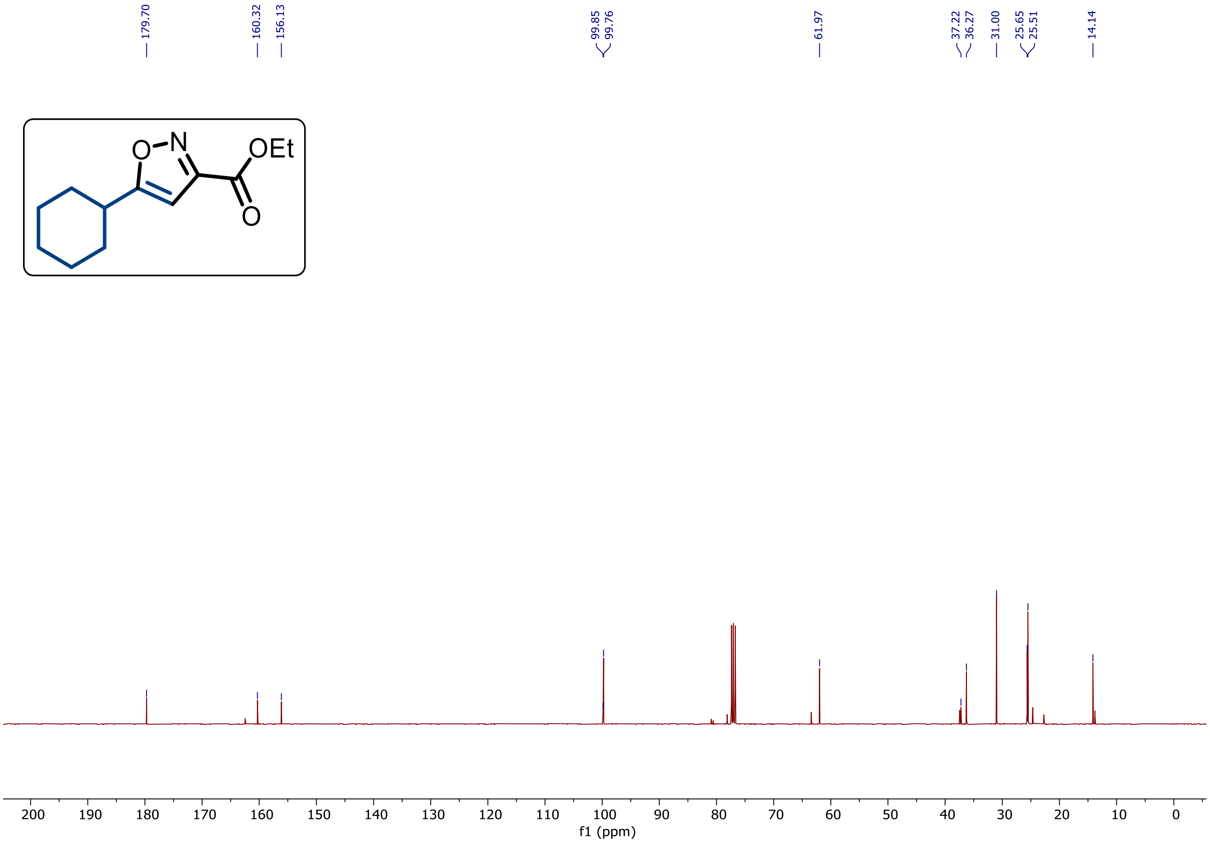

^{1}H-NMR (300 MHz) of Compound 6f

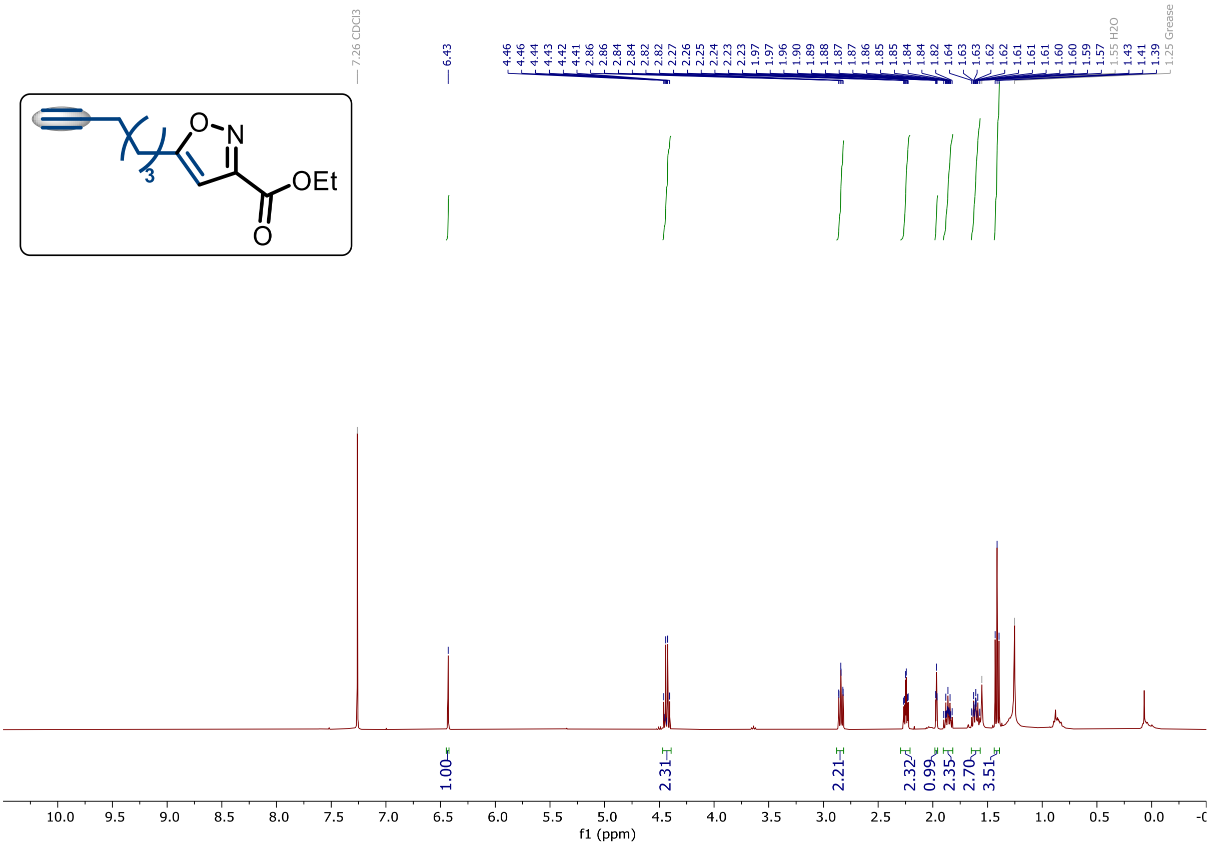

^{13}C-NMR (75 MHz) of Compound 6f

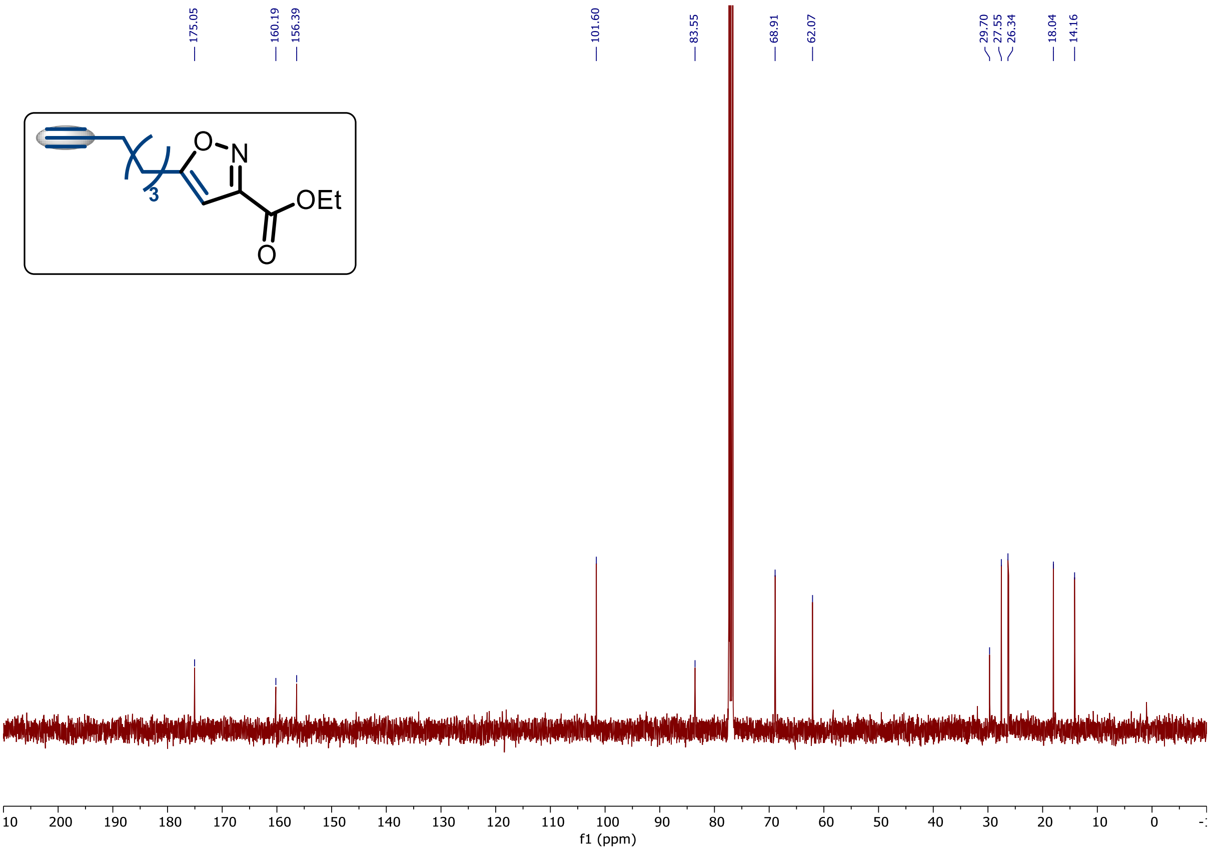

^{1}H-NMR (300 MHz) of Compound 6g

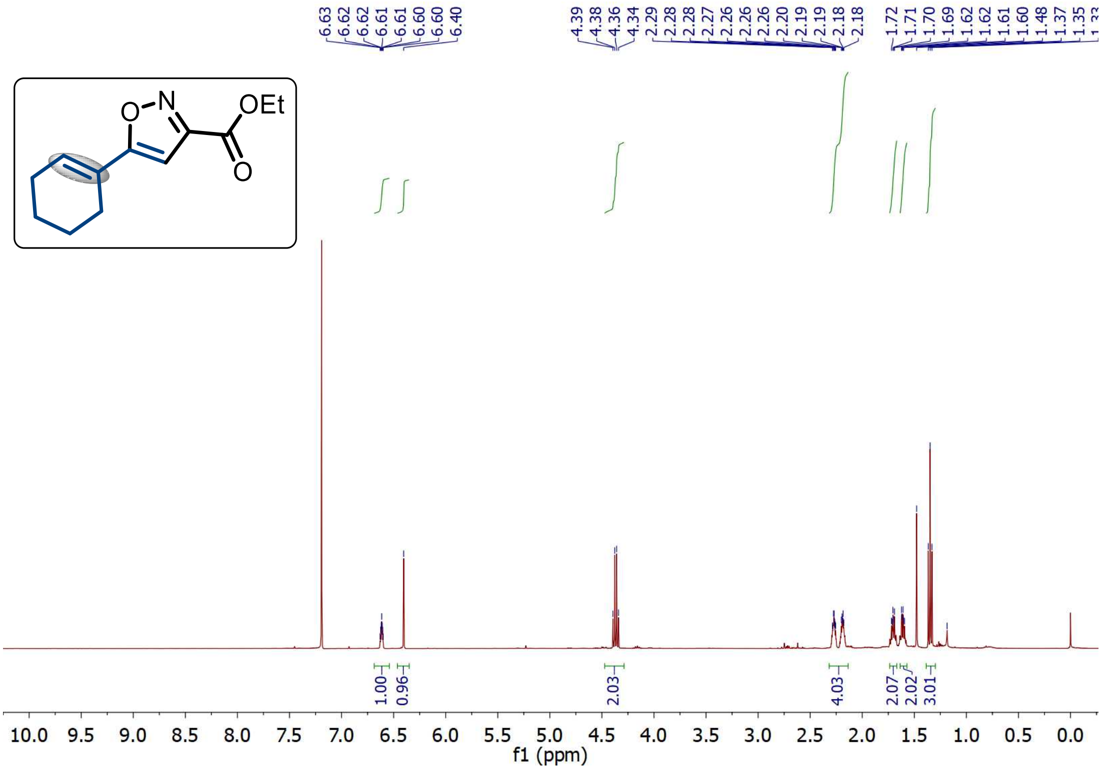

^{13}C-NMR (75 MHz) of Compound 6g

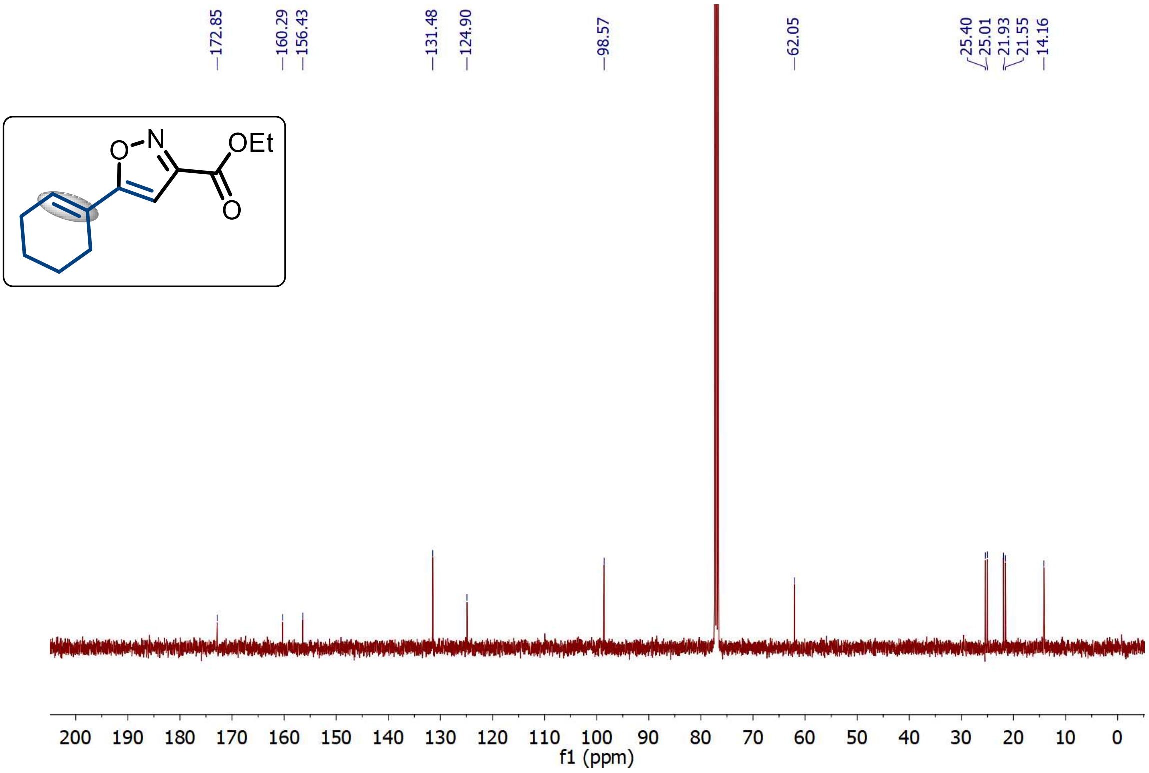

^{1}H-NMR (300 MHz) of Compound 3ah

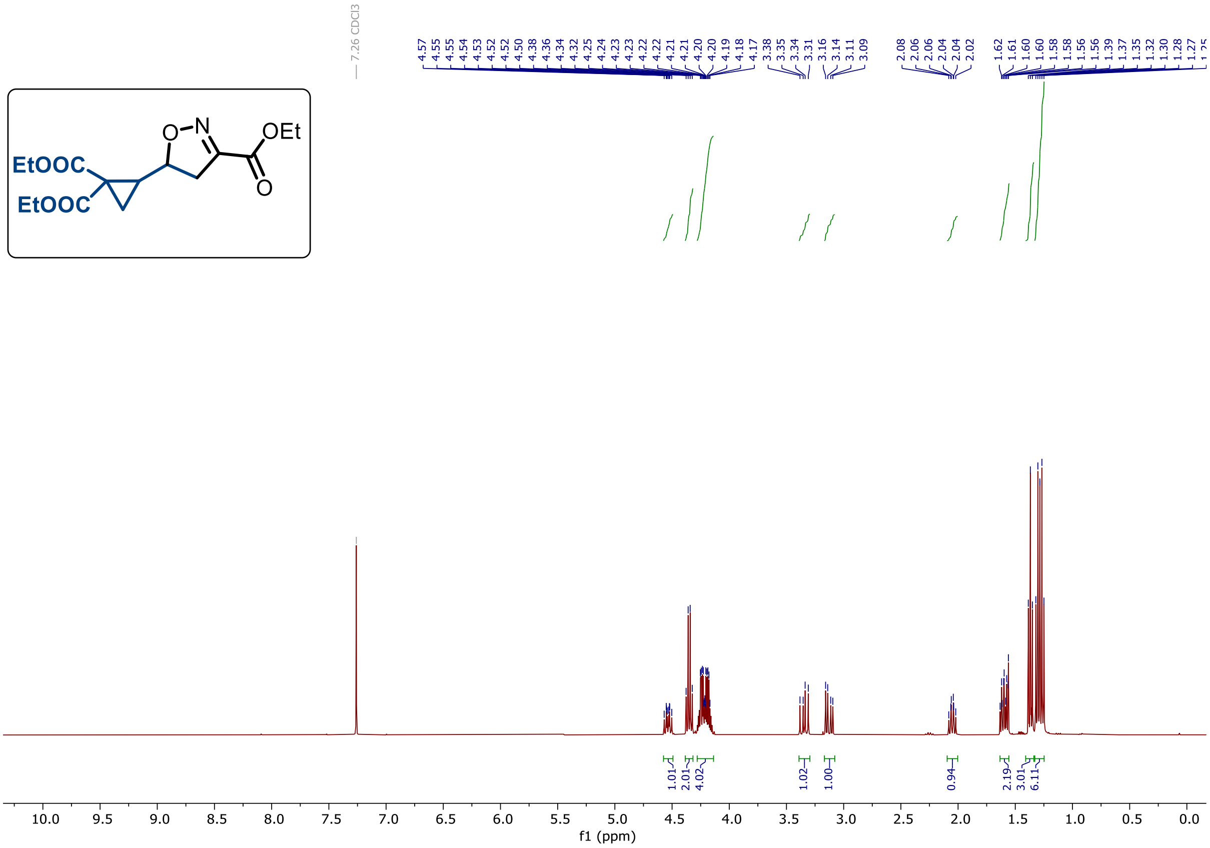

^{13}C-NMR (75 MHz) of Compound 3ah

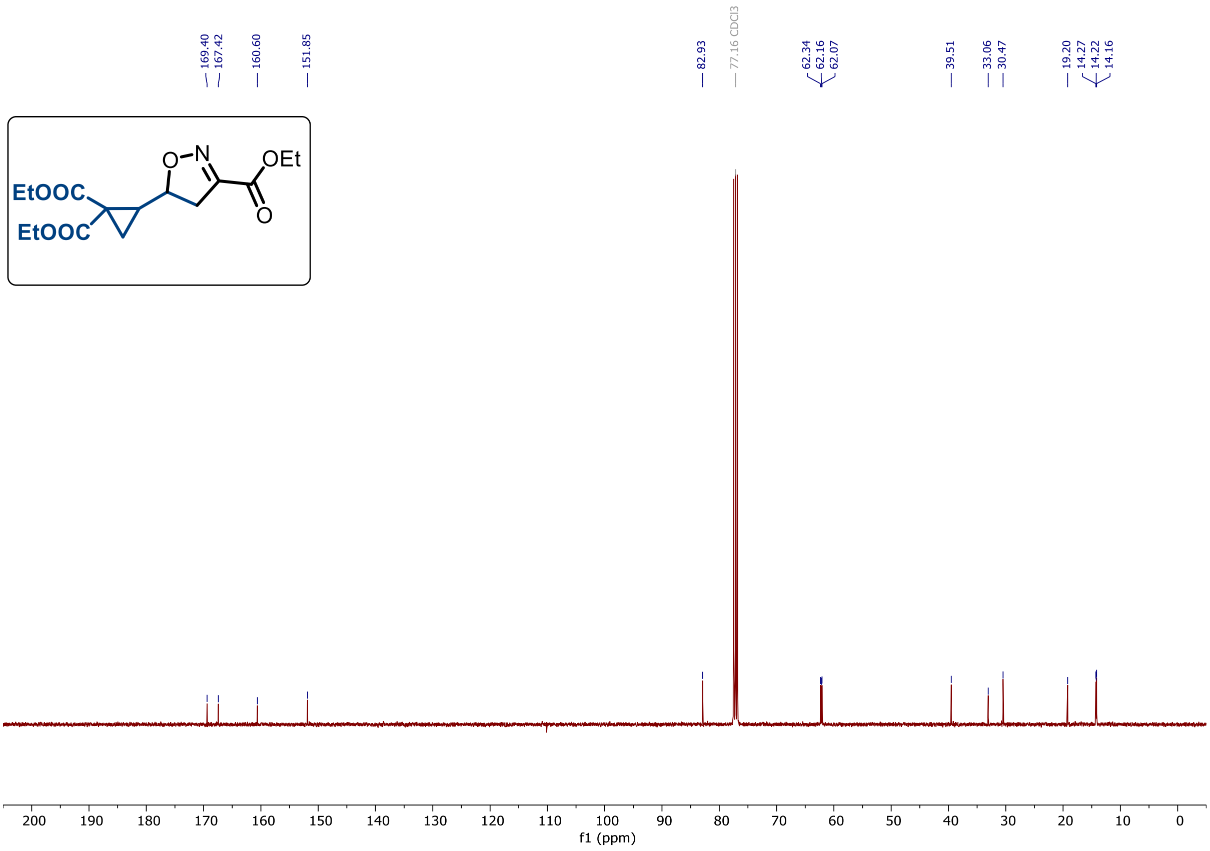

^{1}H-NMR (300 MHz) of Compound 3ai

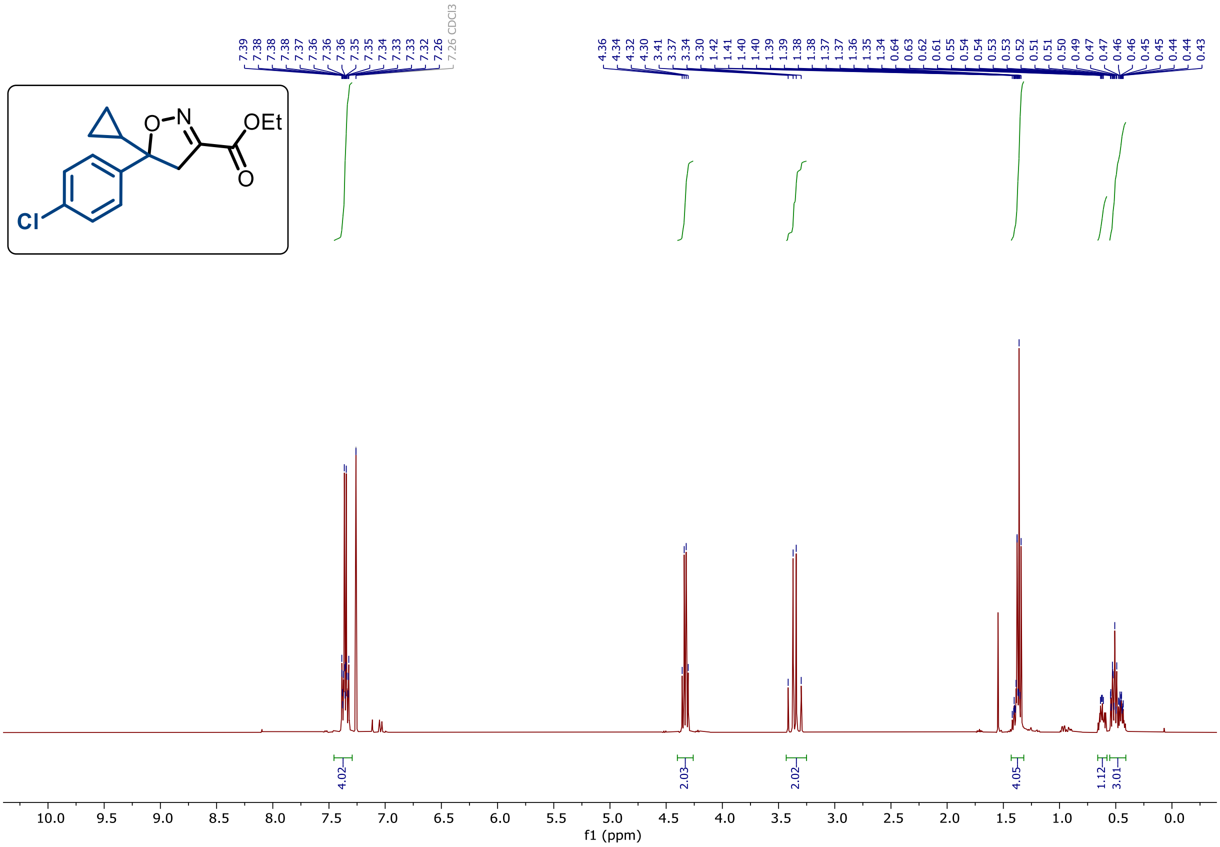

^{13}C-NMR (75 MHz) of Compound 3ai

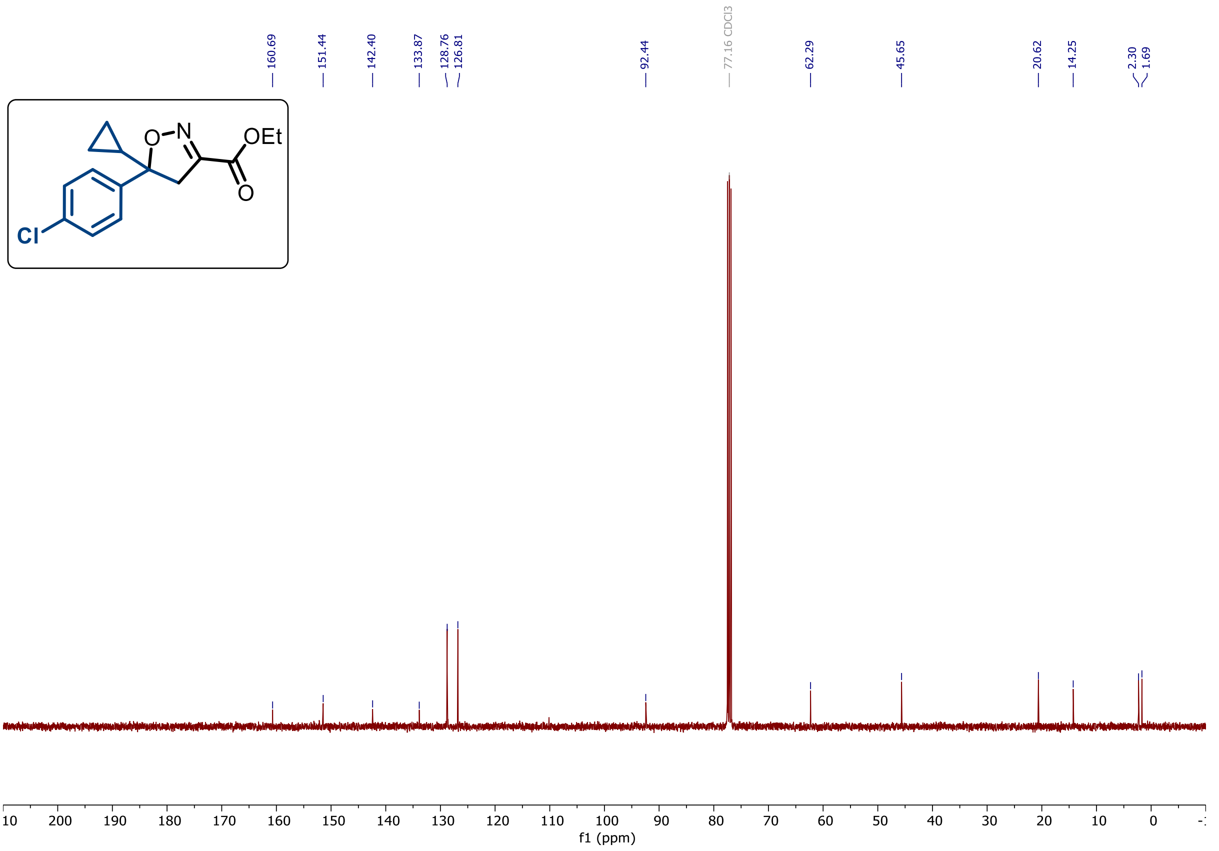

11.2 X- Ray Crystal structure of 3j

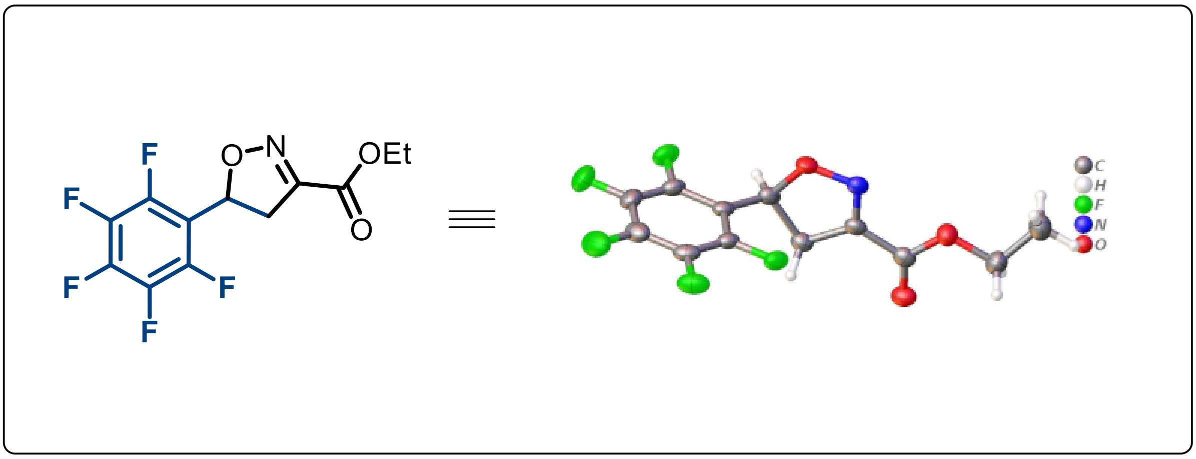

ethyl 5-(perfluorophenyl)-4,5-dihydroisoxazole-3-carboxylate (3j)

Crystal Data. $C_{12}H_8F_5NO_3$, M_r = 309.19, monoclinic, $P2_1/c$ (No. 14), a = 4.82200(10) Å, b = 27.1492(6) Å, c = 9.8277(3) Å, β = 104.174(3)°, $\alpha = \gamma$ = 90°, V = 1247.41(6) Å^3, T = 123.00(10) K, Z = 4, Z' = 1, μ(Cu K_α) = 1.494, 24059 reflections measured, 2368 unique (R_{int} = 0.0254) which were used in all calculations. The final wR_2 was 0.0784 (all data) and R_1 was 0.0322 (I≥2 σ(I)).

```
Bond precision:   C-C = 0.0022 A           Wavelength=1.54184

Cell:              a=4.8220(1)      b=27.1492(6)          c=9.8277(3)
                   alpha=90         beta=104.174(3)       gamma=90
Temperature:       123 K

                  Calculated                    Reported
Volume            1247.41(6)                    1247.41(6)
Space group       P 21/c                        P 1 21/c 1
Hall group        -P 2ybc                       -P 2ybc
Moiety formula    C12 H8 F5 N O3                C12 H8 F5 N O3
Sum formula       C12 H8 F5 N O3                C12 H8 F5 N O3
Mr                309.19                        309.19
Dx,g cm-3         1.646                         1.646
Z                 4                             4
Mu (mm-1)         1.494                         1.494
F000              624.0                         624.0
F000'             626.96
h,k,lmax          6,33,12                       5,33,11
Nref              2503                          2368
Tmin,Tmax         0.898,0.956                   0.921,1.000
Tmin'             0.836

Correction method= # Reported T Limits: Tmin=0.921 Tmax=1.000
AbsCorr = GAUSSIAN

Data completeness= 0.946             Theta(max)= 73.883

                                                         wR2(reflections)=
R(reflections)= 0.0322( 2092)                            0.0784( 2368)

S = 1.030                   Npar= 191
```
